# Life course trajectory segregation and political polarization in contemporary Sweden

**DOI:** 10.1038/s41467-026-75072-y

**Published:** 2026-07-30

**Authors:** Bo Malmberg, Juta Kawalerowicz, Eva K. Andersson

**Affiliations:** Stockholm University, Department of Human Geography, Stockholm, Sweden

**Keywords:** Geography, Sociology

## Abstract

Understanding how and why places differ geographically has long been central to human geography—a question sharpened by the growing geographical polarization of political views. Spatial polarization can result from an uneven geographical distribution of life-course trajectories, trajectories that emerge from individuals’ successive positions across interconnected, possibly spatially differentiated social fields such as education, employment, family, and housing. Here we show that the uneven geographical distribution of such trajectories produces landscapes where contrasting life experiences, opportunities, and aspirations become inscribed in geographical space. A key finding is that segregation intensifies over the life-course, with young adults substantially less segregated than older adults, particularly after homeownership transitions. Moreover, studies relying on single indicators such as income and education may underestimate residential segregation strength. Homeownership is a major segregation driver, creating highly separated residential areas in later life phases. The spatial concentration of trajectory types also corresponds with geographical patterns in political polarization.

## Abstract (web summary)

Where people live reflects their life paths in relation to education, employment, and family. This results in landscapes of contrasting life experiences that follow a metropolitan to rural gradient, also reflected in voting patterns.

## Introduction

Understanding patterns of spatial differentiation, that is, how geographical places differ from one another and why, has long been a central concern in human geography. This question has become particularly relevant as political views, in addition to being influenced by class, show increasing geographical polarization^1^. Explanations for geographical polarization have taken several forms. Some scholars emphasize residential segregation, whereby individuals with similar political views cluster together^2^. Others focus on structural economic changes that create left behind places characterized by industrial decline and reduced opportunities^3,4^. Still others examine how place-based identities and resentment develop in response to perceived neglect^5,6^.

At any given point, individuals simultaneously occupy positions in multiple social and spatial life domains, creating chains of interconnected life-course positions over time^7–9^. Such domains can be seen as corresponding to what Bourdieu calls social fields that are characterized by specific forms of practice and have their own logic and rules of the game. For Bourdieu, a trajectory is a series of positions successively occupied within a social field over time, essentially a sociological career path that shows how individuals move through structured positions within interconnected fields like education, employment, families, and housing^10^. The way individuals attain positions in a field is influenced by their habitus, that is, dispositions formed by socialization and consisting of both inclinations and their specific cognitive frameworks. The attainment of positions will also depend on resources, making cross-domain effects important for the way one’s life-course trajectory unfolds^8^. Regional and local housing systems can also be understood as social fields where individuals struggle for positions^11^. For the present paper, this idea is helpful, since it transforms Bourdieu’s largely aspatial understanding of trajectories into a theory of how geographical places are formed. One’s position in a regional housing system will be characterized not only by type of tenure and type of dwelling but also by a specific location. Since the position one attains in the local housing systems will be correlated with the position one attains in other domains, one can expect individuals following similar trajectories, and thus sharing habitus, to be sharing a neighborhood too. Complementary perspectives on the formation of life course trajectories include the role of institutional channelling^12^, cognitive ability^13^, health^14–16^, and childhood adversity^17,18^.

Bourdieu’s assumption that trajectories are formed by differentiated dispositions and by the structured positions that constitute a field resonates well with latent class analysis (LCA), the statistical approach that we will use to identify life-course trajectories. In LCA, it is assumed that the probability of attaining a position that corresponds to specific values on a set of observed indicator variables is determined by one’s membership in an unobserved latent class. We maintain that Bourdieu’s idea of social trajectories implies that there will exist patterns in life-course trajectory data that corresponds to specific trajectory types, and that these typical trajectories can be identified using LCA. Expressed differently, that by applying LCA to trajectory data, we can empirically identify social trajectories that reflect both the differentiated dispositions and the structured positions available to them within social fields. Trajectories can also be identified using sequence analysis. A prominent example is provided by Fasang and Aisenbrey. They employed multi-channel sequence analysis to examine relationships between family trajectories and employment trajectories in data from the US National Longitudinal Survey of Youth^19,20^.

Because of cross-domain correlations, we expect the identified trajectories to be differentially represented across geographical space. These correlations reflect a dynamic process: neighborhood contexts shape individual characteristics like employment and income that subsequently influence residential mobility, creating feedback loops where initial neighborhood disadvantage constrains future residential options^21^. The spatial clustering of trajectory types thus reflects both selective residential mobility and cumulative neighborhood effects over the life course. In this way, Bourdieu’s social trajectories become geographically embedded. Bourdieu acknowledges that even though individuals that occupy the same positions in social space are likely to have the same habitus^10^, it is still the case that individual and collective representation that agents may acquire of the social world and of their place in it may well be constructed according to completely different categories (p. 8). We argue, however, that location matters. When people with similar life trajectories also live in the same geographic area, a shared trajectory type is more likely to translate into shared ideas about social identity.

The analysis of social trajectories presented by Bourdieu suggests that typical life-course trajectories are meaningful social constructs. At the same time, an LCA-solution will reproduce the response patterns found in indicator variables in an optimal way. LCA of multi-domain, longitudinal data, thus, will generate categorizations that make individuals recognizable as instances of typical social trajectories. This contrasts with categorizations built on single variables such as income, education, or ethnicity, for which within-category variation can be high.

Modern geo-demographics provides sophisticated tools for analyzing population composition patterns, with applications ranging from public health research and urban planning to marketing strategies^22^. Recent work demonstrates how these methods can identify complex relationships between demographic characteristics and spatial distribution in contemporary cities^23,24^. Theoretically, the spatial patterns studied by geo-demographers can be seen as the result of selective migration, for example, related to family status^25^ and to health^26^, but can also be related to the effects of local context^27,28^ and of mobility^29,30^ on life-course trajectories. Current approaches build upon a rich tradition of studying spatial differentiation in urban sociology. The theoretical foundations trace back to the Chicago school of sociology’s human ecology framework^31^, which pioneered the integration of case studies with statistical analysis of urban structures. A crucial methodological evolution occurred through the work of Shevky and Bell^32^, who shifted the field toward more systematic analysis using census-based area statistics and multivariate classification methods. Subsequent researchers like Murdie^33^, and Berry^34^ further developed these techniques, revealing distinct spatial patterns organized around life cycle stages (showing concentric patterns relative to city centers), socioeconomic status (manifesting in sectoral patterns radiating from city centers), and minority group concentrations (displaying nucleated patterns).

The novelty introduced with a trajectory approach lies in how individuals are represented. In geodemographics, individuals influence the classification of an area by their contribution to the averages of the different indicators. In the trajectory approach, individuals are instead represented as belonging to or associated with different types of life-course trajectories. Areas will differ with respect to their composition in terms of such trajectories, and in this way, “individual trajectories leave traces that produce different types of space and shape how geographic space evolves” (translated)^35^. The life-course trajectory representation thus expresses areal differentiation as a resulting from different types of individuals residing in different areas. It will allow us to characterize places based on which life-course trajectories that are present locally. In geodemographics, this link is less direct. Moreover, as has been demonstrated in earlier work^36^, the life-course trajectory representation can be connected to qualitative life-course research, and in this way the trajectory approach opens for a mixed methods approach to the analysis of areal differentiation^37^.

Here, we explore whether our theoretical framework can be implemented empirically. Using Swedish geocoded longitudinal register data covering the entire population from 1990 to 2019, and applying latent class analysis across multiple life domains and five successive life phases, we identify 25 distinctive life-course trajectory types and analyze their spatial distribution across Sweden. We demonstrate that these trajectories are more strongly separated in residential space than classifications based on single indicators such as income or education. We show that segregation intensifies markedly over the life course, driven primarily by the transition to homeownership in middle age, and that the resulting geography of trajectory types closely mirrors patterns of political polarization—suggesting that place-based political identities are rooted in the shared lived experiences that become concentrated within neighborhoods.

## Results

### Life-course trajectories

**Fig. 1:**
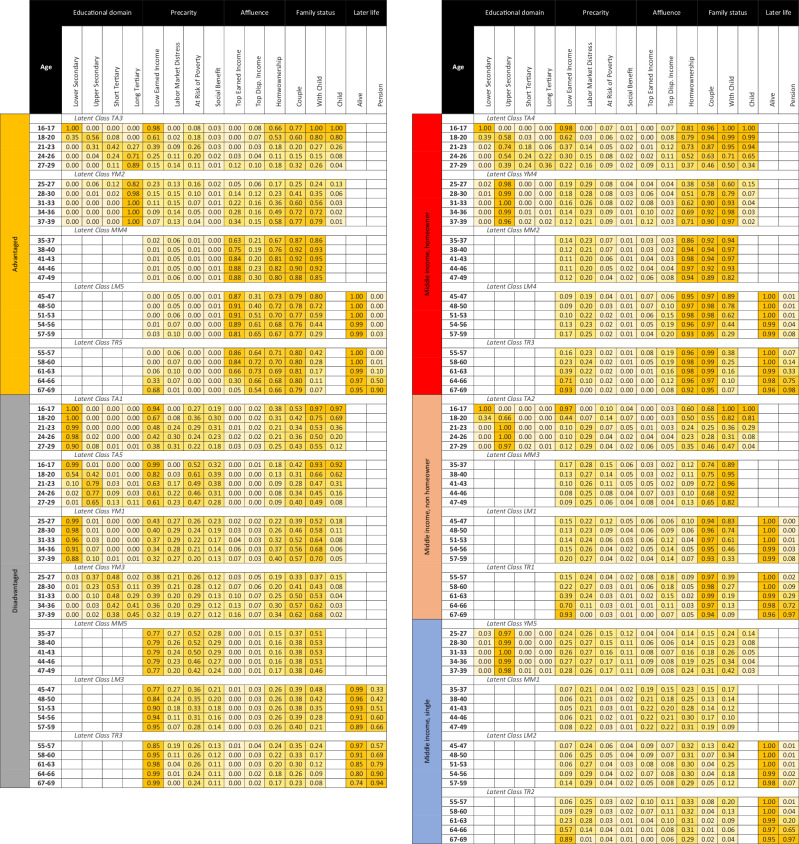
Proportions of life-course states (average proportions of three-year age brackets) for 25 trajectory types across five life phases: Transition to adulthood (TA) 16–29 years. Young middle-age (YM) 25–39 years. Middle middle age (MM) 35–49 years. Late middle age (LM) 45–59 years. Transition to retirement (TR) 55-69 years. Educational progress represented by four state variables, columns 1–4: Completed lower secondary, completed upper secondary, completed short tertiary, and completed long tertiary. Precarious life situation, columns 5–8: Low earned income, labor market distress, at-risk-of-poverty, social benefit. Affluence, columns 9–11: Top earned income, top disposable income, and homeownership. Family status, columns 12–14: Couple, with child, child. Later life indicators, column 15–16: Alive, pension. Color of row heading indicate trajectory type: Gold for Advantaged, Red for Middle-income homeownership, Peach for Middle-income non-homeownership, Blue for Middle income single, and Gray for Disadvantaged. The profiles are based on an analysis of trajectory data for the years 1990–2019.

In Fig. 1, the characteristics of the 25 life-course trajectories we have identified are presented using graded yellow tones to represent the proportion of the members in each latent class that are in different states. By comparing yellow tones within life phases, the distinguishing traits of each trajectory can be observed.

**Fig. 2:**
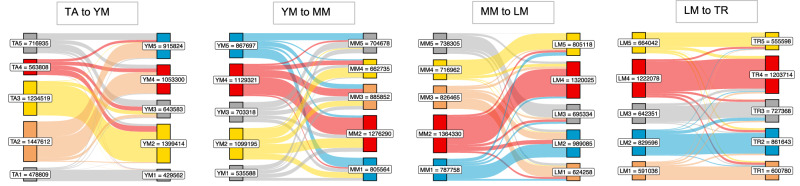
Sankey charts for trajectory transitions between life phases. TA: Transition to adulthood 16–29 years. YA: Young middle-age 25–39 years. MM: Middle middle age 35–49 years. LM: Late middle age 45–59 years. TR: Transition to retirement 55–69 years.

**Fig. 3:**
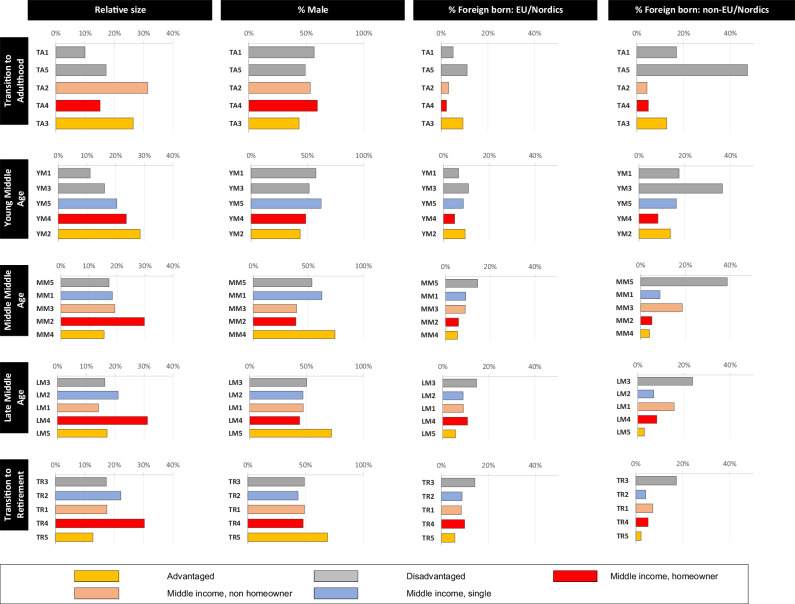
Descriptive statistics for latent classes. Grey = disadvantaged trajectories, blue = middle-income single, apricot = middle-income non-homeownership, red = middle-income homeownership, gold = advantaged.

**Fig. 4:**
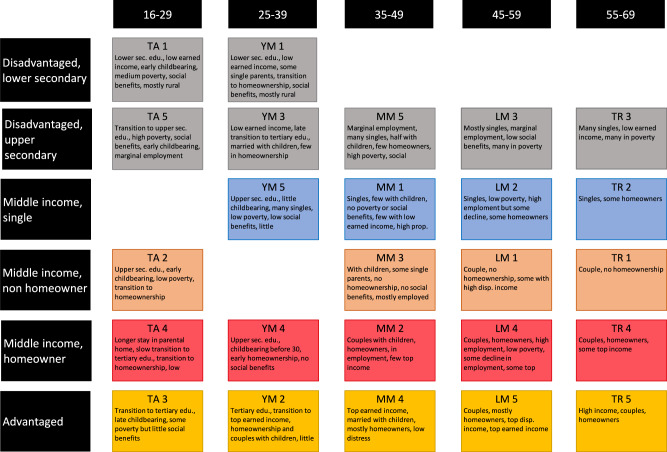
Overview of trajectories identified in latent class analysis. By assigned stratification group. Descriptions based on proportions in different life-course states. Grey = disadvantaged trajectories, blue = middle-income single, peach = middle-income non- homeownership, red = middle-income homeownership, gold = advantaged. Missing cells indicate that none of the trajectories identified in a life phase fitted within a certain stratification group.

An important result of the analysis presented in Fig. 1 is that the trajectories can be interpreted as belonging to different stratification groups. In each life phase, it is possible to find disadvantaged trajectories, marked grey, in younger life phases characterized by having a short education, in older life phases by high proportions of low earnings, high proportions at risk of poverty, high proportions of social benefit recipients, early retirement, and higher mortality. Similarly, across life phases, it is possible to identify advantaged or upward trajectories, marked yellow, in younger life phases characterized by having or transitioning to a long tertiary education, in older life phases by high proportions of top earnings, of top income, and of homeownership. Middle-income, homeownership trajectories constitute a third type of trajectory, marked red, characterized by having an upper secondary school education (in younger life phases), high rates of homeownership, being in a couple, having children, but little top earning or top income. In contrast, for three older life phases we can also distinguish a middle–income non–homeownership trajectory type, marked peach, with children in the household, living in couples, but little top income. In the four oldest life phases, there is also a trajectory type with singles, marked blue. Membership in a stratification group is relatively stable across life phases, especially after 35 years of age, see Fig. 2. As shown in Fig. 3, the numerically largest trajectory types in the older life phases, MM, LM, and TR, are the middle-income homeowners. Middle-income homeowners and middle-income non-homeowners together constitute close to 50% of the individuals. This suggests that there is not much hollowing out of the middle class in Sweden, a possible consequence of the Swedish welfare state^38^. Fig. 3 also shows that the gender composition of the trajectories changes across life phases, with men becoming increasingly dominant in the affluent trajectories. This suggests that in the flows depicted in Fig. 2, there can be a gender imbalance for flows into or away from affluence. For additional information about the trajectory types, see Fig. 4. Plotted trajectory profiles for the different life phases are presented in Supplementary Figs. 2–6.

If it is read column-wise, that is, indicator by indicator, Fig. 1 gives an overview of life-course patterns in different domains. Thus, during the 16–29 life phase, differences in educational attainment open up, differences that stabilize during the 25–39 life phase. In relation to the indicators of precarity it can be noted that the incidence of social benefit and being at risk of poverty is uneven already before age 20. The incidence is high in several trajectory groups in the 25–39 life phase, but around age 40 it becomes concentrated to the most disadvantaged group. Low earned income develops in a similar way, but here there is more of a gradient across trajectories in the same life phase. Overall, the indicators of precarity in general delineate disadvantaged trajectories. The disadvantaged group also experiences an early increase in pension receipts, as well as an early tick-up in mortality (proportion alive drops below 0.9 after 56 years of age). With respect to the indicators of affluence, differentiation opens only after age 30 and becomes especially pronounced around 50 years of age, suggesting an influence of cumulative advantage. Differences within life phases with respect to the family status indicators are high already in the transition to adulthood life phase, they are somewhat reduced in the young middle age phase, but become substantial again in the transition to retirement life phase. Homeownership is somewhat differentiated already before age 30 and becomes increasingly so after age 35. Being in a couple is also differentiated between trajectories, with not in a couple being a clear minority status. As can be expected, having a child in the household becomes a high incidence status around age 35 and into the late 40 s, again accompanied by one trajectory type where individuals live in childless households. Figure 1, thus, demonstrates how trajectories cut across different life domains, making stratification intersectional^19^.

The trajectories identified in the analysis of latent classes capture considerable variation across several life domains, making the trajectories clearly distinct. As argued above, these differences are likely mirrored in different residential patterns.

### Spatial segregation of trajectory types

**Fig. 5:**
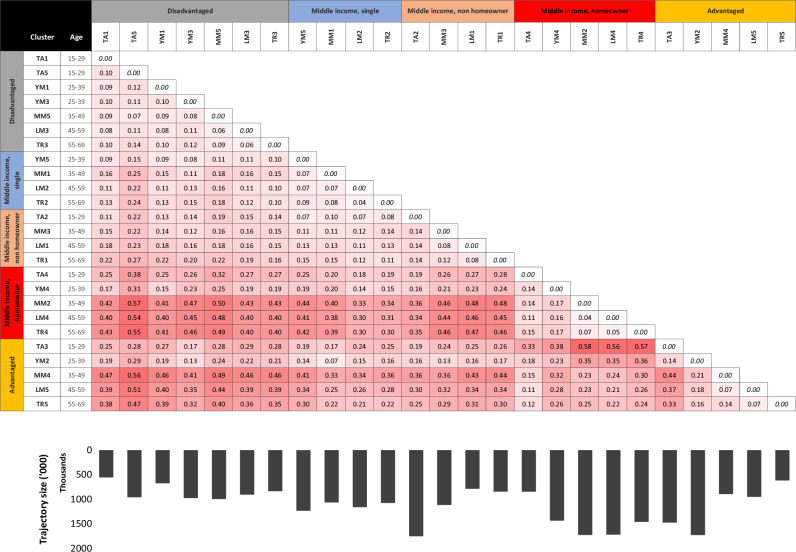
Separation index between trajectory types. High values in darker red, low values in lighter red. Based on the proportion of individuals in different trajectory types among the *k* = 400 nearest neighbors. Disadvantaged trajectories are in top rows and in the left-hand columns. Disadvantaged trajectories are followed by singleton trajectories, middle-income non-homeownership trajectories, middle-income homeownership trajectories, and advantaged trajectories. Within these groups, the trajectories are ordered with the youngest life phase first and the oldest life phase last.

The level of separation of different trajectory types is presented in Fig. 5, using the separation index as the measure of residential segregation^39^. In Fig. 5, trajectories have been ordered according to stratification groups, implying that separation values found along the diagonal are for trajectories that have similar socio-demographic characteristics. Within stratification groups, trajectories have been ordered by life phase. Here, darker shade indicates high values for the separation index, implying that two groups, to a large extent, are found in different residential areas. Lighter shade indicates low values on the separation index, which reflects that two groups tend to co-reside in the same local areas. The local area here is defined as consisting of the 400 nearest neighbors. As can be seen in the figure, separation values along the diagonal are generally low, implying that trajectories that have similar socio-demographic characteristics tend to be co-residing.

**Fig. 6:**
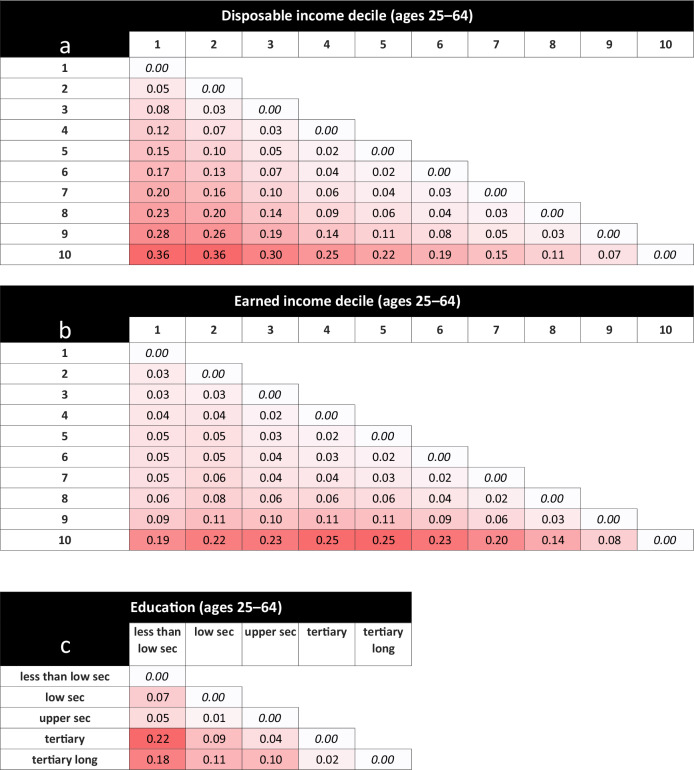
Separation index for single variable categories. Separation index in 25–64 population for (**a**) educational groups, **b** disposable income deciles, **c** labor earnings deciles. Based on proportions in *k* = 400 neighborhoods in 2019.

Supplementary Figs. 7–31 provides maps showing the spatial distribution of individuals belonging to the different types of life-course trajectories. These maps show that trajectories that have low levels of separation are very similar, whereas maps for trajectories with high levels of separation are very different. Overall, these maps also show that the different trajectories display very distinct geographical patterns, lending support to the idea that classifications based on multiple indicators over time identify groups affected by similar locational forces. This idea is given further support if we compare the separation indices for pairs of trajectories with separation indices for classification based on single variables, see Fig. 6. Thus, the highest separation index for individuals with different levels of education is 0.22, for earned income deciles, the highest value is 0.25, and for disposable income deciles, the highest value for the separation index is 0.36. In contrast, for different trajectory types the separation index can reach as high as 0.58, and for substantial number of trajectory pairs it is higher than 0.40. This suggests that there is a risk that segregation measures for single variable classification underestimate the strength of segregation processes that affect individuals following typical life-course trajectories, and that a trajectory-based understanding of segregation can be helpful for designing and evaluating anti-segregation measures.

Across the three oldest life phases, middie-income homeownership trajectories and advantaged trajectories tend to be separated from most other trajectory types. Separation between middle-income homeownership trajectories and advantaged trajectories in the later life phases is relatively low.

A shared characteristic of these trajectories is that they have very high levels of homeownership, from close to 70% to above 95%. Thus, their spatial separation reflects the existence of homogeneous homeownership residential areas, and thus, a transition to this type of housing results in a spatial separation from other trajectory types.

What is interesting is that the segregation patterns for the two youngest life phases are less clear-cut. During transition to adulthood and early middle age, the advantaged life-course and the middle-income homeownership life course are much less separated than they become in later life phases. This is because they are at a stage in their housing careers that involves living in neighborhoods with a population that is more mixed with respect to different life-course trajectories.

The advantaged trajectory of the 16–29 life phase is moderately to strongly separated from other trajectories, especially in relation to later life advantaged life courses and later life middle-income homeownership life courses. This probably reflects participation in tertiary education and concentration to residential areas where other students live.

In contrast, the middle-income homeownership trajectory of the 16–29 life phase shows little separation from later life advantaged and later life middle-income homeownership, possibly because they stay in their parental home until after 25 years of age.

Figure 5 demonstrates that our classification of life courses results in a typology of trajectories that display very distinct patterns of geographical differentiation. We see this as related to the fact that the identified trajectories differ with respect to a range of characteristics that are of importance for residential segregation. Another important finding is that for advantaged trajectories and for middle-income homeownership trajectories, patterns of segregation vary over the life course. During later life phases, these groups are strongly segregated, but not necessarily during the transition to adulthood and during young middle age. There have been studies showing that becoming a parent is associated with mobility out from ethnically diverse neighbourhoods^40,41^. But we can generalize this finding and propose that for advantaged groups, residential experience before 35 years of age can be much more diversified than residential experience after age 40.

However, what Fig. 5 also shows is that spatial separation is only strong for, and in relation to, advantaged trajectories and middle-income homeownership trajectories in later life. For other trajectory types, segregation is low to modest. This is in line with US findings of high levels of income diversity in many neighborhoods^42^ but speaks against an idea of deep and persistent inequality among^43^ neighborhoods as a general pattern. Clearly, there is substantial social mixing, even if this mixing does not involve separated homeownership trajectories.

### Geographical patterns

As outlined in our theoretical considerations, we understand the spatial separation of trajectory types to result from how the position one holds in different life domains is reflected in the geographical position that individuals attain in regional housing systems.

**Fig. 7:**
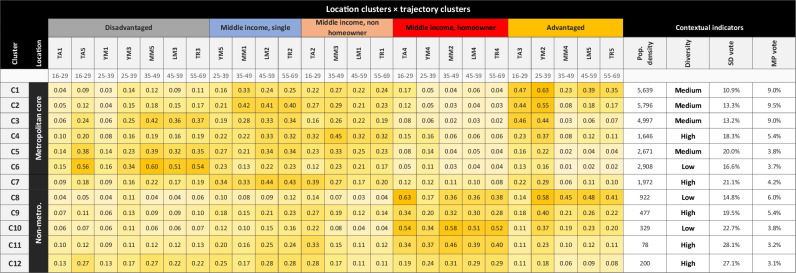
Average trajectory composition of neighborhood populations in *k* = 400 neighborhoods for 12 different clusters (proportions of all trajectories in one life phase sum to 100%). Clusters have been ranked according to their map location along a metropolitan-non-metropolitan gradient (see Fig. 8), cluster C1 and C2 being in the metropolitan core, cluster C11 and C12 being the most peripheral in relation to metropolitan areas. Right-most columns show average population density, level of diversity based on entropy, proportion of SD votes (Sverigedemokraterna, right wing populist party), and proportion of MP votes (Miljöpartiet, green party) in the 2022 parliamentary election.

**Fig. 8:**
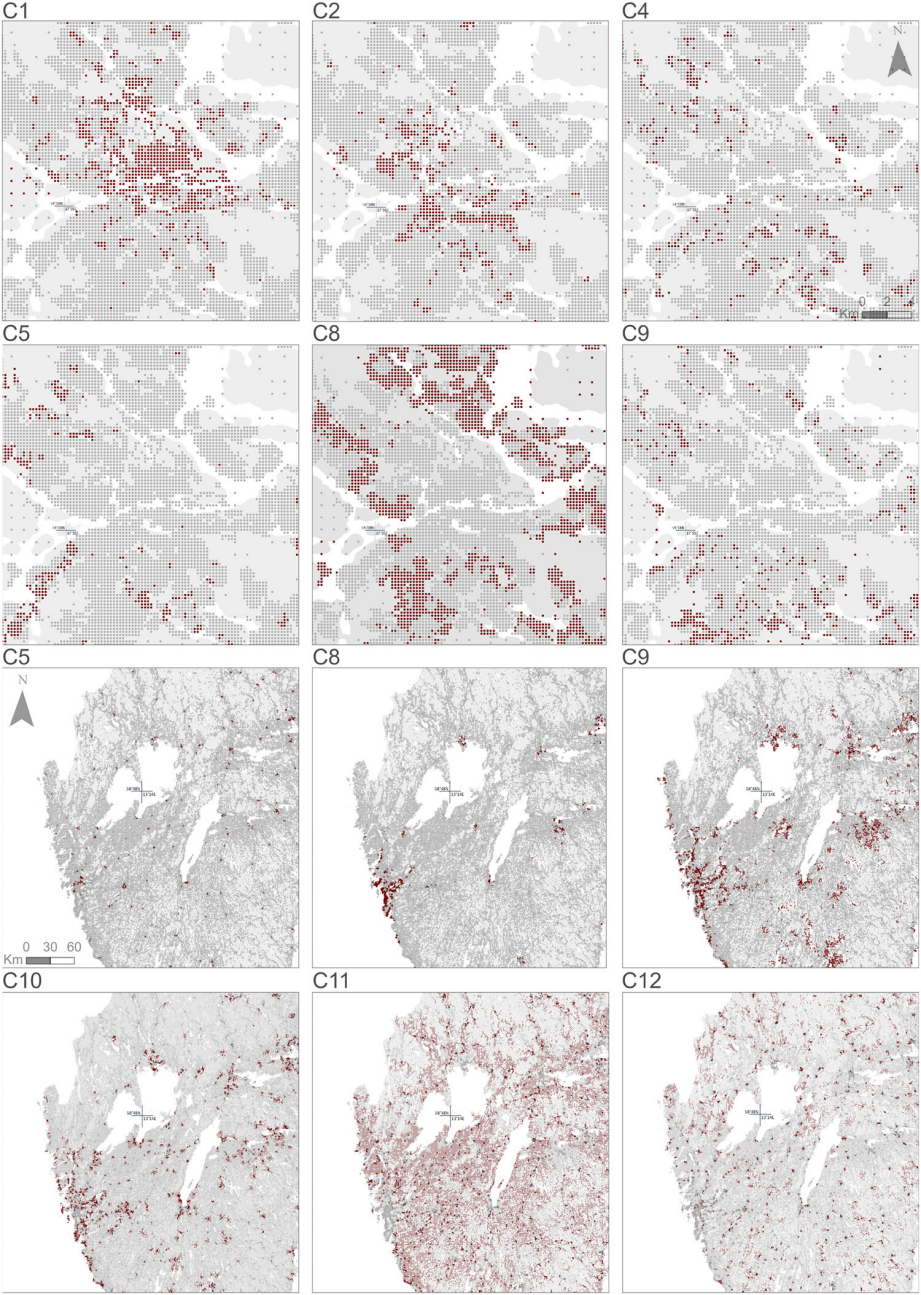
Location of clusters with different compositions of life-course trajectories among the nearest 400 neighbors, see Fig. 7. Top rows show location within Stockholm for clusters C1, C2, C4, C5, C8, and C9. Bottom rows show location in Western Sweden for clusters C5, C8, C9, C10, C11, and C12.

A key characteristic of the resulting geographical patterns is that trajectory types with low separation indices exhibit similar geographical distributions. This is demonstrated in Fig. 7 where *k* = 400 neighborhoods with similar trajectory compositions have been grouped into clusters using *k*-means clustering. Here, one finds that a low separation index in Fig. 5 corresponds to clusters in Fig. 7 with high proportions of the involved trajectories. Figure 8 presents the geographical location for a selection of these clusters and demonstrates the way in which trajectory composition and geographical location are strongly linked. Thus, in addition to relatively high levels of spatial separation between trajectory groups, there are also systematic patterns in how different trajectory groups are allocated in across Sweden: Singles and young advantages trajectories are found in the urban core (C1). Advantaged trajectories successfully compete for central urban positions (C2) and suburban locations close to the metropolitan center (C8). Middle-income, non-homeowners are found outside the urban core. Middle-income trajectories achieve homeownership but primarily in less central locations (C10 and C11). Disadvantaged trajectories are found in urban margins (C5) and remote areas (C12), reflecting their precarious position. Supplementary Fig. 32 and Fig. 33 present maps for all clusters. This suggests that the forces that are responsible for the concentration of (different) trajectories into specific neighborhoods at the same time will generate a differentiated social landscape of geographical places/social areas with very different characteristics. This gradient, from metropolitan core to remote periphery, is more continuous than a simple urban-rural dichotomy suggests, with the most pronounced segregation of middle-income homeowner trajectories concentrated in semi-peripheral areas that fall outside the metropolitan core yet dominate numerically in the Swedish population.

## Discussion

The metropolitan/non-metropolitan gradient we find in the trajectory composition of different trajectories aligns with geographical differences in the support for right-wing populist parties that is reported in the literature on political polarization. A similar gradient is present also in Swedish voting patterns, see the two right-most columns of Fig. 7. Right-wing populist party votes are highest in remote rural areas with a high proportion of disadvantaged trajectories and with few in advantaged trajectories in a way that fit with the notions of left-behind places^44^ and place-based resentment^5^, In contrast, right-wing populist party votes are lower in the metropolitan areas where disadvantaged trajectories are clustered alongside advantaged trajectories, which aligns with recent findings for both Europe and the US that right wing populism is weaker in regions with high income inequality^45^. This pattern both supports and qualifies existing theories. Concentration of disadvantaged trajectories in remote areas aligns with structural accounts of left behind places. However, populist support is lower in metropolitan areas where disadvantaged trajectories coexist alongside advantaged ones, which suggests that individual disadvantage alone is not sufficient. What appears to matter is the local mix of life-course experiences: where contrasting trajectories coexist, worldviews are more contested, and populist support is dampened.

This link between the trajectory composition and the way party alignments have developed, points to the possibility of relating populist radical right support, or lack of support, to the way world views and attitudes are molded in an interaction between individual life experiences and the life experiences that are represented in the local context. It has been proposed that an understanding of the development of populism needs an analysis that considers both objective conditions, individual resources, and group level interpretations^6,46^. We would argue that rich empirical constructs such as the life-course trajectories we have identified can be helpful for that type of analysis and for understanding spatial differentiation in general. The reason for this is that trajectories reflect local economic conditions through effects on individual economic outcomes. Trajectories also reflect individual resources, and they signal differences in life experience. The local trajectory composition, finally, captures the local context within which group level interpretations develop.

Moreover, factors that have structured Swedish life-course trajectory landscapes—including a concentration of knowledge-intensive activities and highly educated individuals to metropolitan areas, sharply increasing housing costs in densely populated regions^47^, and large inflows of migrant groups that settle in urban areas^48^—are of importance not only in Sweden. This suggests that an exploration of life-course trajectory landscapes in other countries would show patterns that can be like those found in Sweden. In this way, our study could provide a template for how geographical polarization can be seen as dependent on underlying geographical segregation of life-course trajectory types. These findings also carry implications for how residential segregation is measured and addressed. Studies confined to metropolitan areas will systematically underestimate segregation among middle-income groups, whose most homogeneous clustering occurs in semi-peripheral regions outside the urban core. At the same time, our results show that metropolitan areas are not uniformly segregated: both advantaged and disadvantaged trajectories are represented there, and younger adults, in particular, tend to inhabit mixed residential environments to a higher degree than after age 35, when many transition to homeownership. This suggests that the period before age 40 represents a window during which residential mixing is both more common and more tractable. Housing policies that expand access to metropolitan housing markets for middle-income families and enable low-income families with children to enter areas currently dominated by middle-income homeowners would target precisely the transition that our analysis identifies as the key mechanism driving persistent segregation. Subsidies or tenure-mix requirements directed at these groups could help extend into later life phases the mixing that metropolitan areas currently provide to younger adults.

## Methods

### Data

To classify life-course trajectories, we use Swedish register data (Statistics Sweden, Geographical context, P0623) from 1990 to 2019 and focus on persons aged 16–69 years. For each person in each year, we assign a life phase as follows: Transition to adulthood, 16–29, *n* = 5,586,095; Young middle age, 25–39, *n* = 6,045,850; Middle middle age, 35–49, *n* = 5,794,252; Late middle life, 45–59, *n* = 5,521,892; Transition to retirement, 55–69, *n* = 4,828,849. Overlapping life phases are used to acknowledge that a certain age can be seen both as the end of one life phase and as the beginning of another. Note that during the 1990–2019 period, an individual can be observed in up to four life phases, see Supplementary Fig. 1. The data is stored and processed using servers and software available at Statistics Sweden. The software includes STATA, SPSS, and Mplus. Access is through the MONA system (Microdata Online Access). For more information about the register data, see Supplementary Methods.

### Indicator variables

The selection of indicator variables has been based on a consideration about which variables, available in our register data, that best capture different socio-demographic positions that individuals attain during adult life. Educational progress is measured using four indicators of education length. We use four variables that can indicate socio-economic marginality: low earned income, social insurance payout (includes unemployment benefit, sick benefit, and early retirement benefit), low-income standard, and social benefit. In contrast, homeownership, top earnings and top disposable income indicate an advantaged life situation. An individual’s family status is measured using an indicator of relationship status, a child in the household indicator, and a being-a-child in the household indicator. Not all positions are relevant in all life phases, and indicator variables that have little or no variation within a life phase have been excluded. Thus, educational variables and being a child are included only for the Transition to Adulthood and Young Adulthood life phases. In a similar fashion, are two last variables, having pension income and being alive are only included for the Late Adulthood and Transition to Retirement life phases. The being-alive variable has been included as an indicator of health. Taken together, these variables capture different important determinants of an individual’s life situation and, therefore, it is of interest to explore how different individuals move within the multidimensional space that is defined by these variables. For details on these variables, see Tables 1 and 2. Moreover, these variables will have effects on housing needs and housing resources, which makes it likely that they will influence geographical outcomes.

**Table 1:** Domains, indicators and states

Indicators	Definition	States
**Educational domain**		
Lower secondary	ISCED 1997 Level = 2	1 = yes; 0 = no
Upper secondary	ISCED 1997 Level = 3	1 = yes; 0 = no
Short tertiary	ISCED 1997 Level = 4	1 = yes; 0 = no
Long tertiary	ISCED 1997 Level = 5 or 6	1 = yes; 0 = no
**Family status**		
Couple	Family type corresponds to married, in partnership, or cohabitation with children	1 = yes; 0 = no
With Child	Family type corresponds to household with children (below 18 years, or 18 years and older)	1 = yes; 0 = no
Child	Family status corresponding to being child in the household. Includes adults who live with parents	1 = yes; 0 = no
**Economic status**		
Social benefit	Reception of social allowance, household	1 = yes; 0 = no
At risk of poverty	Equalized disposable income less than 60% of the median	1 = yes; 0 = no
Top disposable income	Equalized disposable income in top equalized disposable income decile	
Homeownership	Residence in owner occupied house	1 = yes; 0 = no
**Employment status**		
Low earned income	Earned income is lower that 40% of the median earned income, can be interpreted as marginal employment	1 = yes; 0 = no
Top earned income	Earned income is in the top earned income decile	1 = yes; 0 = no
Labor market distress	Reception of sick leave compensation, unemployment benefits, or early retirement benefit	1 = yes; 0 = no
**Later life**		
Pension	Receives old-age pension benefits and early retirement benefits that exceed 49 percent of the total earnings received each year. Total earnings include old-age pension benefits, early retirement benefits, employment income, and unemployment benefits (Kridahl and Kolk,^58^; Qi, Helgertz, and Bengtsson,^59^)	1 = yes; 0 = no
Alive	Date of death is missing or larger than the current year	1 = yes; 0 = no

**Table 2:** Indicators used in latent class analysis by life phase

	Life phase				
	Transition to adult-hood, TA	Young middle age, YM	Middle middle age, MM	Late middle age, LM	Transition to retire-ment, TR
	16–29	25–39	35–49	45–59	55–69
**Educational progression**					
Lower secondary	Yes	Yes	No	No	No
Upper secondary	Yes	Yes	No	No	No
Short tertiary	Yes	Yes	No	No	No
Long tertiary	Yes	Yes	No	No	No
**Precarious life situation**					
Low earned income	Yes	Yes	Yes	Yes	Yes
Labor market distress	Yes	Yes	Yes	Yes	Yes
At risk of poverty	Yes	Yes	Yes	Yes	Yes
Social benefit	Yes	Yes	Yes	Yes	Yes
**Affluent**					
Top earned income	Yes	Yes	Yes	Yes	Yes
Top disposable income	Yes	Yes	Yes	Yes	Yes
Homeownership	Yes	Yes	Yes	Yes	Yes
**Family status**					
Couple	Yes	Yes	Yes	Yes	Yes
With Child	Yes	Yes	Yes	Yes	Yes
Child	Yes	Yes	No	No	No
**Later life**					
Pension	No	No	No	Yes	Yes
Alive	No	No	No	Yes	Yes
**Number of indicators**	**14**	**14**	**8**	**12**	**12**

### Latent class analysis

From the indicators used for a given life phase, we build multi-domain life-course trajectories, where states are followed in the life phase age span. To examine how these trajectories formed typical life-course experiences of individuals aged 16–69 between 1990 and 2019 in Sweden we apply latent class analysis. Latent class analysis is a model-based approach where the central assumption of latent class analysis is that there is an underlying data generating process, the parameters of which can be estimated from the observed data^49^. Latent class analysis can be used to obtain typical life-course trajectories in a similar way to sequence analysis, although one advantage of latent class analysis is that this method performs better for larger datasets. Latent class analysis was done separately for each life phase, where the sample consisted of all individuals who were in life phase between 1990 and 2019. Based on earlier experiences with latent class analysis of life-course trajectories, we chose to assign 5 latent classes to each life phase, striking a balance between having enough capture variation between latent classes in each life phases and a manageable number of latent classes for 5 life phases^50,51^.

We have also run LCA with 3–7 classes. See Supplementary Table 4. For MM, LM, and TR, there are only small gains in adjusted BIC as we increase from 5 classes to 6 classes and 7 classes. For TA, the gains are small going beyond 3 classes. The gain from going from 5 to 6 classes for YM is relatively large though, indicating that 6 classes could be considered. Also, the entropy values suggest that 6 classes could be a good choice for YM. A comparison of the 5-class and 6-class solution (see Supplementary Fig. 34) shows that one class in the 5-class solution, YM3, is a mixture of disadvantaged and advantaged individuals and splits into two more distinct classes in the 6-class solution, indicating that the 6-class solution is to be preferred. However, the new advantaged class is very small, with only 6% of the individuals in the life phase. We have therefore chosen to keep the 5-class solution for YM in the analysis, but it should be noted that the YM3 class should be interpreted with some care.

### *K*-nearest neighbor measurement of population composition

*K*-nearest neighbor was utilized to measure local population composition. This is based on an algorithm that for everyone, identifies the nearest neighbors and then computes the composition of the neighborhood’s population. This approach provides geographical detail without disclosing information about individuals. It also ensures that local context is measured in a uniform way. Other labels used for this approach are bespoke neighbourhoods^52^, egohoods^53^, and individualized neighbourhoods^54^, and it can be implemented in different ways (see, for example, http://equipop.kultgeog.uu.se and https://github.com/PonHen/geocontext). In this study, we have used *k* = 400 neighborhoods, where *k* is computed using the total population in each grid cell. The trajectory composition of the *k* = 400 neighborhoods is based on the location of classified individuals in 2019. Individuals that in 2019 are classified in two different life phases have been assigned to the later life phase if they have spent four calendar years in the later life phase, and if not, they are assigned to the younger life phase.

The *k*-nearest neighbor approach was implemented using kd-tree algorithms to efficiently identify nearest neighbors across the population dataset, *n* = 6,400,439. For each 250 m grid cell, concentric buffers were extended until encompassing at least 400 individuals from the 2019 population. This computational approach enables analysis of individualized neighborhoods across the entire study area while maintaining reasonable processing times for large-scale population data.

Several limitations should be noted. First, our coordinate data are recorded at 250-m grid resolution in built-up areas. While this represents high spatial granularity, individuals sharing the same grid coordinates may live some distance apart within that cell, introducing unmeasured micro-scale variation. Second, we focus on a single scale (*k* = 400) for analytical clarity, though residential contexts operate across multiple spatial scales. We selected *k* = 400 as it represents a balance between local detail (average radius 2.92 km, approximating 15-min neighborhoods) and sufficient population for measuring trajectory composition across 25 types. Smaller neighborhoods would be inadvisable given our coordinate resolution and the need to measure multiple trajectory types; larger neighborhoods would obscure the local homogeneity of interest^55^.

### Segregation measurement

The separation index was calculated using a formulation proposed by Fossett in his comprehensive and thoroughly researched book on segregation measurement methodology^56^. Substantively, separation index is a measure of segregation which looks at evenness of distribution of groups in space, similar to Dissimilarity Index, DI. However, it has been argued that separation index performs better for analyses which includes sparsely populated areas^57^. A high value of separation index indicates that two groups, A and B, differ in their average level of residential contact with group A, this difference would be 0 under even distribution and 1 under complete segregation. For calculating separation index, we use 2019, the final year for which data is available, and consider composition in small neighborhoods, which for each inhabited grid cell consists of 400 closest neighbors.

### Classification of neighborhoods

Differences in the spatial distribution of life-course trajectories, when overlayed one upon the other, will produce a geography where different locations will be characterized by a specific mixing of life-course trajectory groups. To explore this geography, we have performed a *k*-means cluster analysis that groups together locations that have a similar mix of trajectories. In determining what number of clusters, we have compared how much of the variation in neighborhoods population composition that can be accounted for by 12 clusters compared to 16 clusters. We find that 12 clusters account for 71.9% of the variance and that 16 clusters can account for 78.6% of the variance. Thus, 16 clusters could be preferred. However, a larger number of clusters will make it more difficult to analyze each individual cluster, and we have therefore decided to use 12 clusters. Population density ascribed to the different clusters is based on the population and the land area of the DESO (Demographic Statistical Area) to which each classified grid cell belongs.

### Statistics and reproducibility

This study is a secondary analysis of Swedish administrative population register data. The central statistical method is latent class analysis (LCA), applied separately for each of five, 15-year long life phases (14 years long for the first life phase), to classify individuals into 25 life-course trajectory types on the basis of 9–14 life-stage specific, binary indicator variables, measured every year, capturing educational attainment, economic precarity, affluence, family status, homeownership, and mortality. LCA was conducted using Mplus; model selection was guided by adjusted Bayesian Information Criterion (BIC) and entropy values, with solutions for 3–7 classes compared. Residential segregation was quantified using the separation index calculated on *k* = 400 nearest-neighbor individualized neighborhoods constructed via kd-tree algorithms. Neighborhood cluster patterns were identified by *k*-means cluster analysis. All data management and supporting analyses were performed in STATA on secure servers at Statistics Sweden via the MONA (Microdata Online Access) system.

No statistical method was used to predetermine sample size. The study uses census-type register data covering all Swedish residents aged 16–69 observed between 1990 and 2019; the full target population is included in each life-phase analysis (n ranging from 4,828,849 to 6,045,850), which is sufficient for reliable estimation of 25 latent trajectory classes. No data were excluded from the analyses, except that records for individuals outside the 16–69 age range were not included, as this age window was pre-specified to capture adult working-age and pre-retirement life phases. The study involves no experimental manipulation, so experiments were not randomized. The investigators were not blinded to allocation during experiments and outcome assessment; blinding is not applicable to this observational register-data study, in which trajectory classification is performed algorithmically on the full population without researcher intervention.

### Reporting summary

Further information on research design is available in the Nature Portfolio Reporting Summary linked to this article.

## Supplementary information

Supplementary information

Reporting summary

Transparent Peer Review file

## Data Availability

The data that support the findings of this study are available from Statistics Sweden, but restrictions apply to the availability of these data, which were used under license for the current study, and so are not publicly available. Data are, however, available through the authors upon request and with permission of Statistics Sweden. Source data for figures and high resolution maps are provided at 10.17045/sthlmuni.32218419.

## Code availability

The code used for this paper is available at https://github.com/bmalmberg/Life-course-trajectory-landscapes, 10.5281/zenodo.19880423.
