# Supplementary material for: Life course trajectory segregation and political polarization in contemporary Sweden
Source: Nat Commun. 2026 Jul 30;17:7116. doi: 10.1038/s41467-026-75072-y (PMC13424875; doi:10.1038/s41467-026-75072-y)
Supplement: Supplementary file 1 — Supplementary information [file 41467_2026_75072_MOESM1_ESM.pdf]

Supplementary information

Life course trajectory segregation and political polarization in contemporary Sweden

Authors

Bo Malmberg[1]*, Juta Kawalerowicz[1], Eva K. Andersson[1]

[1]Stockholm University, Department of Human Geography, 106 91 Stockholm, Sweden

* e-mail: bo.malmberg@humangeo.su.se.

Full resolution maps are available at https://doi.org/10.17045/sthlmuni.32218419

Table of contents

Swedish Register Data: The LISA database 3
Supplementary Table S 1 Number of observations with non-missing variables by life phase and decile 5
Supplementary Table S 2 Proportion of non-missing values by life phase and decile 5
Supplementary Table S 3 Probability of assignment by life phase and decile of non-missing value 6
Supplementary Table S 4 Diagnostics for the latent class analysis. Adjusted BIC and Entropy values obtained for 3 to 7 classes. 6
Supplementary Figure S 1 Lexis diagram of cohorts used to generate input data. 7
Supplementary Figure S 2 Trajectory profiles: Transition to adulthood 16-29 years 8
Supplementary Figure S 3 Trajectory profiles: Young middle age 25-39 years 8
Supplementary Figure S 4 Trajectory profiles: Middle middle age 35-49 years 9
Supplementary Figure S 5 Trajectory profiles: Late middle age 45-59 years 9
Supplementary Figure S 6 Trajectory profiles: Transition to retirement 55-69 years 10
Supplementary Figure S 7 Spatial distribution of disadvantaged life course TA1 11
Supplementary Figure S 8 Spatial distribution of disadvantaged life course TA5 13
Supplementary Figure S 9 Spatial distribution of disadvantaged life course YM1 15
Supplementary Figure S 10 Spatial distribution of disadvantaged life course YM3 17
Supplementary Figure S 11 Spatial distribution of disadvantaged life course MM5 19
Supplementary Figure S 12 Spatial distribution of disadvantaged life course LM3 21
Supplementary Figure S 13 Spatial distribution of disadvantaged life course TR3 23
Supplementary Figure S 14 Spatial distribution of middle income singles life course YM5 25
Supplementary Figure S 15 Spatial distribution of middle income singles life course MM1 27
Supplementary Figure S 16 Spatial distribution of middle income singles life course LM2 29
Supplementary Figure S 17 Spatial distribution of middle income singles life course TR2 31
Supplementary Figure S 18 Spatial distribution of middle income non homeowner life course TA2 33
Supplementary Figure S 19 Spatial distribution of middle income non homeowner life course MM3 35
Supplementary Figure S 20 Spatial distribution of middle income non homeowner life course LM1 37
Supplementary Figure S 21 Spatial distribution of middle income non homeowner life course TR1 39
Supplementary Figure S 22 Spatial distribution of middle income homeowner life course TA4 41
Supplementary Figure S 23 Spatial distribution of middle income homeowner life course YM4 43
Supplementary Figure S 24 Spatial distribution of middle income homeowner life course MM2 45
Supplementary Figure S 25 Spatial distribution of middle income homeowner life course LM4 47
Supplementary Figure S 26 Spatial distribution of middle income homeowner life course TR4 49
Supplementary Figure S 27 Spatial distribution of advantaged life course TA3 51
Supplementary Figure S 28 Spatial distribution of advantaged life course YM2 53
Supplementary Figure S 29 Spatial distribution of advantaged life course MM4 55
Supplementary Figure S 30 Spatial distribution of advantaged life course LM5 57
Supplementary Figure S 31 Spatial distribution of advantaged life course TR5 59
Supplementary Figure S 32: Locations of life- course trajectories clusters in Western Sweden 61
Supplementary Figure S 33: Locations of life- course trajectories clusters in Stockholm 62
Supplementary Figure S 34: Proportions of life course states for 5-class and 6-class solution 63

Supplementary Methods

Swedish Register Data: The LISA database

Our main data source is the Swedish longitudinal integrated database for health insurance and labour market studies (LISA). A description of LISA is given below.

Types of data collected:

The LISA database integrates multiple data domains through linkage of national registers. The socioeconomic and demographic variables include:

- **Education**: Highest attained educational level coded according to the SUN2000 nomenclature, with data completeness >98% for individuals aged 25-64 years. Education data are sourced from over 30 institutions including schools, universities, and immigration authority questionnaires.
- **Income and financial variables**: Individual gross earnings from employment, business income, capital income, and various social benefits (parental allowance, sick leave benefits, disability pension, unemployment benefits, social welfare). All income variables are recorded as multiples of 100 SEK. Disposable income is calculated as total income minus taxes, available at individual, family, and consumption-weighted household levels.
- **Employment and occupation**: Employment status (estimated from salary reports and labor force surveys), occupation classified according to SSYK (Swedish Standard Classification of Occupations) with 95% completeness, and socioeconomic classification (SEI, YSEG, ESeG systems depending on year). *Not used in this study.*
- **Family composition**: Civil status, household structure, number and ages of children, and family identification numbers enabling linkage of family members.
- **Geographic information**: County and municipality of residence as of December 31 annually. *In addition we have residential coordinates for 250 meter squares in built up areas an for 1000 meter squares outside built up areas.*

Population included:

LISA encompasses all individuals aged ≥15 years (≥16 years during 1990-2009) who are registered as residents in Sweden on December 31 each year, constituting approximately 10 million individuals in 2017. The database has nationwide geographic coverage across all Swedish counties and municipalities.

Notable characteristics include:

- Compulsory participation in Swedish government-administered registers minimizes selection bias
- Small over-coverage of approximately 0.5% due to delayed reporting of deaths and emigration
- Immigration of approximately 97,000 individuals annually (2000-2017), representing about 1% of the population
- Data on parents available for >99% of individuals born since 1950

Data collection procedures:

LISA data are compiled annually by Statistics Sweden from multiple administrative sources:

- **Primary data sources**: Swedish Social Insurance Agency (sick leave, disability pension), Swedish Public Employment Service (unemployment), Education Register, Register of Income and Taxation, Occupation Register, and linkage to the Total Population Register for demographic data.
- **Linkage methodology**: The Swedish personal identity number (PIN), assigned to all Swedish residents, serves as the unique identifier enabling deterministic linkage across registers with minimal linkage error.
- **Reporting mechanisms**: Employers are legally required to report employee identities and salaries to tax authorities. These "kontrolluppgift" reports form the basis for employment and income variables. Self-employed individuals are similarly required to report income.
- **Data quality assurance**: Education data accuracy for highest attained level is estimated at 85%. Occupation data completeness is approximately 95%, with missing data primarily from unreported firms and certain employment categories.
- **Temporal considerations**: LISA is updated annually with approximately 15-month delay. Most statistics originate from December 31 each year, though some variables (e.g., RAMS employment data) reflect November status, and retirement pension data may reflect either December 31 or last payment date.
- **Ethical considerations**: As a government-administered register with mandatory participation, individual consent is not required. However, researcher access requires ethical approval from the Swedish Ethical Review Authority and formal application to Statistics Sweden, with data delivered in de-identified format for approved research projects.

Source: Ludvigsson, J. F., Svedberg, P., Olén, O., Bruze, G., & Neovius, M. (2019). The longitudinal integrated database for health insurance and labour market studies (LISA) and its use in medical research: JF Ludvigsson et al. *European journal of epidemiology*, *34*(4), 423-437.

Supplementary Tables

Supplementary Table S1 Number of observations with non-missing variables by life phase and proportion of non-missing variables

	Number of observations				
Proportion non-missing	TA	YM	MM	LM	TR
0 — 0.1	371526	427806	308457	449660	357394
0.1 — 0.2	431856	732993	620306	477113	385241
0.2 — 0.3	628758	373352	309631	278039	352917
0.3 — 0.4	322398	649211	595943	467972	368097
0.4 — 0.5	556127	313666	279361	253432	315690
0.5 — 0.6	269293	554675	540800	264169	353240
0.6 — 0.7	273755	272100	262637	496899	321689
0.7 — 0.8	516937	505163	517925	249641	315713
0.8 — 0.9	280744	258544	258770	503791	303773
0.9 — 1	1934701	1958340	2100422	2081176	1755095
All observation	5586095	6045850	5794252	5521892	4828849

Supplementary Table S2 Proportion of observations with non-missing variables by life phase and proportion of non-missing variables

	Proportion of observations				
Proportion non-missing	TA	YM	MM	LM	TR
0 — 0.1	6.7%	7.1%	5.3%	8.1%	7.4%
0.1 — 0.2	7.7%	12.1%	10.7%	8.6%	8.0%
0.2 — 0.3	11.3%	6.2%	5.3%	5.0%	7.3%
0.3 — 0.4	5.8%	10.7%	10.3%	8.5%	7.6%
0.4 — 0.5	10.0%	5.2%	4.8%	4.6%	6.5%
0.5 — 0.6	4.8%	9.2%	9.3%	4.8%	7.3%
0.6 — 0.7	4.9%	4.5%	4.5%	9.0%	6.7%
0.7 — 0.8	9.3%	8.4%	8.9%	4.5%	6.5%
0.8 — 0.9	5.0%	4.3%	4.5%	9.1%	6.3%
0.9 — 1	34.6%	32.4%	36.3%	37.7%	36.3%
All observation	100.0%	100.0%	100.0%	100.0%	100.0%

Supplementary Table S3 Probability of assignment to latent class by life phase and proportion of non-missing variables

	Probability of assignment				
Proportion non-missing	TA	YM	MM	LM	TR
0 — 0.1	66.4%	76.1%	78.1%	56.8%	64.6%
0.1 — 0.2	79.5%	93.1%	92.3%	94.6%	94.1%
0.2 — 0.3	86.4%	96.1%	95.7%	97.2%	95.8%
0.3 — 0.4	93.2%	97.8%	96.8%	98.1%	97.7%
0.4 — 0.5	96.1%	98.3%	97.5%	98.4%	97.2%
0.5 — 0.6	97.7%	98.8%	98.0%	98.6%	98.4%
0.6 — 0.7	97.9%	98.9%	98.3%	98.8%	98.5%
0.7 — 0.8	98.2%	99.1%	98.4%	98.9%	98.8%
0.8 — 0.9	98.4%	99.2%	98.5%	99.0%	98.7%
0.9 — 1	98.6%	99.3%	98.6%	99.1%	99.0%

Comment: Assignment probabilities increase rapidly with increasing proportions of non-missing values. Observations with a proportion of non-missing values between 0.1 and 0.2 have 17 to 42 non-missing variables.

Supplementary Table S4 Diagnostics for the latent class analysis. Adjusted BIC and Entropy values obtained for 3 to 7 classes.

**Adjusted BIC**	Life phase				
Number of latent classes	TA	YM	MM	LM	TR
3	4 667 687	5 602 987	3 811 063	4 065 270	3 305 841
4	4 532 013	5 360 532	3 609 218	3 833 404	3 114 194
5	4 414 910	5 159 113	3 451 327	3 663 868	2 982 839
6	4 308 289	5 005 230	3 351 874	3 562 881	2 893 648
7	4 232 716	4 855 418	3 267 757	3 466 373	2 815 211
**Entropy**	Life phase				
Number of latent classes	TA	YM	MM	LM	TR
3	0.905	0.962	0.937	0.930	0.921
4	0.896	0.949	0.940	0.924	0.933
5	0.895	0.945	0.941	0.924	0.929
6	0.887	0.956	0.941	0.925	0.920
7	0.884	0.944	0.941	0.923	0.923

Supplementary Figures

Life phase						Years of observation																													
TA	YM	MM	OM	TR	Year of birth	1990	1991	1992	1993	1994	1995	1996	1997	1998	1999	2000	2001	2002	2003	2004	2005	2006	2007	2008	2009	2010	2011	2012	2013	2014	2015	2016	2017	2018	2019
				1925	1925	65	66	67	68	69	70	71	72	73	74	75	76	77	78	79	80	81	82	83	84	85	86	87	88	89	90	91	92	93	94
					1926	64	65	66	67	68	69	70	71	72	73	74	75	76	77	78	79	80	81	82	83	84	85	86	87	88	89	90	91	92	93
					1927	63	64	65	66	67	68	69	70	71	72	73	74	75	76	77	78	79	80	81	82	83	84	85	86	87	88	89	90	91	92
					1928	62	63	64	65	66	67	68	69	70	71	72	73	74	75	76	77	78	79	80	81	82	83	84	85	86	87	88	89	90	91
					1929	61	62	63	64	65	66	67	68	69	70	71	72	73	74	75	76	77	78	79	80	81	82	83	84	85	86	87	88	89	90
					1930	60	61	62	63	64	65	66	67	68	69	70	71	72	73	74	75	76	77	78	79	80	81	82	83	84	85	86	87	88	89
				69	1931	59	60	61	62	63	64	65	66	67	68	69	70	71	72	73	74	75	76	77	78	79	80	81	82	83	84	85	86	87	88
				68	1932	58	59	60	61	62	63	64	65	66	67	68	69	70	71	72	73	74	75	76	77	78	79	80	81	82	83	84	85	86	87
				67	1933	57	58	59	60	61	62	63	64	65	66	67	68	69	70	71	72	73	74	75	76	77	78	79	80	81	82	83	84	85	86
				66	1934	56	57	58	59	60	61	62	63	64	65	66	67	68	69	70	71	72	73	74	75	76	77	78	79	80	81	82	83	84	85
			1935	65	1935	55	56	57	58	59	60	61	62	63	64	65	66	67	68	69	70	71	72	73	74	75	76	77	78	79	80	81	82	83	84
				64	1936	54	55	56	57	58	59	60	61	62	63	64	65	66	67	68	69	70	71	72	73	74	75	76	77	78	79	80	81	82	83
				63	1937	53	54	55	56	57	58	59	60	61	62	63	64	65	66	67	68	69	70	71	72	73	74	75	76	77	78	79	80	81	82
				62	1938	52	53	54	55	56	57	58	59	60	61	62	63	64	65	66	67	68	69	70	71	72	73	74	75	76	77	78	79	80	81
				61	1939	51	52	53	54	55	56	57	58	59	60	61	62	63	64	65	66	67	68	69	70	71	72	73	74	75	76	77	78	79	80
				60	1940	50	51	52	53	54	55	56	57	58	59	60	61	62	63	64	65	66	67	68	69	70	71	72	73	74	75	76	77	78	79
			59		1941	49	50	51	52	53	54	55	56	57	58	59	60	61	62	63	64	65	66	67	68	69	70	71	72	73	74	75	76	77	78
			58		1942	48	49	50	51	52	53	54	55	56	57	58	59	60	61	62	63	64	65	66	67	68	69	70	71	72	73	74	75	76	77
			57		1943	47	48	49	50	51	52	53	54	55	56	57	58	59	60	61	62	63	64	65	66	67	68	69	70	71	72	73	74	75	76
			56		1944	46	47	48	49	50	51	52	53	54	55	56	57	58	59	60	61	62	63	64	65	66	67	68	69	70	71	72	73	74	75
		1945	55		1945	45	46	47	48	49	50	51	52	53	54	55	56	57	58	59	60	61	62	63	64	65	66	67	68	69	70	71	72	73	74
			54		1946	44	45	46	47	48	49	50	51	52	53	54	55	56	57	58	59	60	61	62	63	64	65	66	67	68	69	70	71	72	73
			53		1947	43	44	45	46	47	48	49	50	51	52	53	54	55	56	57	58	59	60	61	62	63	64	65	66	67	68	69	70	71	72
			52		1948	42	43	44	45	46	47	48	49	50	51	52	53	54	55	56	57	58	59	60	61	62	63	64	65	66	67	68	69	70	71
			51		1949	41	42	43	44	45	46	47	48	49	50	51	52	53	54	55	56	57	58	59	60	61	62	63	64	65	66	67	68	69	70
			50		1950	40	41	42	43	44	45	46	47	48	49	50	51	52	53	54	55	56	57	58	59	60	61	62	63	64	65	66	67	68	69
		49			1951	39	40	41	42	43	44	45	46	47	48	49	50	51	52	53	54	55	56	57	58	59	60	61	62	63	64	65	66	67	68
		48			1952	38	39	40	41	42	43	44	45	46	47	48	49	50	51	52	53	54	55	56	57	58	59	60	61	62	63	64	65	66	67
		47			1953	37	38	39	40	41	42	43	44	45	46	47	48	49	50	51	52	53	54	55	56	57	58	59	60	61	62	63	64	65	66
		46			1954	36	37	38	39	40	41	42	43	44	45	46	47	48	49	50	51	52	53	54	55	56	57	58	59	60	61	62	63	64	65
	1955	45			1955	35	36	37	38	39	40	41	42	43	44	45	46	47	48	49	50	51	52	53	54	55	56	57	58	59	60	61	62	63	64
		44			1956	34	35	36	37	38	39	40	41	42	43	44	45	46	47	48	49	50	51	52	53	54	55	56	57	58	59	60	61	62	63
		43			1957	33	34	35	36	37	38	39	40	41	42	43	44	45	46	47	48	49	50	51	52	53	54	55	56	57	58	59	60	61	62
		42			1958	32	33	34	35	36	37	38	39	40	41	42	43	44	45	46	47	48	49	50	51	52	53	54	55	56	57	58	59	60	61
		41			1959	31	32	33	34	35	36	37	38	39	40	41	42	43	44	45	46	47	48	49	50	51	52	53	54	55	56	57	58	59	60
		40		1960	1960	30	31	32	33	34	35	36	37	38	39	40	41	42	43	44	45	46	47	48	49	50	51	52	53	54	55	56	57	58	59
		39			1961	29	30	31	32	33	34	35	36	37	38	39	40	41	42	43	44	45	46	47	48	49	50	51	52	53	54	55	56	57	58
		38			1962	28	29	30	31	32	33	34	35	36	37	38	39	40	41	42	43	44	45	46	47	48	49	50	51	52	53	54	55	56	57
	37				1963	27	28	29	30	31	32	33	34	35	36	37	38	39	40	41	42	43	44	45	46	47	48	49	50	51	52	53	54	55	56
	36				1964	26	27	28	29	30	31	32	33	34	35	36	37	38	39	40	41	42	43	44	45	46	47	48	49	50	51	52	53	54	55
1965	35				1965	25	26	27	28	29	30	31	32	33	34	35	36	37	38	39	40	41	42	43	44	45	46	47	48	49	50	51	52	53	54
	34				1966	24	25	26	27	28	29	30	31	32	33	34	35	36	37	38	39	40	41	42	43	44	45	46	47	48	49	50	51	52	53
	33				1967	23	24	25	26	27	28	29	30	31	32	33	34	35	36	37	38	39	40	41	42	43	44	45	46	47	48	49	50	51	52
	32				1968	22	23	24	25	26	27	28	29	30	31	32	33	34	35	36	37	38	39	40	41	42	43	44	45	46	47	48	49	50	51
	31				1969	21	22	23	24	25	26	27	28	29	30	31	32	33	34	35	36	37	38	39	40	41	42	43	44	45	46	47	48	49	50
	30		1970		1970	20	21	22	23	24	25	26	27	28	29	30	31	32	33	34	35	36	37	38	39	40	41	42	43	44	45	46	47	48	49
	29				1971	19	20	21	22	23	24	25	26	27	28	29	30	31	32	33	34	35	36	37	38	39	40	41	42	43	44	45	46	47	48
	28				1972	18	19	20	21	22	23	24	25	26	27	28	29	30	31	32	33	34	35	36	37	38	39	40	41	42	43	44	45	46	47
27					1973	17	18	19	20	21	22	23	24	25	26	27	28	29	30	31	32	33	34	35	36	37	38	39	40	41	42	43	44	45	46
26					1974	16	17	18	19	20	21	22	23	24	25	26	27	28	29	30	31	32	33	34	35	36	37	38	39	40	41	42	43	44	45
25					1975	15	16	17	18	19	20	21	22	23	24	25	26	27	28	29	30	31	32	33	34	35	36	37	38	39	40	41	42	43	44
24					1976	14	15	16	17	18	19	20	21	22	23	24	25	26	27	28	29	30	31	32	33	34	35	36	37	38	39	40	41	42	43
23					1977	13	14	15	16	17	18	19	20	21	22	23	24	25	26	27	28	29	30	31	32	33	34	35	36	37	38	39	40	41	42
22					1978	12	13	14	15	16	17	18	19	20	21	22	23	24	25	26	27	28	29	30	31	32	33	34	35	36	37	38	39	40	41
21					1979	11	12	13	14	15	16	17	18	19	20	21	22	23	24	25	26	27	28	29	30	31	32	33	34	35	36	37	38	39	40
20		1980			1980	10	11	12	13	14	15	16	17	18	19	20	21	22	23	24	25	26	27	28	29	30	31	32	33	34	35	36	37	38	39
19					1981	9	10	11	12	13	14	15	16	17	18	19	20	21	22	23	24	25	26	27	28	29	30	31	32	33	34	35	36	37	38
18					1982	8	9	10	11	12	13	14	15	16	17	18	19	20	21	22	23	24	25	26	27	28	29	30	31	32	33	34	35	36	37
17					1983	7	8	9	10	11	12	13	14	15	16	17	18	19	20	21	22	23	24	25	26	27	28	29	30	31	32	33	34	35	36
16					1984	6	7	8	9	10	11	12	13	14	15	16	17	18	19	20	21	22	23	24	25	26	27	28	29	30	31	32	33	34	35
					1985	5	6	7	8	9	10	11	12	13	14	15	16	17	18	19	20	21	22	23	24	25	26	27	28	29	30	31	32	33	34
					1986	4	5	6	7	8	9	10	11	12	13	14	15	16	17	18	19	20	21	22	23	24	25	26	27	28	29	30	31	32	33
					1987	3	4	5	6	7	8	9	10	11	12	13	14	15	16	17	18	19	20	21	22	23	24	25	26	27	28	29	30	31	32
					1988	2	3	4	5	6	7	8	9	10	11	12	13	14	15	16	17	18	19	20	21	22	23	24	25	26	27	28	29	30	31
					1989	1	2	3	4	5	6	7	8	9	10	11	12	13	14	15	16	17	18	19	20	21	22	23	24	25	26	27	28	29	30
	1990				1990	0	1	2	3	4	5	6	7	8	9	10	11	12	13	14	15	16	17	18	19	20	21	22	23	24	25	26	27	28	29
					1991		0	1	2	3	4	5	6	7	8	9	10	11	12	13	14	15	16	17	18	19	20	21	22	23	24	25	26	27	28
					1992			0	1	2	3	4	5	6	7	8	9	10	11	12	13	14	15	16	17	18	19	20	21	22	23	24	25	26	27
					1993				0	1	2	3	4	5	6	7	8	9	10	11	12	13	14	15	16	17	18	19	20	21	22	23	24	25	26
					1994					0	1	2	3	4	5	6	7	8	9	10	11	12	13	14	15	16	17	18	19	20	21	22	23	24	25
					1995						0	1	2	3	4	5	6	7	8	9	10	11	12	13	14	15	16	17	18	19	20	21	22	23	24
					1996						-1	0	1	2	3	4	5	6	7	8	9	10	11	12	13	14	15	16	17	18	19	20	21	22	23
					1997								0	1	2	3	4	5	6	7	8	9	10	11	12	13	14	15	16	17	18	19	20	21	22
					1998									0	1	2	3	4	5	6	7	8	9	10	11	12	13	14	15	16	17	18	19	20	21
					1999										0	1	2	3	4	5	6	7	8	9	10	11	12	13	14	15	16	17	18	19	20
2000					2000											0	1	2	3	4	5	6	7	8	9	10	11	12	13	14	15	16	17	18	19

Supplementary Figure S 1: Lexis diagram of cohorts used to generate input data for latent class analysis of overlapping life phases. Right-most columns show cohorts included in the sample for each life phase. These columns also show the ages at which individuals that are in two life phases are assigned to the older life phase when their geographical location is analyzed.

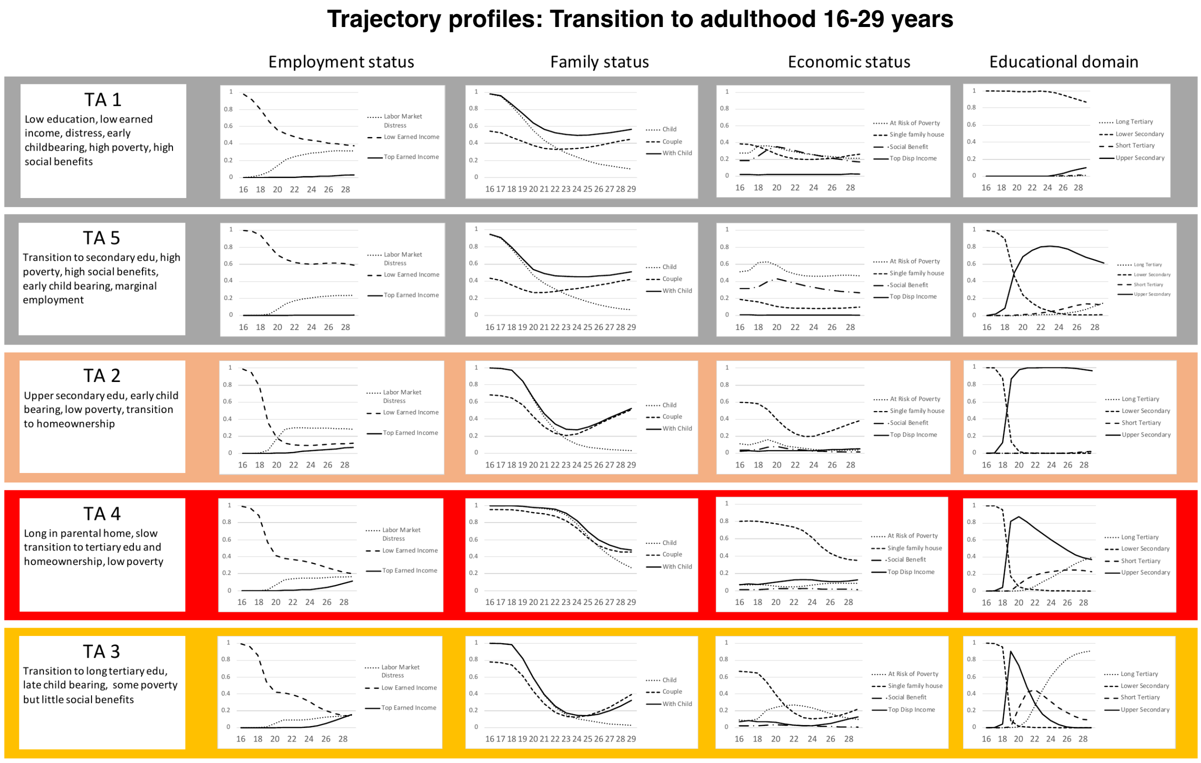

Vertical axis shows proportion of individuals that are in life course state.
Colors show stratification group: grey = disadvantaged trajectories, blue = middle income single, apricot = middle income not homeowner, red = middle income homeowner, gold = advantaged

Supplementary Figure S 2 Trajectory profiles: Transition to adulthood 16-29 years

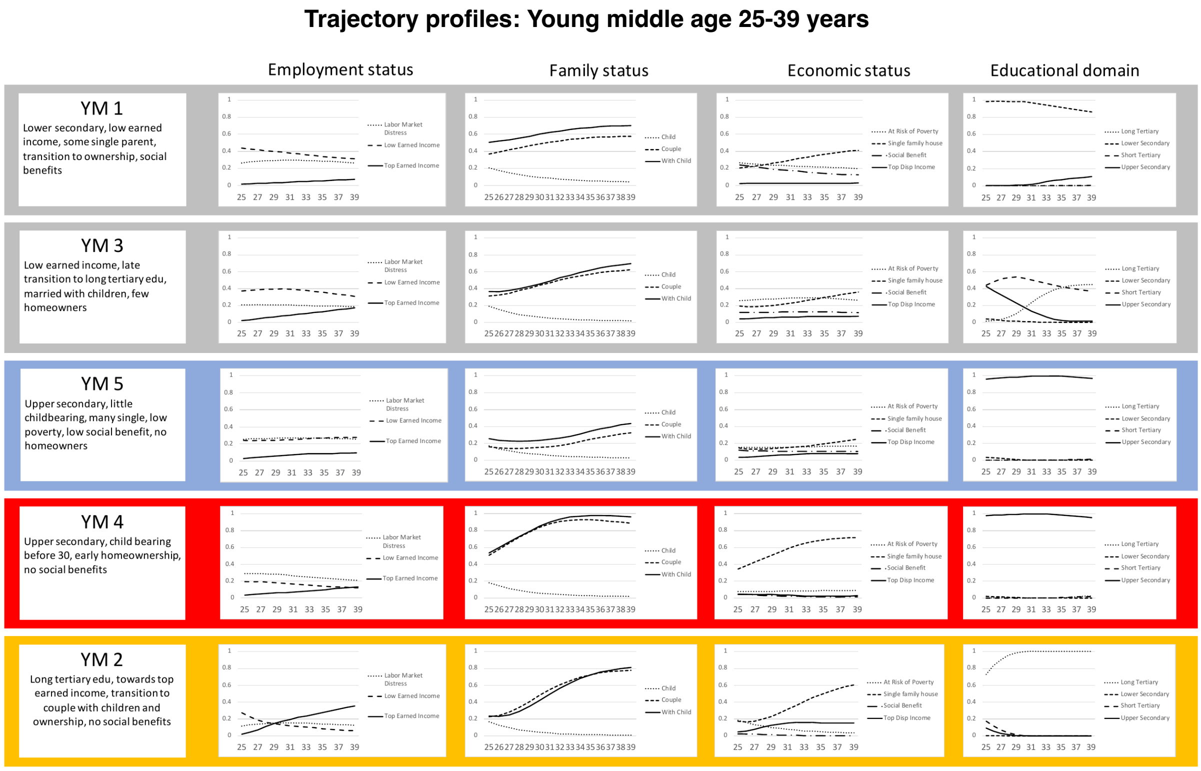

Vertical axis shows proportion of individuals that are in life course state.
Colors show stratification group: grey = disadvantaged trajectories, blue = middle income single, apricot = middle income not homeowner, red = middle income homeowner, gold = advantaged

Supplementary Figure S 3 Trajectories: Young middle age 25-39 years

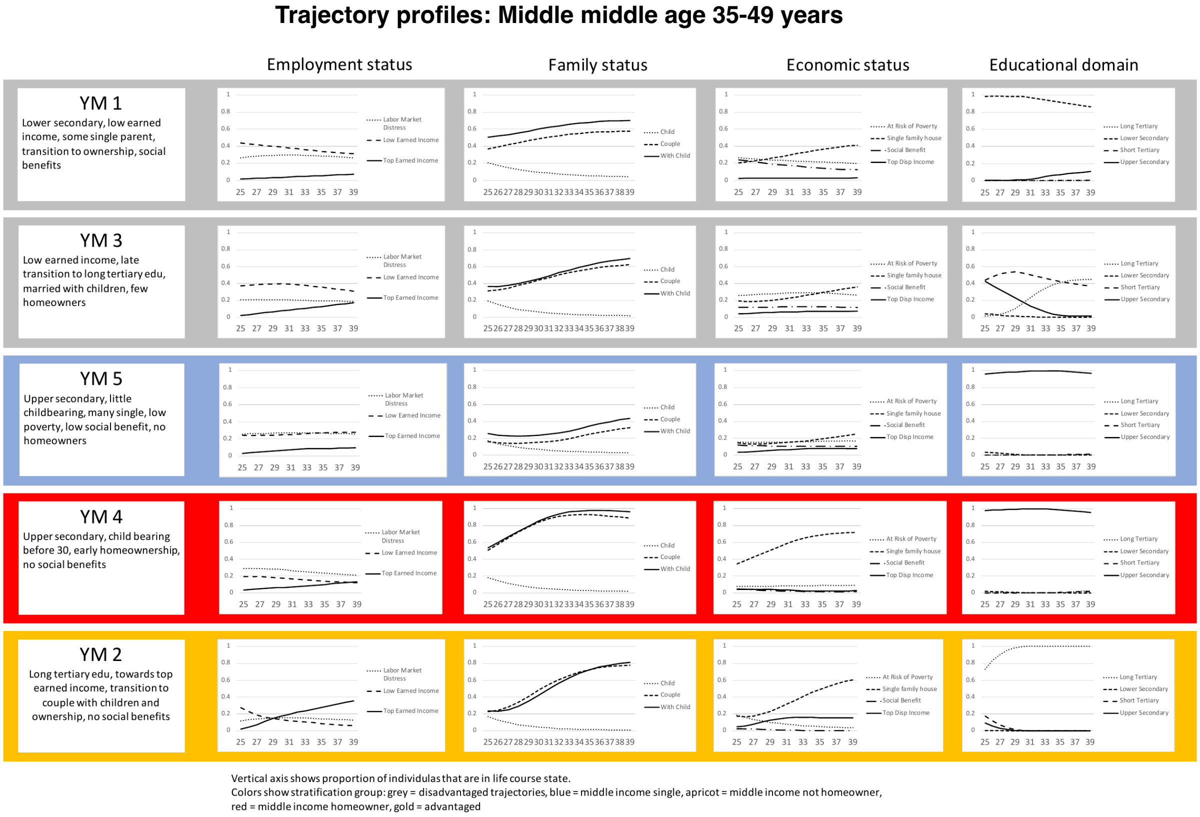

Supplementary Figure S 4 Trajectory profiles: Middle middle age 35-49 years

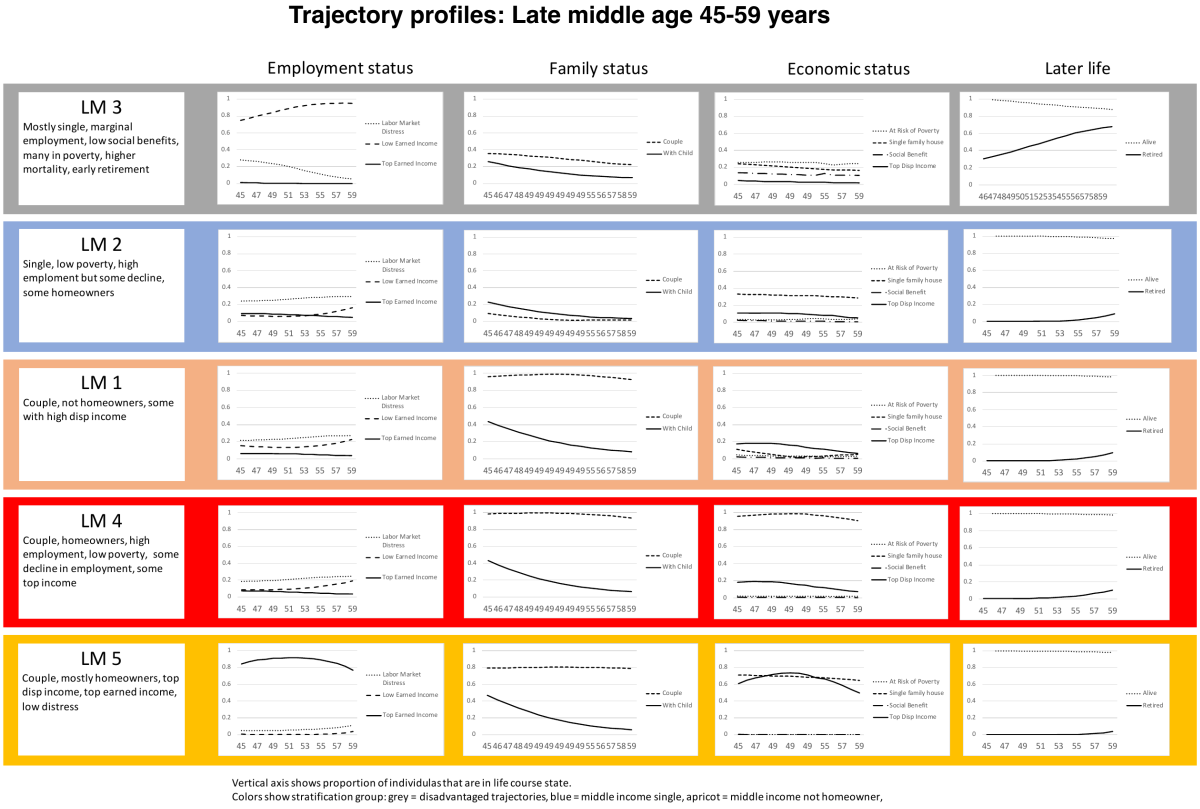

Supplementary Figure S 5 Trajectories: Late middle age 45-59 years

Trajectory profiles: Transition to retirement 55-69 years

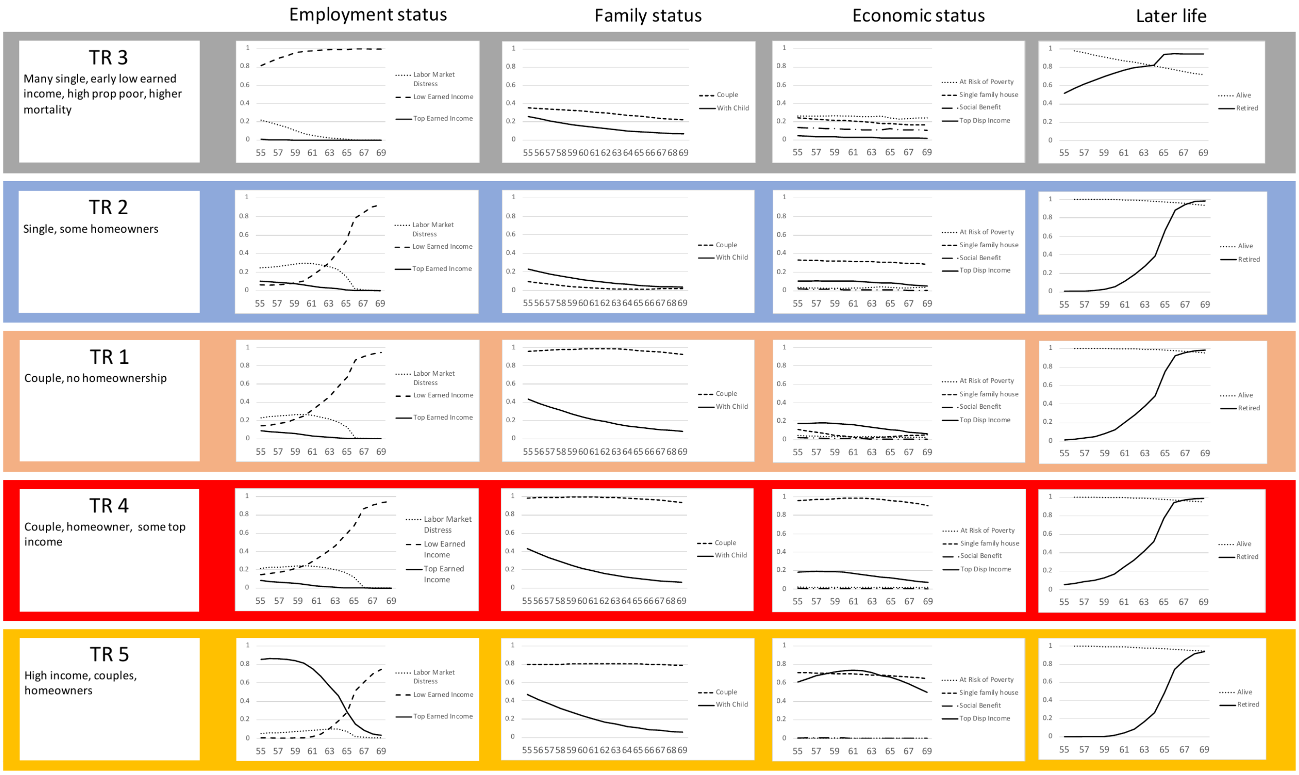

Vertical axis shows proportion of individulas that are in life course state.
Colors show stratification group: grey = disadvantaged trajectories, blue = middle income single, apricot = middle income not homeowner,

Supplementary Figure S 6 Trajectory profiles: Transition to retirement 55-69 years

Disadvantaged

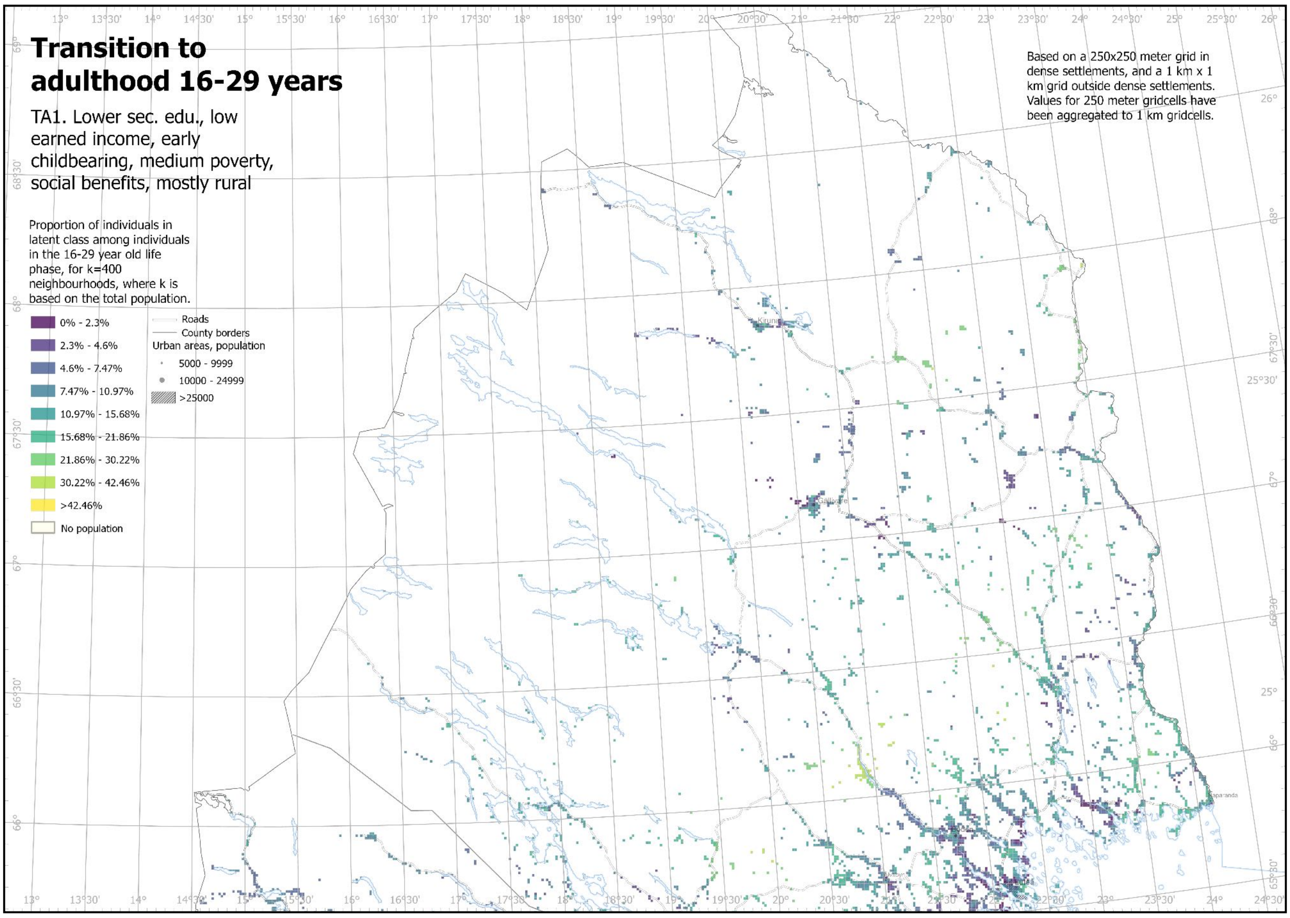

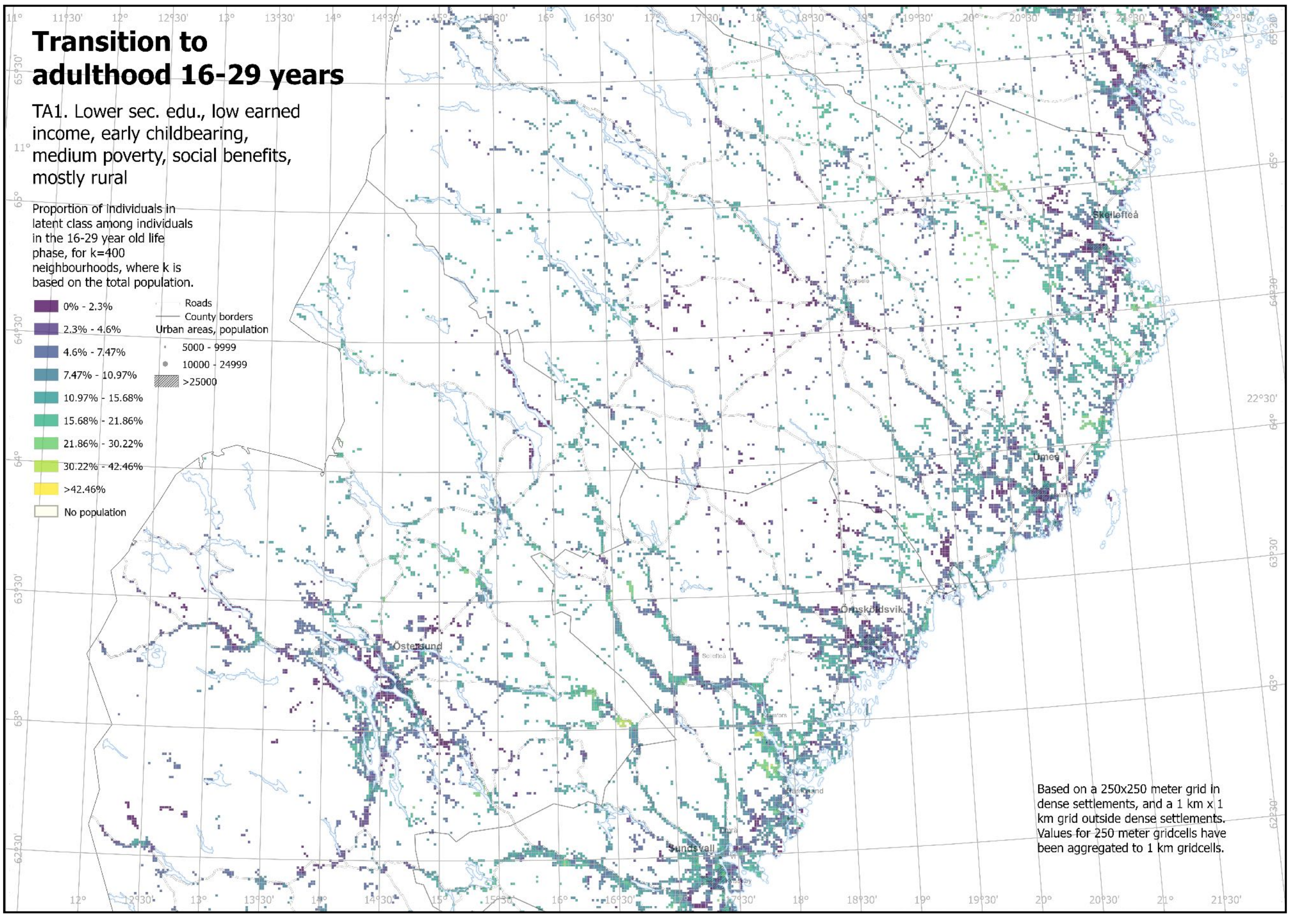

Supplementary Figure S 7 Spatial distribution of disadvantaged life course TA1

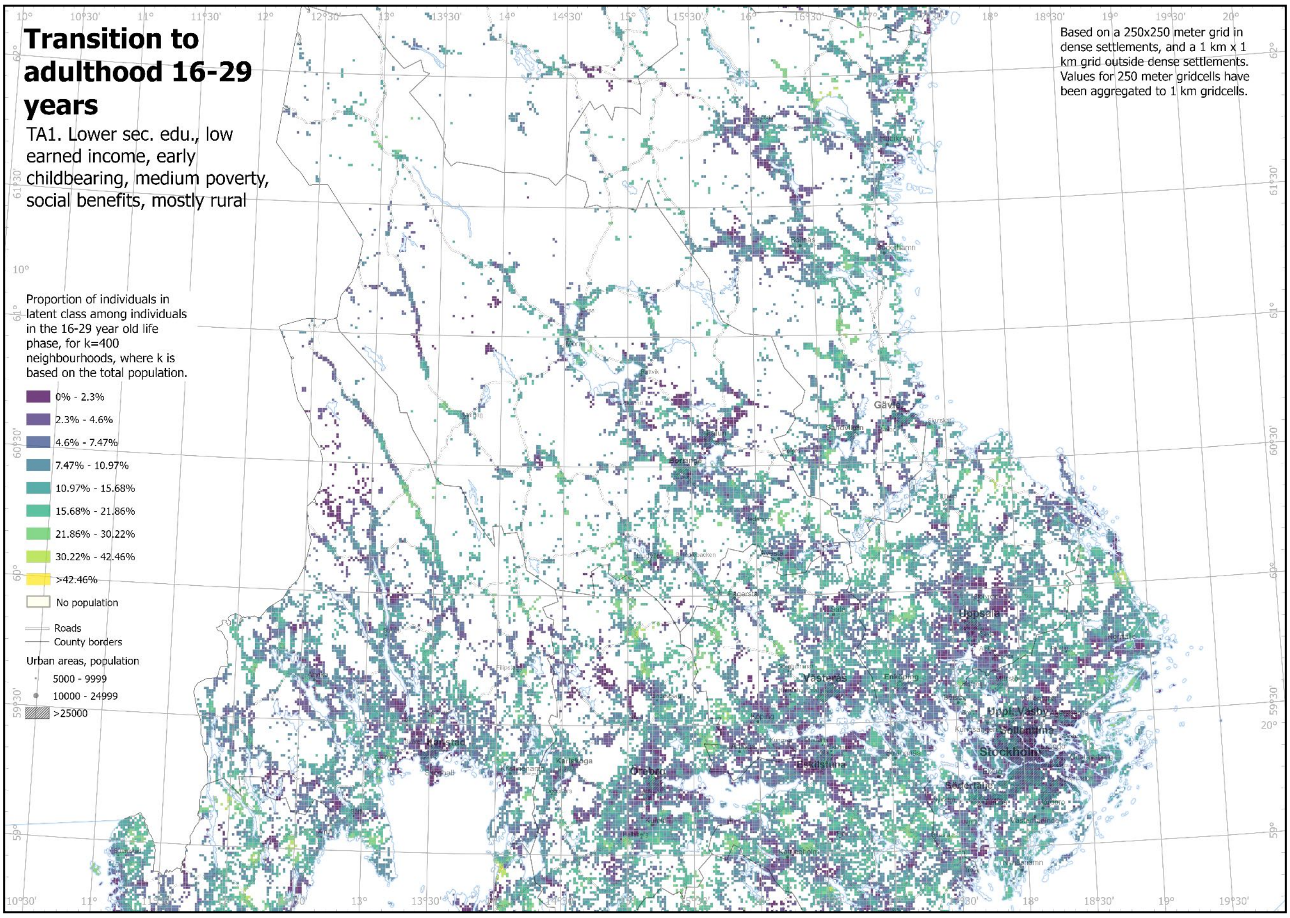

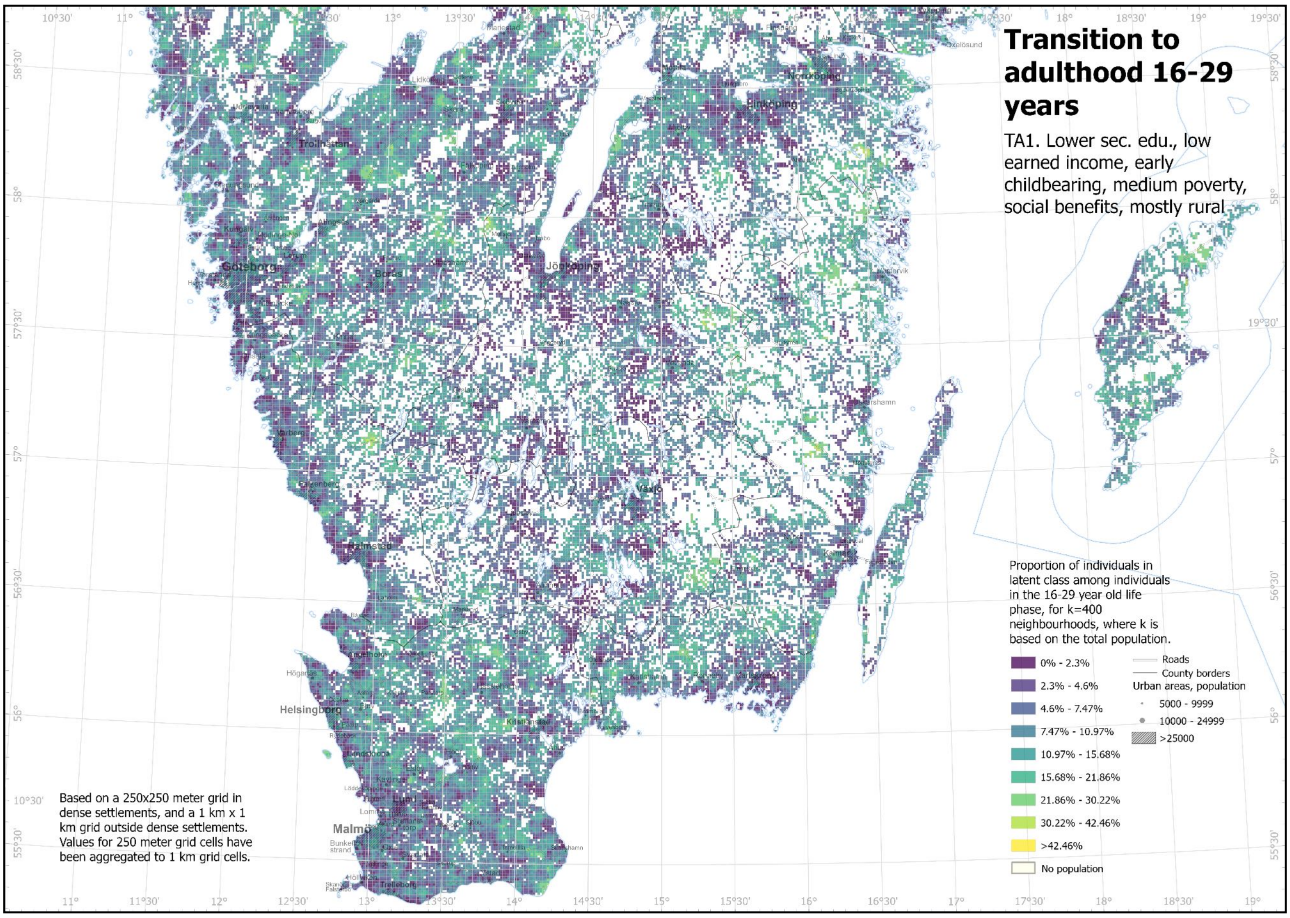

Supplementary Figure S 7 Spatial distribution of disadvantaged life course TA1, continued

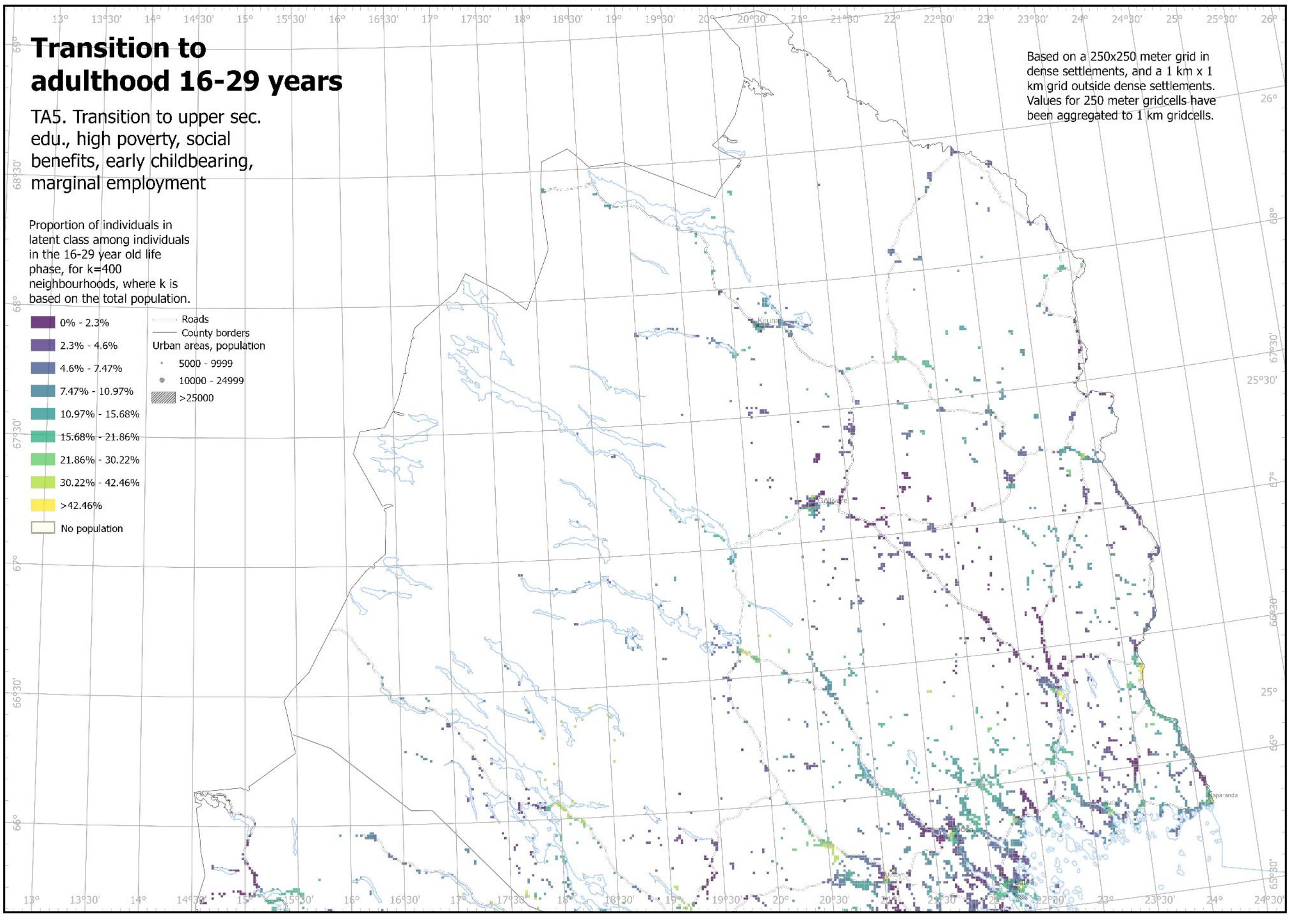

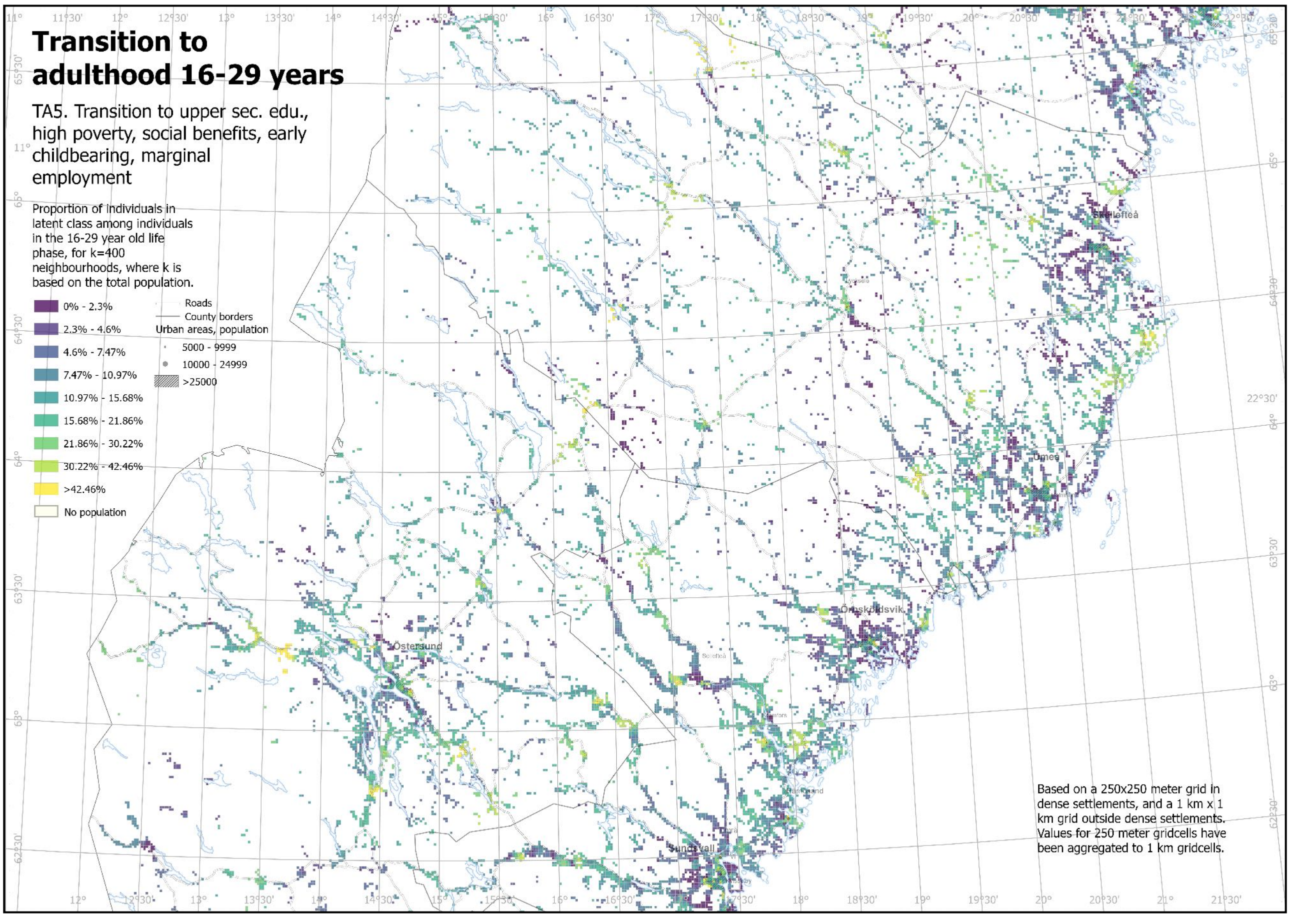

Supplementary Figure S 8 Spatial distribution of disadvantaged life course TA5

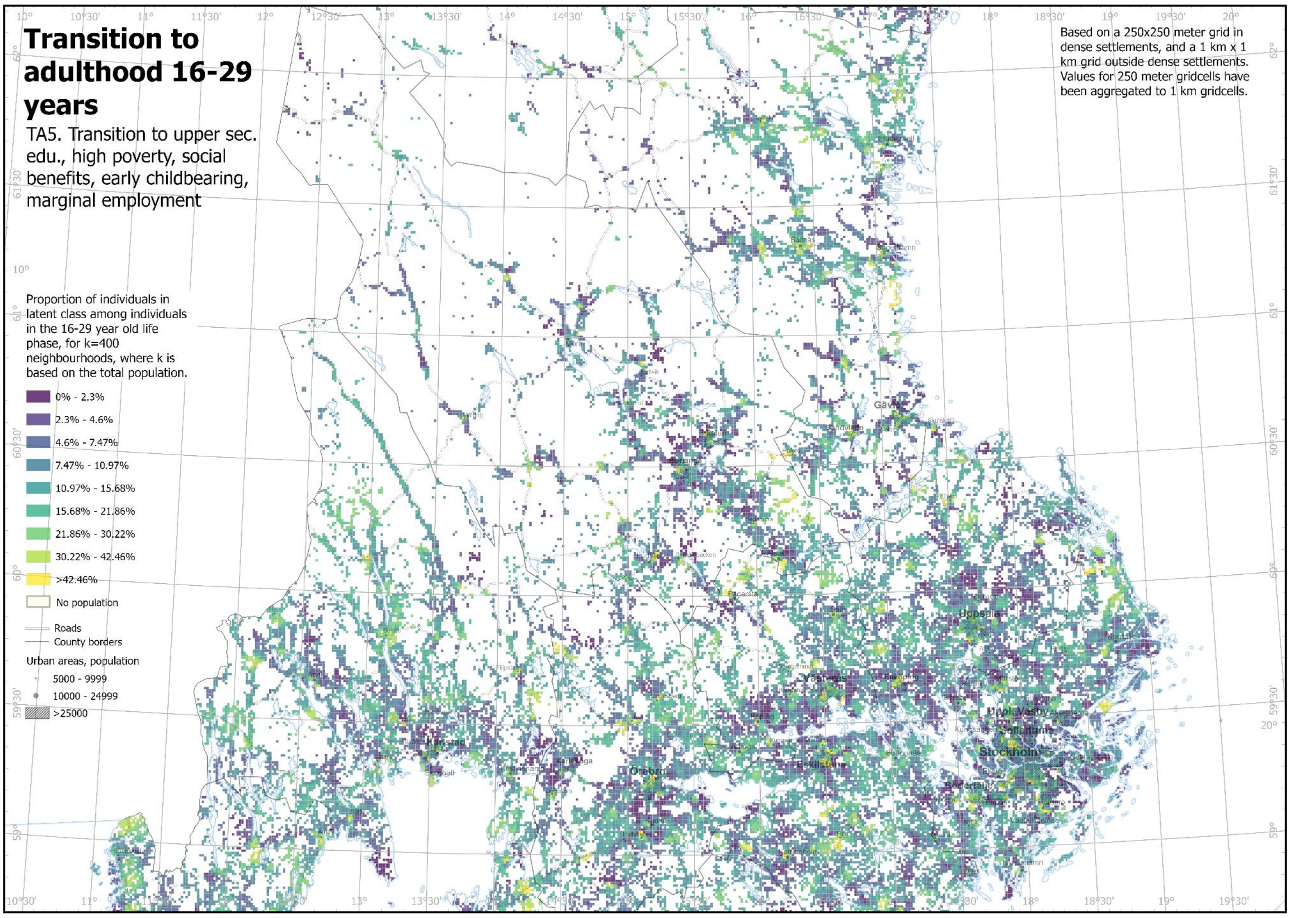

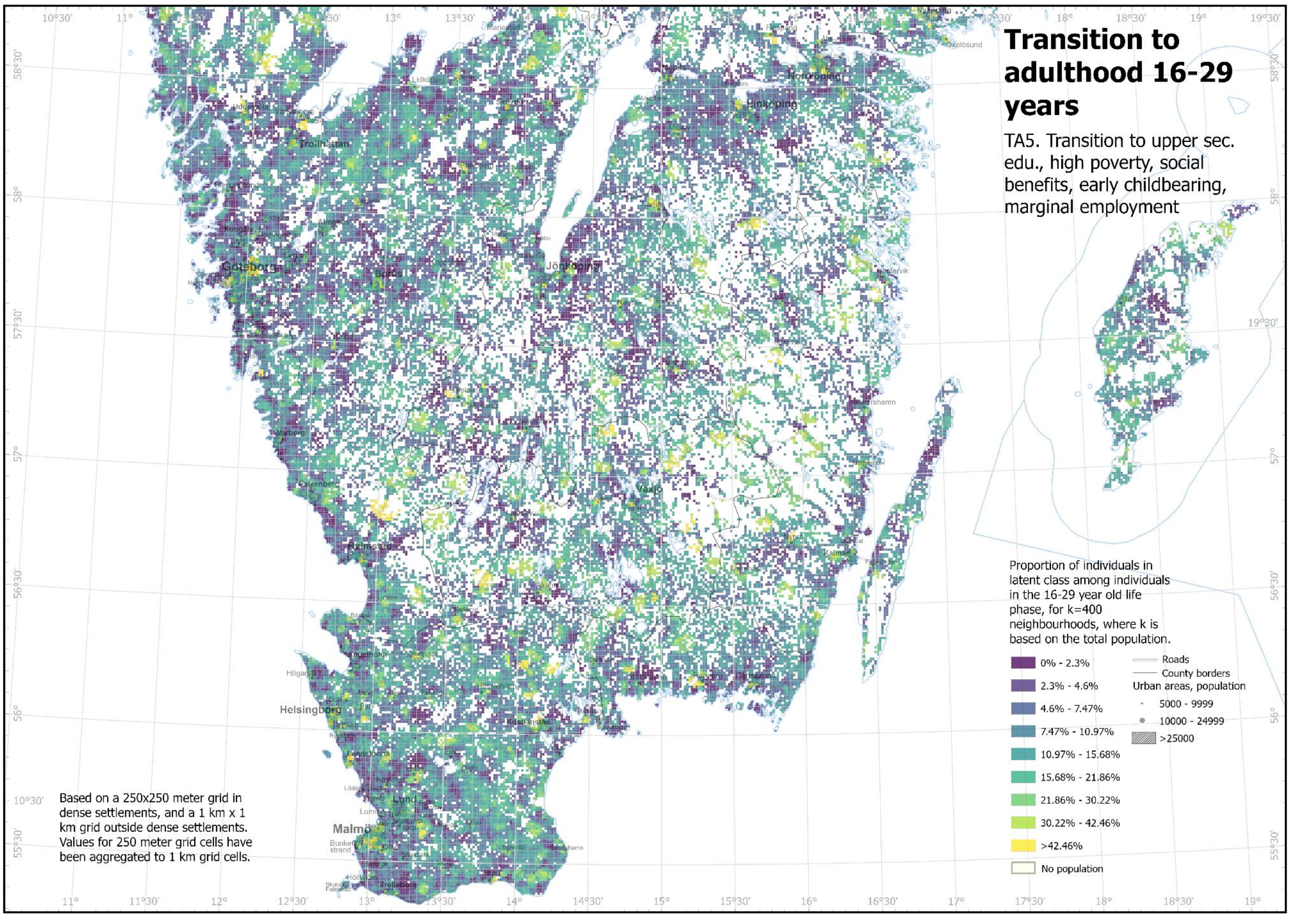

Supplementary Figure S 8 Spatial distribution of disadvantaged life course TA5, continued

Disadvantaged

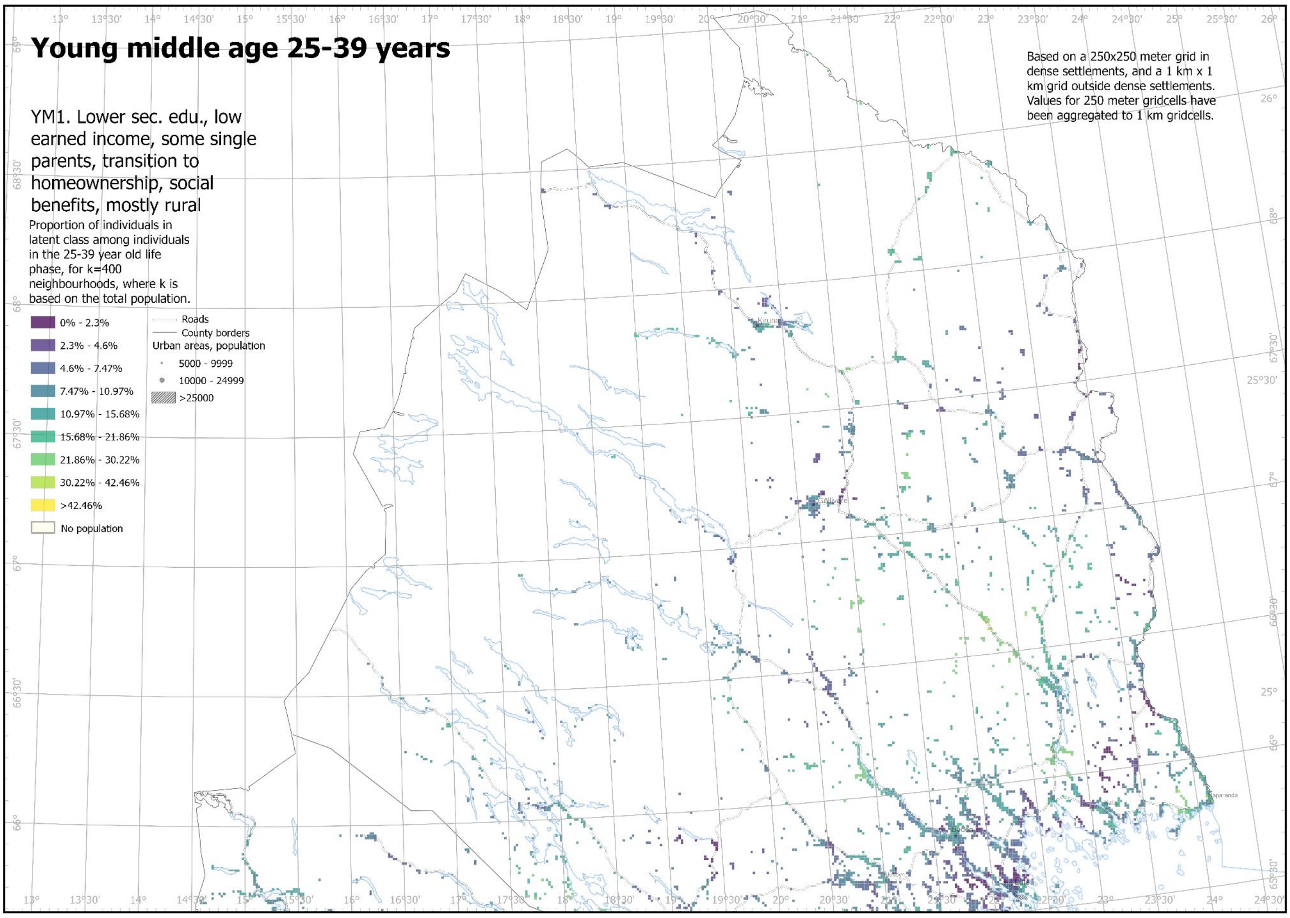

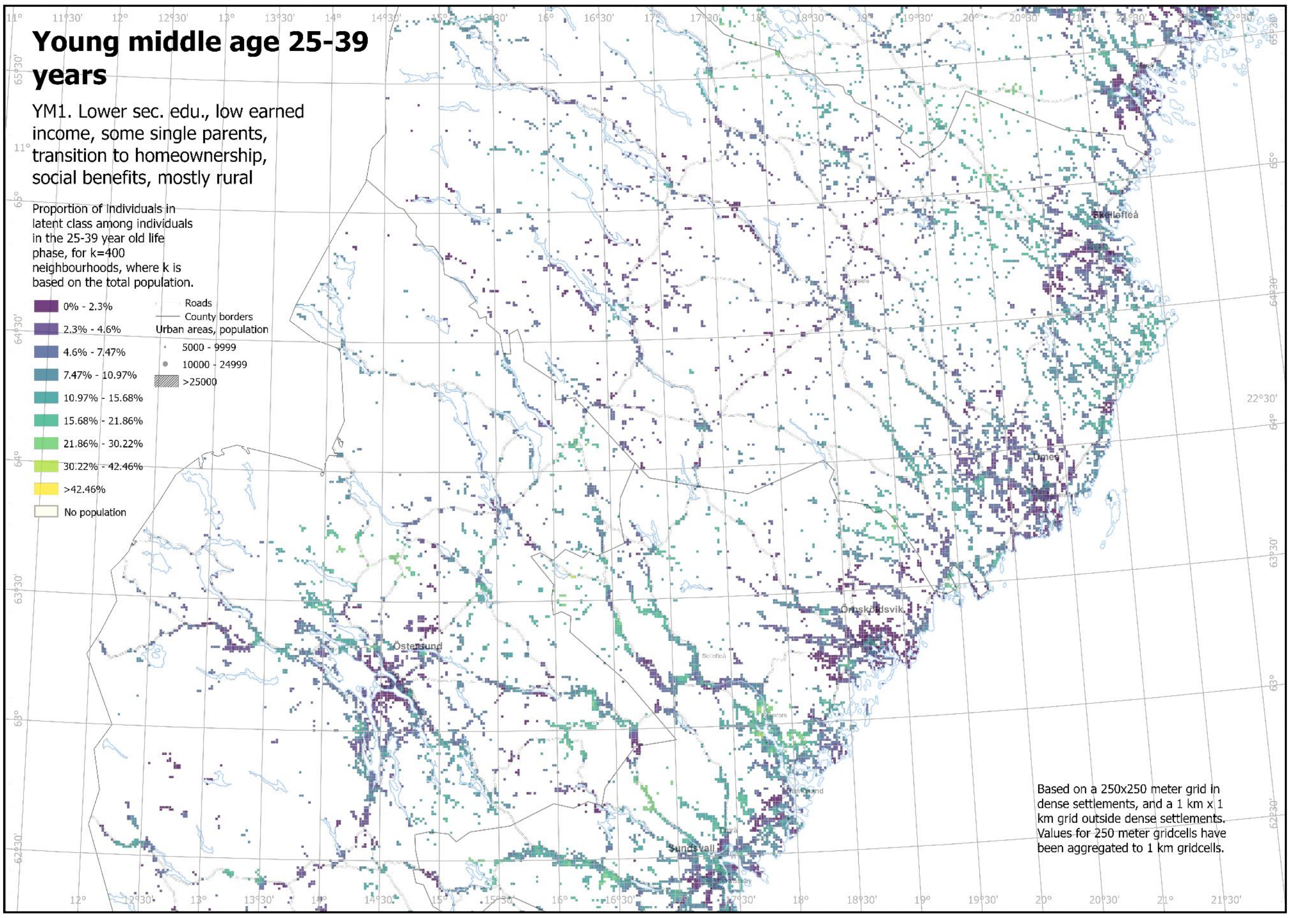

Supplementary Figure S 9 Spatial distribution of disadvantaged life course YM1

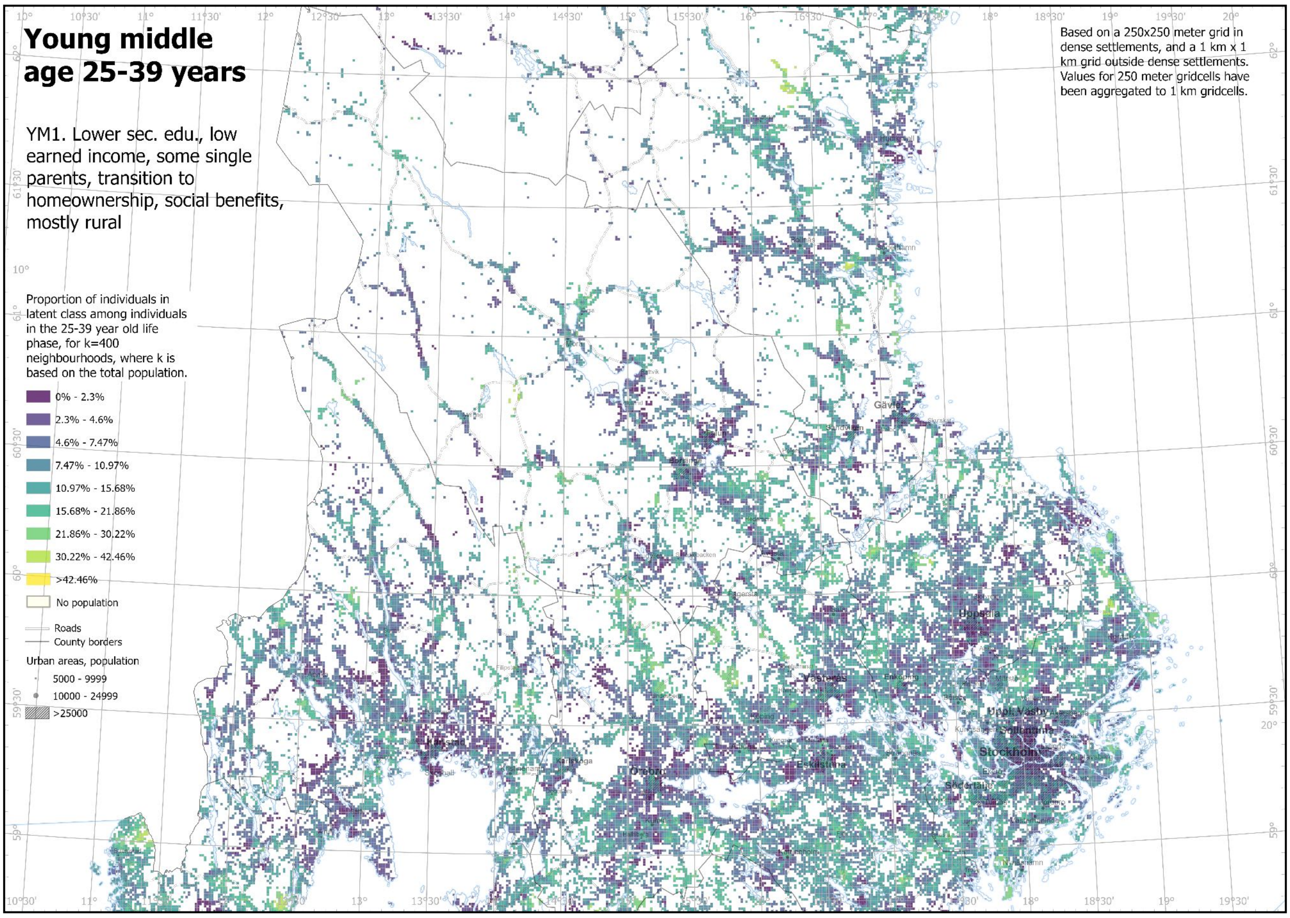

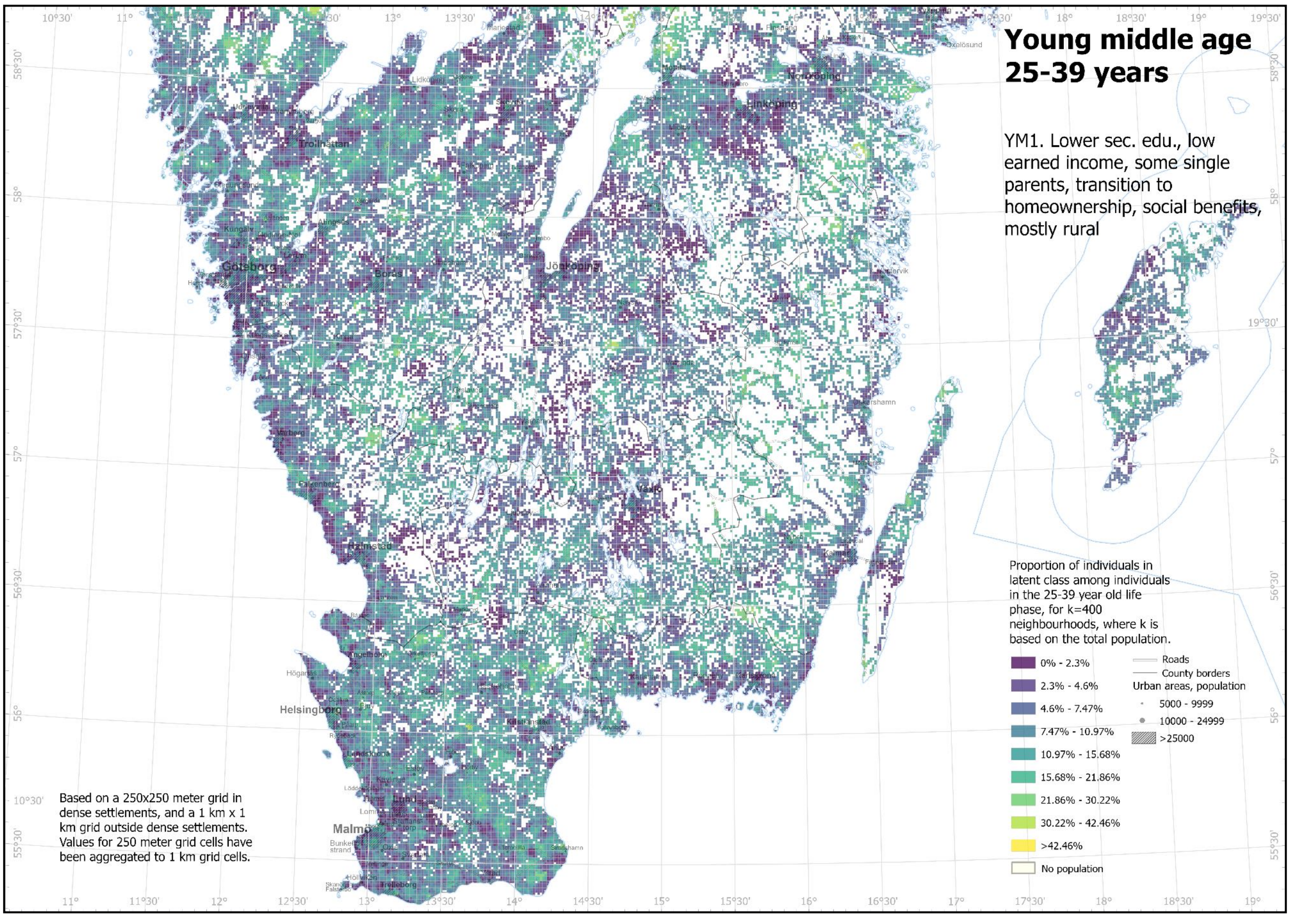

Supplementary Figure S 9 Spatial distribution of disadvantaged life course YM1, continued

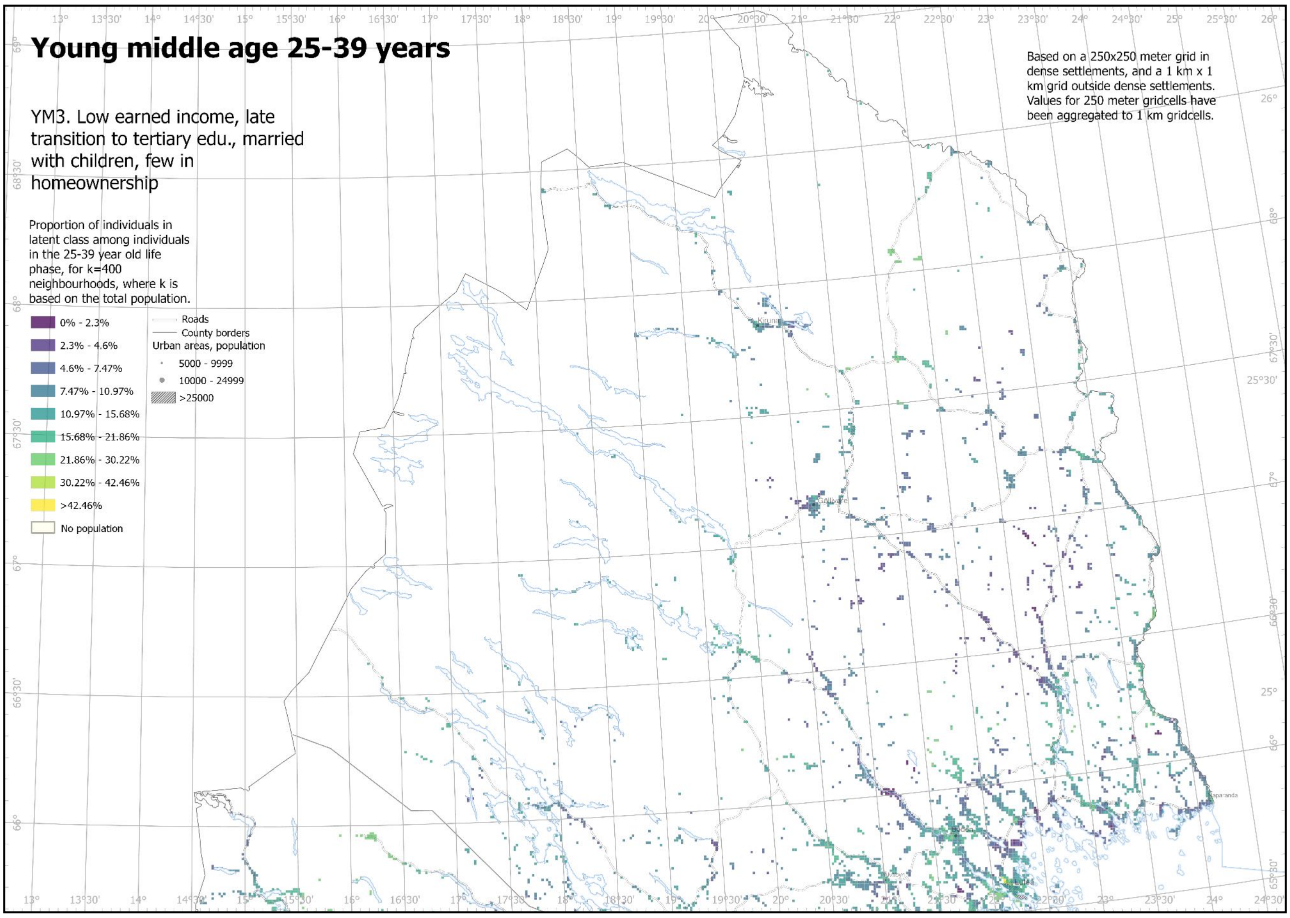

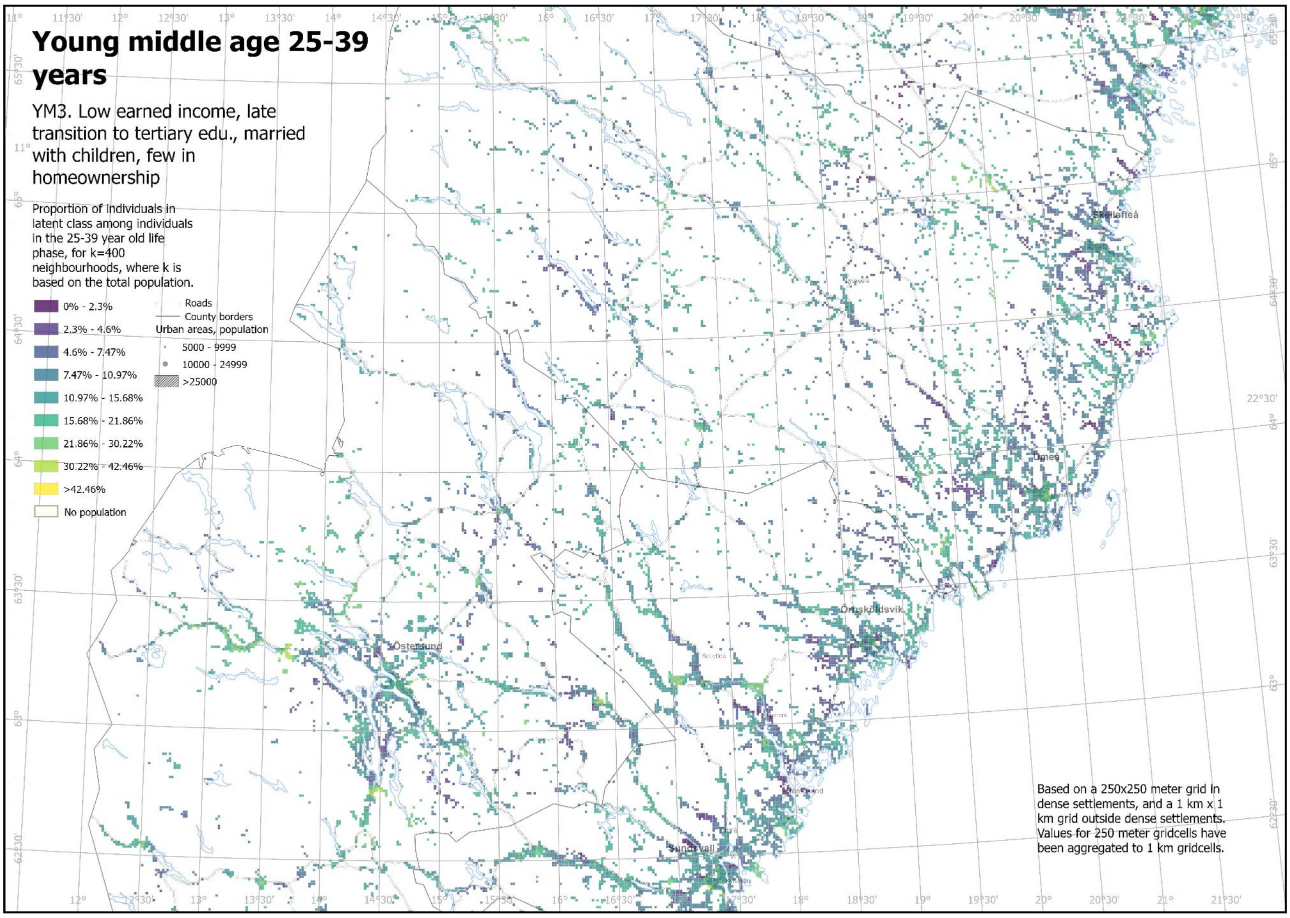

Supplementary Figure S 10 Spatial distribution of disadvantaged life course YM3

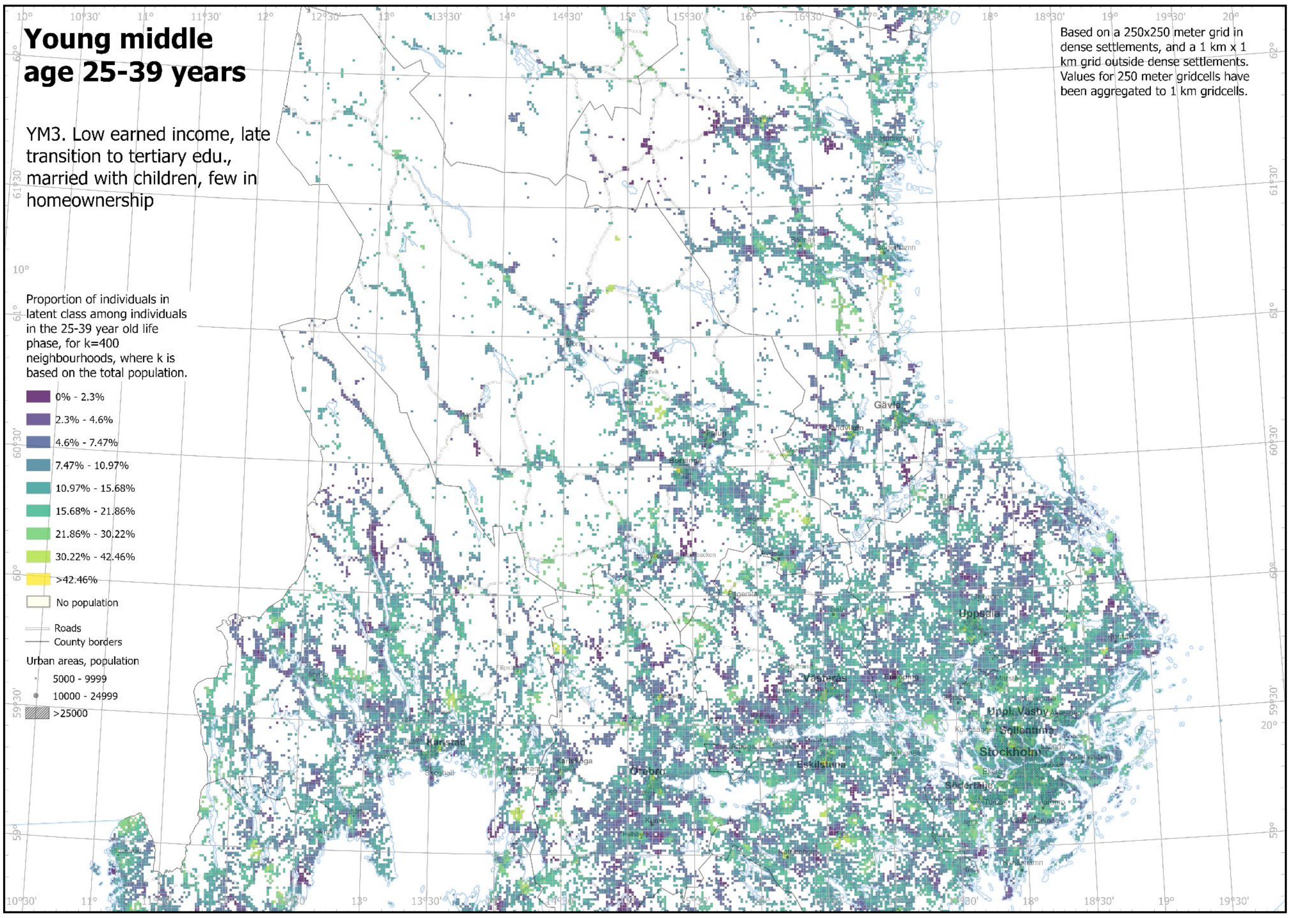

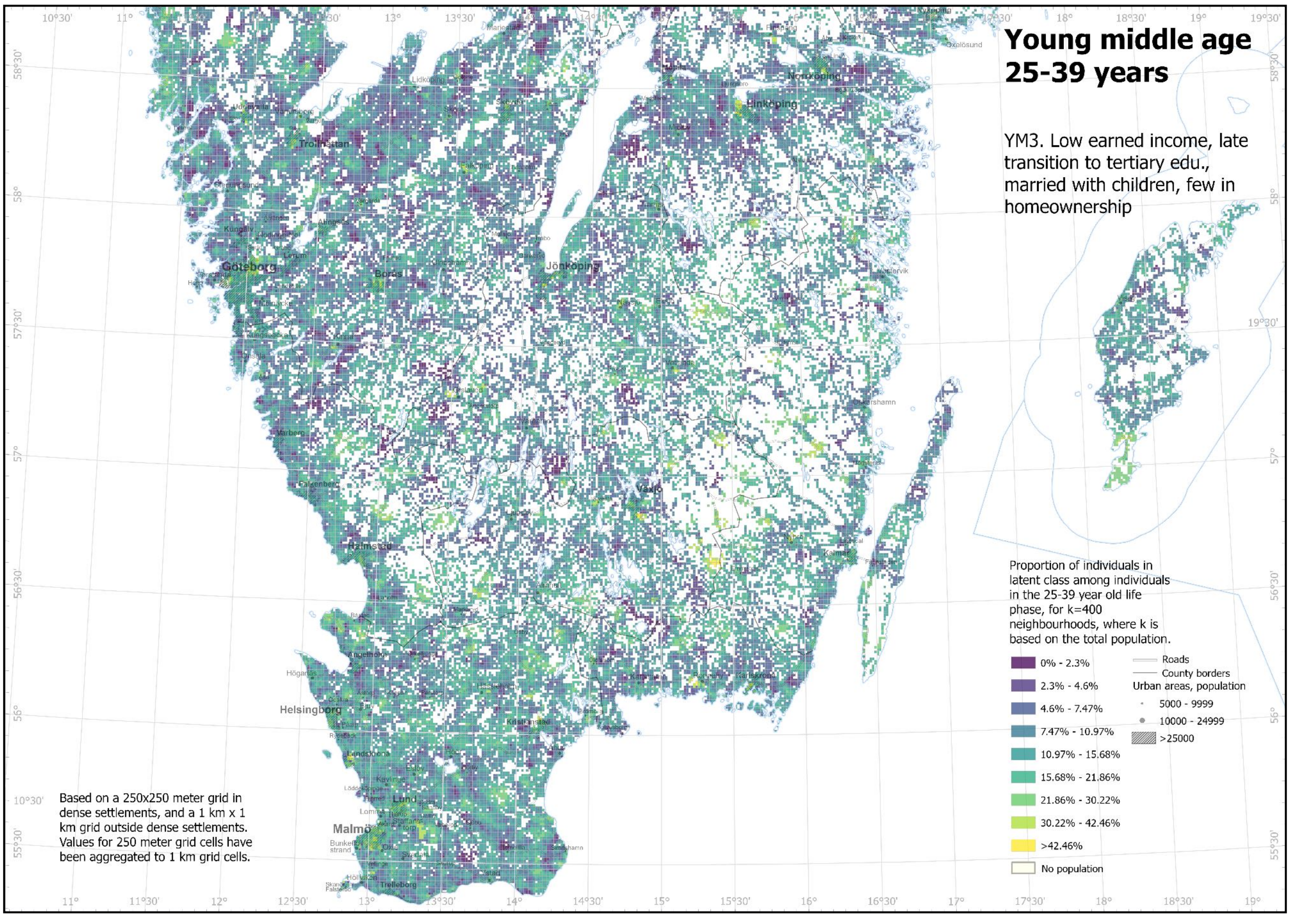

Supplementary Figure S 10 Spatial distribution of disadvantaged life course YM3, continued

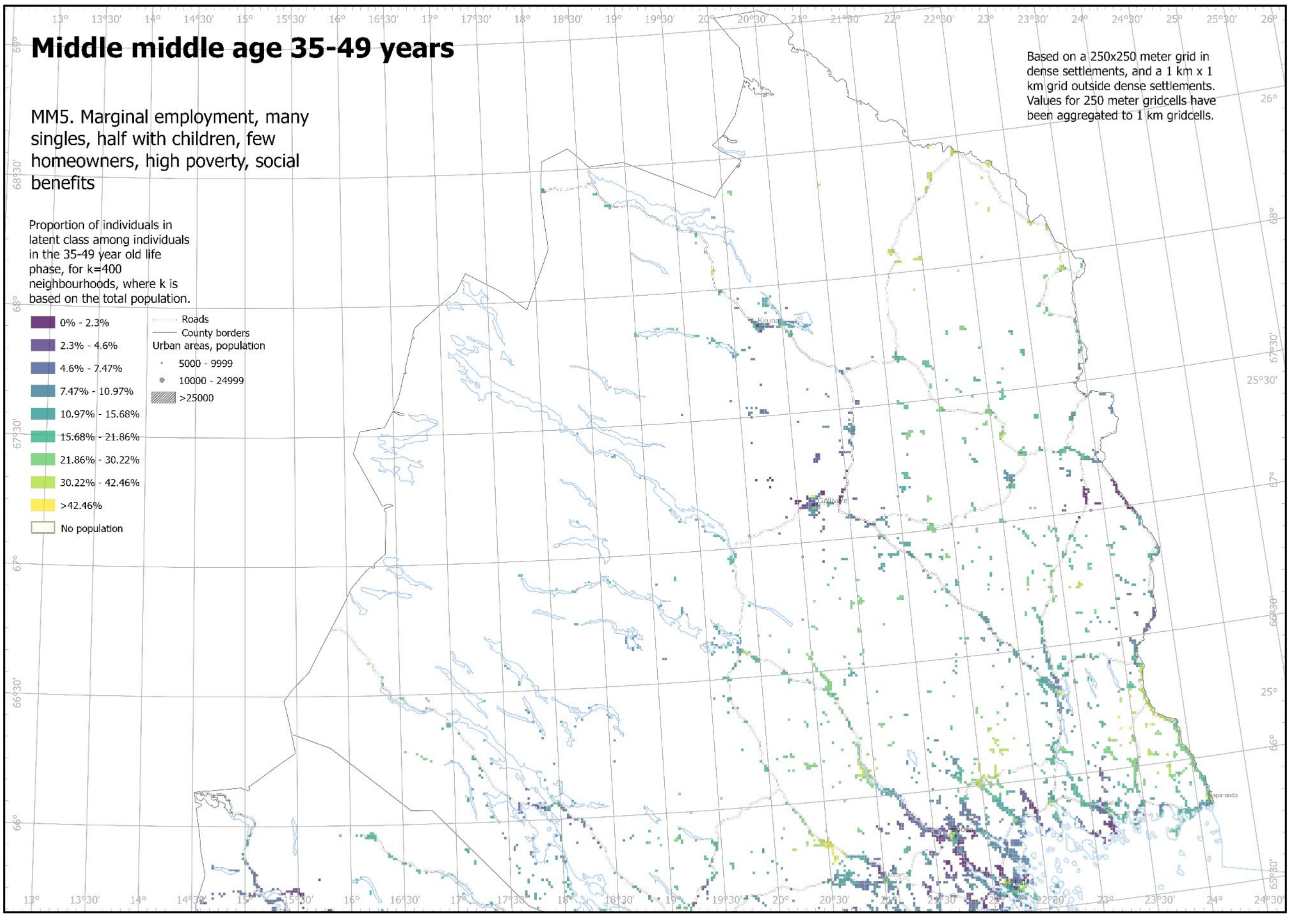

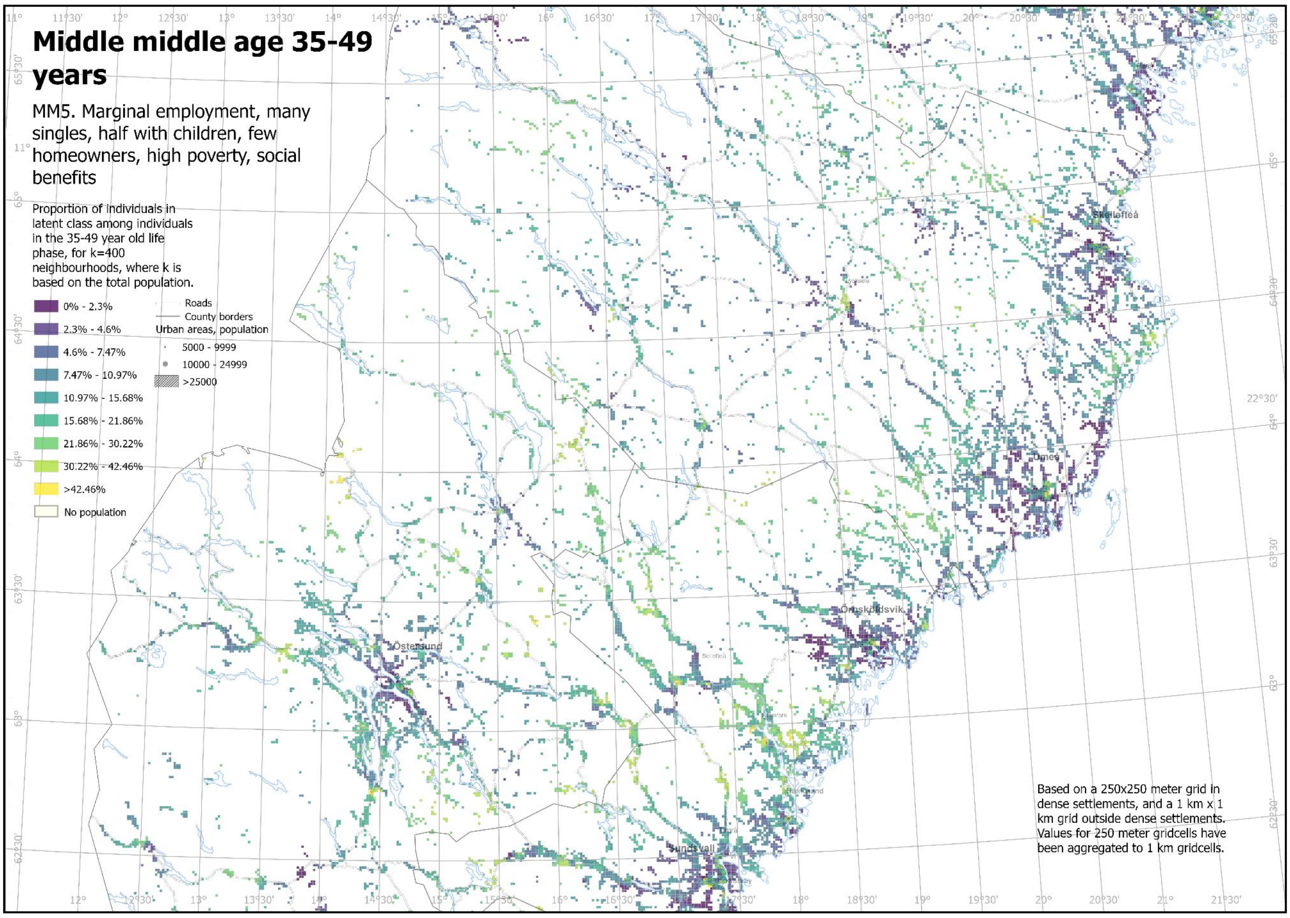

Supplementary Figure S 11 Spatial distribution of disadvantaged life course MM5

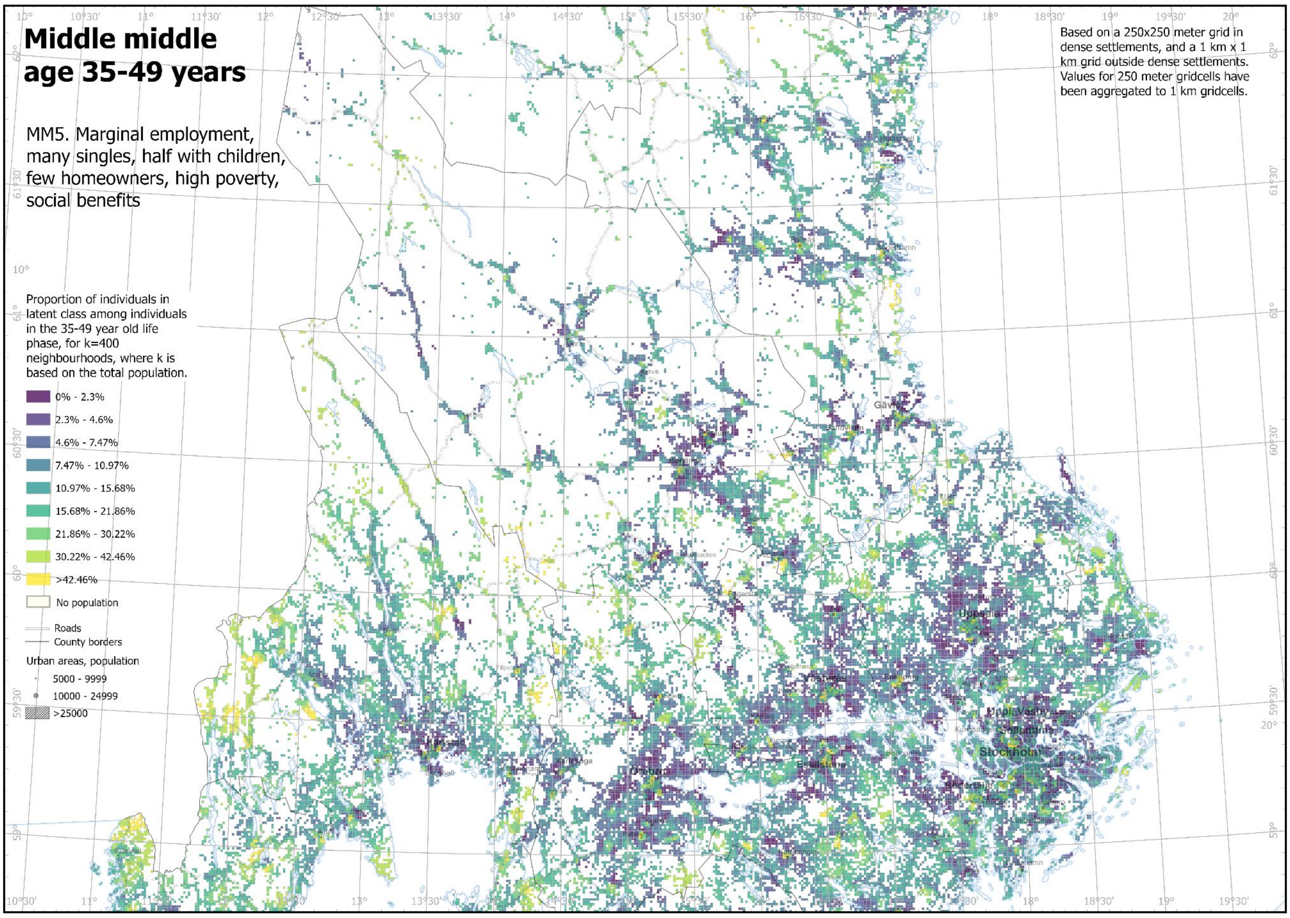

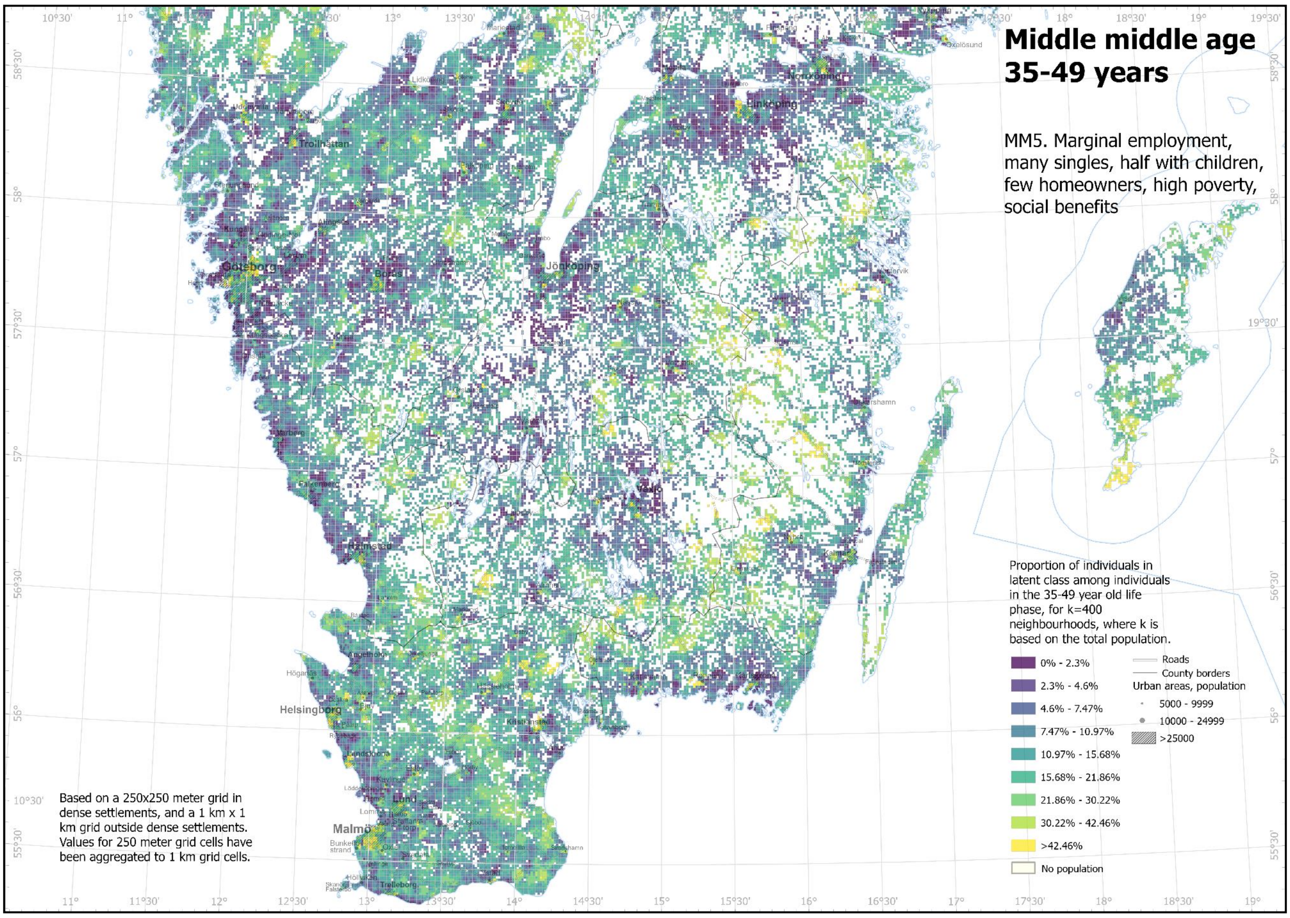

Supplementary Figure S 11 Spatial distribution of disadvantaged life course MM5, continued

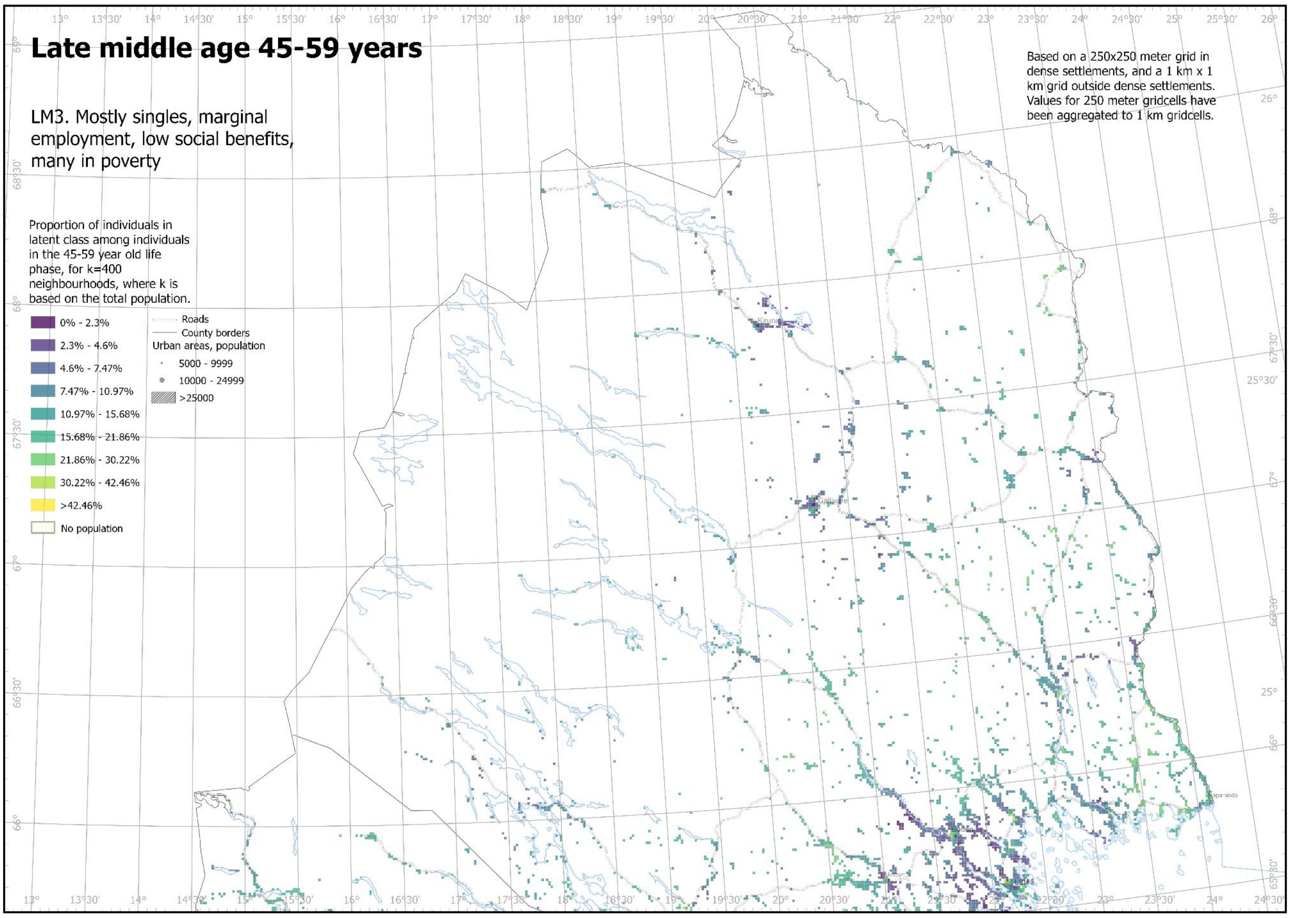

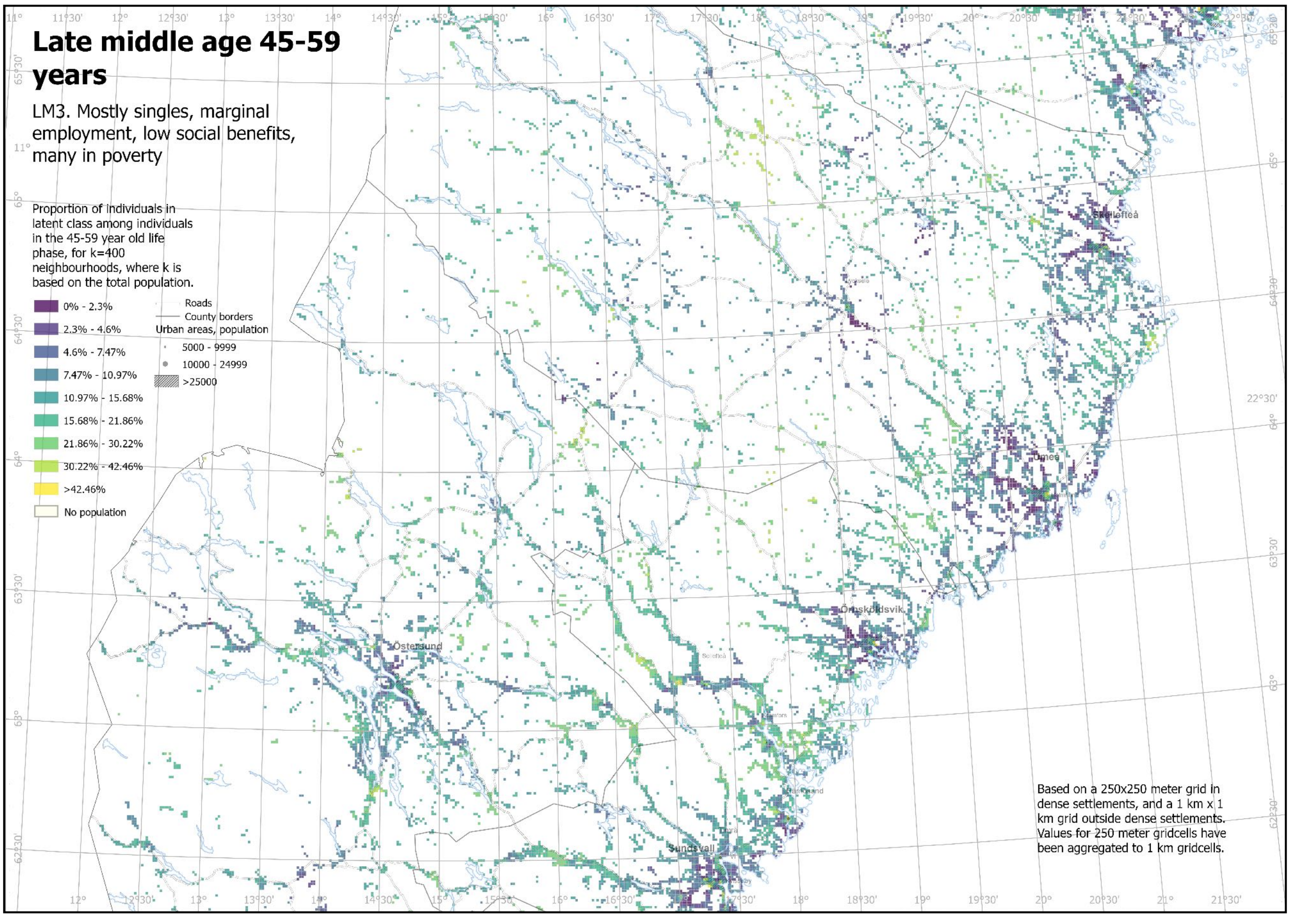

Supplementary Figure S 12 Spatial distribution of disadvantaged life course LM3

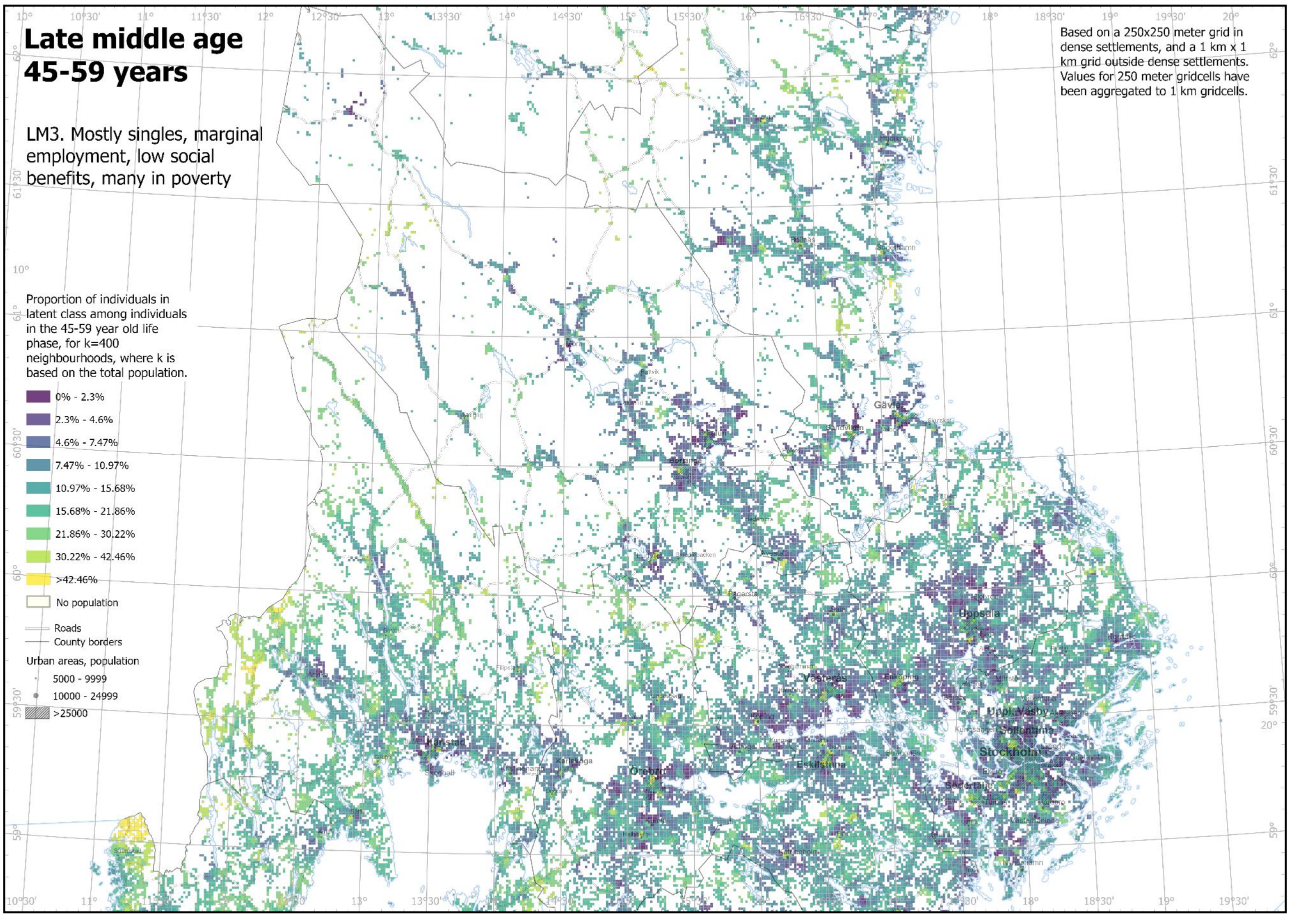

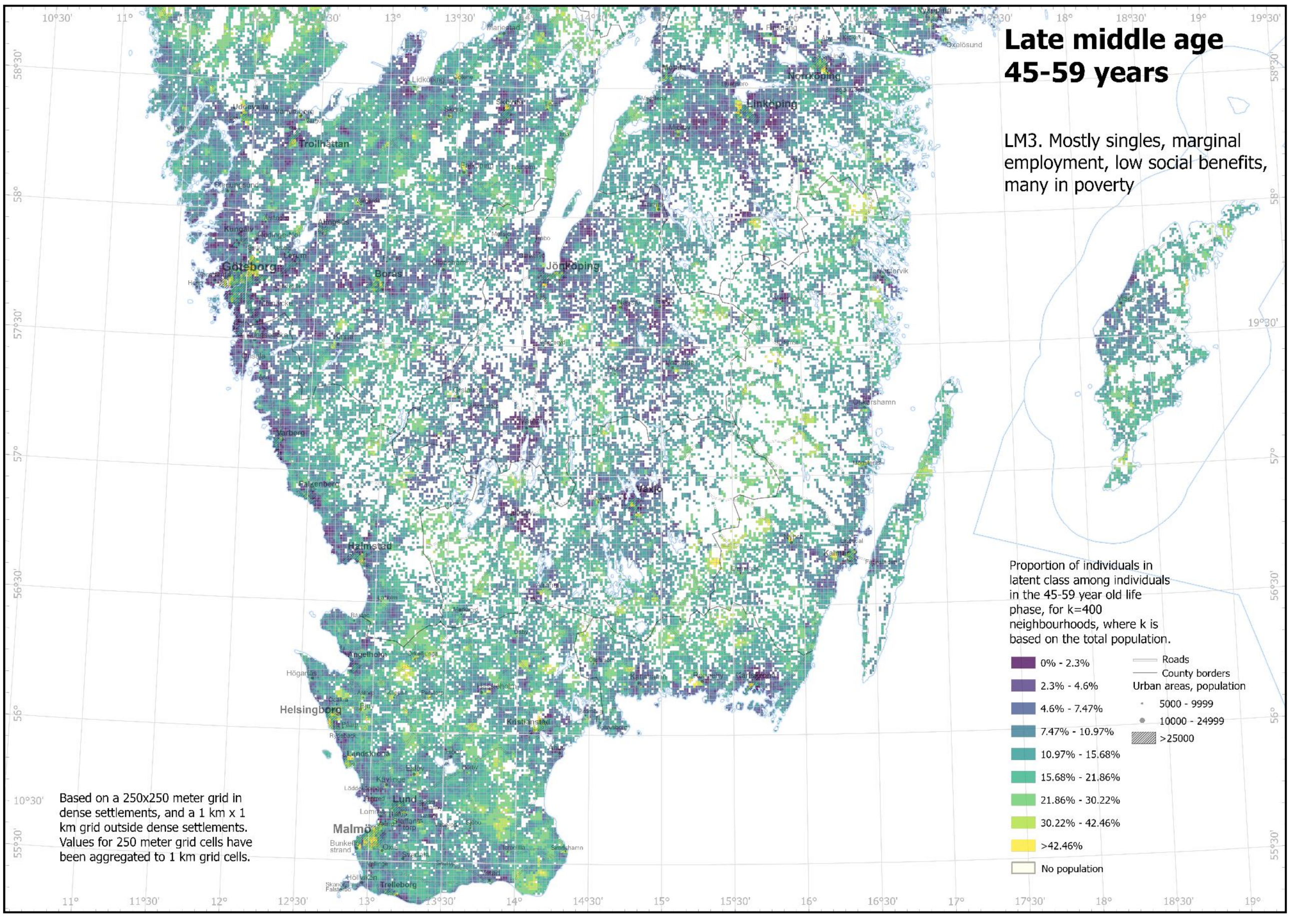

Supplementary Figure S 12 Spatial distribution of disadvantaged life course LM3, continued

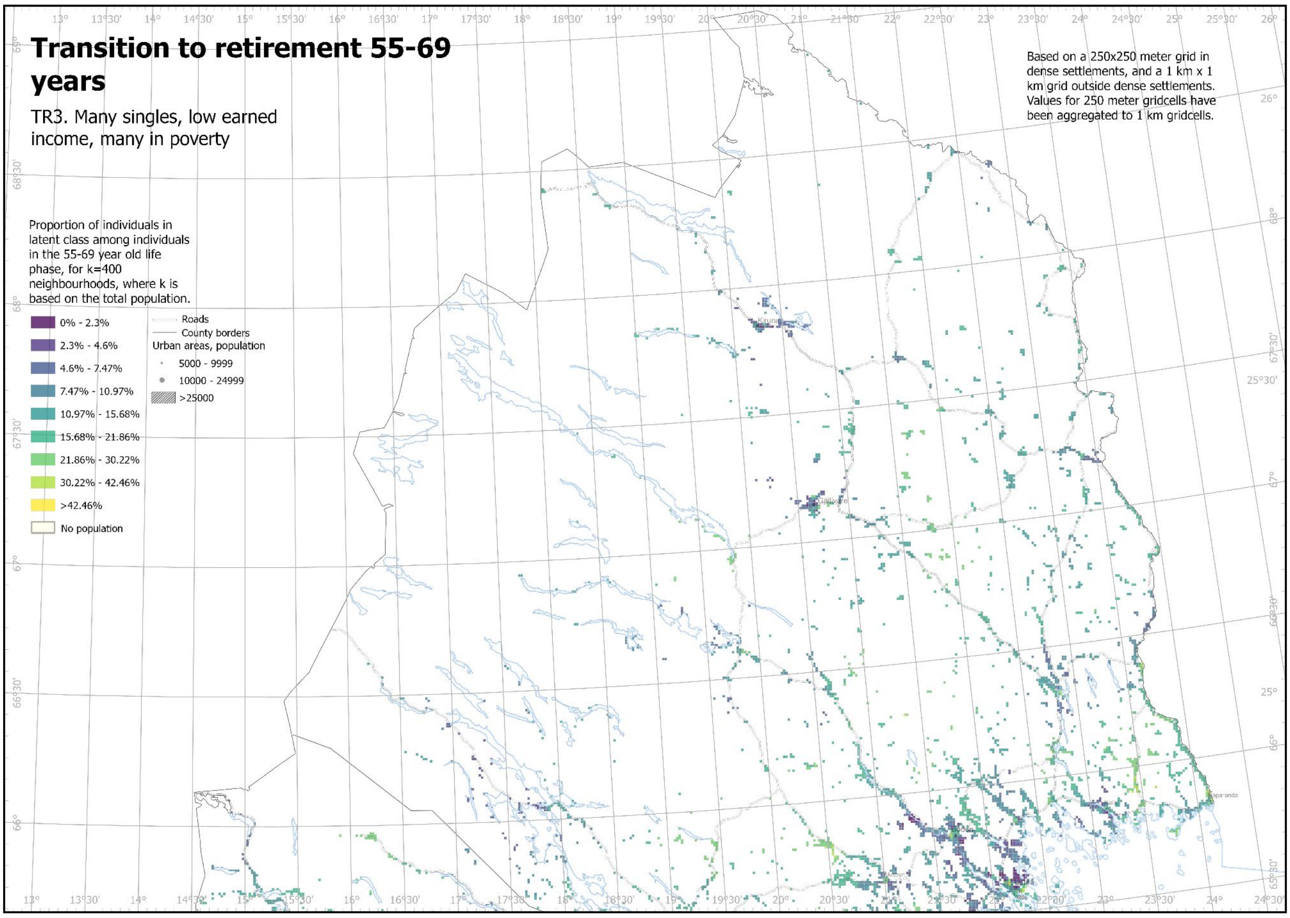

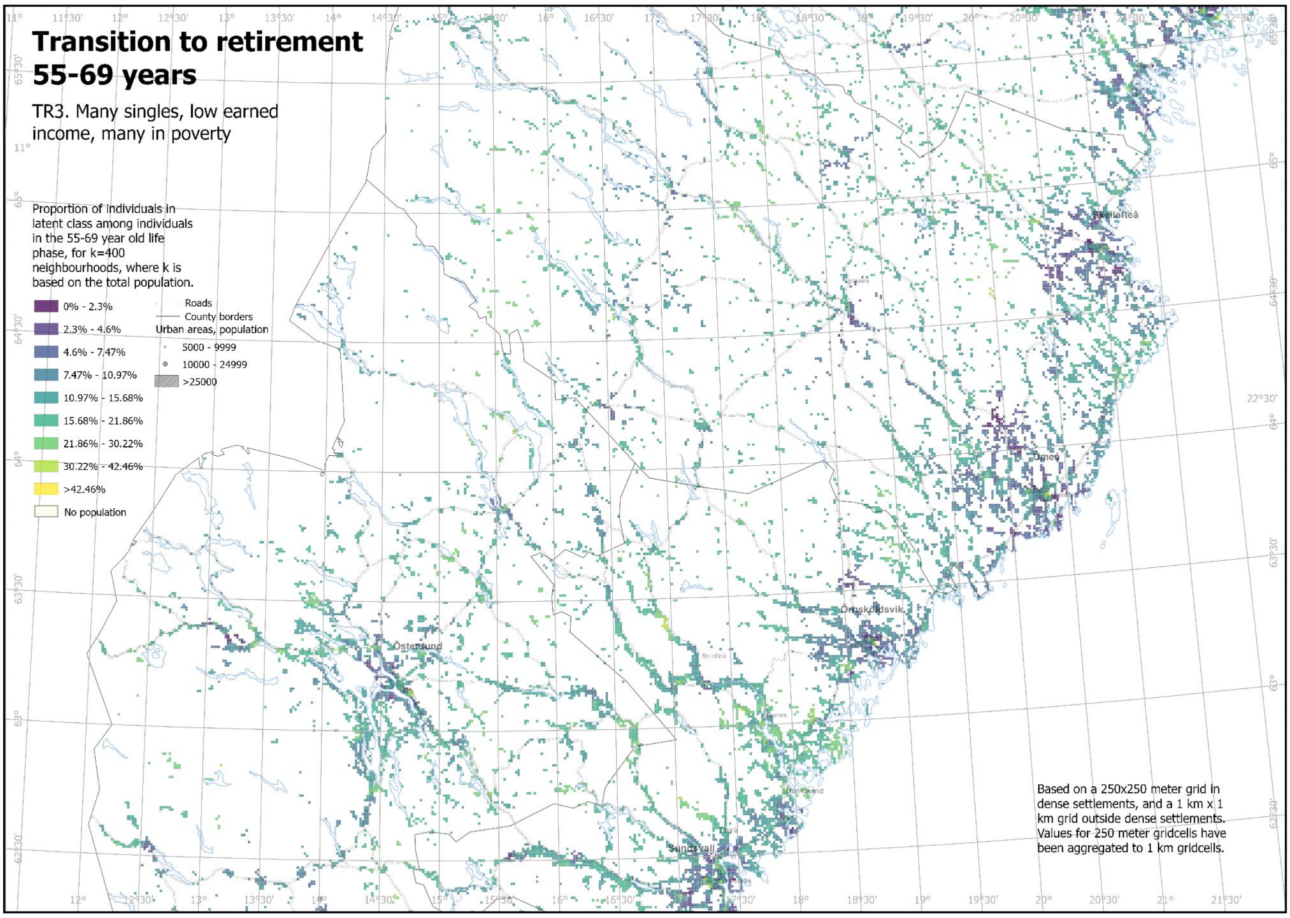

Supplementary Figure S 13 Spatial distribution of disadvantaged life course TR3

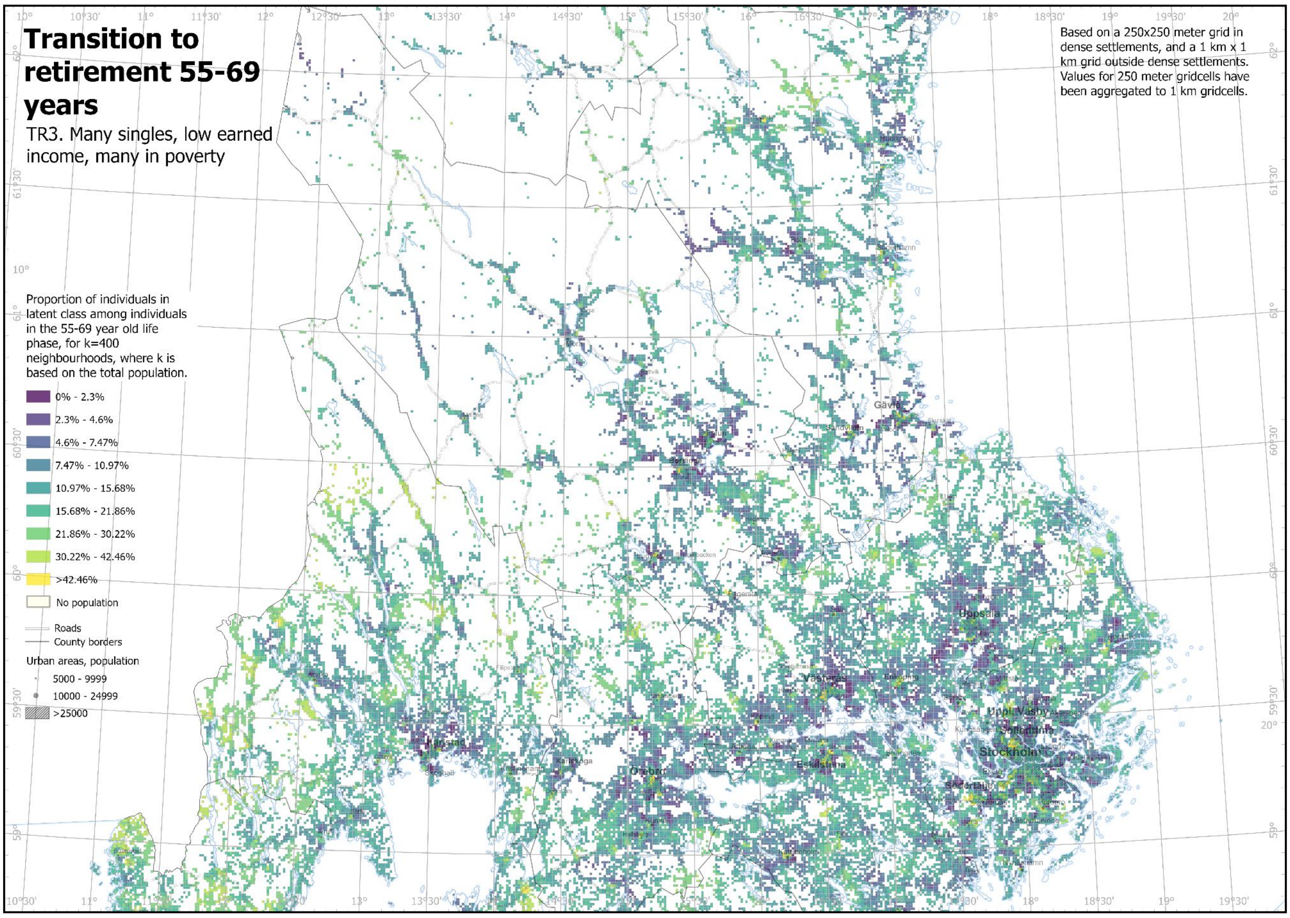

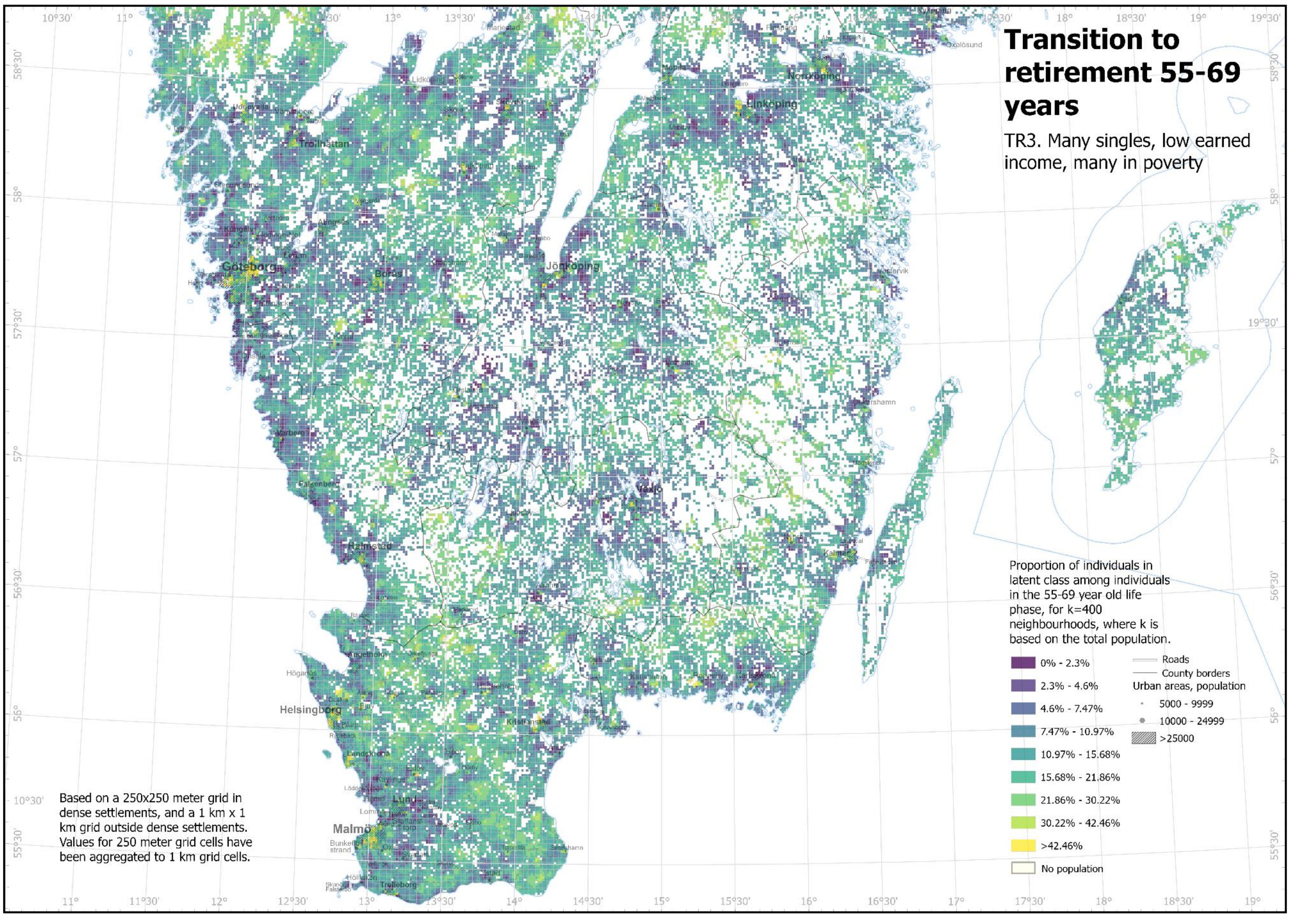

Supplementary Figure S 13 Spatial distribution of disadvantaged life course TR3, continued

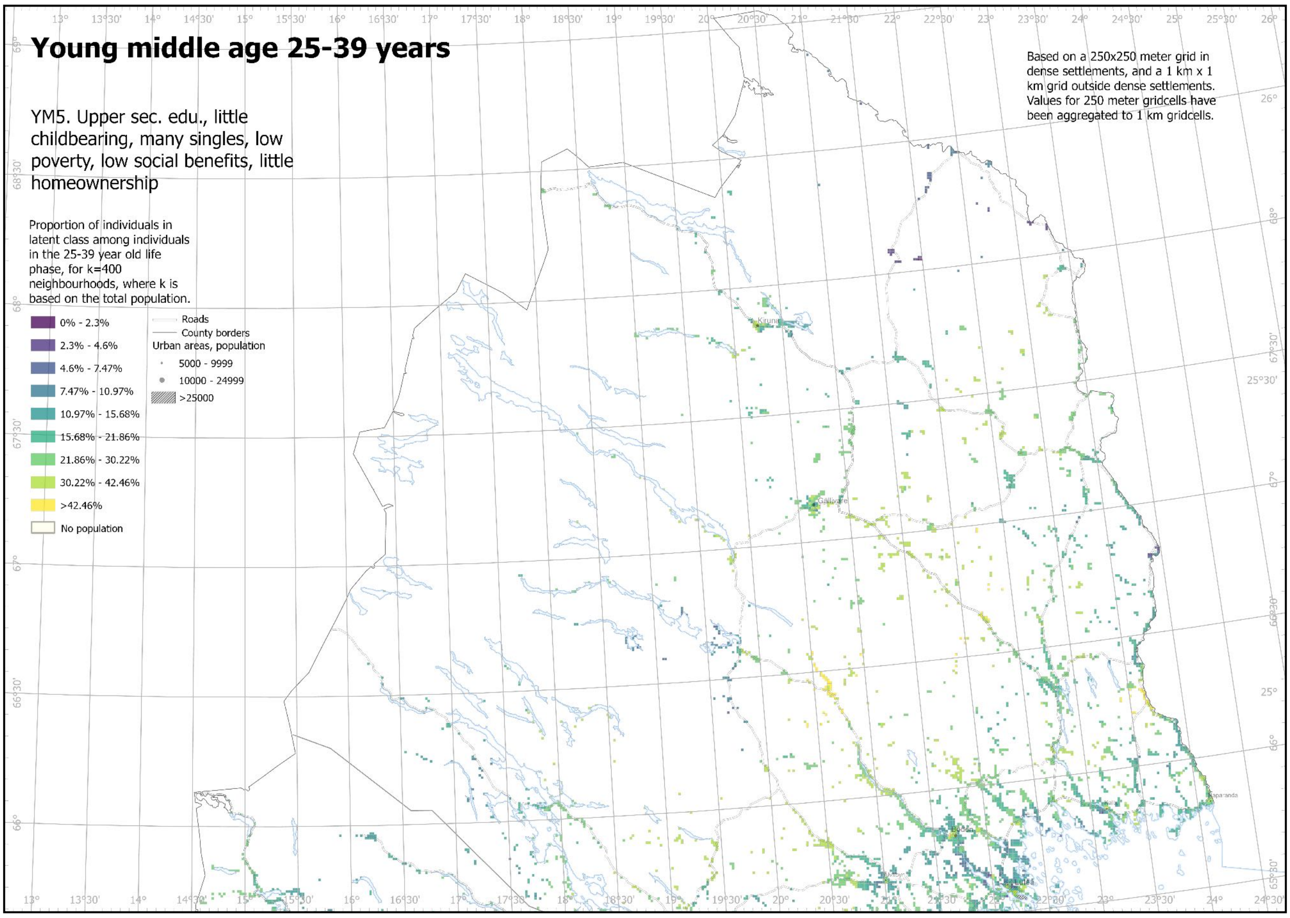

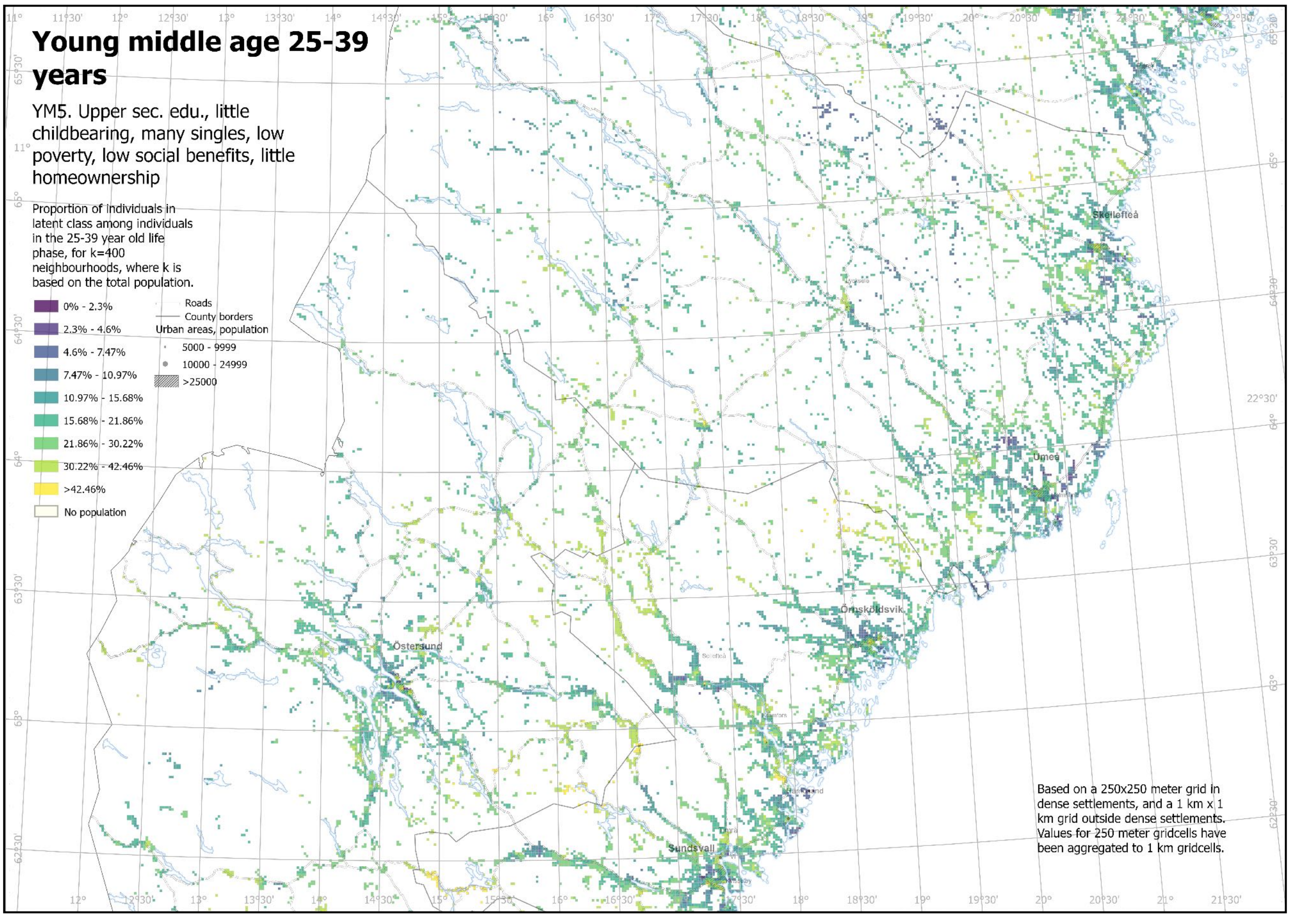

Supplementary Figure S 14 Spatial distribution middle income singles life course YM5

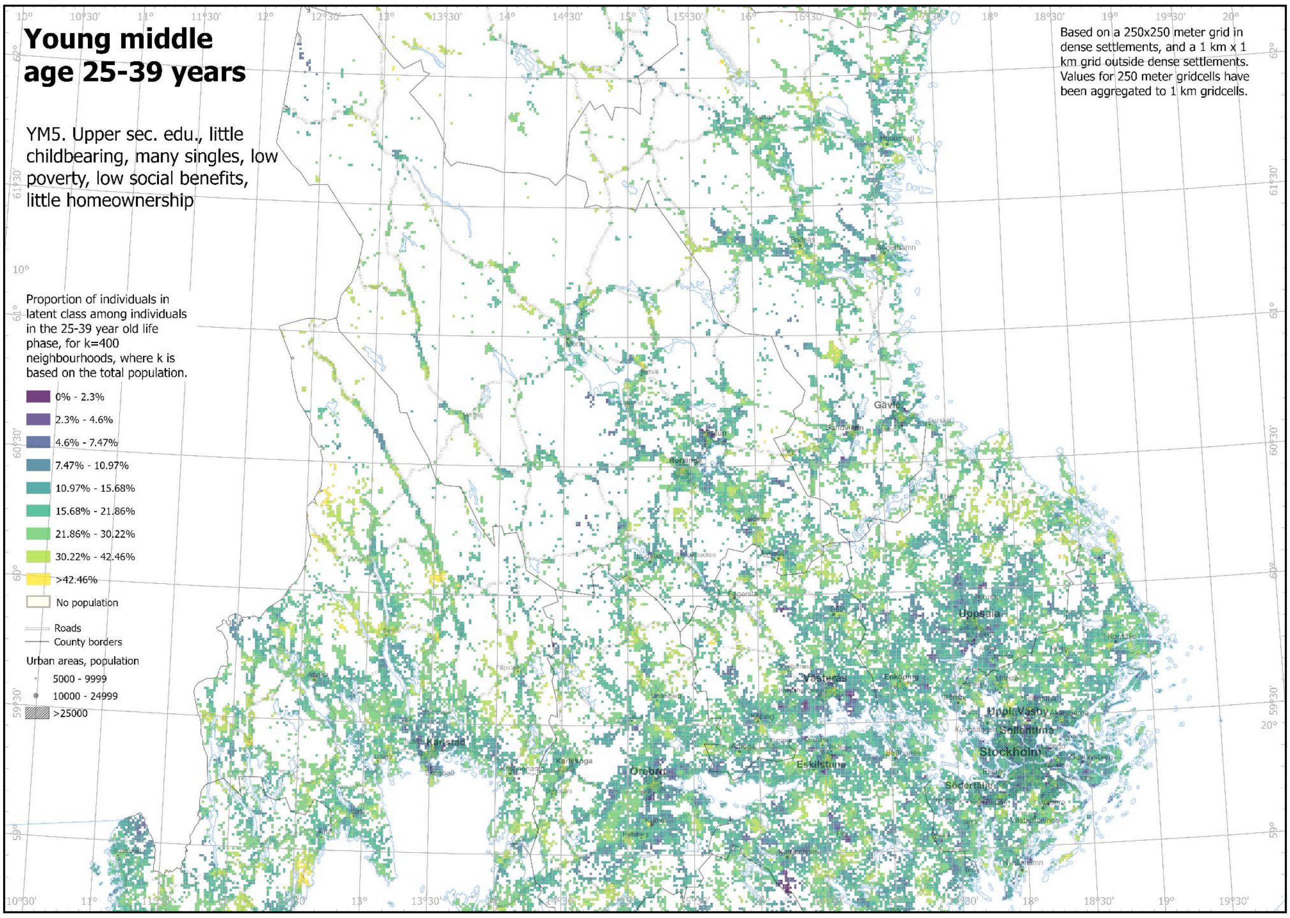

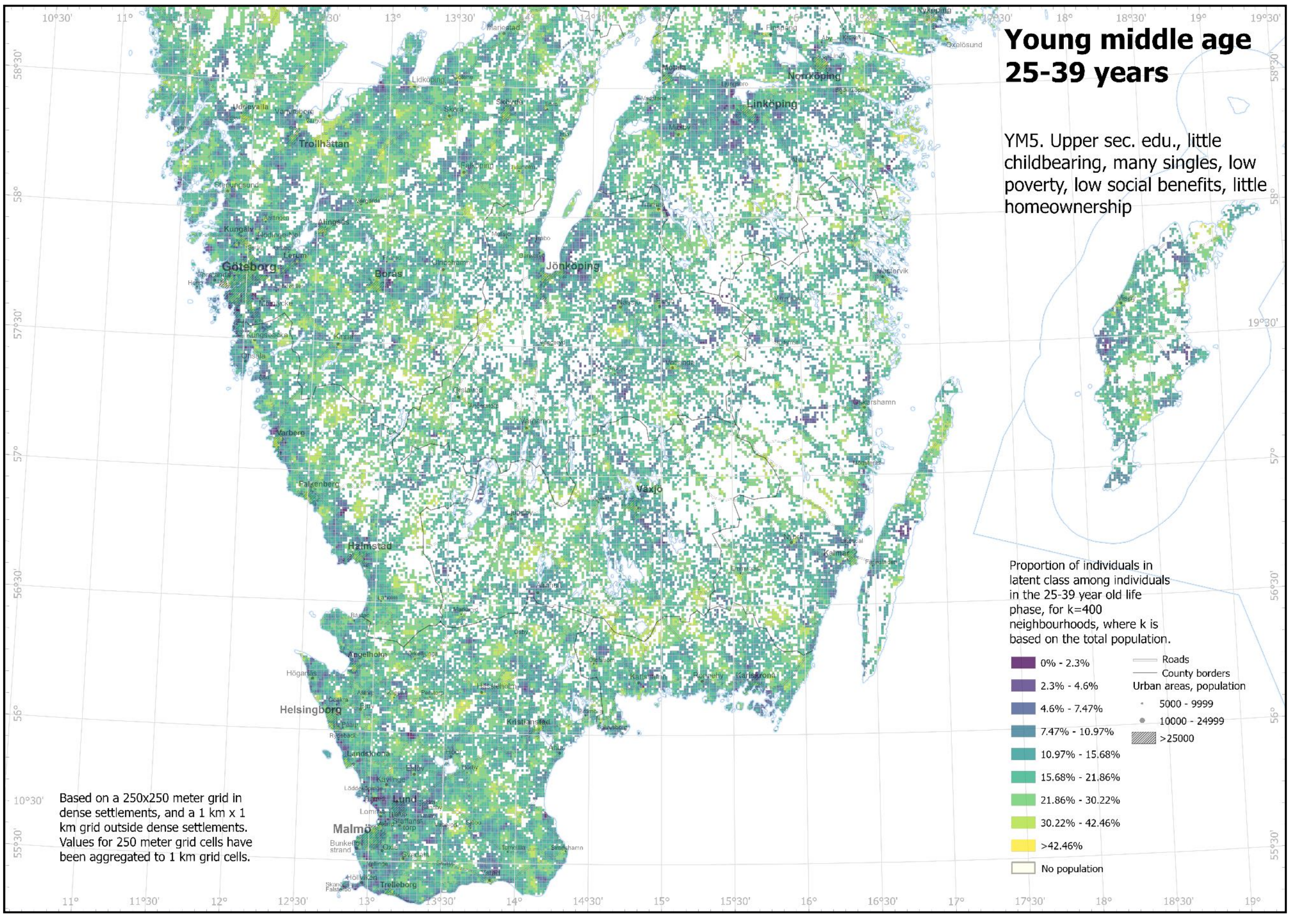

Supplementary Figure S 14 Spatial distribution middle income singles life course YM5, continued

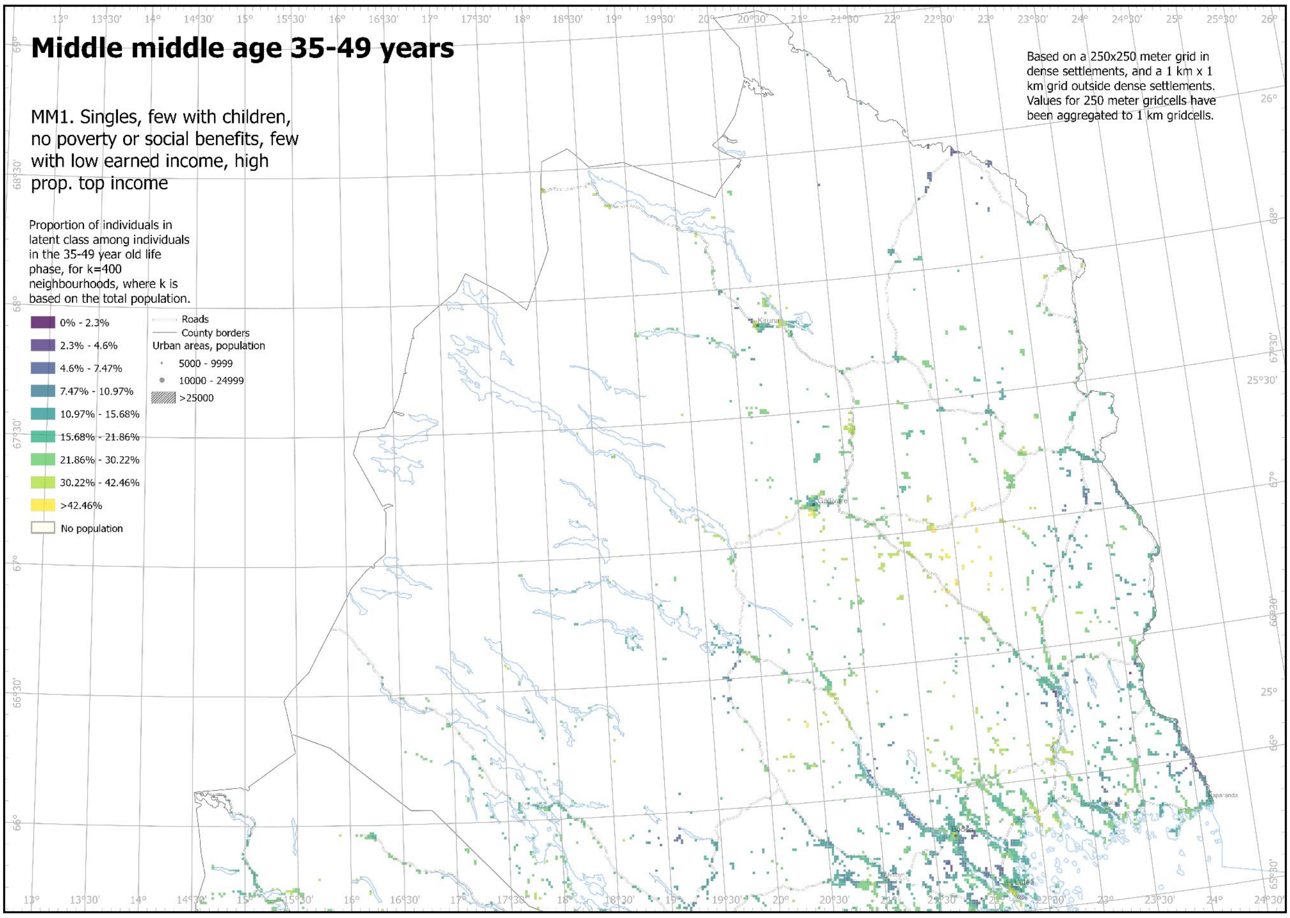

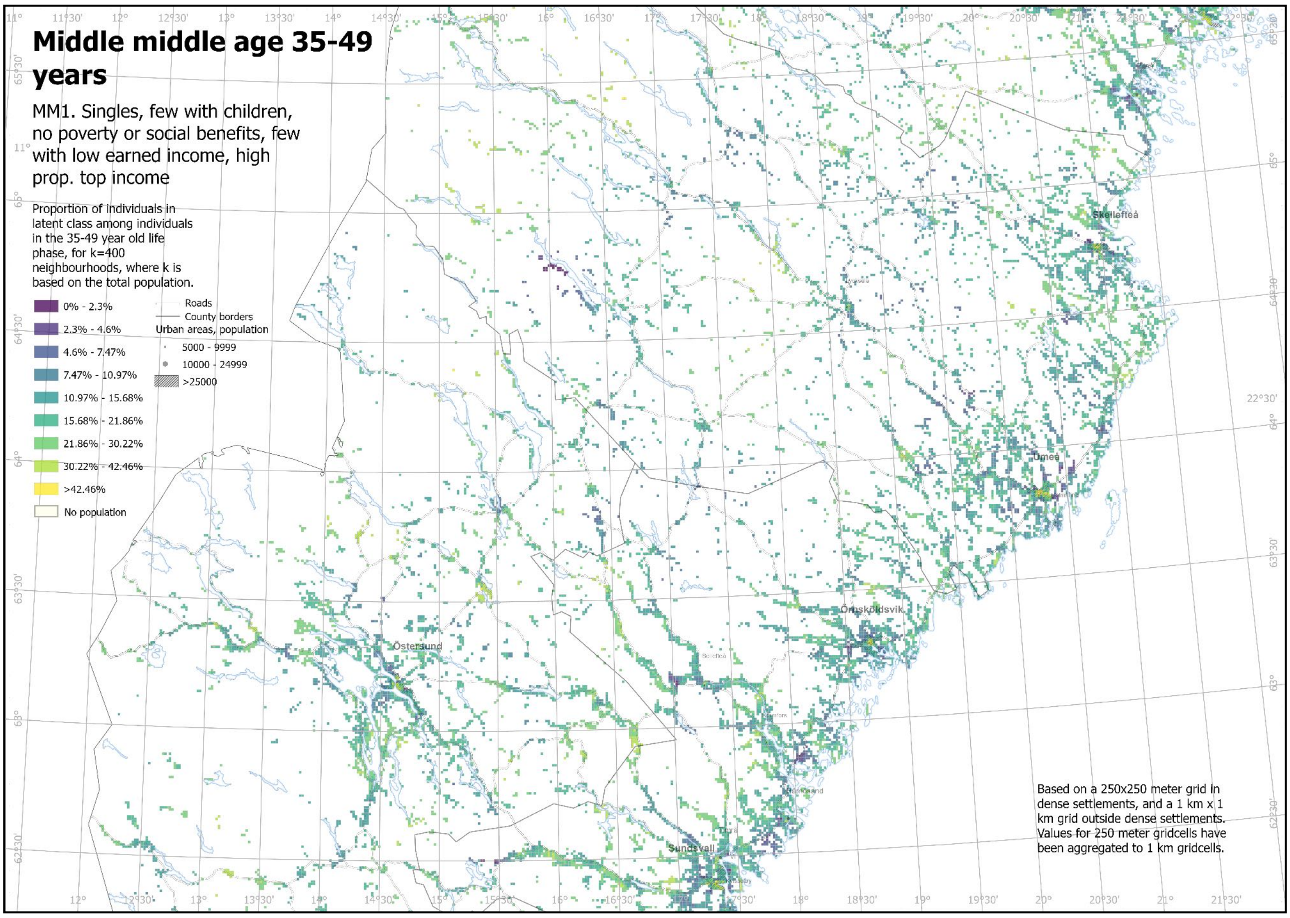

Supplementary Figure S 15 Spatial distribution of middle income singles life course MM1

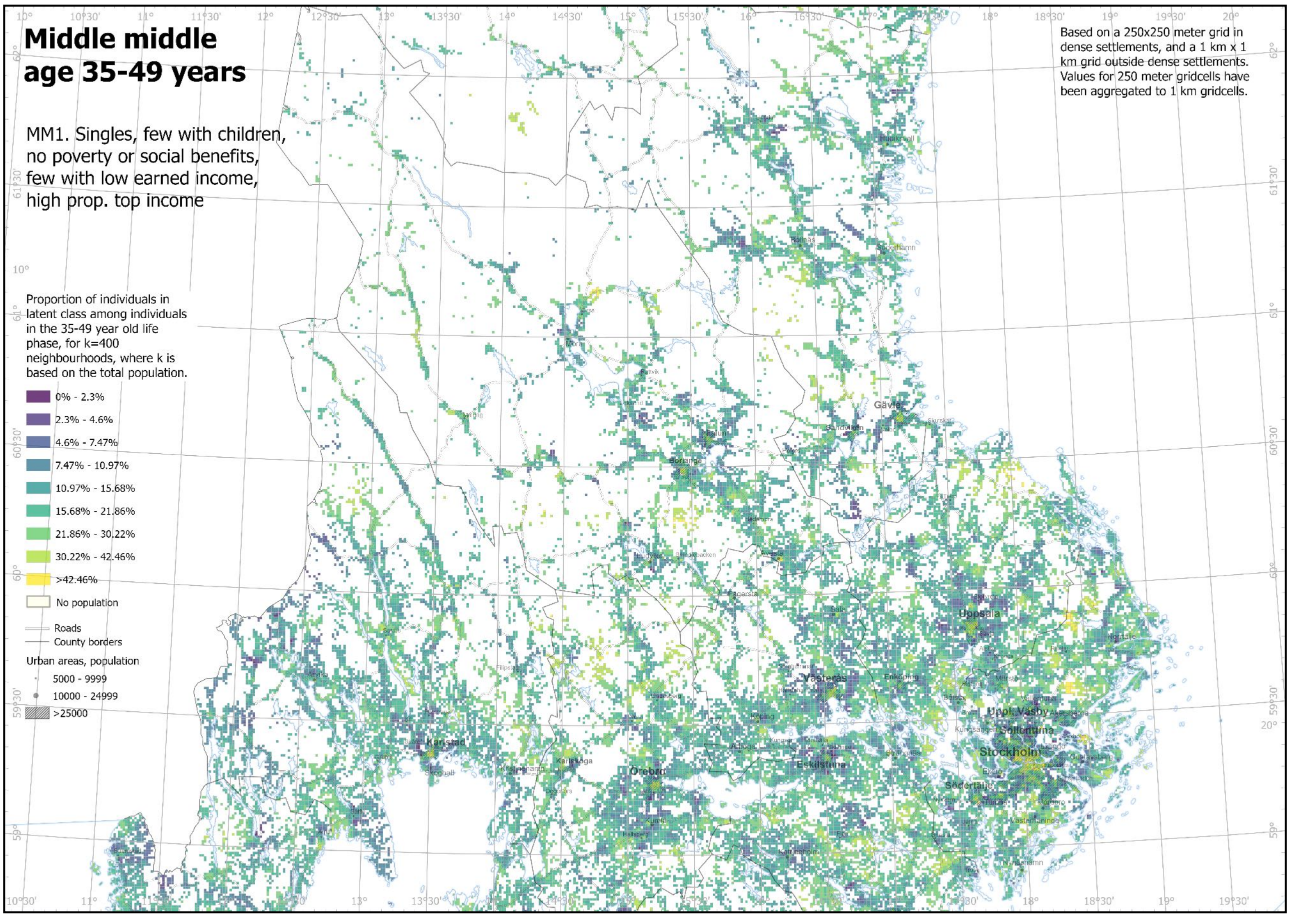

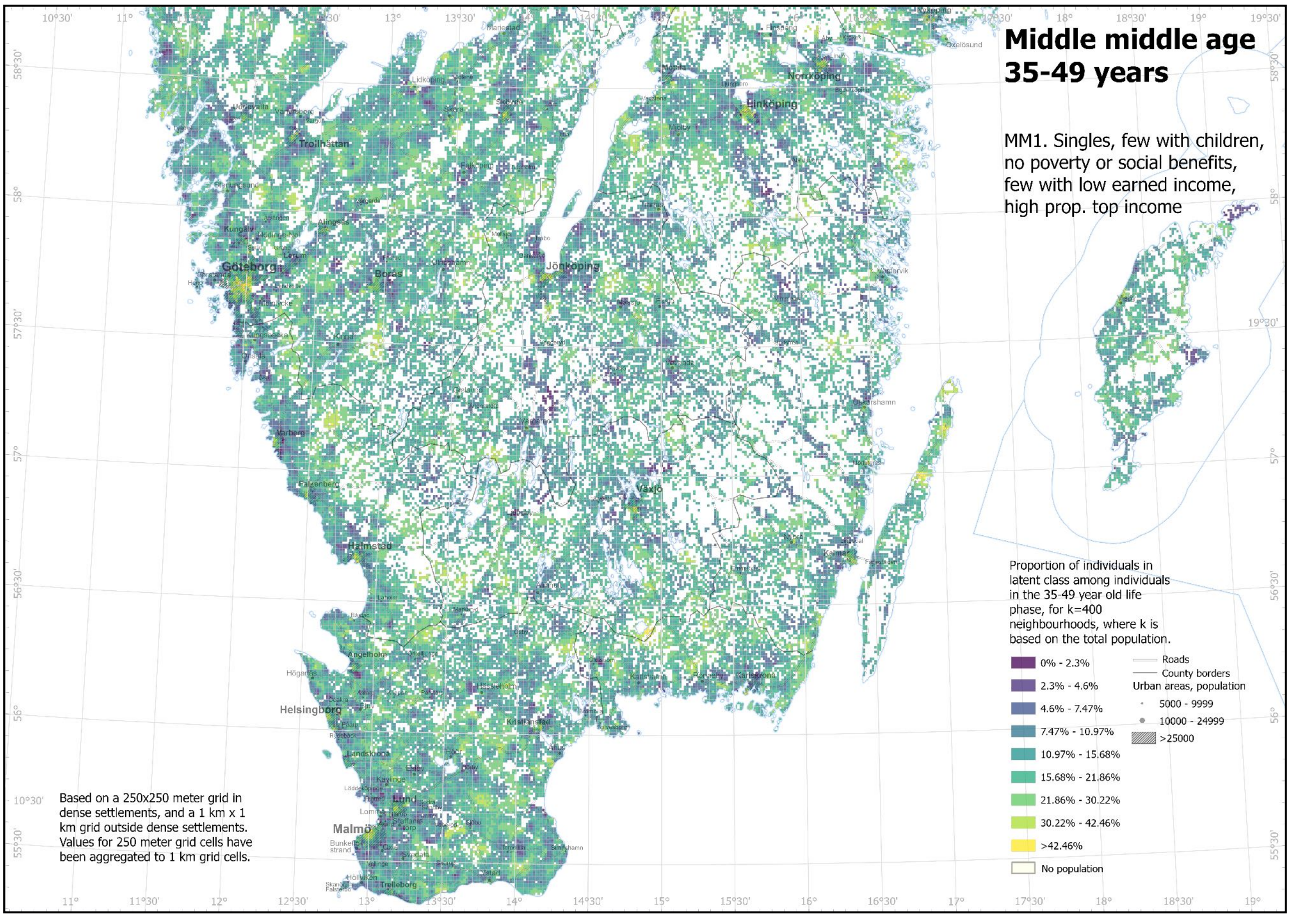

Supplementary Figure S 15 Spatial distribution of middle income singles life course MM1, continued

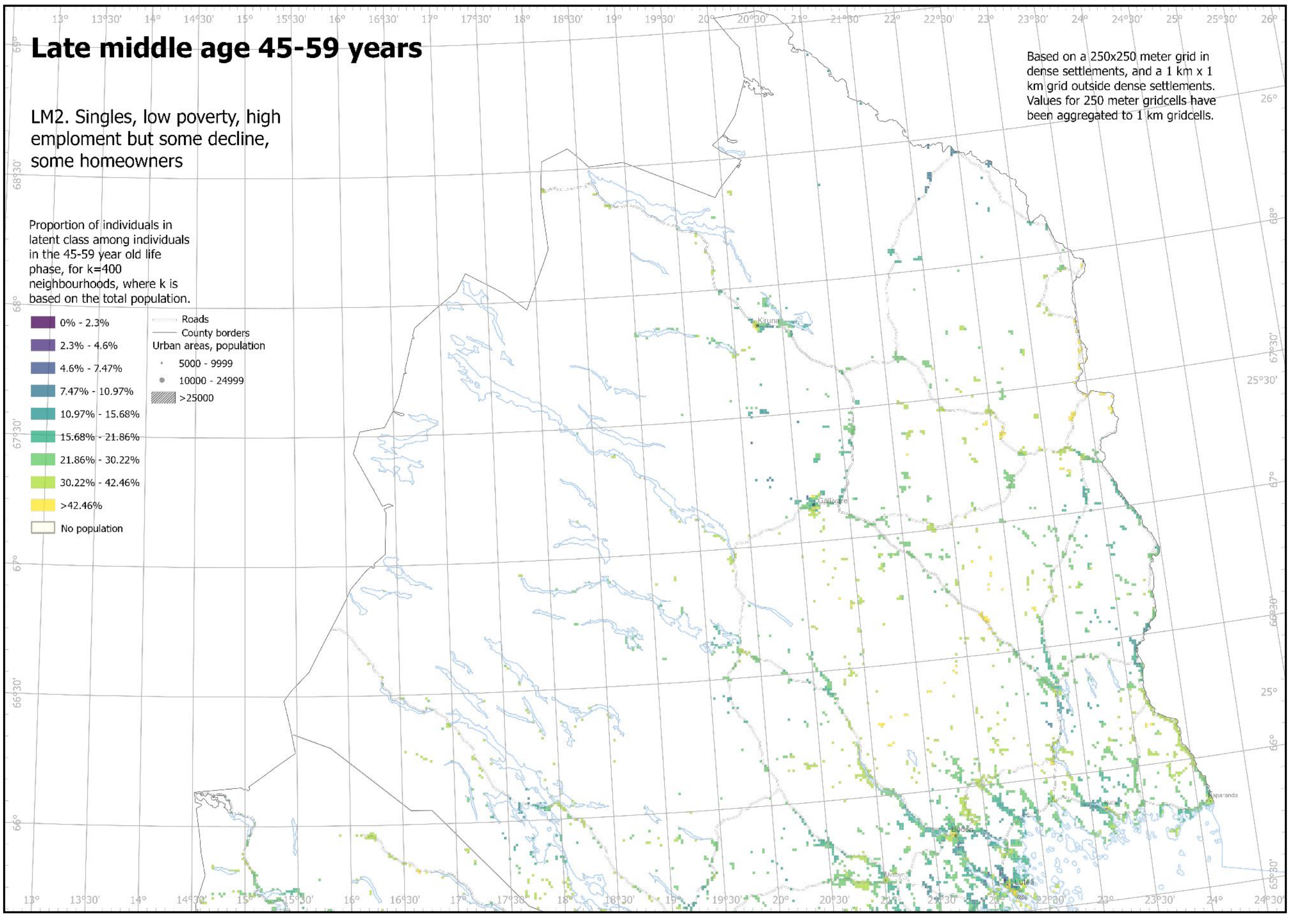

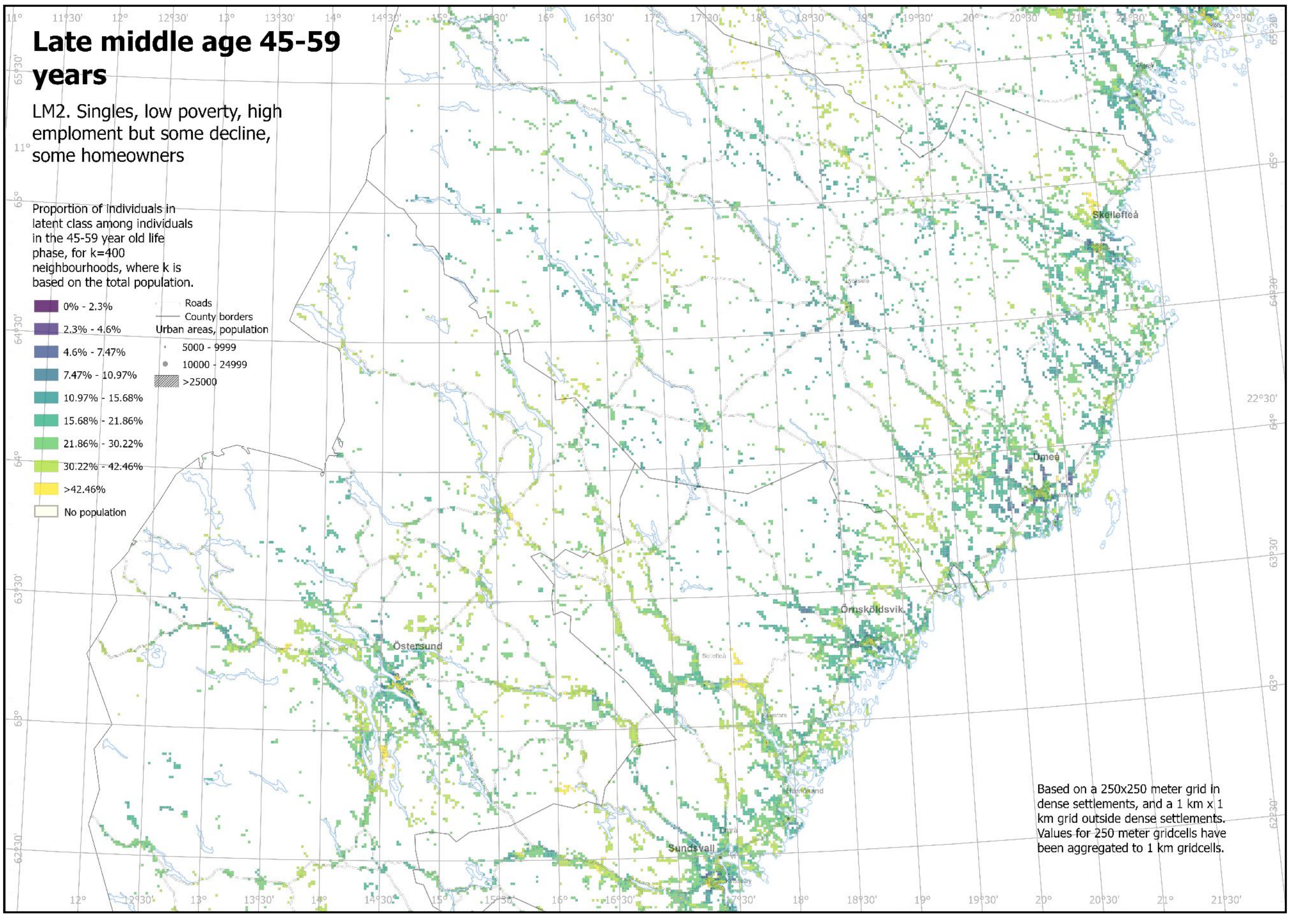

Supplementary Figure S 16 Spatial distribution of middle income singles life course LM2

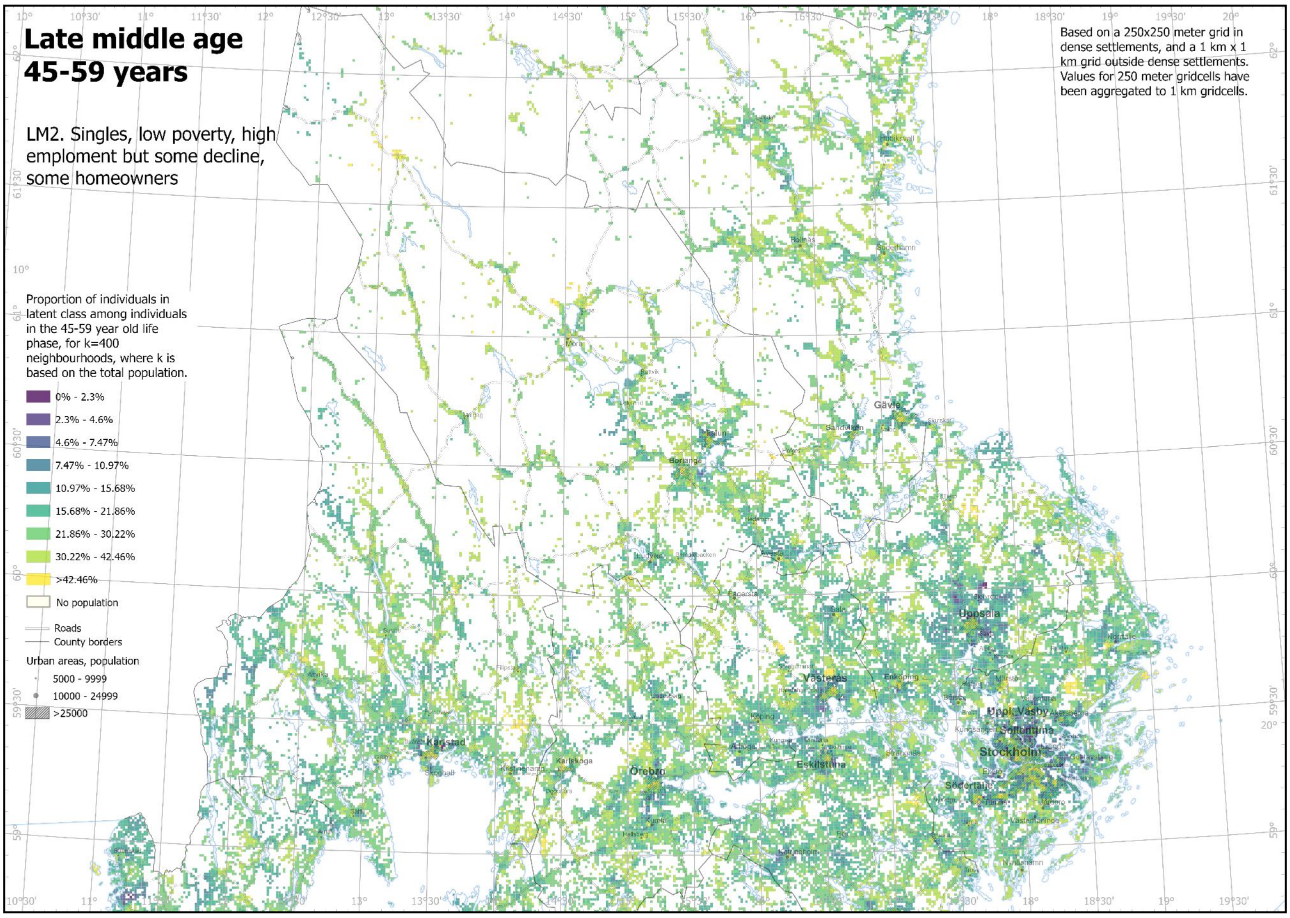

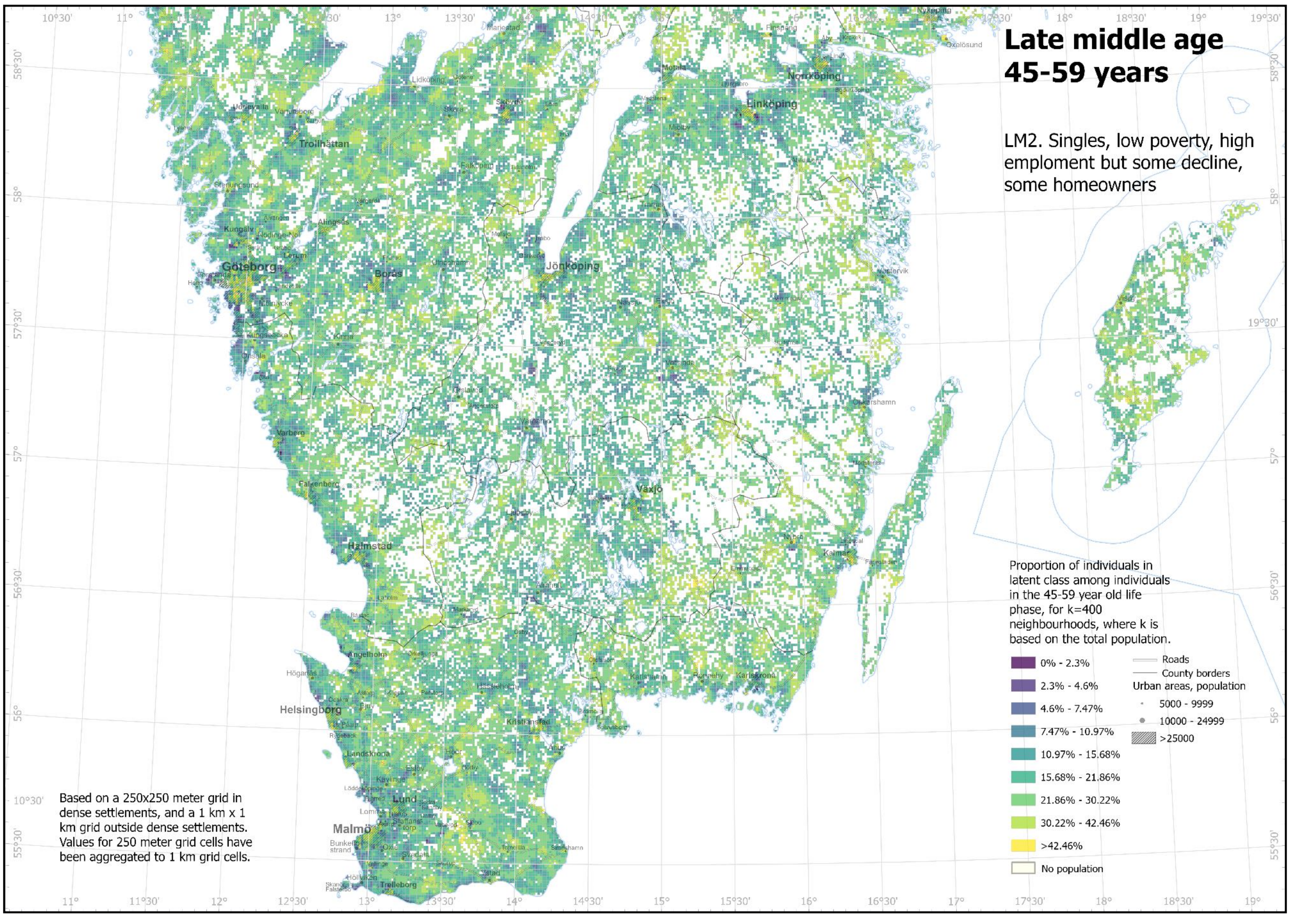

Supplementary Figure S 16 Spatial distribution of middle income singles life course LM2, continued

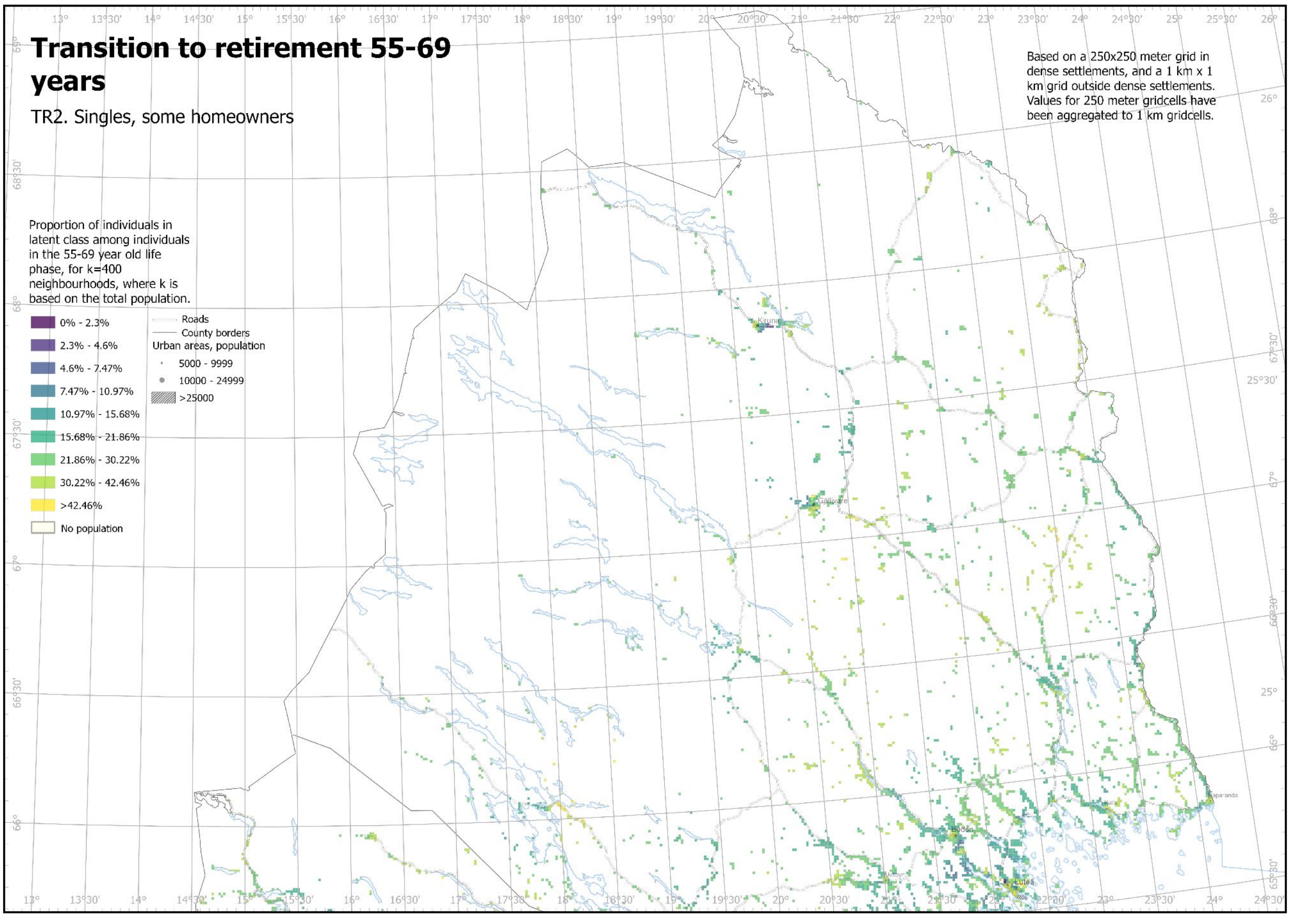

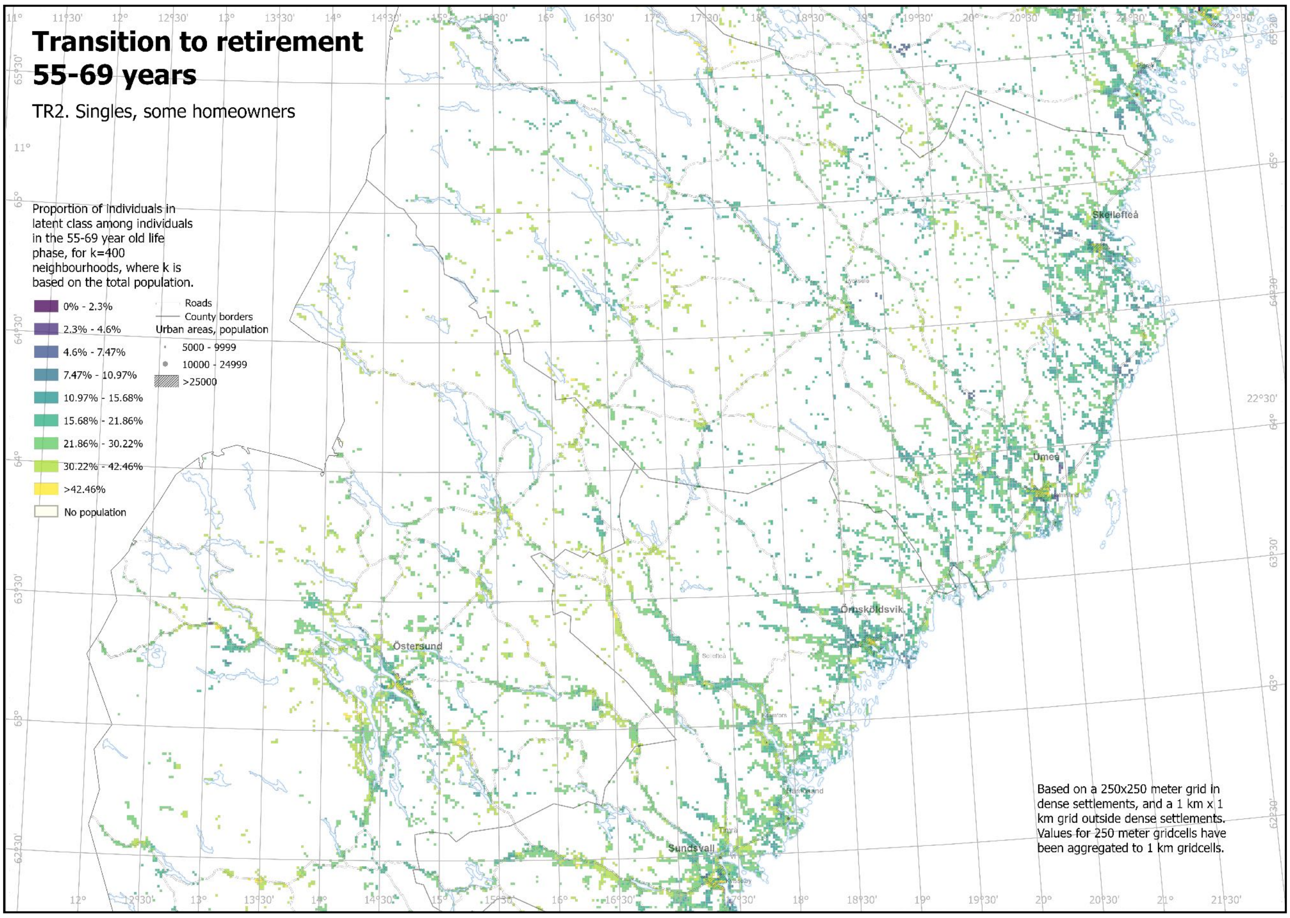

Supplementary Figure S 17 Spatial distribution of middle income singles life course TR2

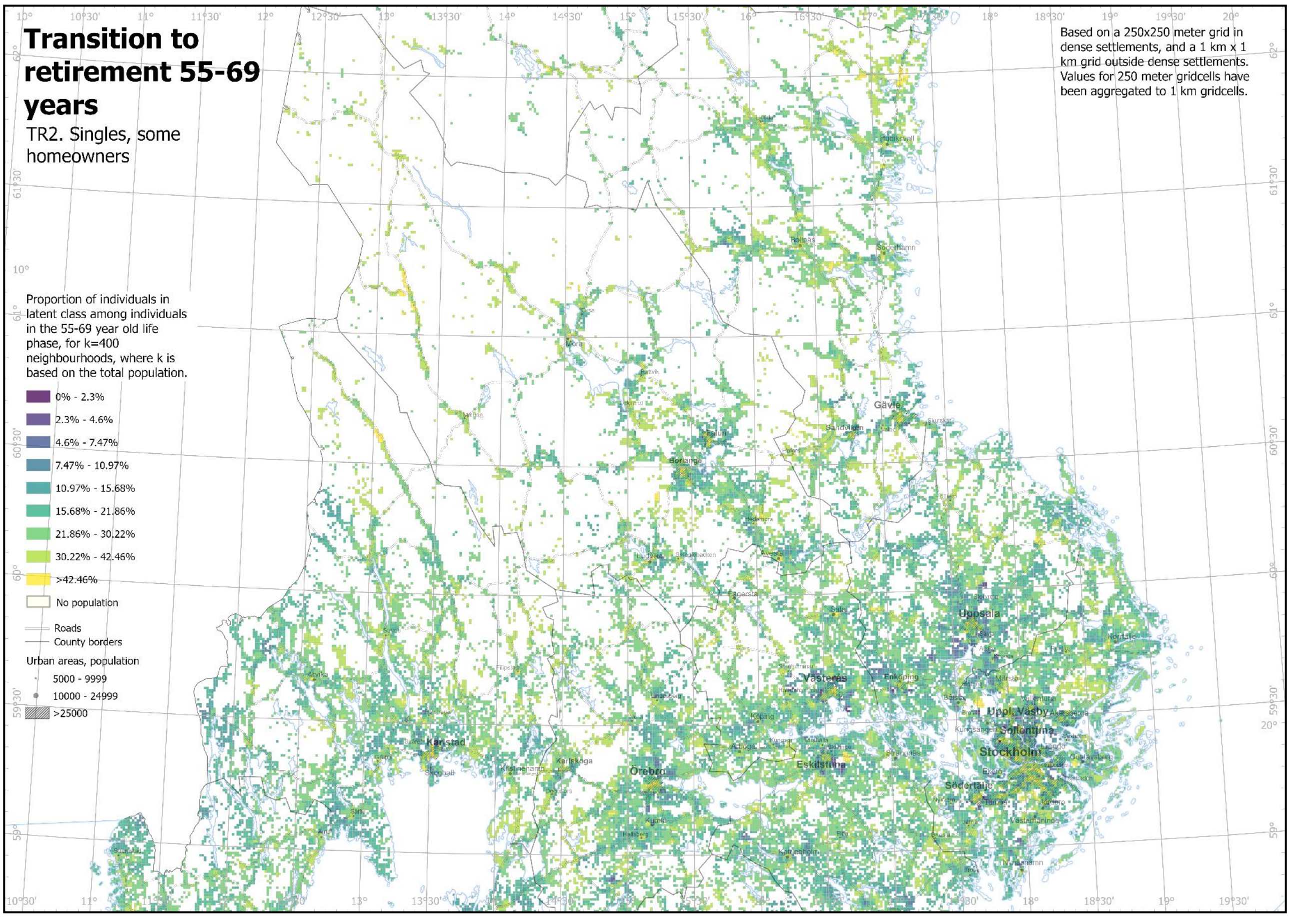

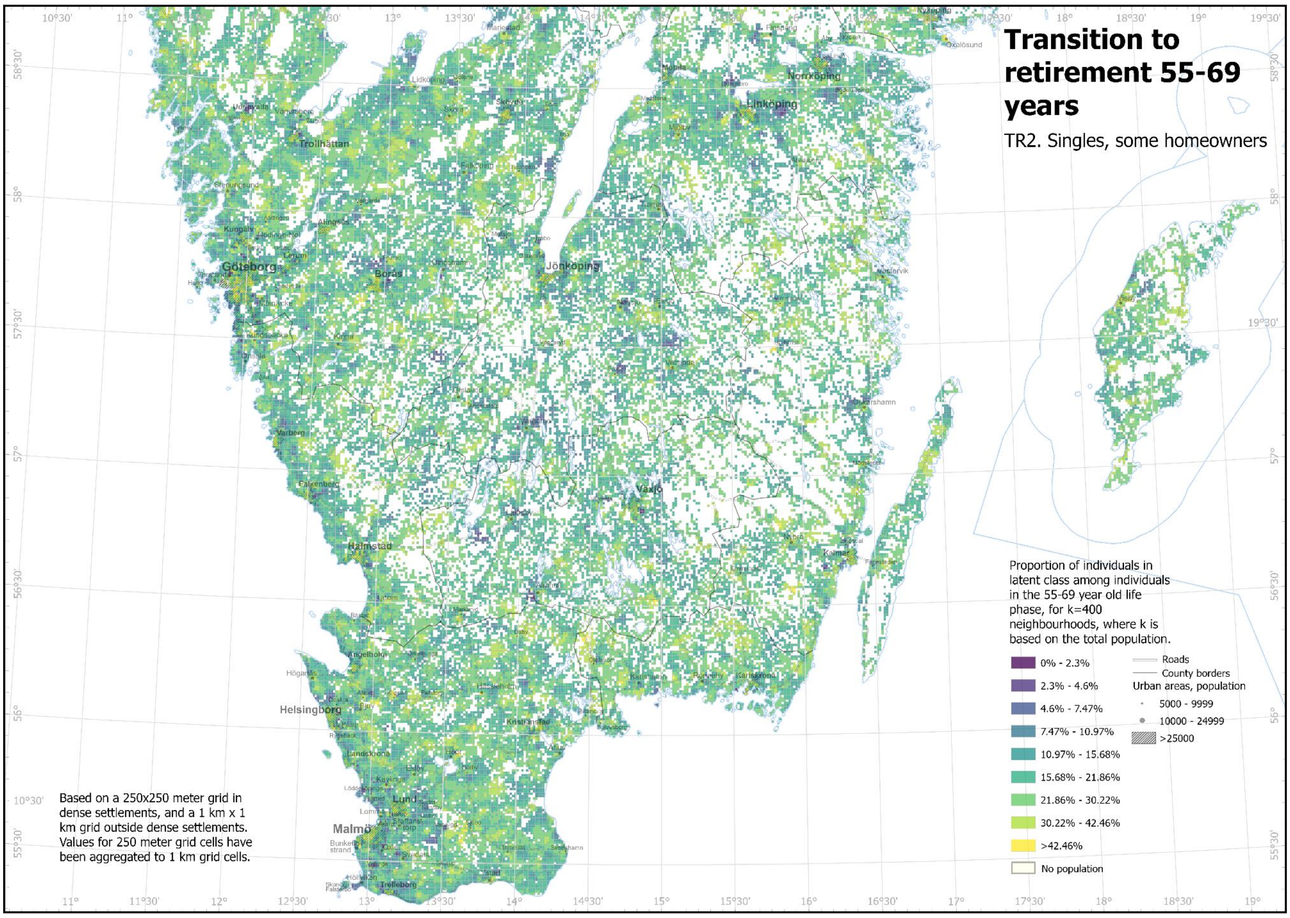

Supplementary Figure S 17 Spatial distribution of middle income singles life course TR2, continued

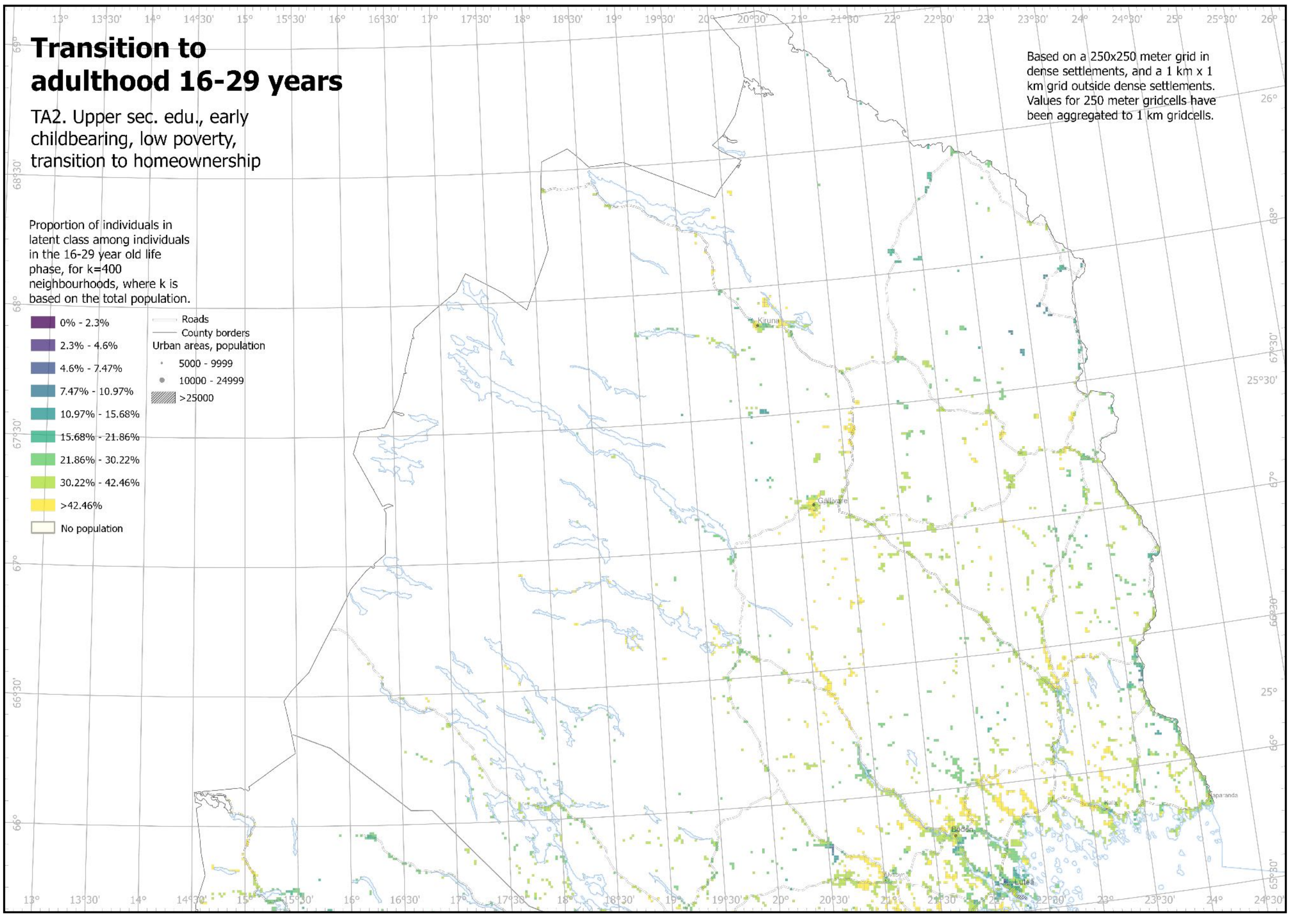

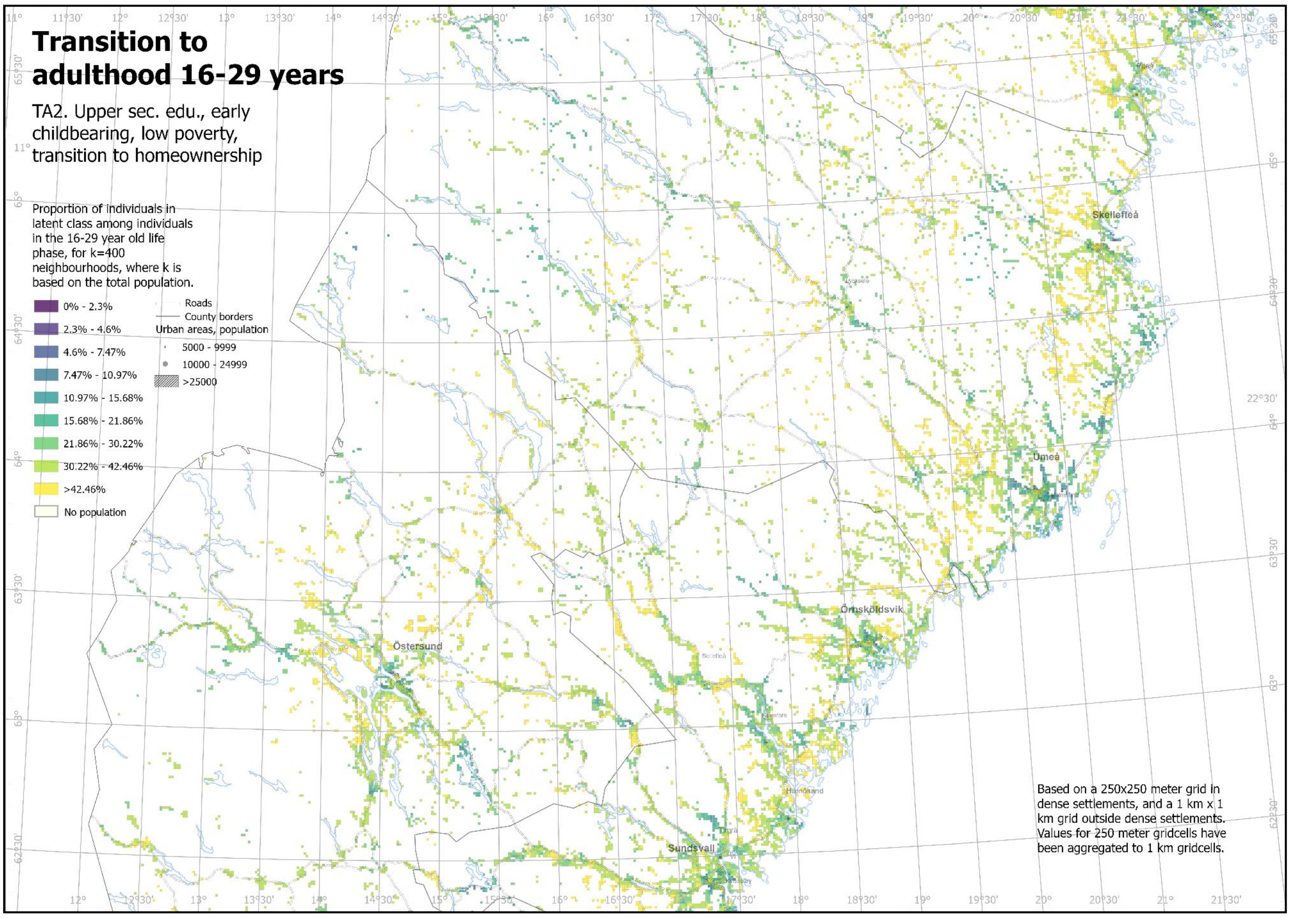

Supplementary Figure S 18 Spatial distribution of middle income non homeowner life course TA2

Middle income non homeowner

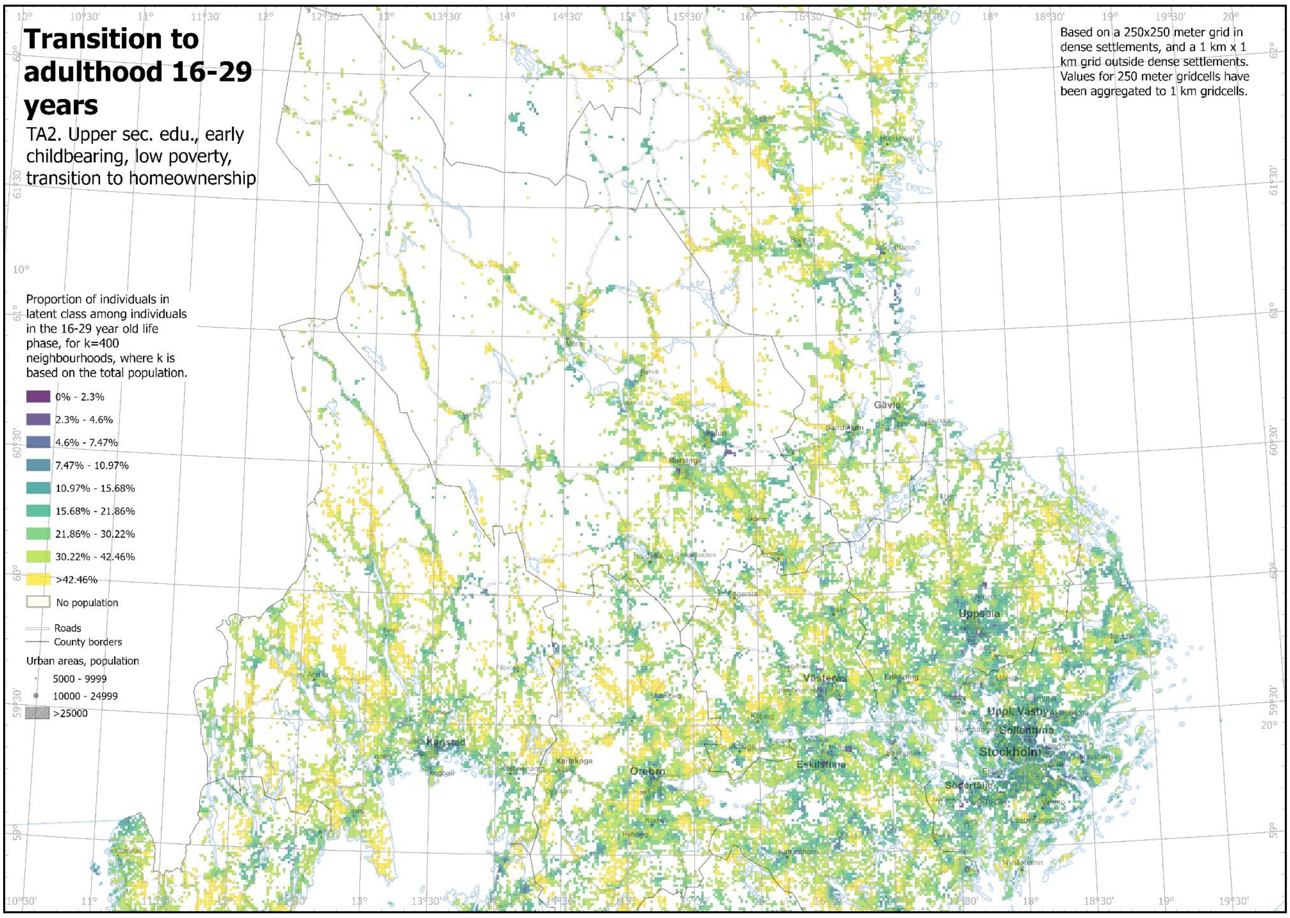

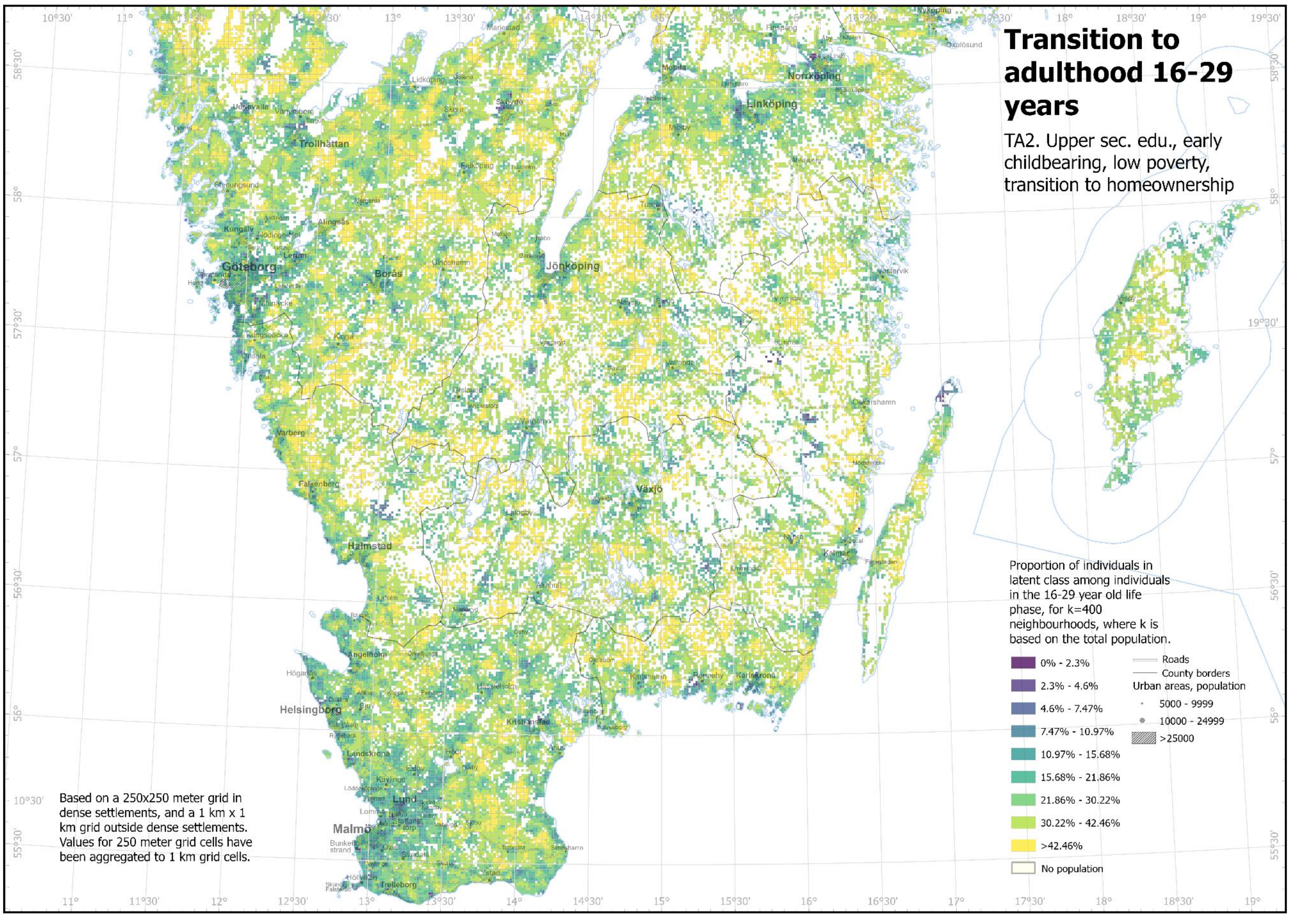

Supplementary Figure S 18 Spatial distribution of middle income non homeowner life course TA2, continued

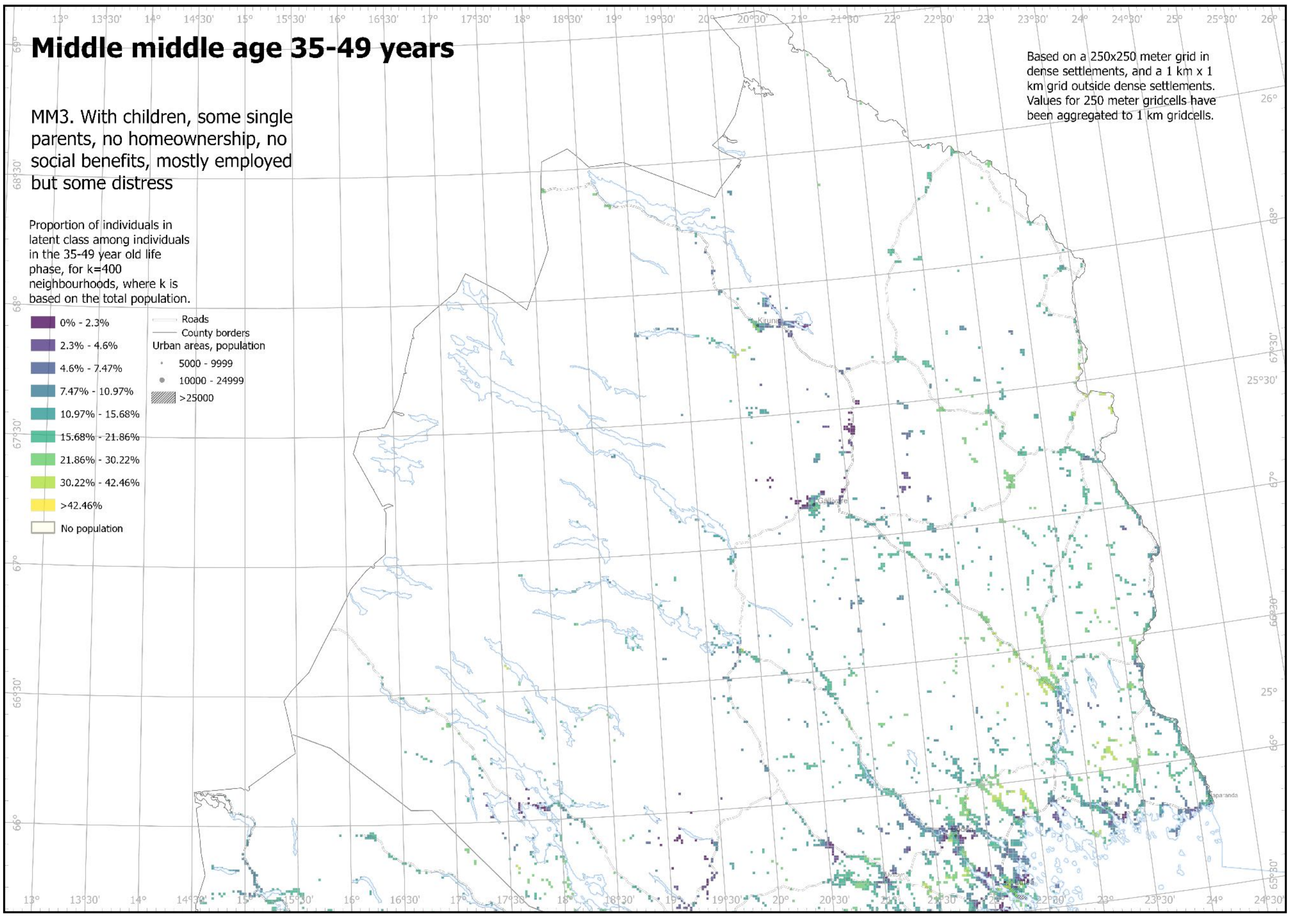

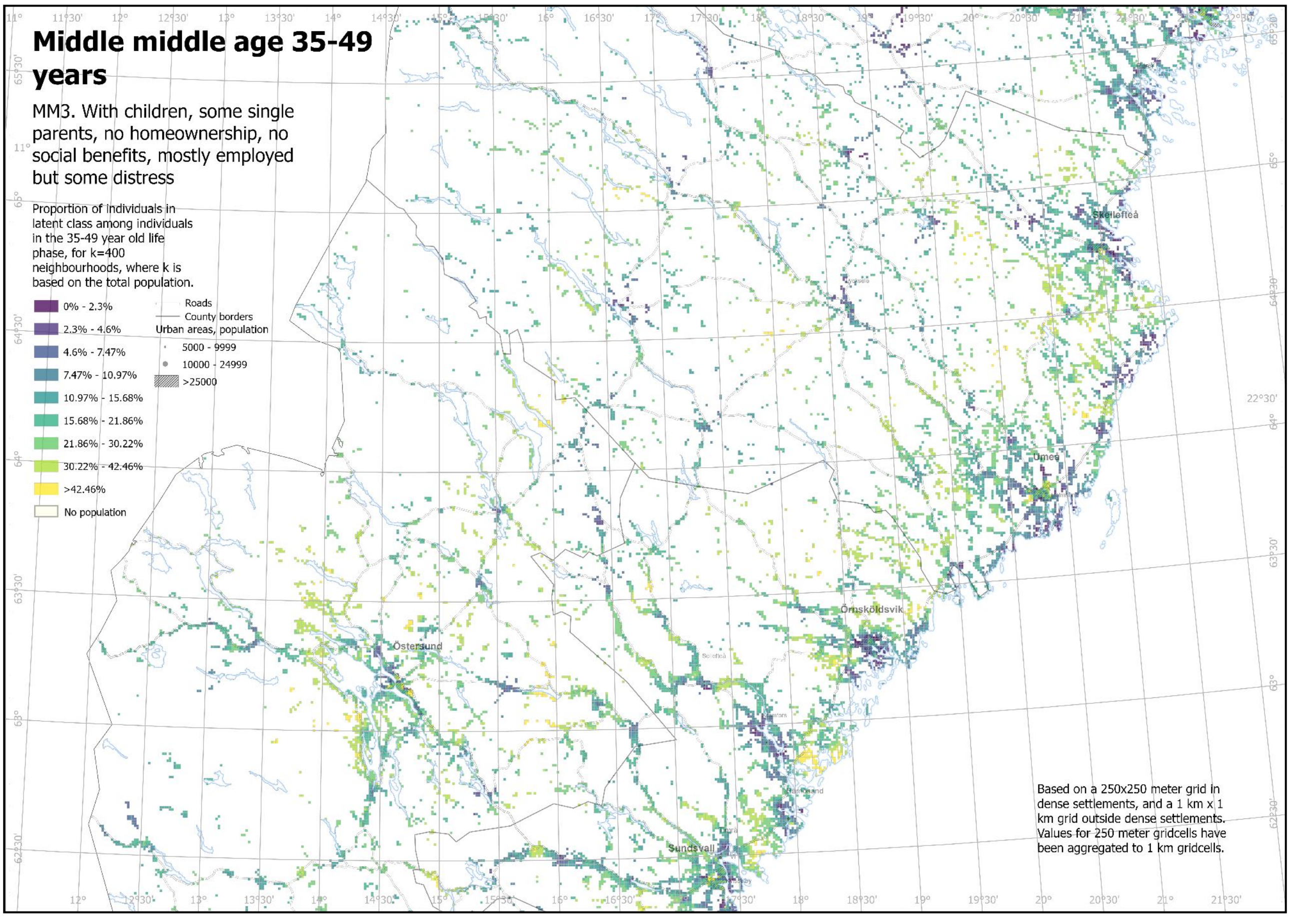

Supplementary Figure S 19 Spatial distribution of middle income non homeowner life course MM3, continued

Middle income non homeowner

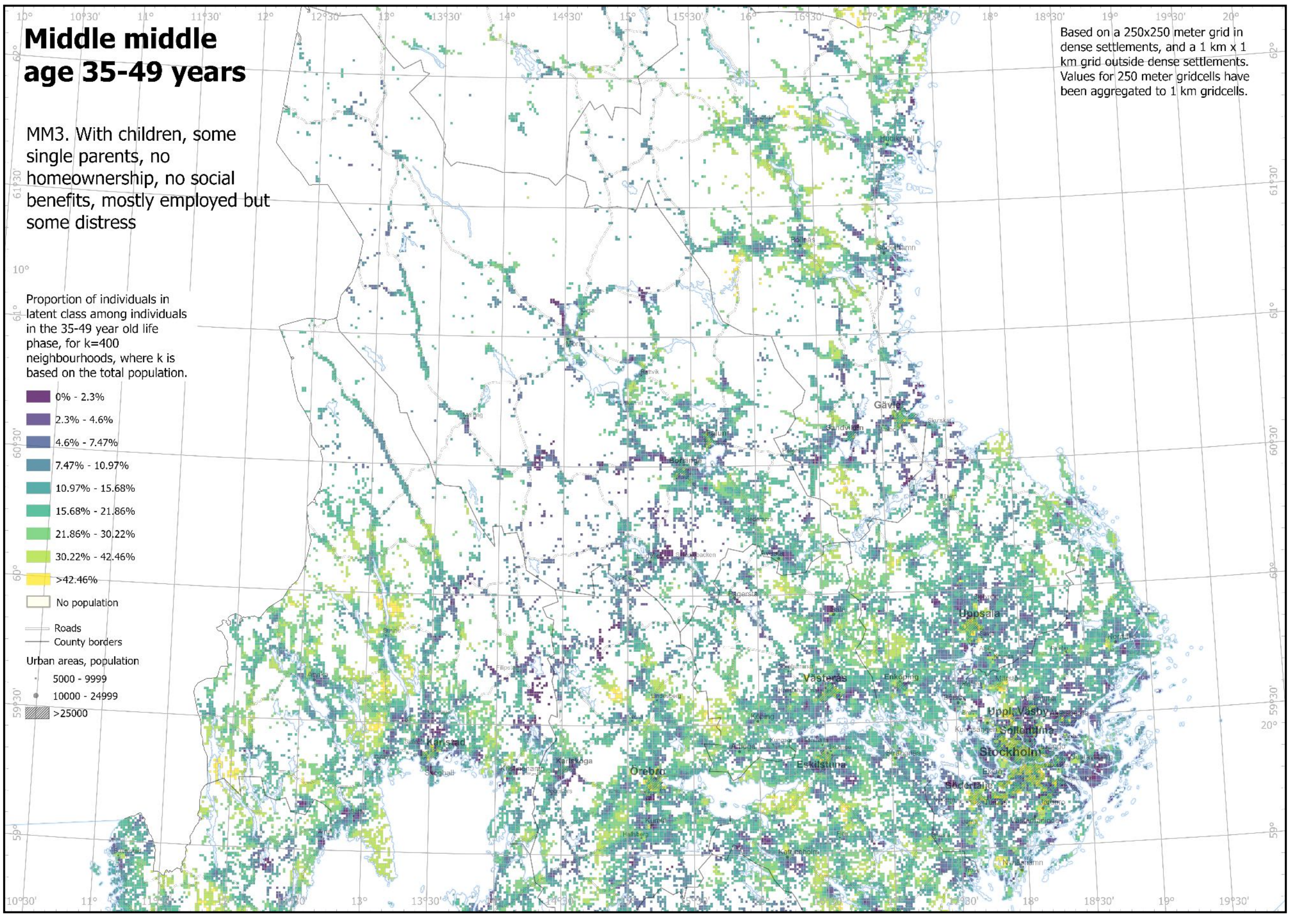

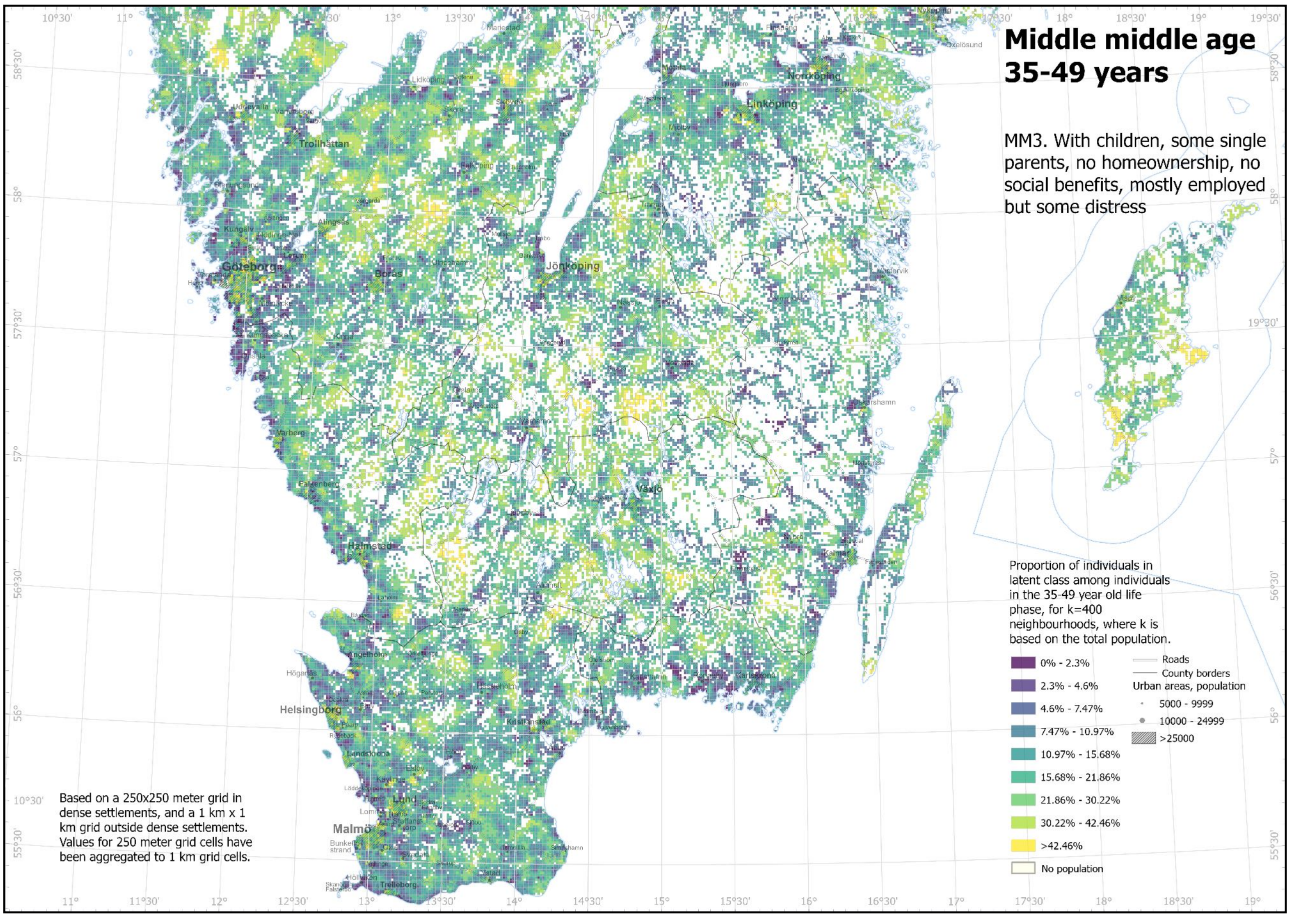

Supplementary Figure S 20 Spatial distribution of middle income non homeowner life course MM3, continued

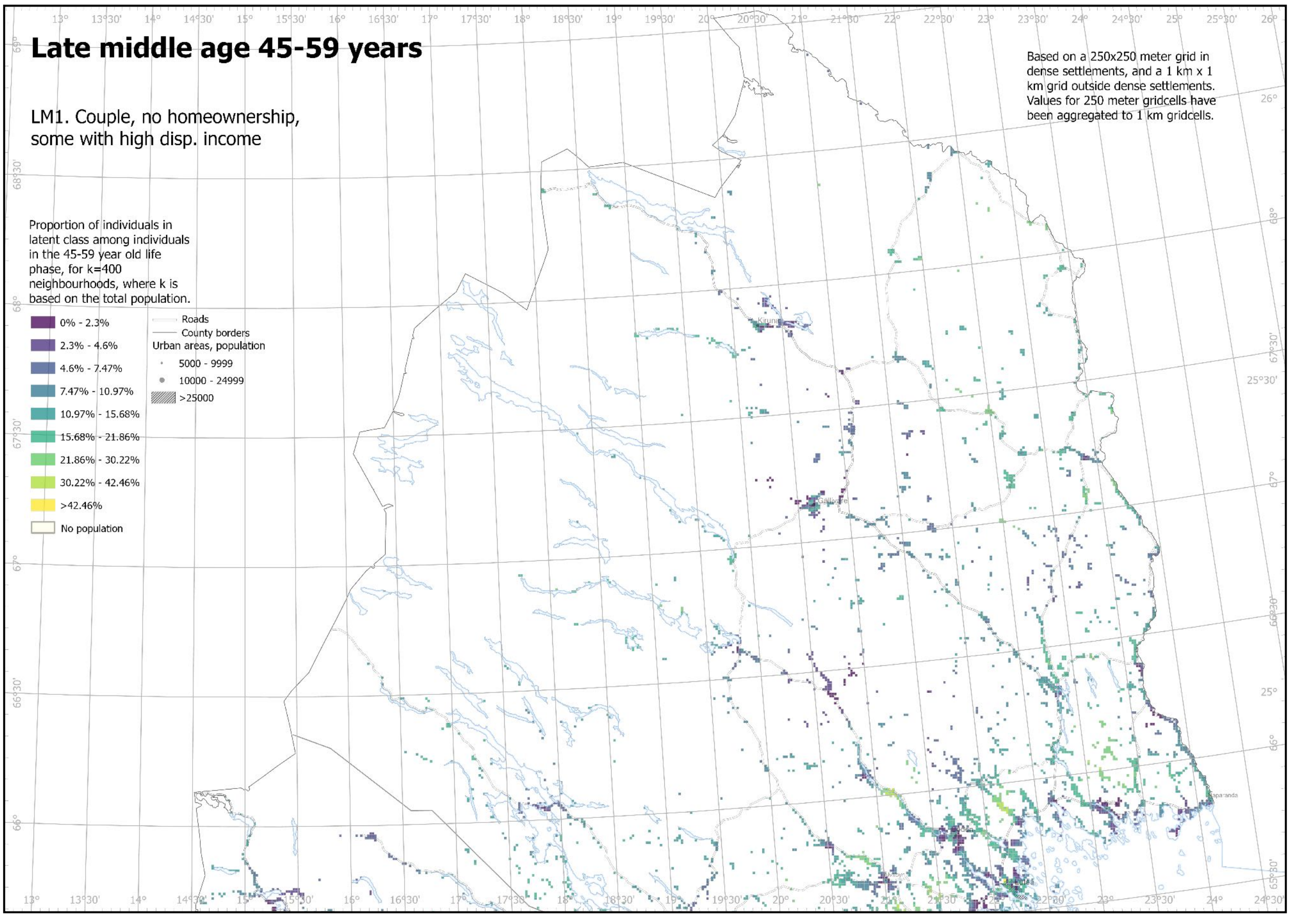

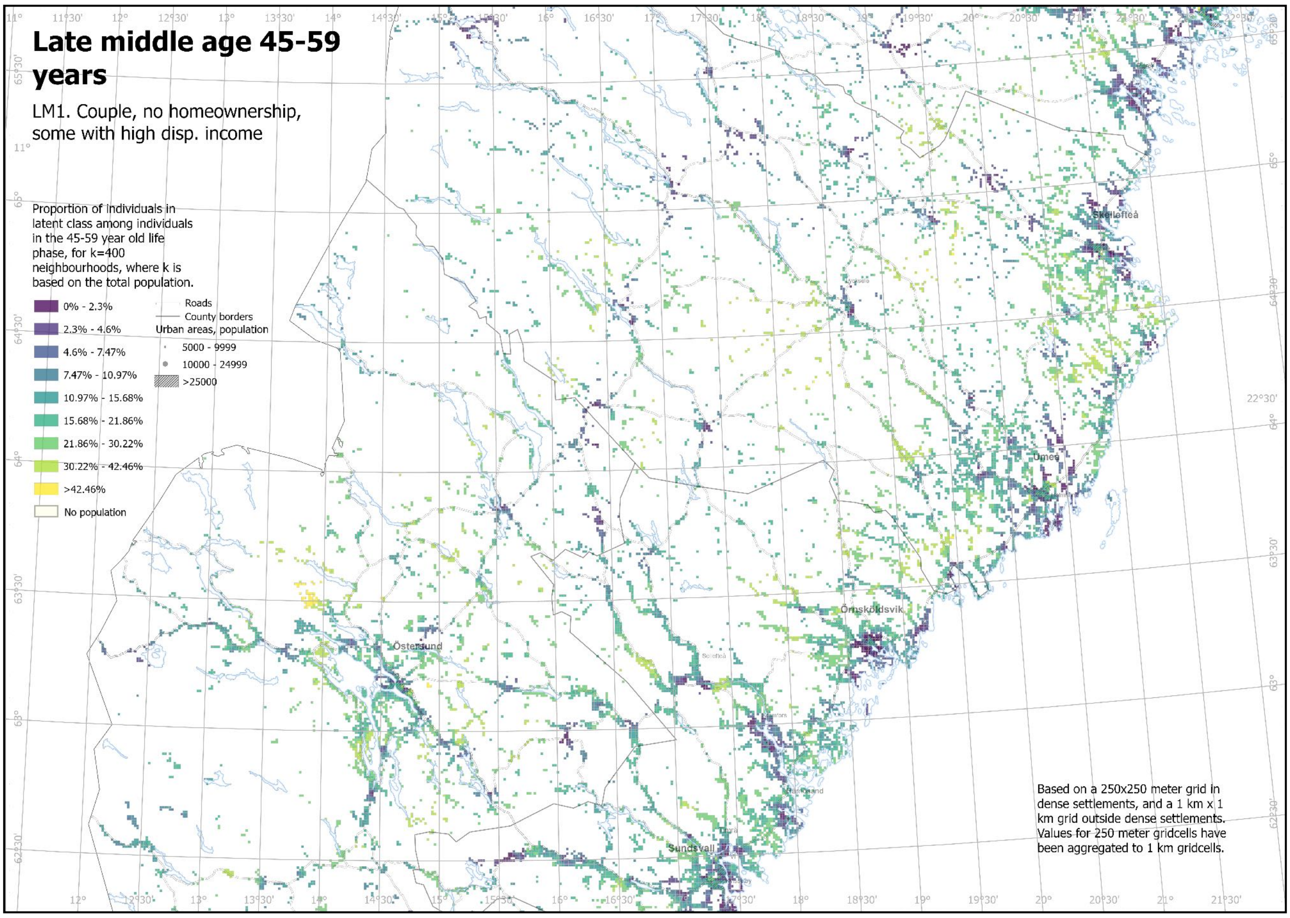

Supplementary Figure S 20 Spatial distribution of middle income non homeowner life course LM1

Middle income non homeowner

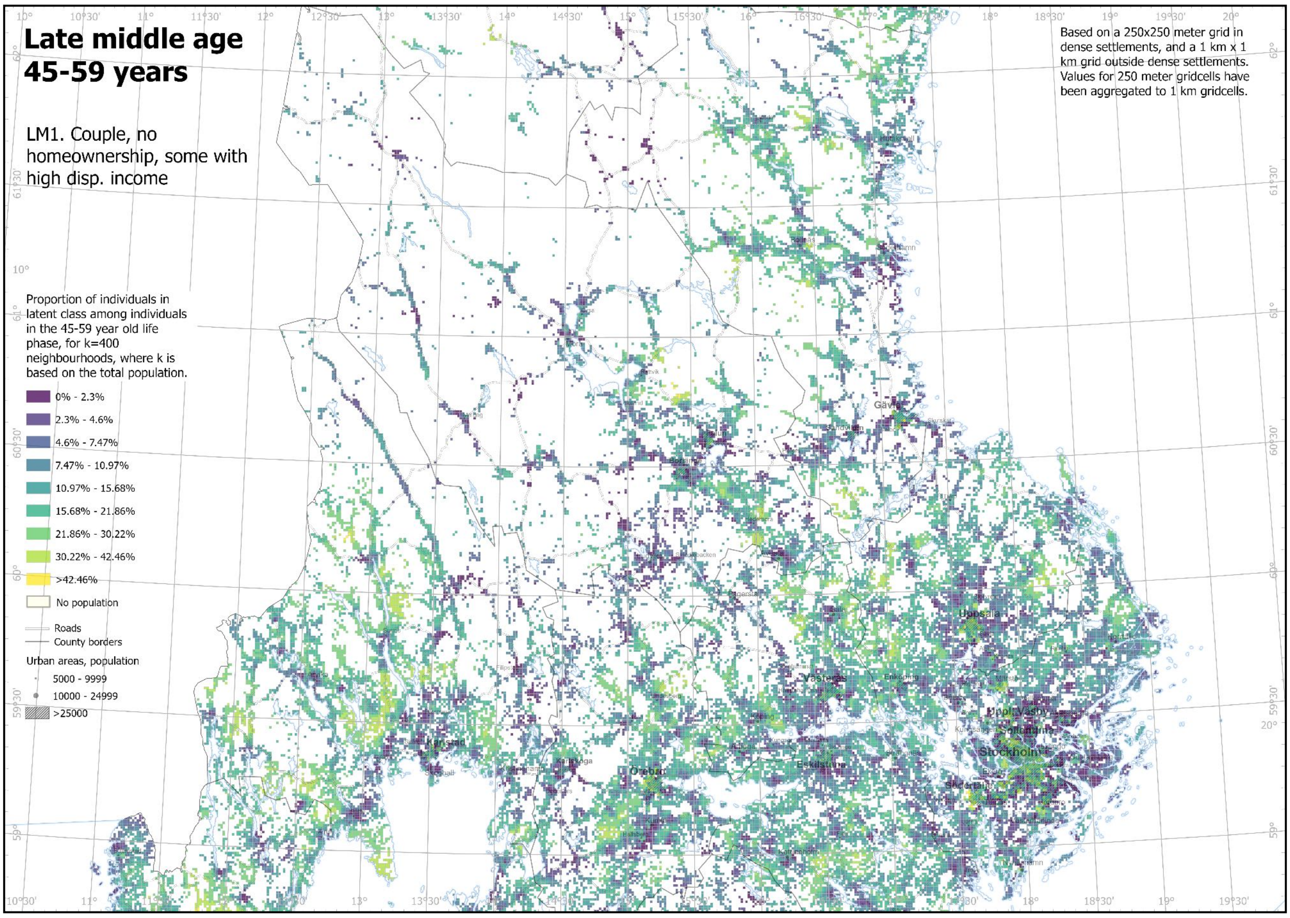

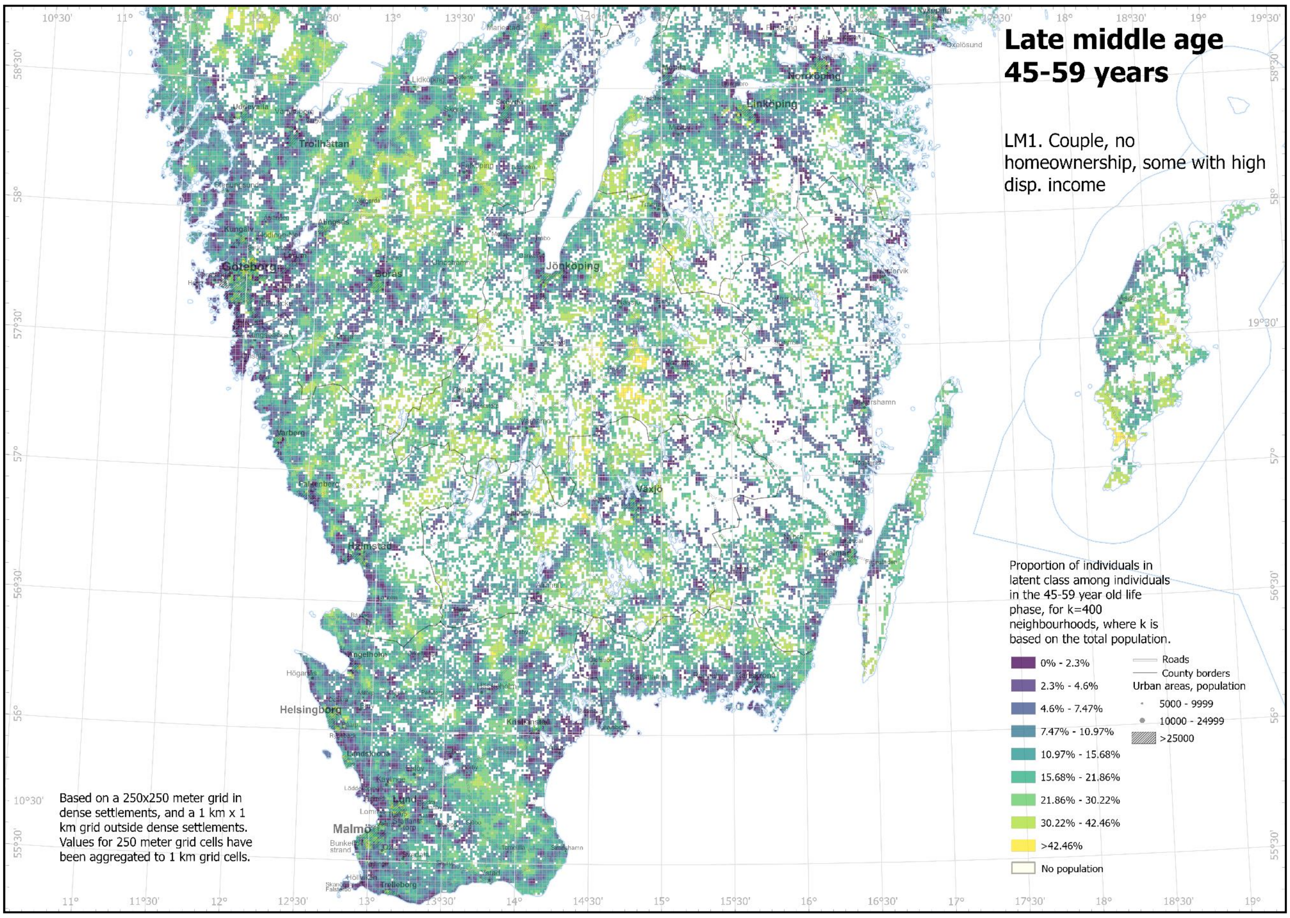

Supplementary Figure S 20 Spatial distribution of middle income non homeowner life course LM1, continued

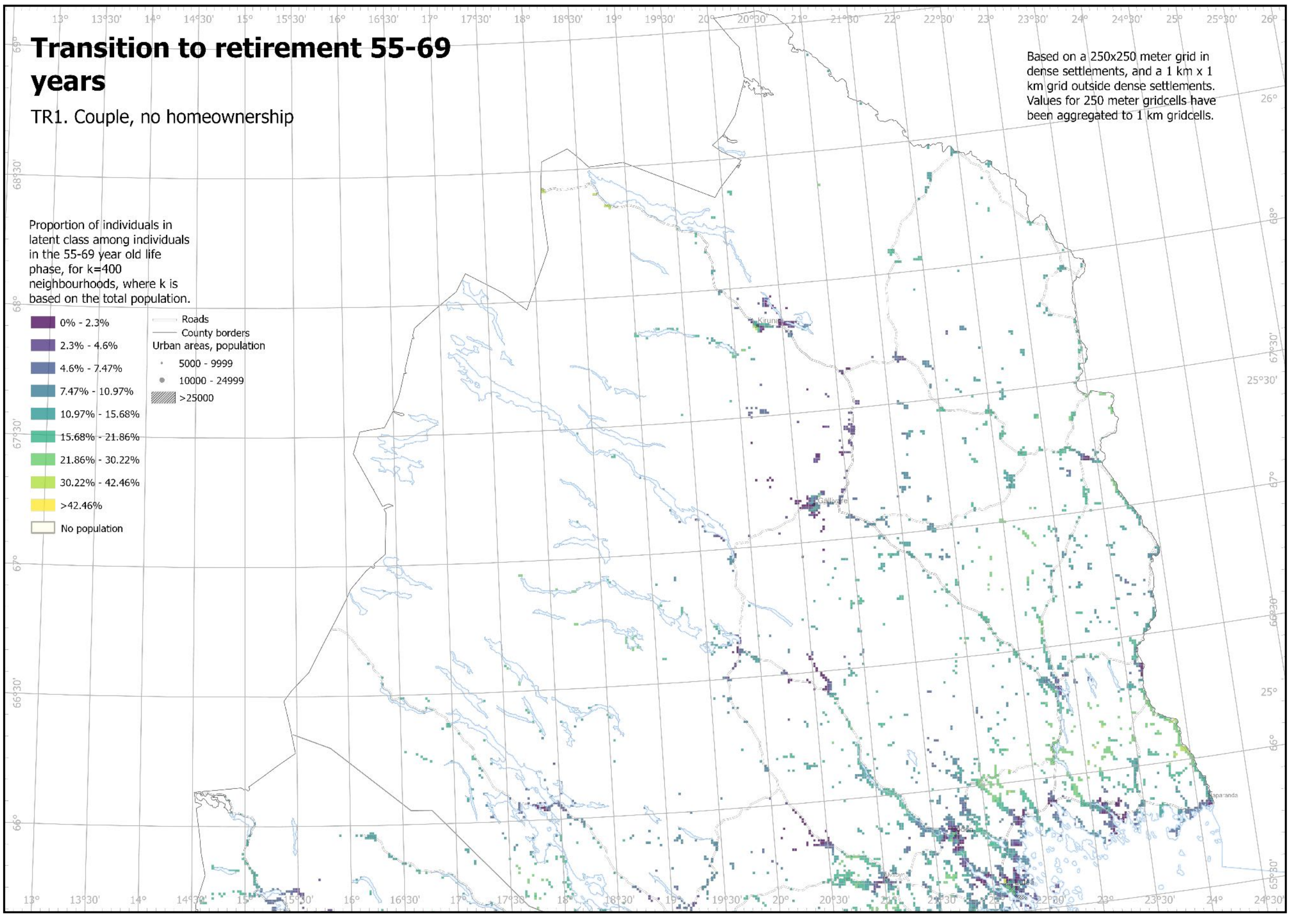

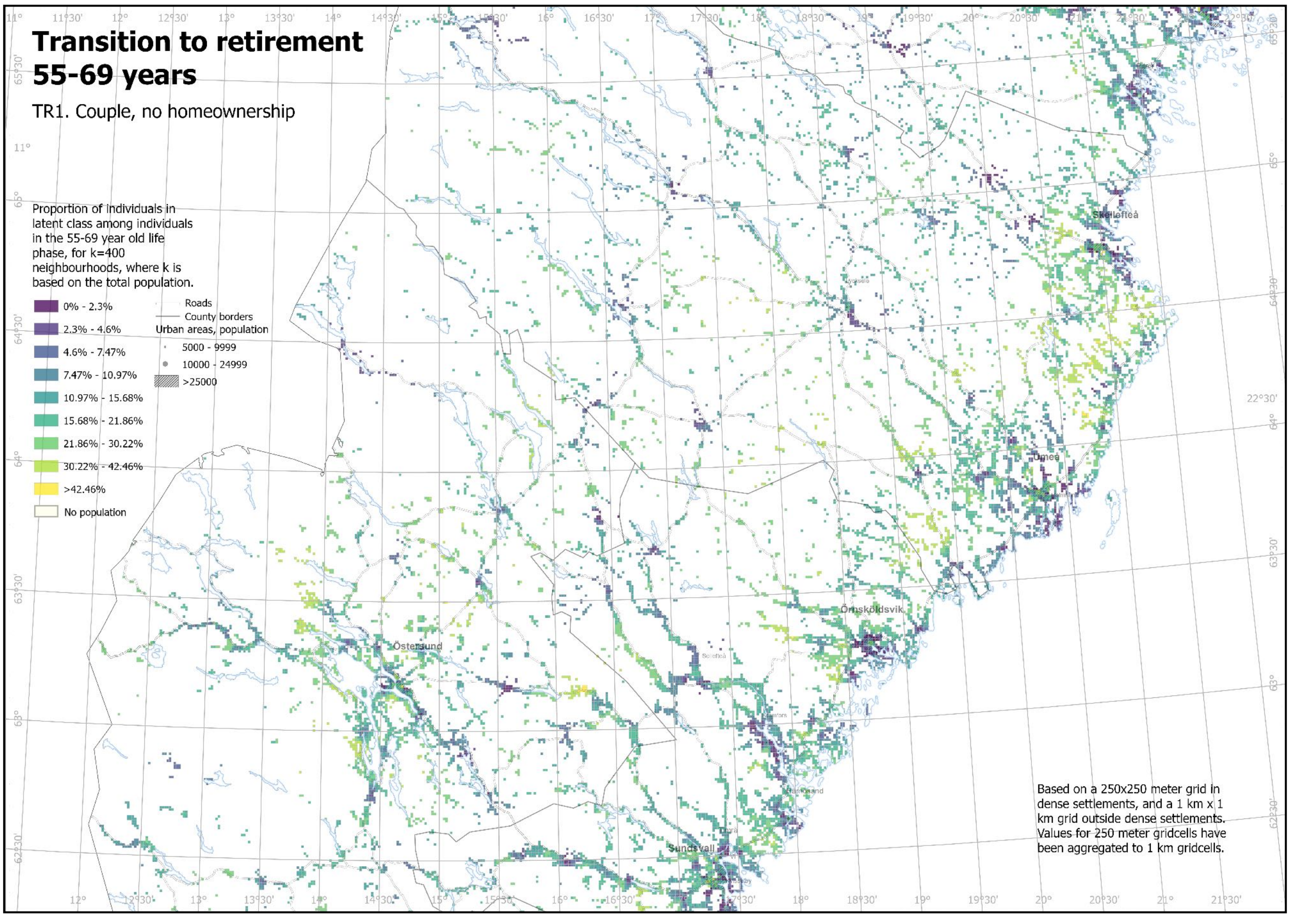

Supplementary Figure S 21 Spatial distribution of middle income non homeowner life course TR1

Middle income non homeowner

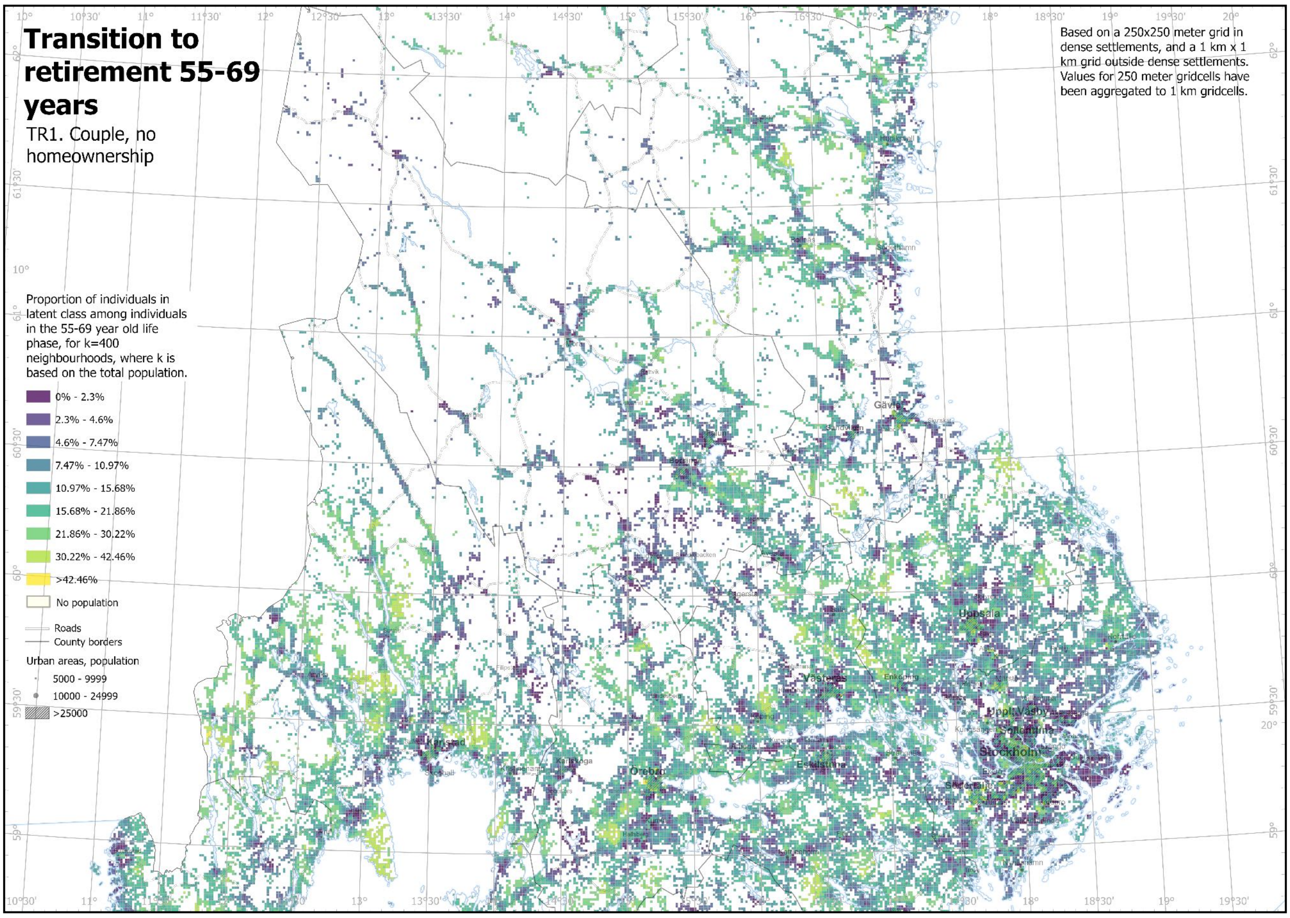

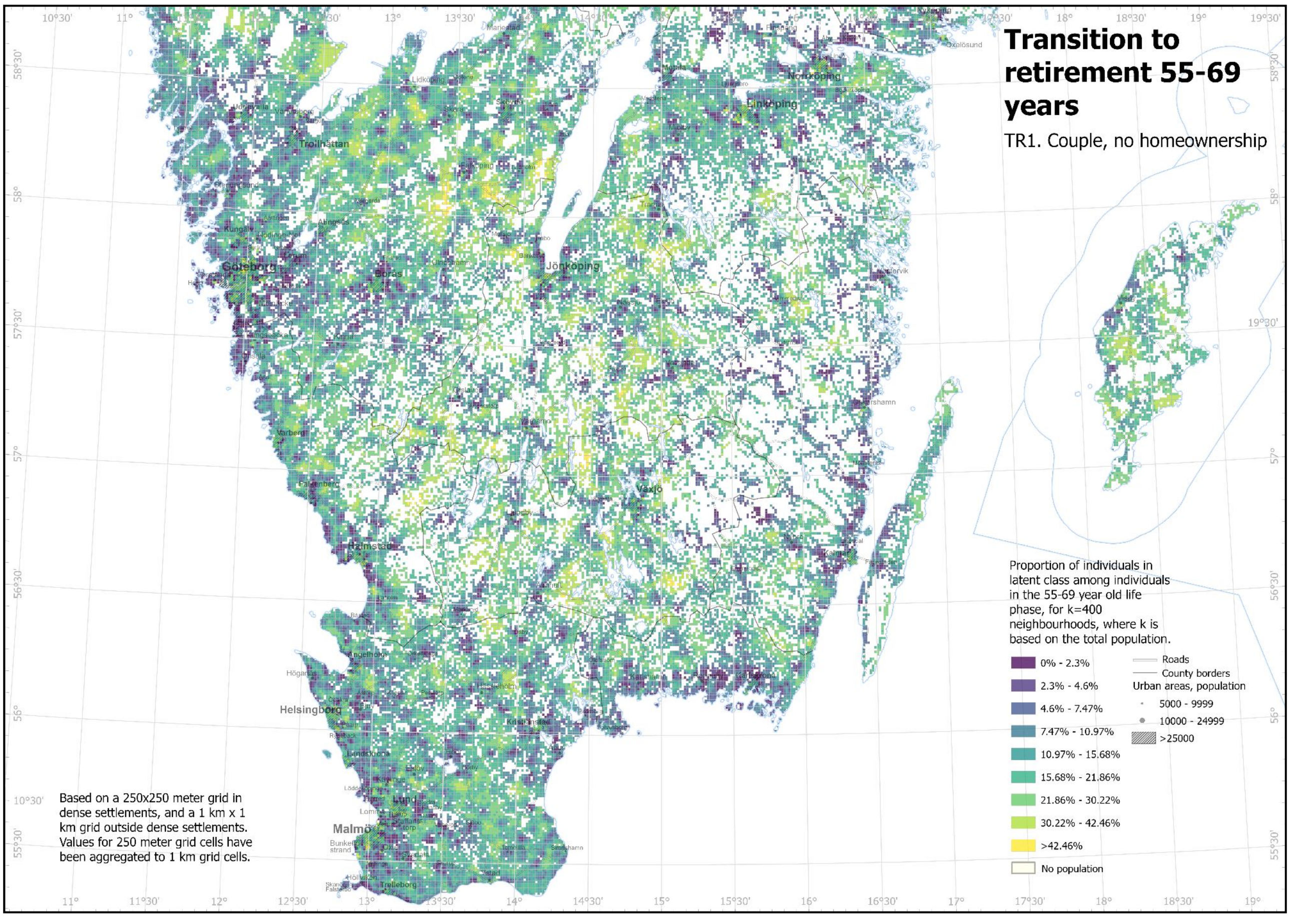

Supplementary Figure S 21 Spatial distribution of middle income non homeowner life course TR1, continued

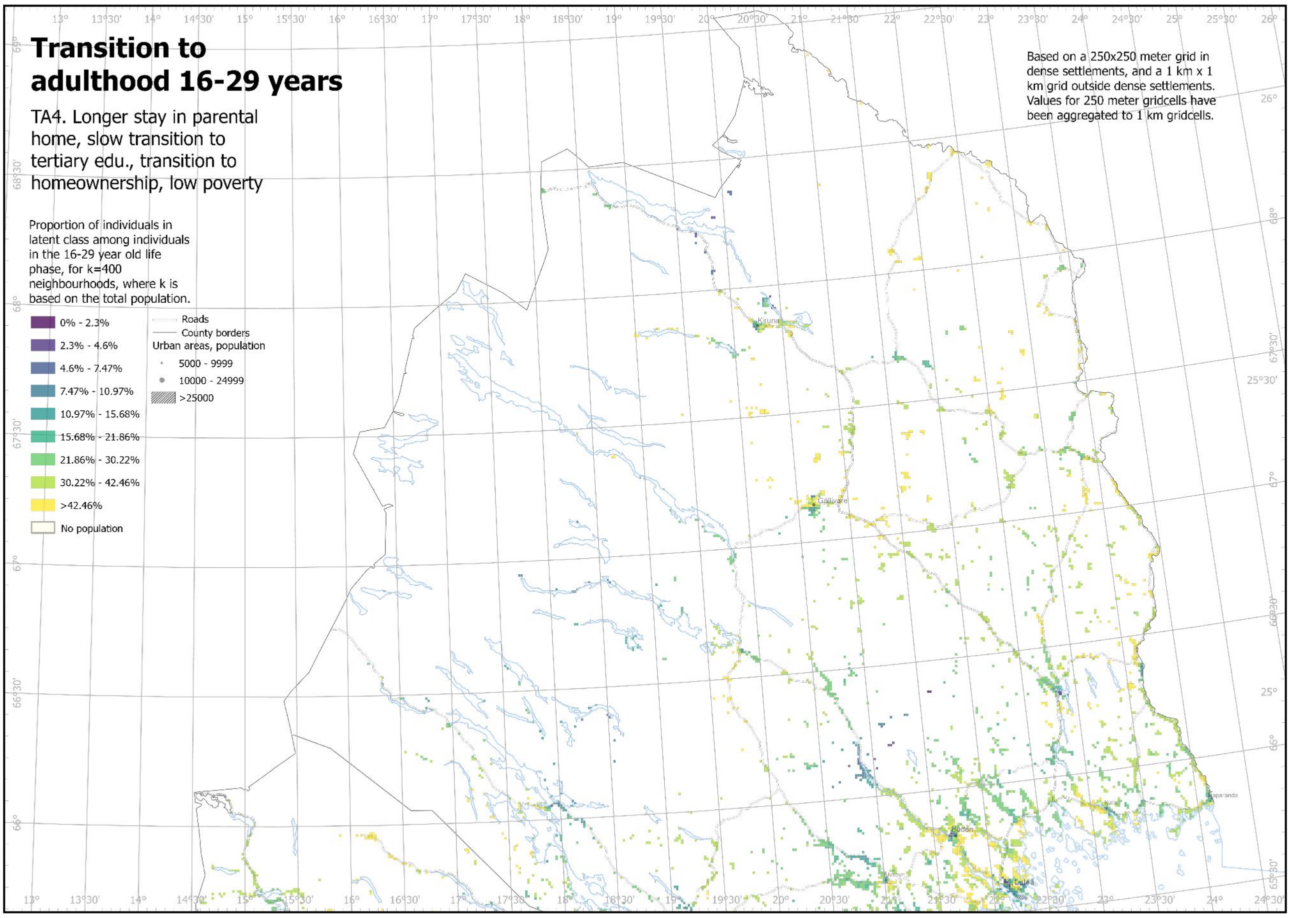

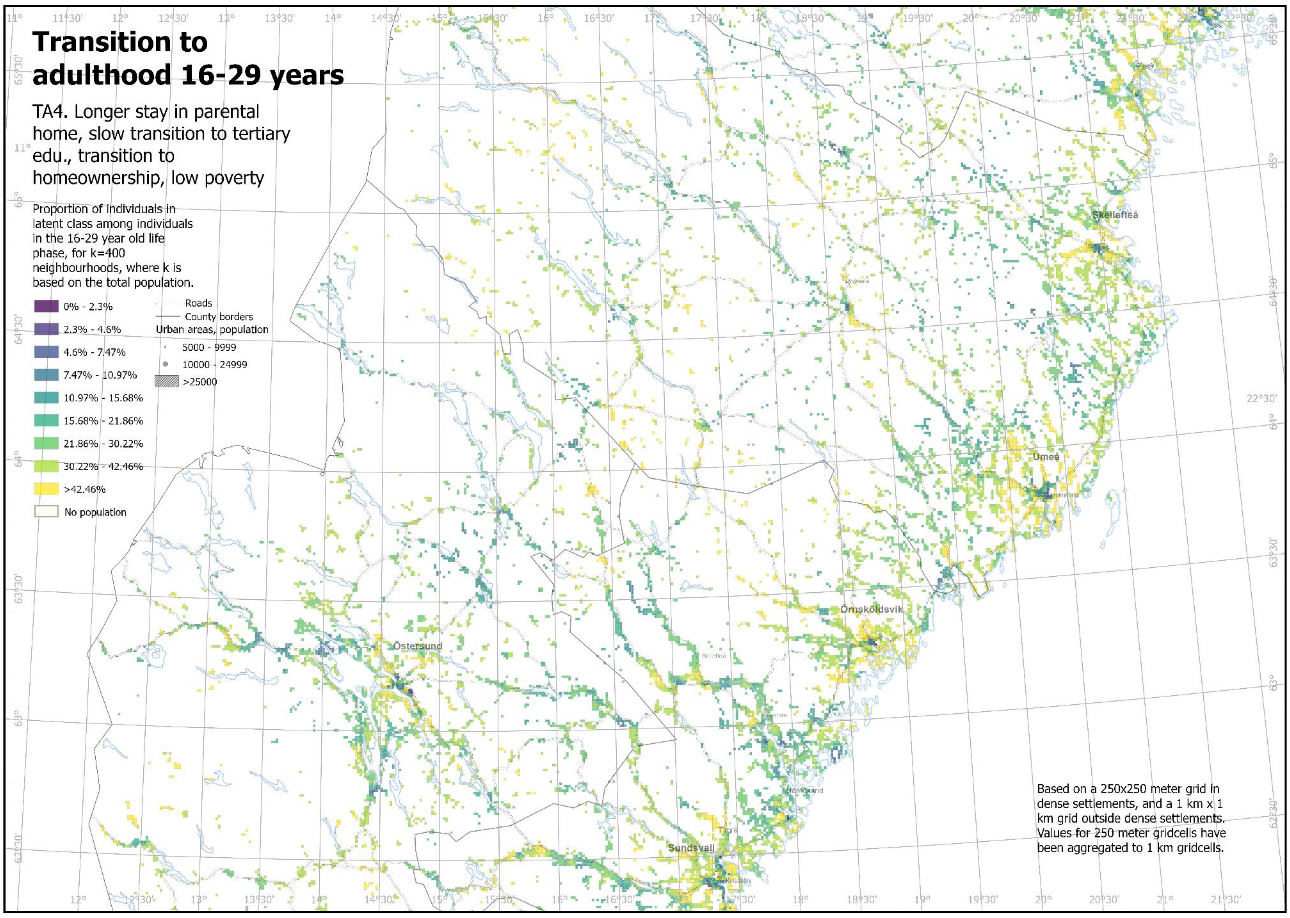

Supplementary Figure S 22 Spatial distribution of middle income homeowner life course TA4

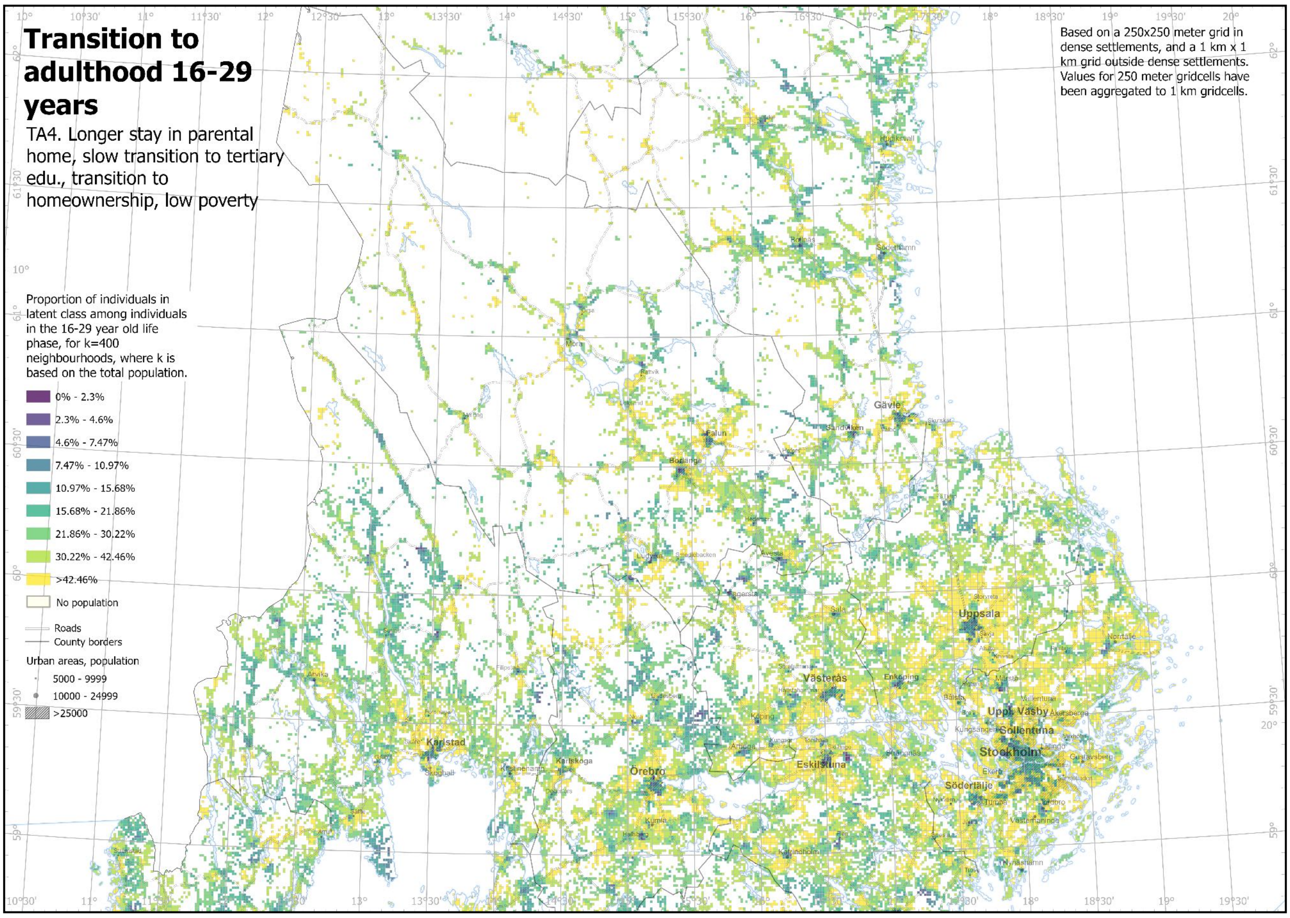

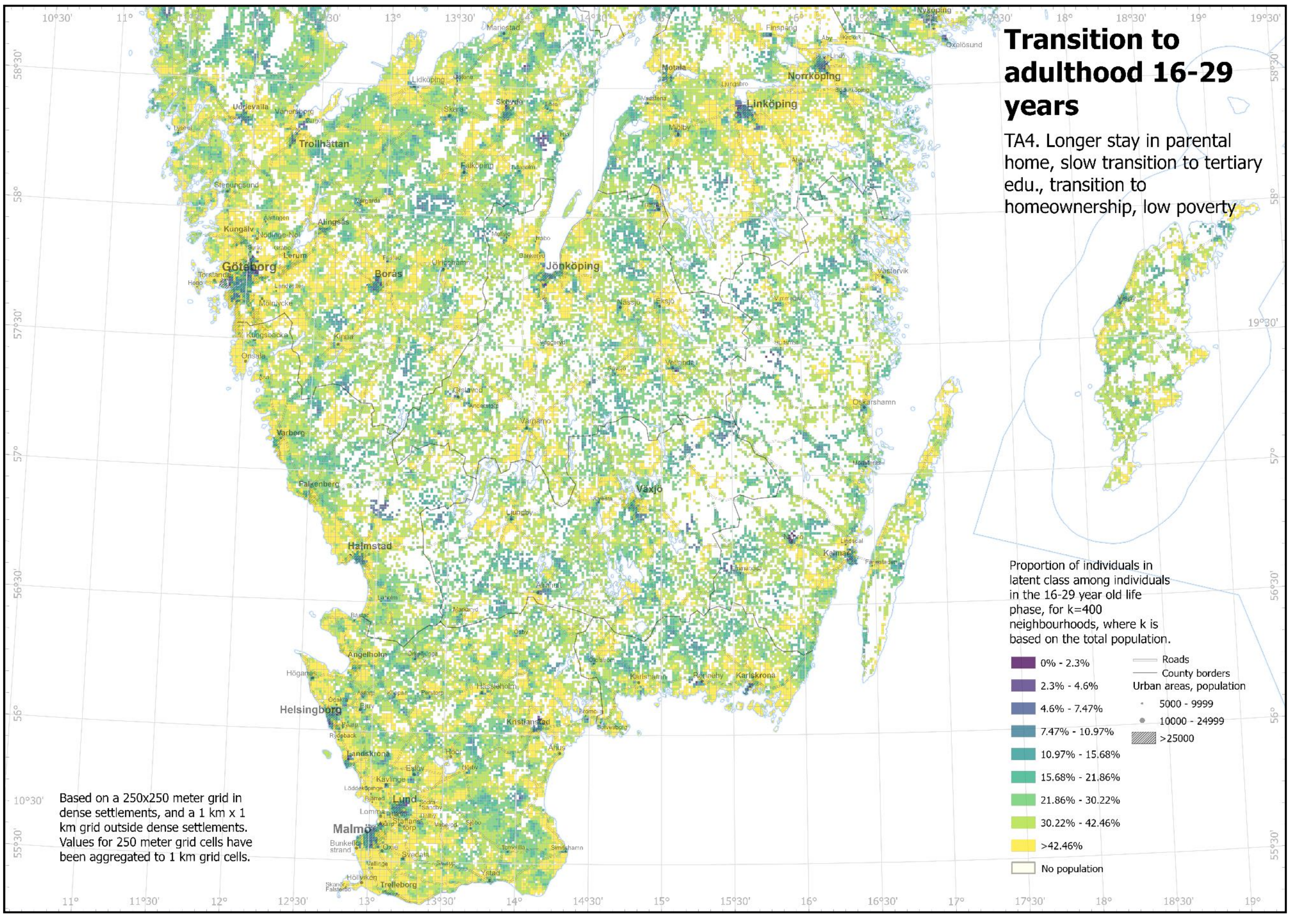

Supplementary Figure S 22 Spatial distribution of middle income homeowner life course TA4, continued

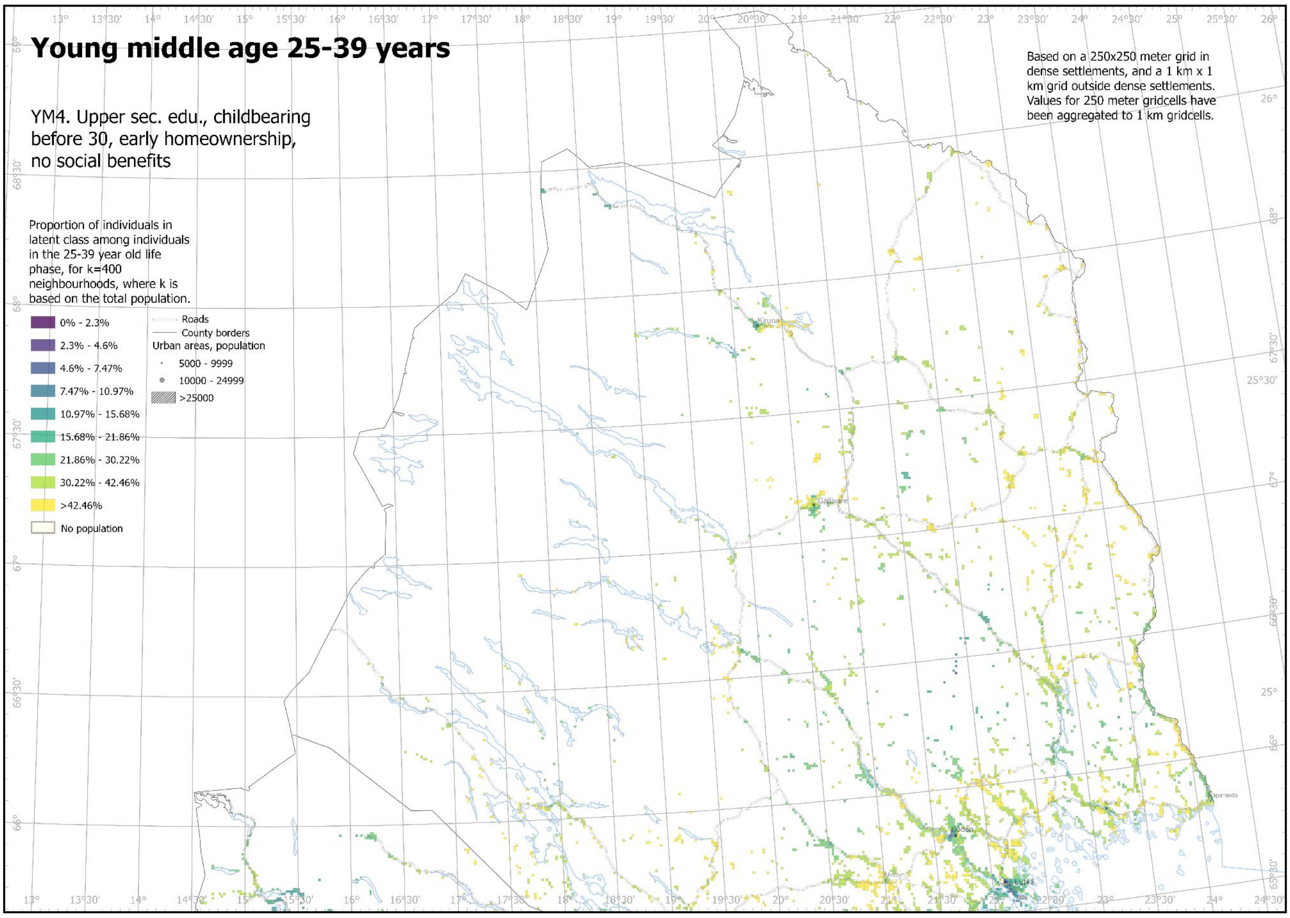

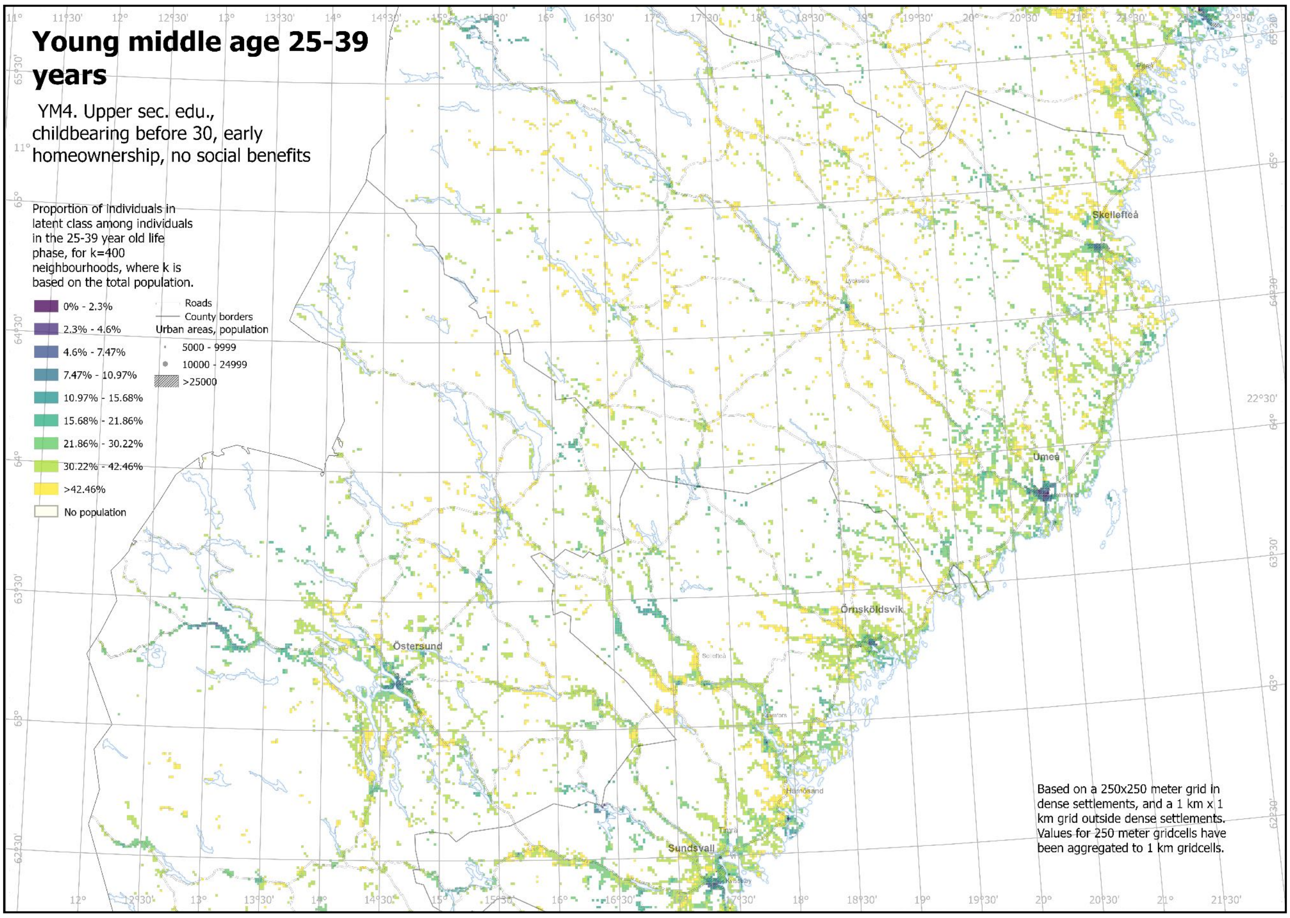

Supplementary Figure S 23 Spatial distribution of middle income homeowner life course YM4

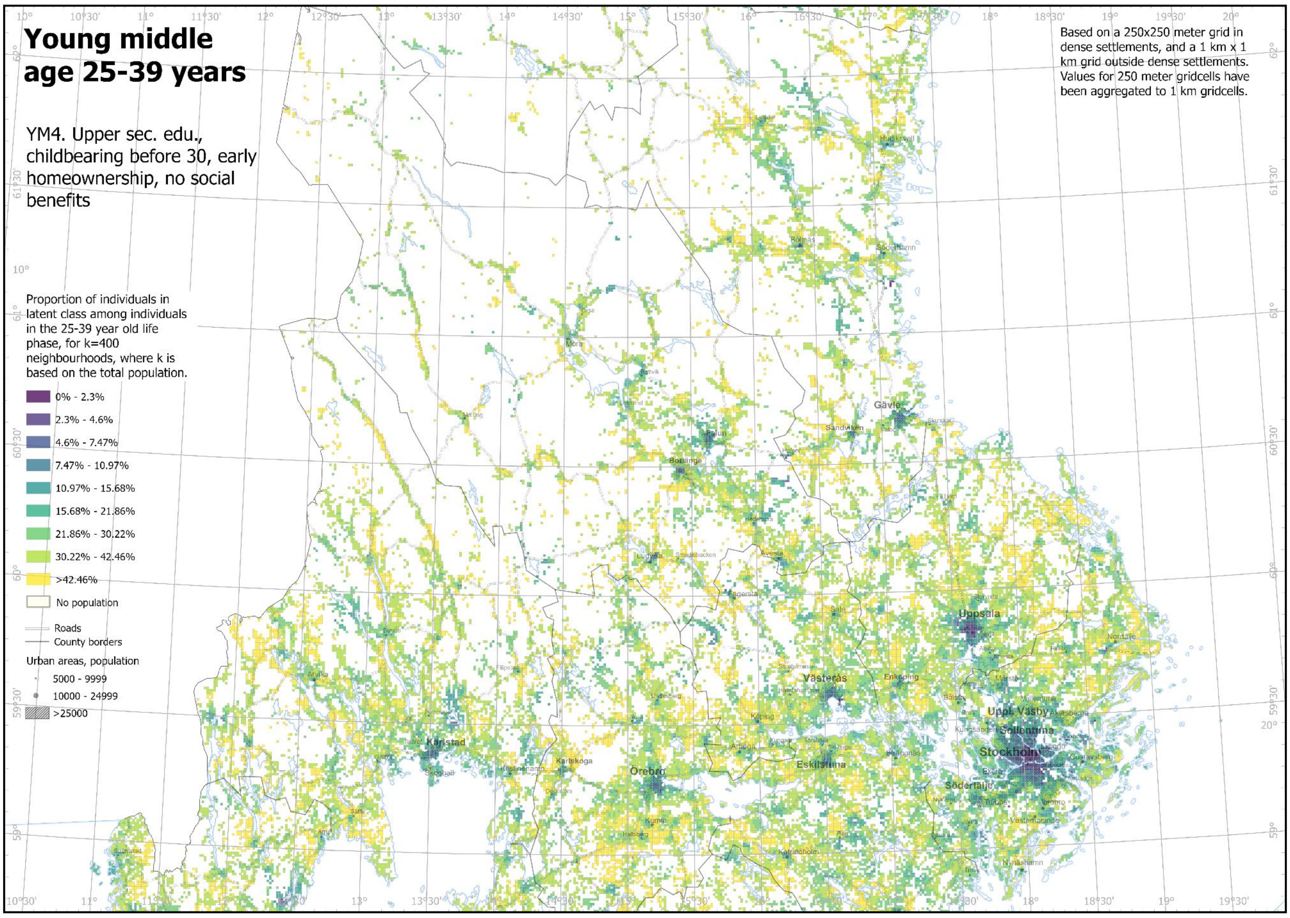

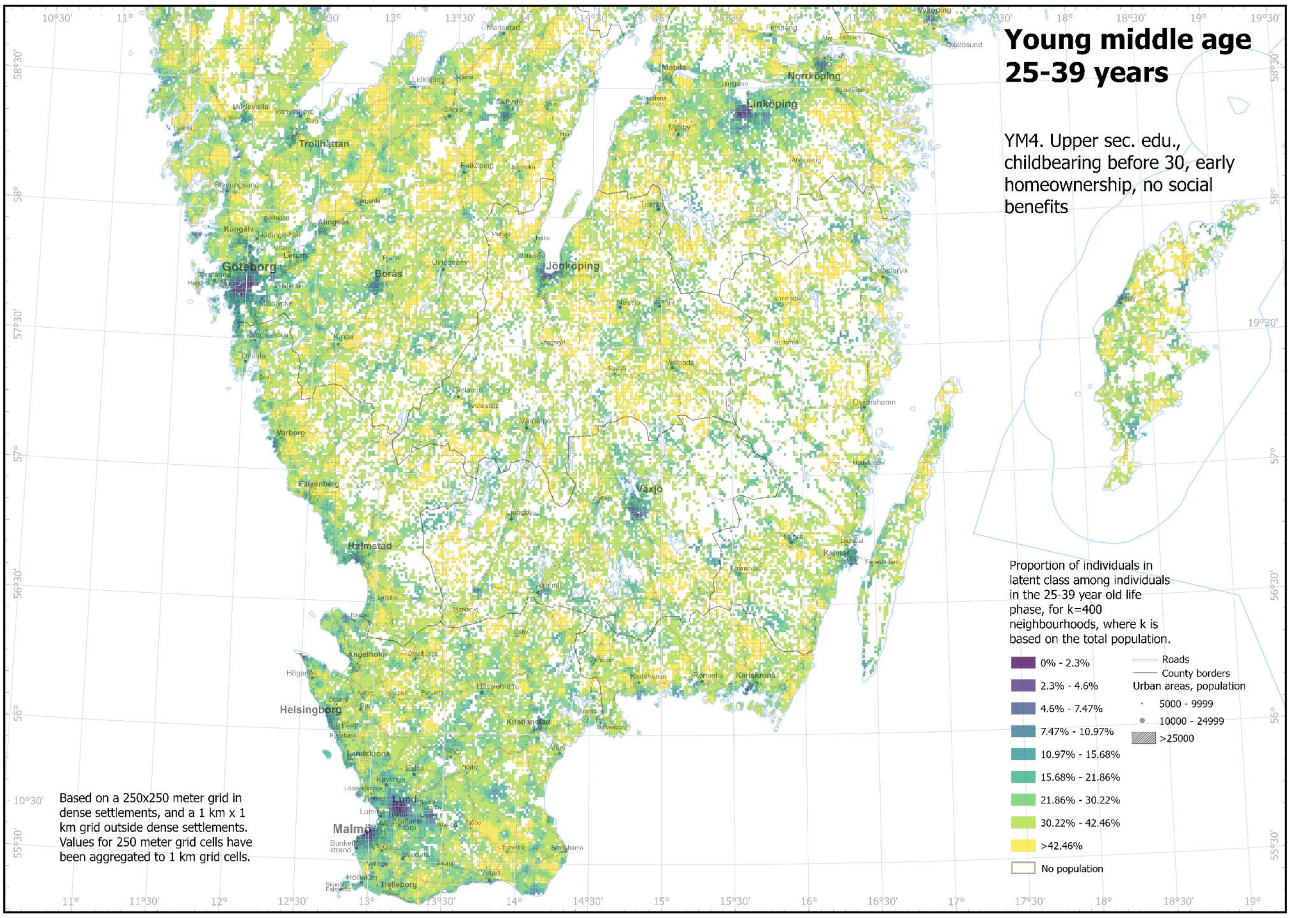

Supplementary Figure S 23 Spatial distribution of middle income homeowner life course YM4, continued

Middle income homeowner

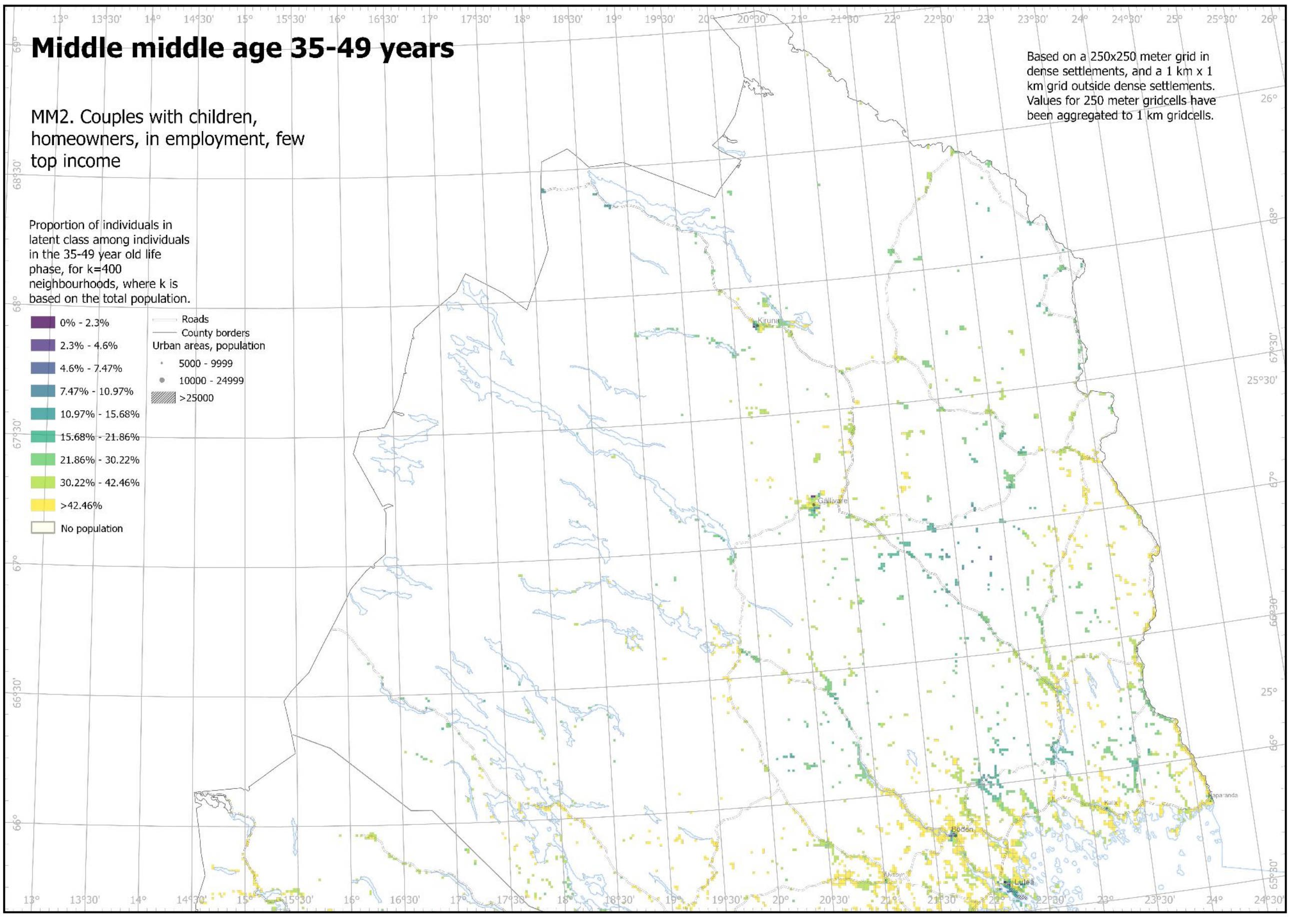

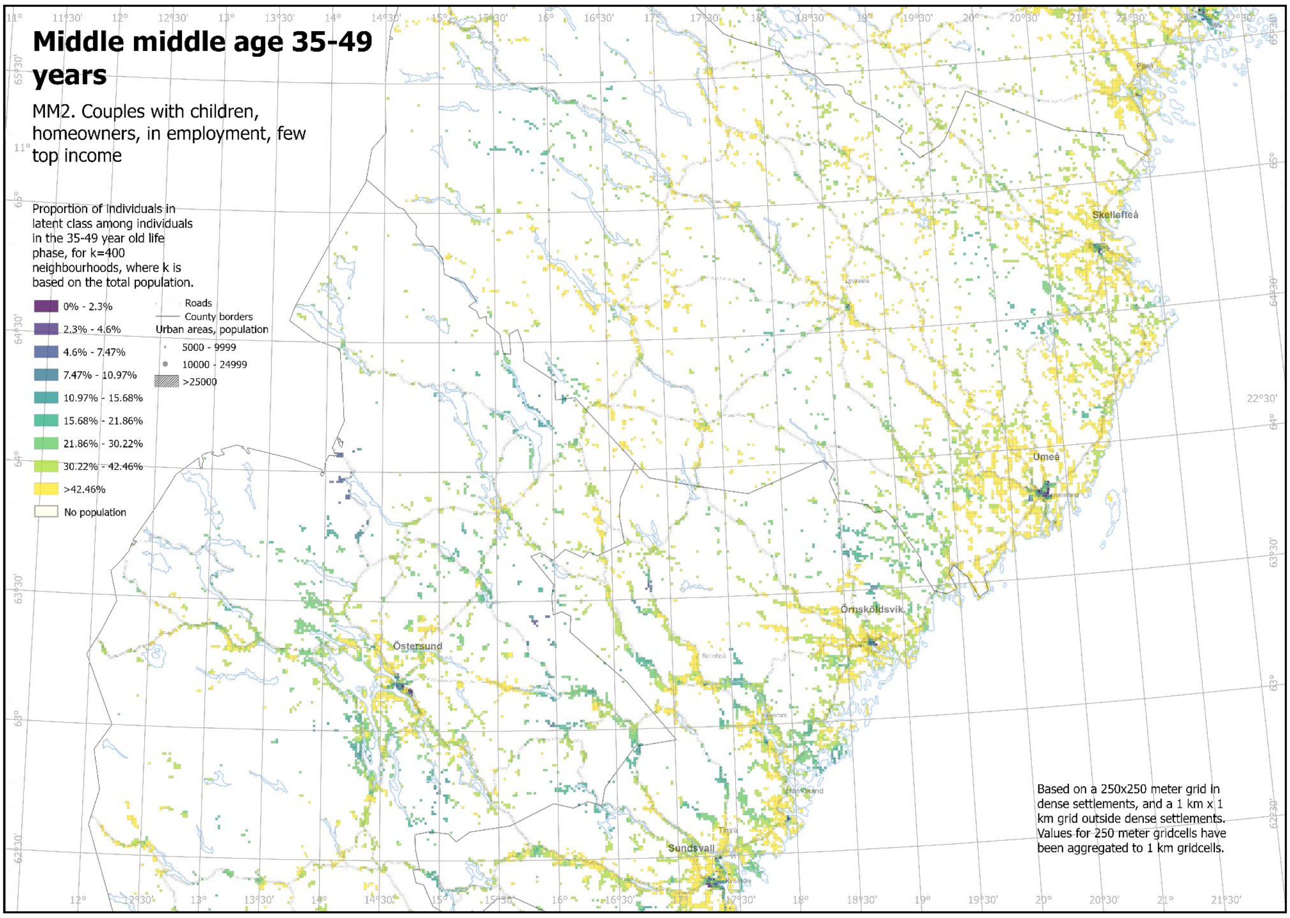

Supplementary Figure S 24 Spatial distribution of middle income homeowner life course MM2

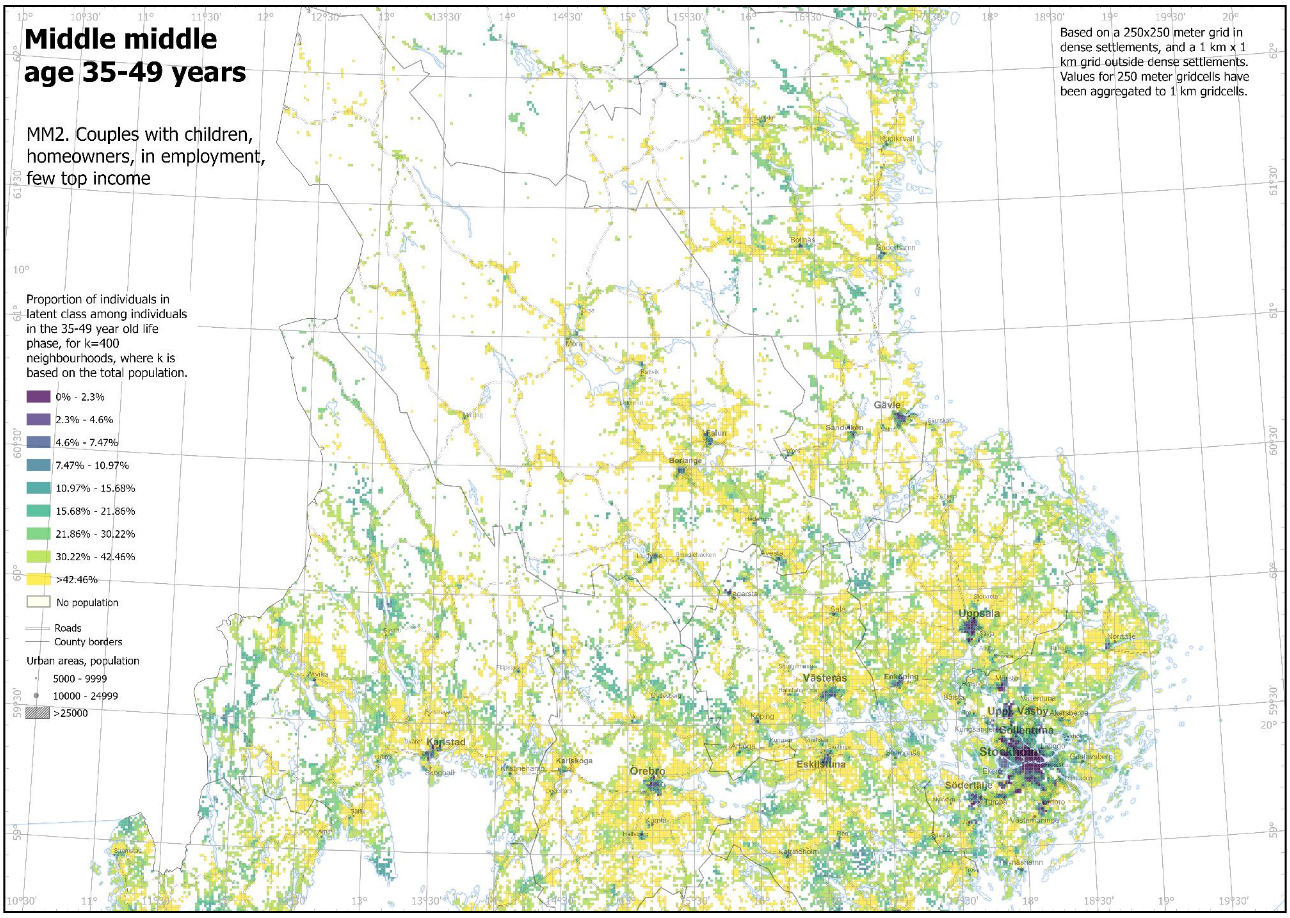

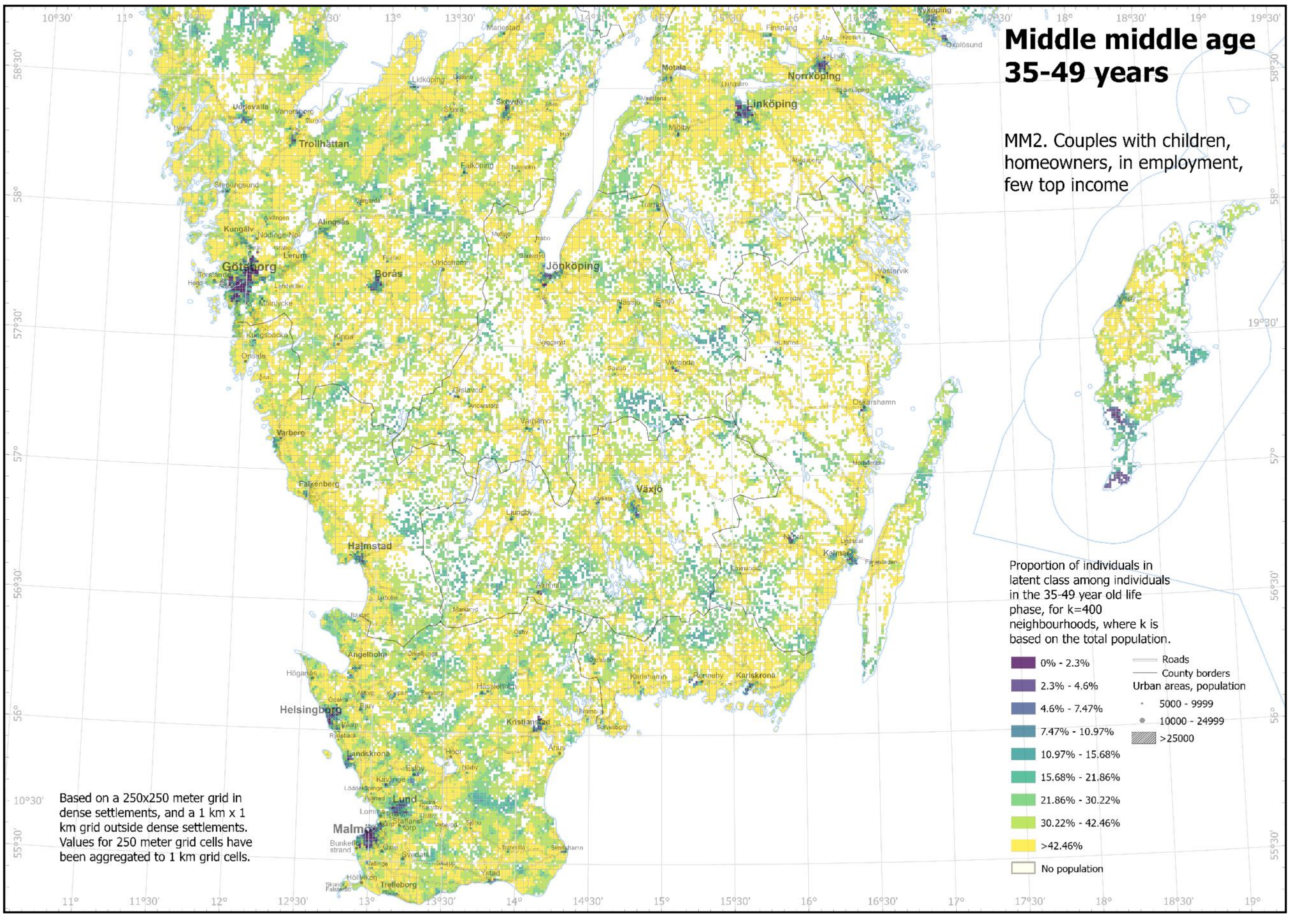

Supplementary Figure S 24 Spatial distribution of middle income homeowner life course MM2, continued

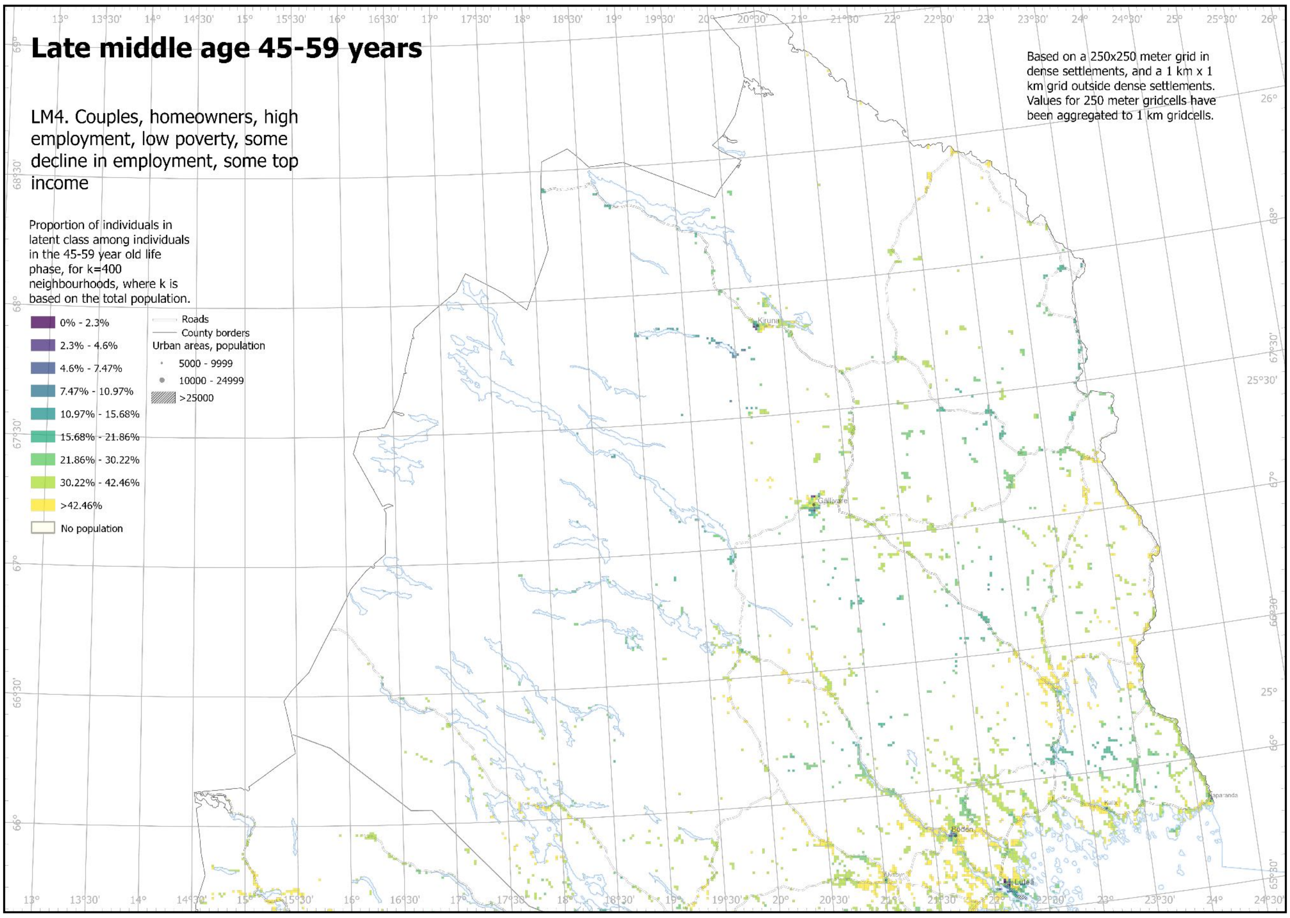

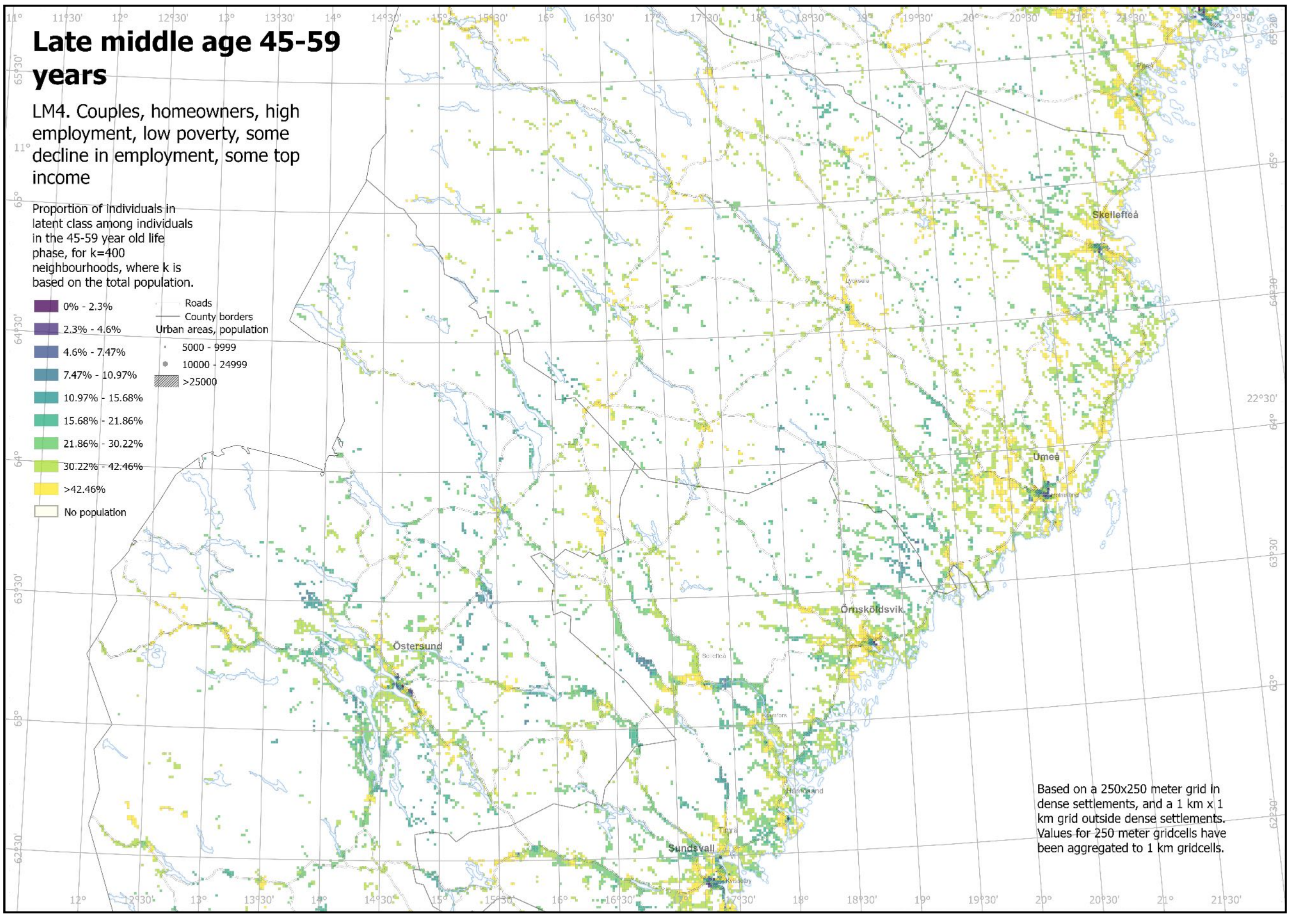

Supplementary Figure S 25 Spatial distribution of middle income homeowner life course LM4

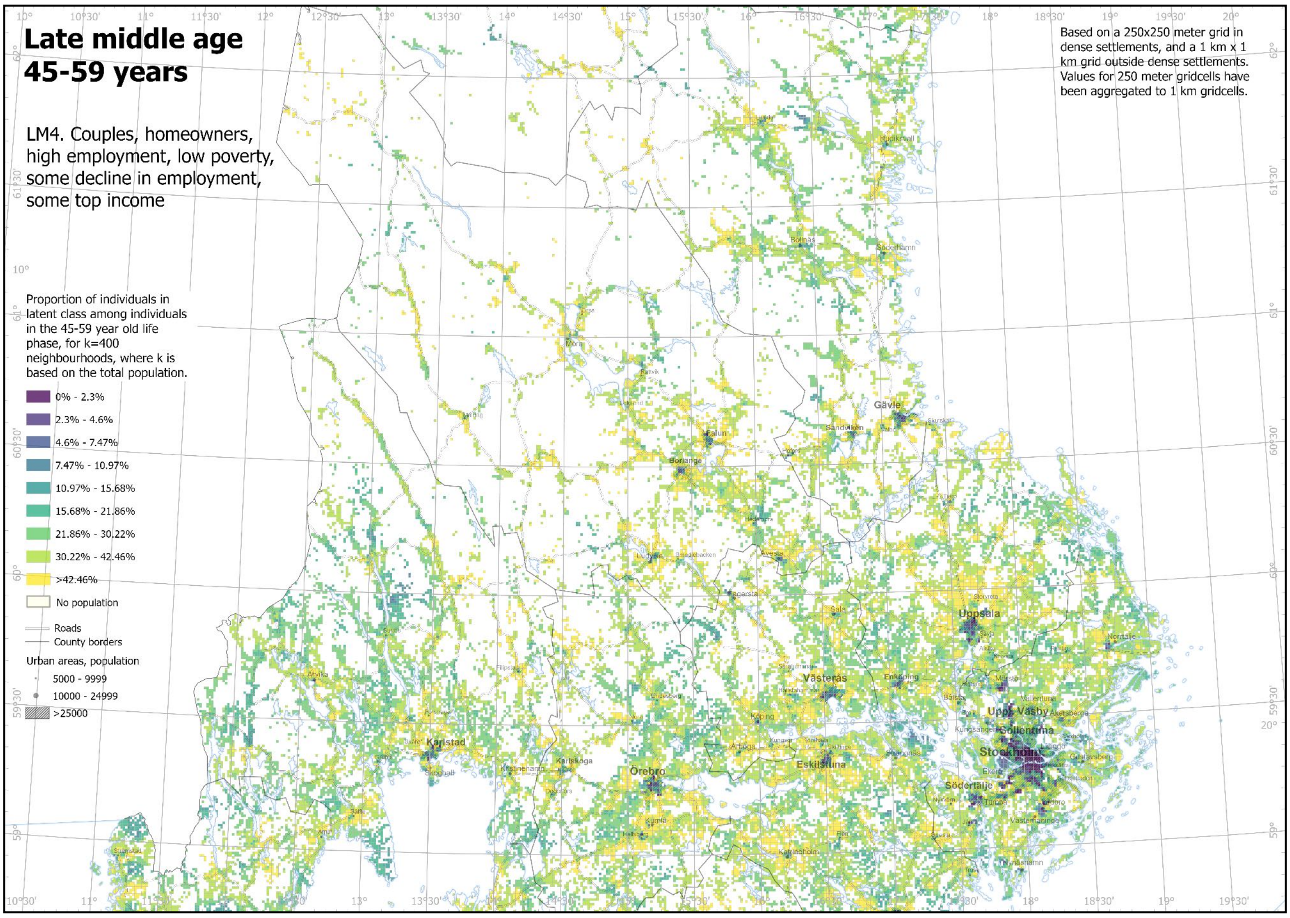

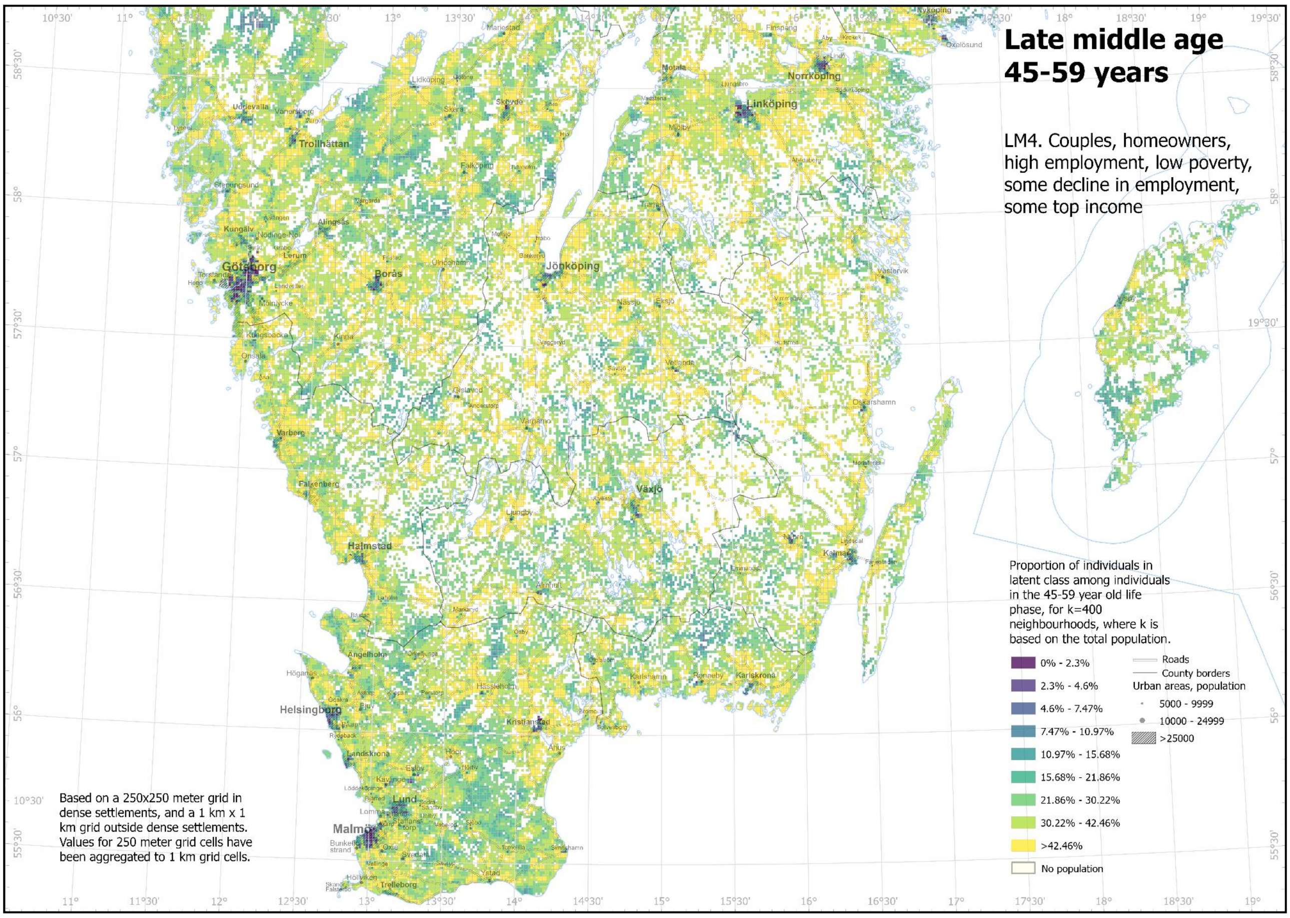

Supplementary Figure S 25 Spatial distribution of middle income homeowner life course LM4, continued

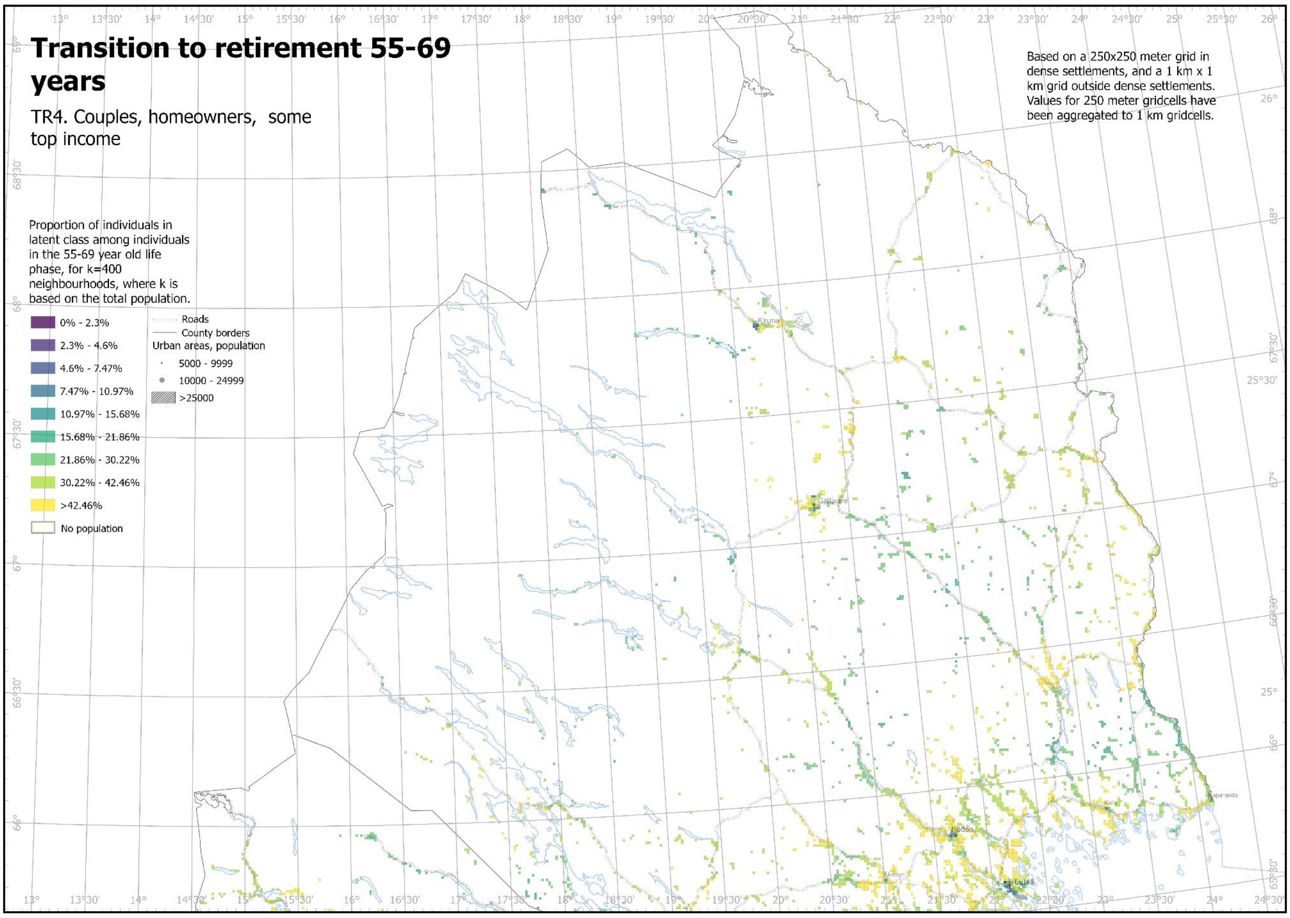

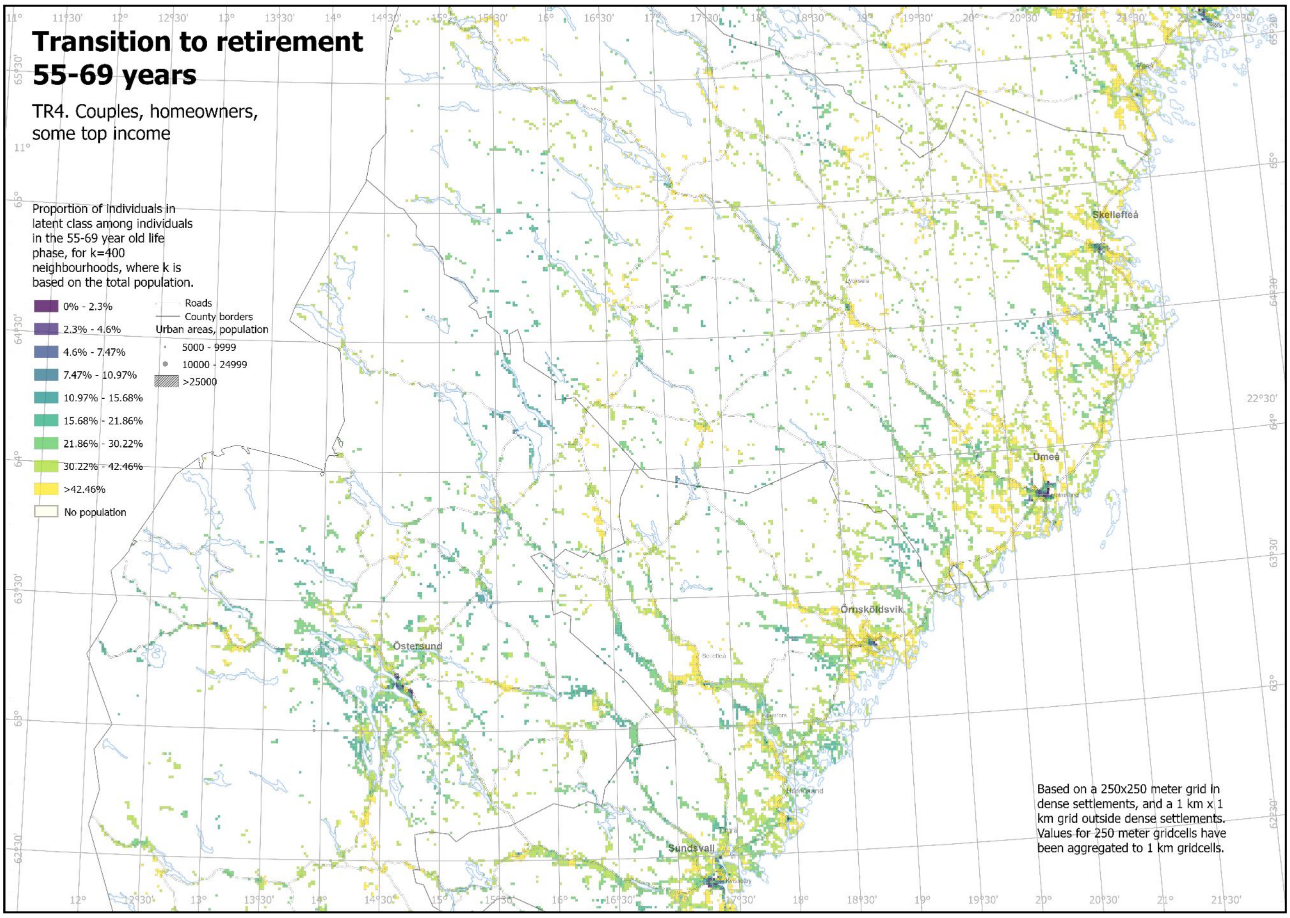

Supplementary Figure S 26 Spatial distribution of middle income homeowner life course TR4

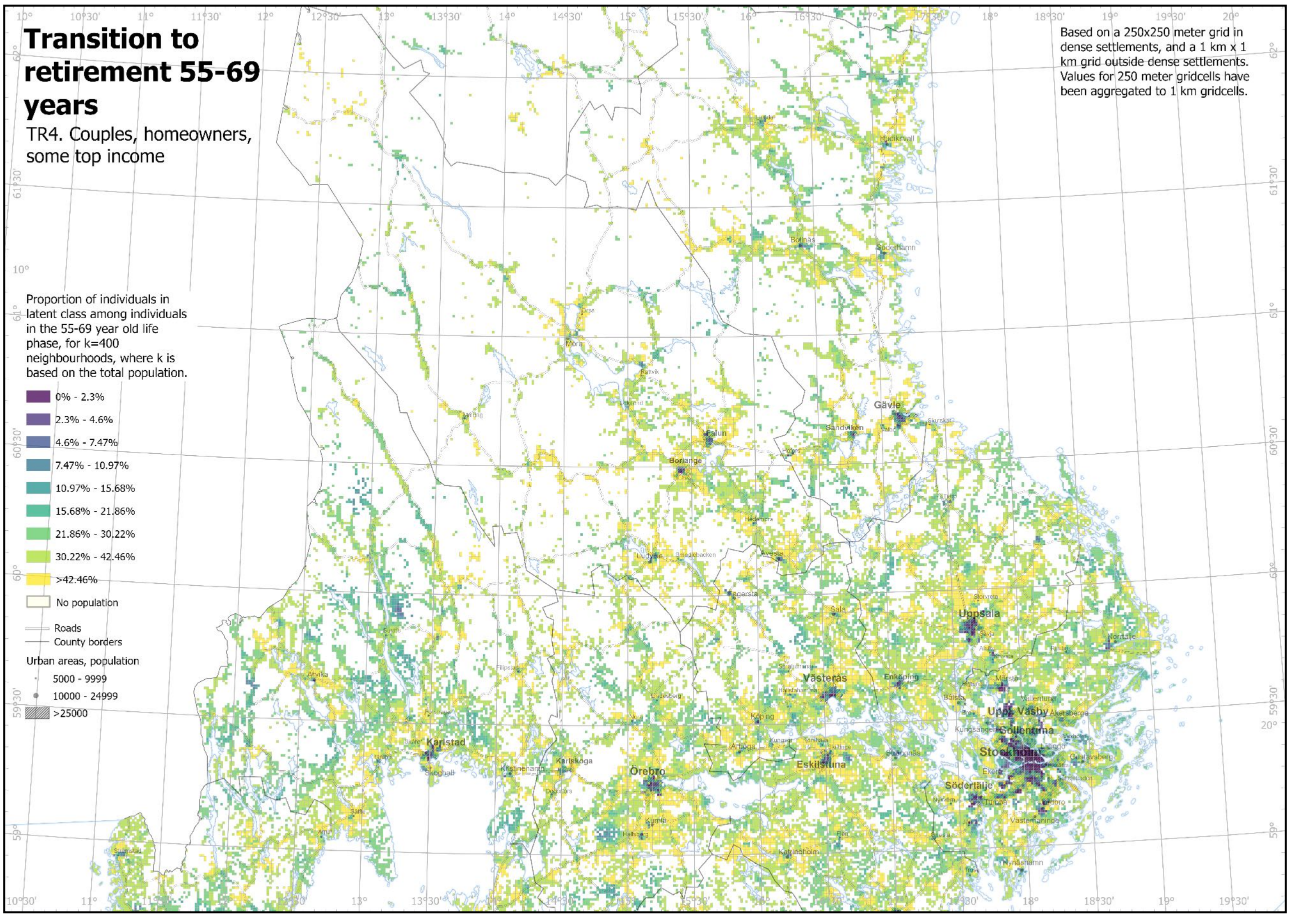

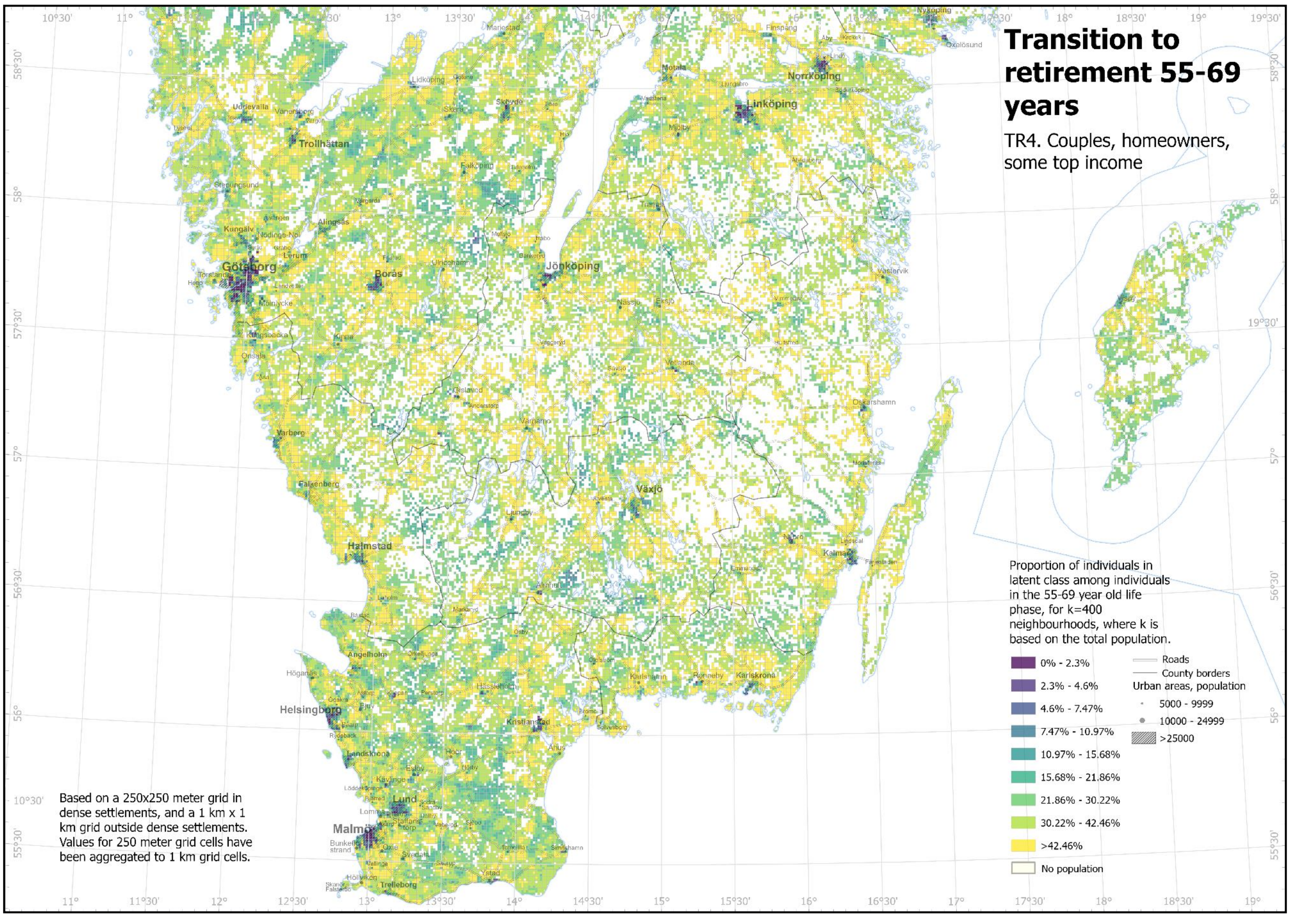

Supplementary Figure S 26 Spatial distribution of middle income homeowner life course TR4, continued

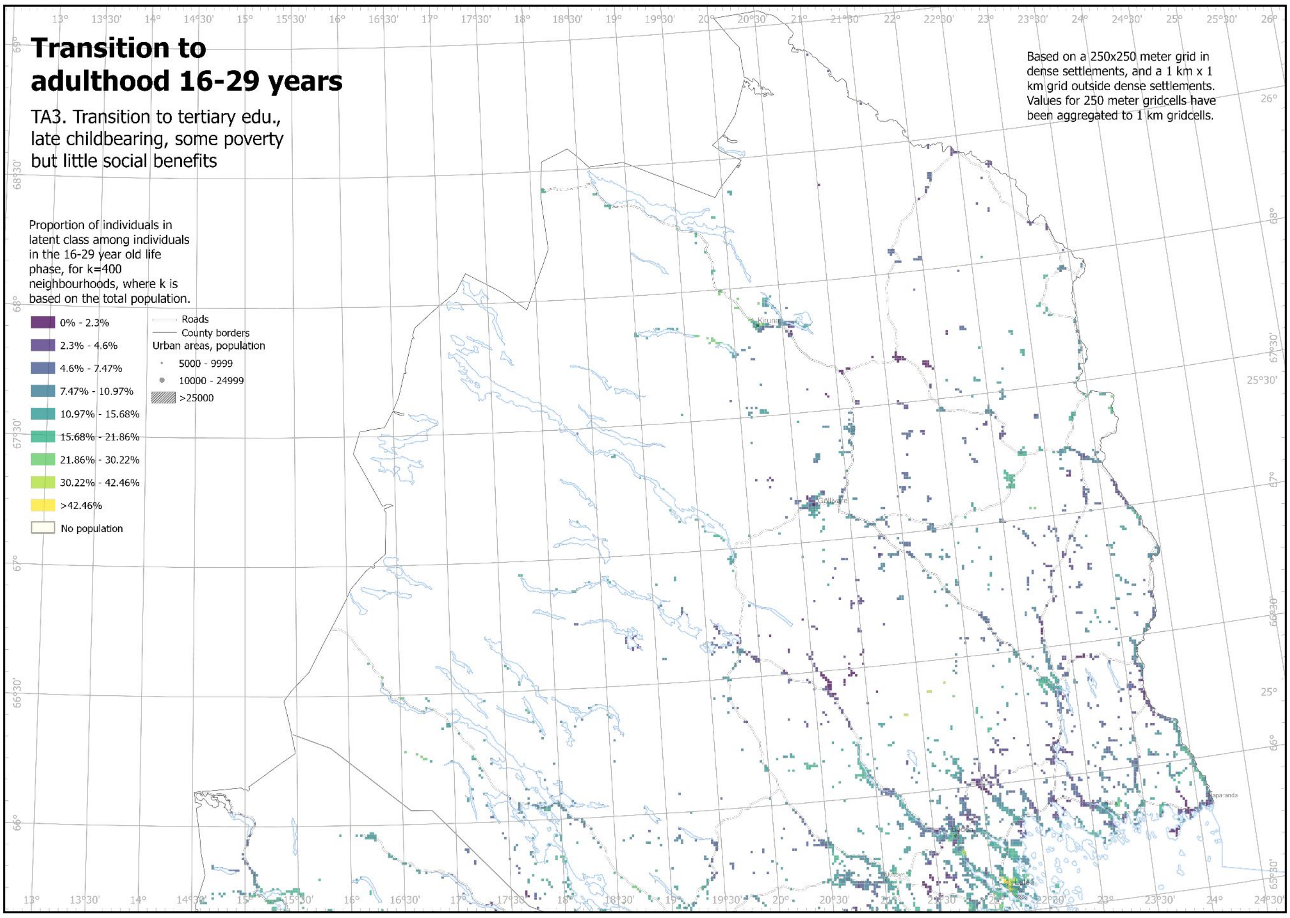

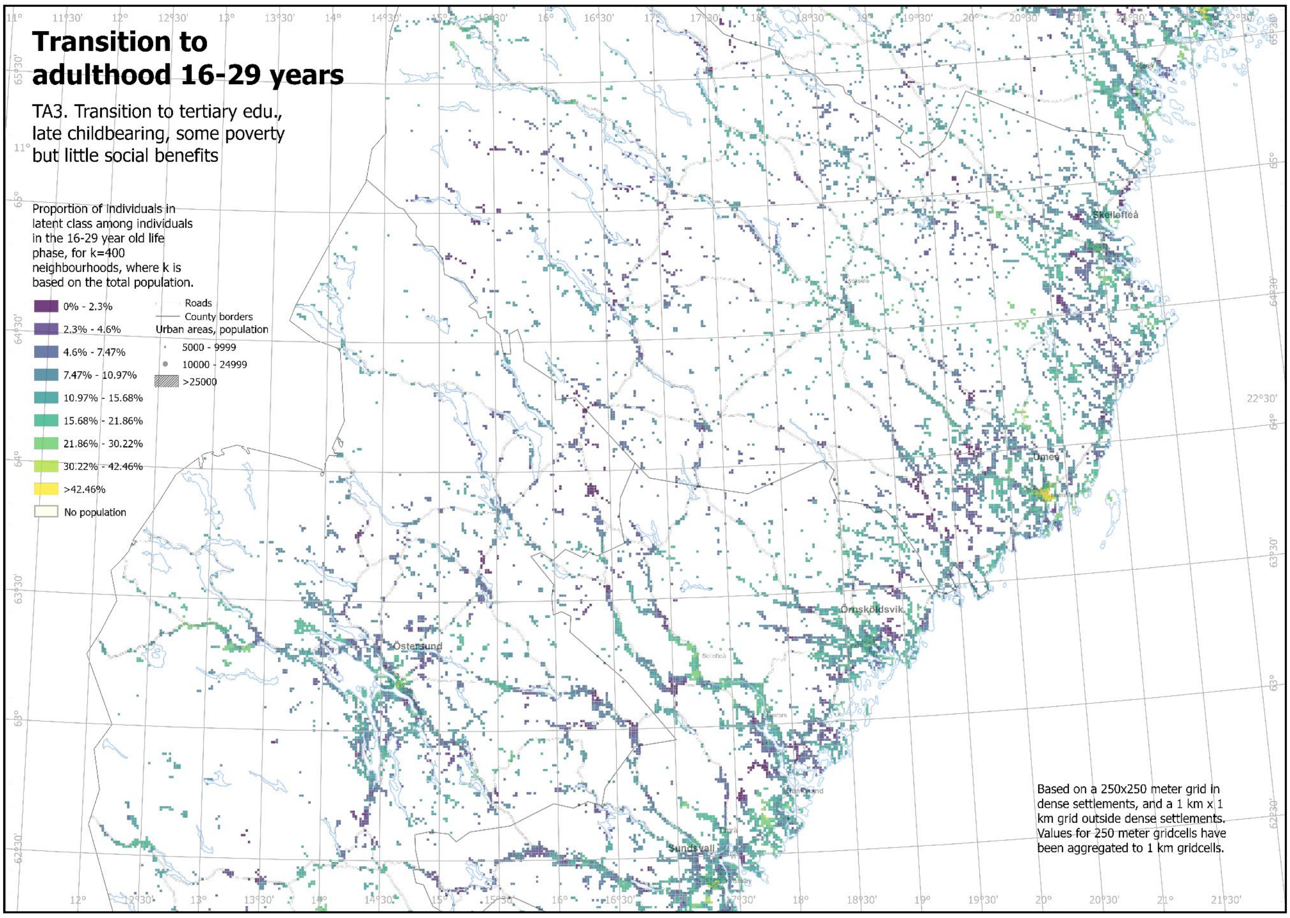

Supplementary Figure S 27 Spatial distribution of advantaged life course TA3

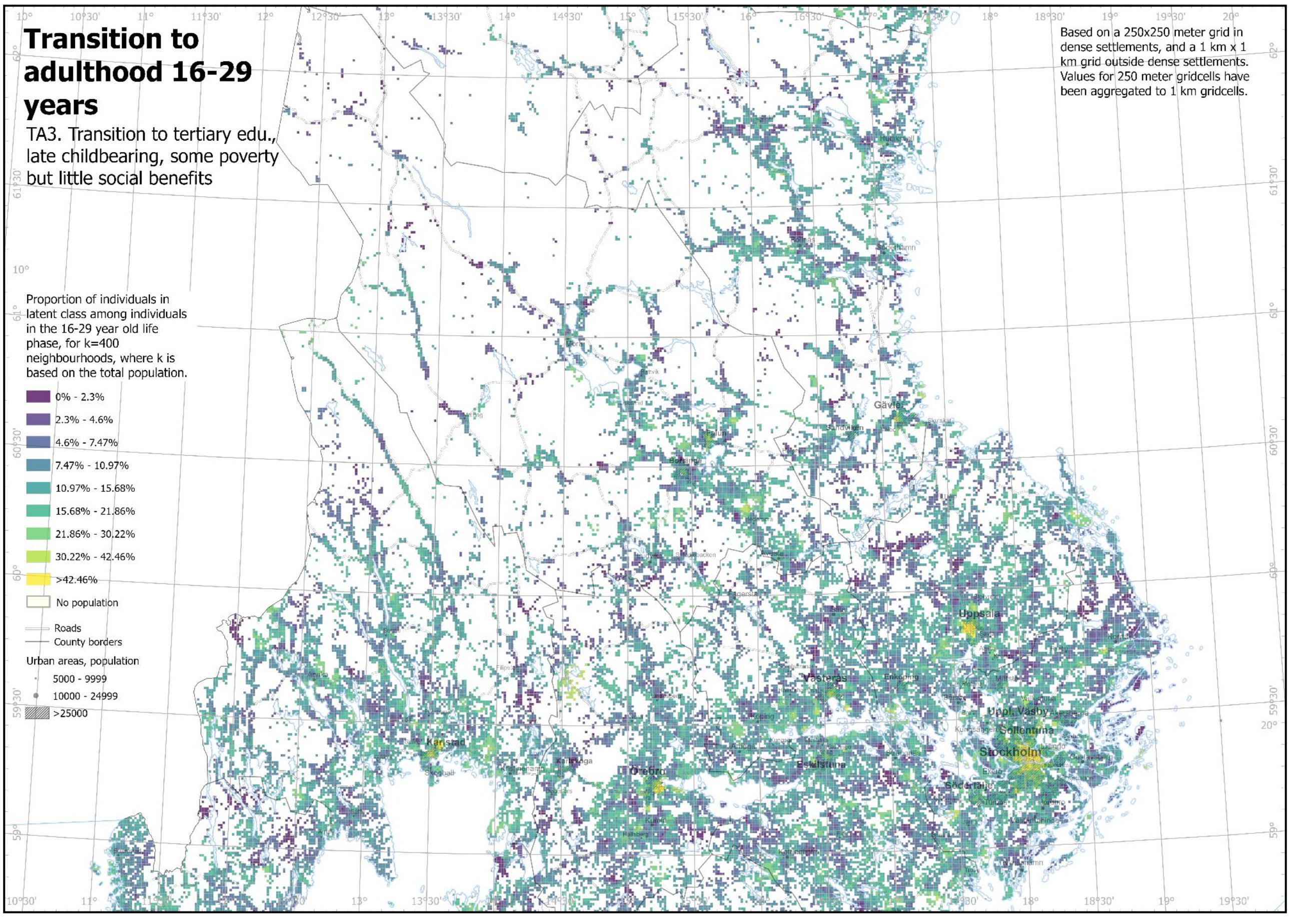

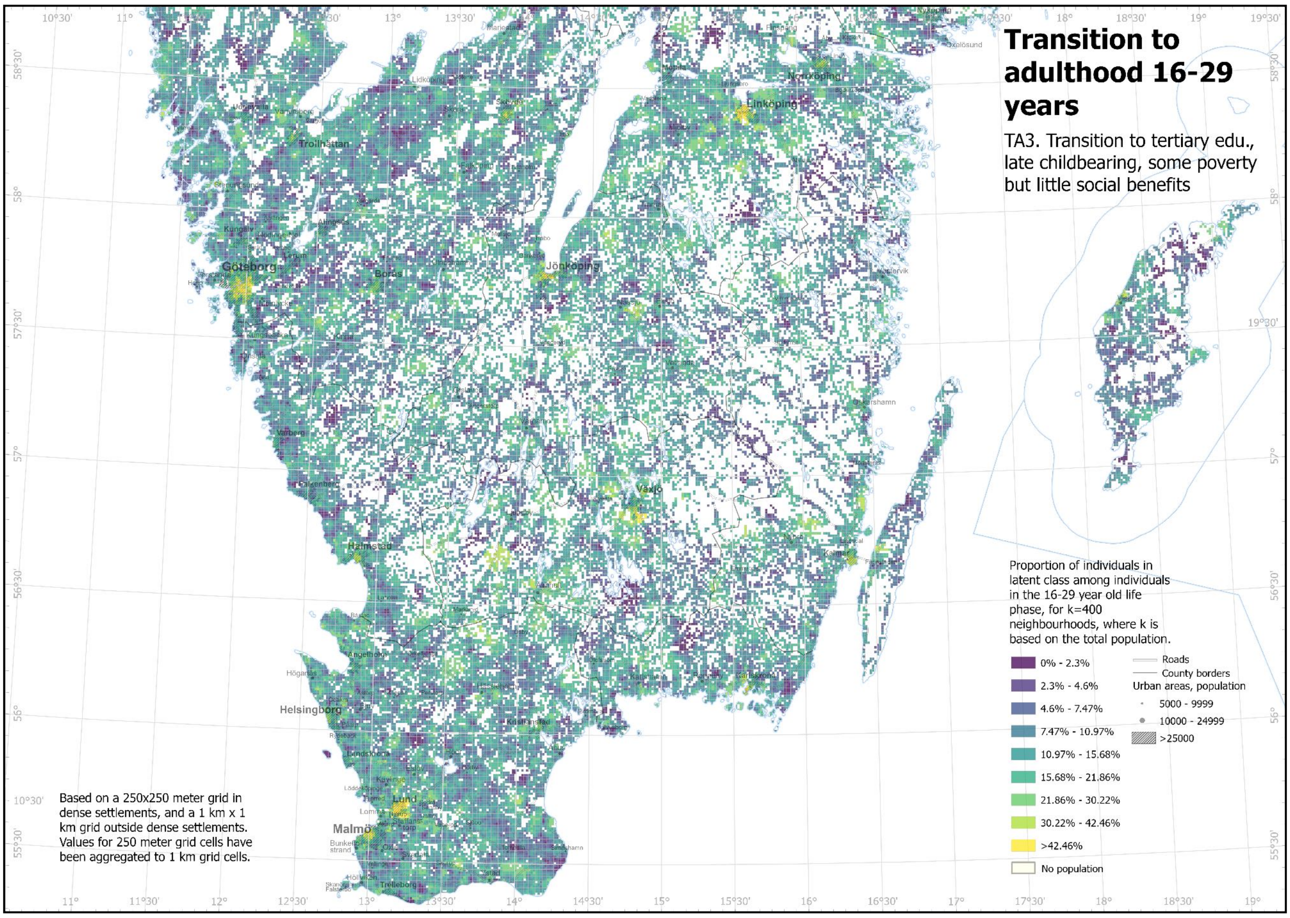

Supplementary Figure S 27 Spatial distribution of advantaged life course TA3, continued

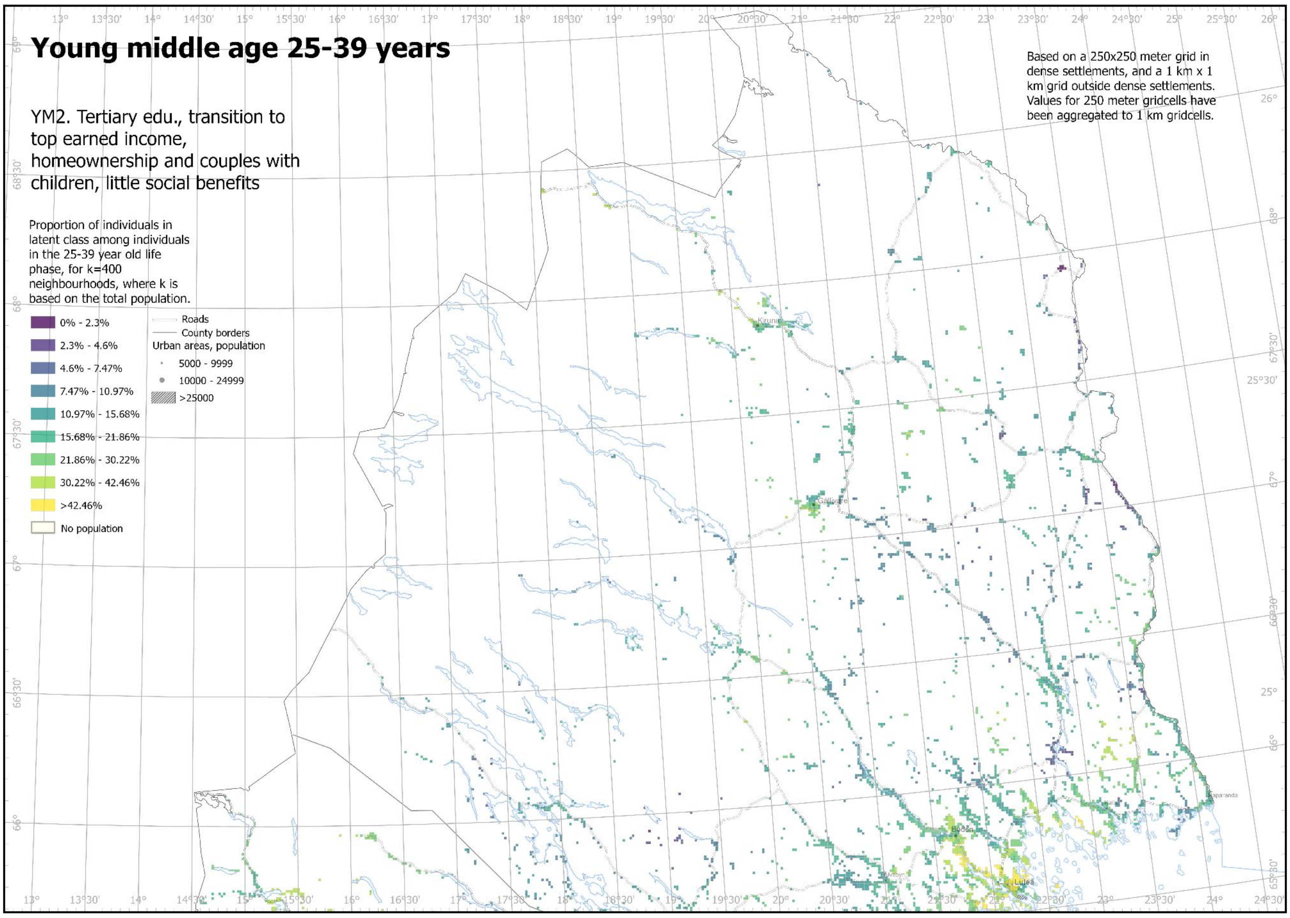

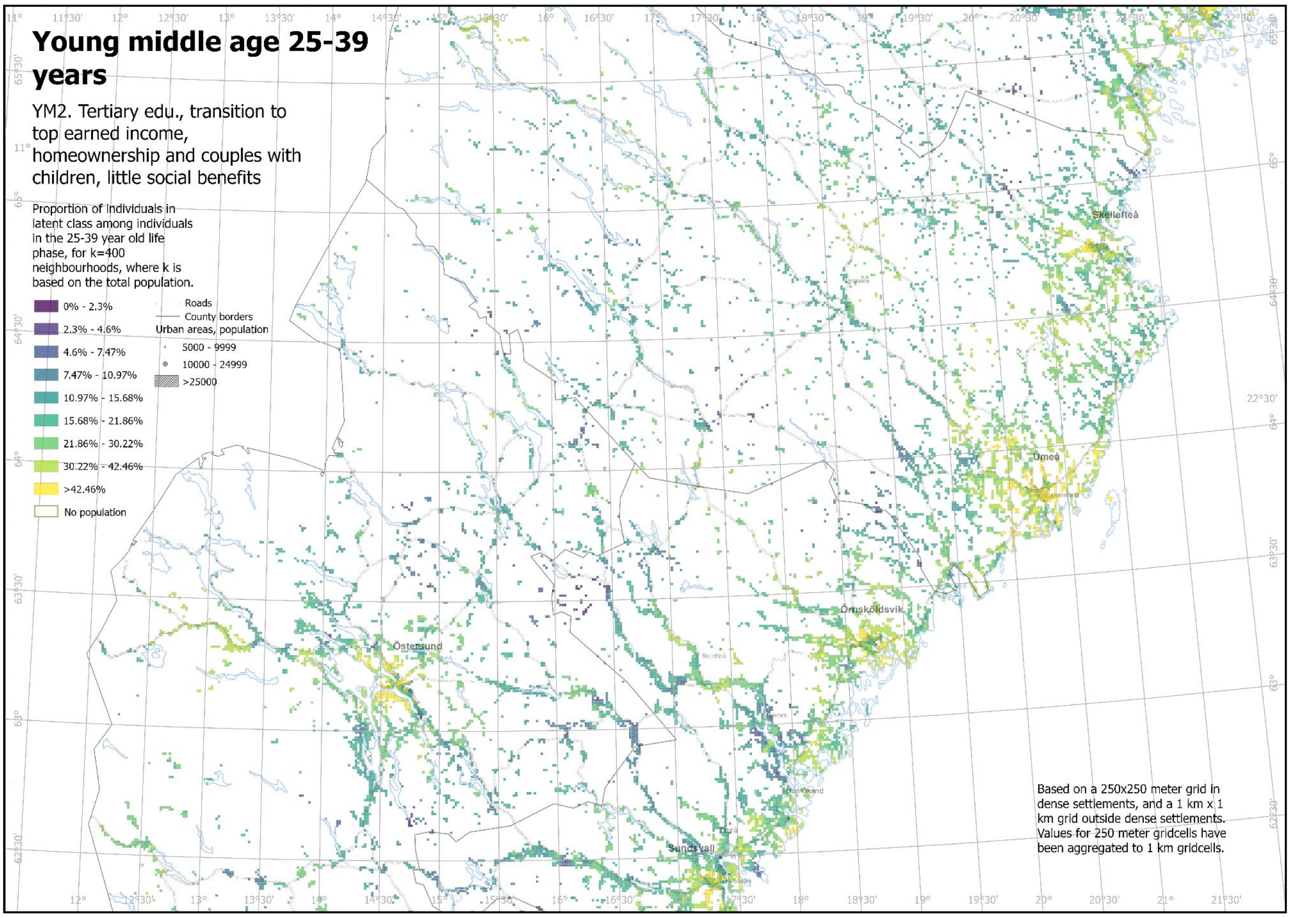

Supplementary Figure S 28 Spatial distribution of advantaged life course YM2

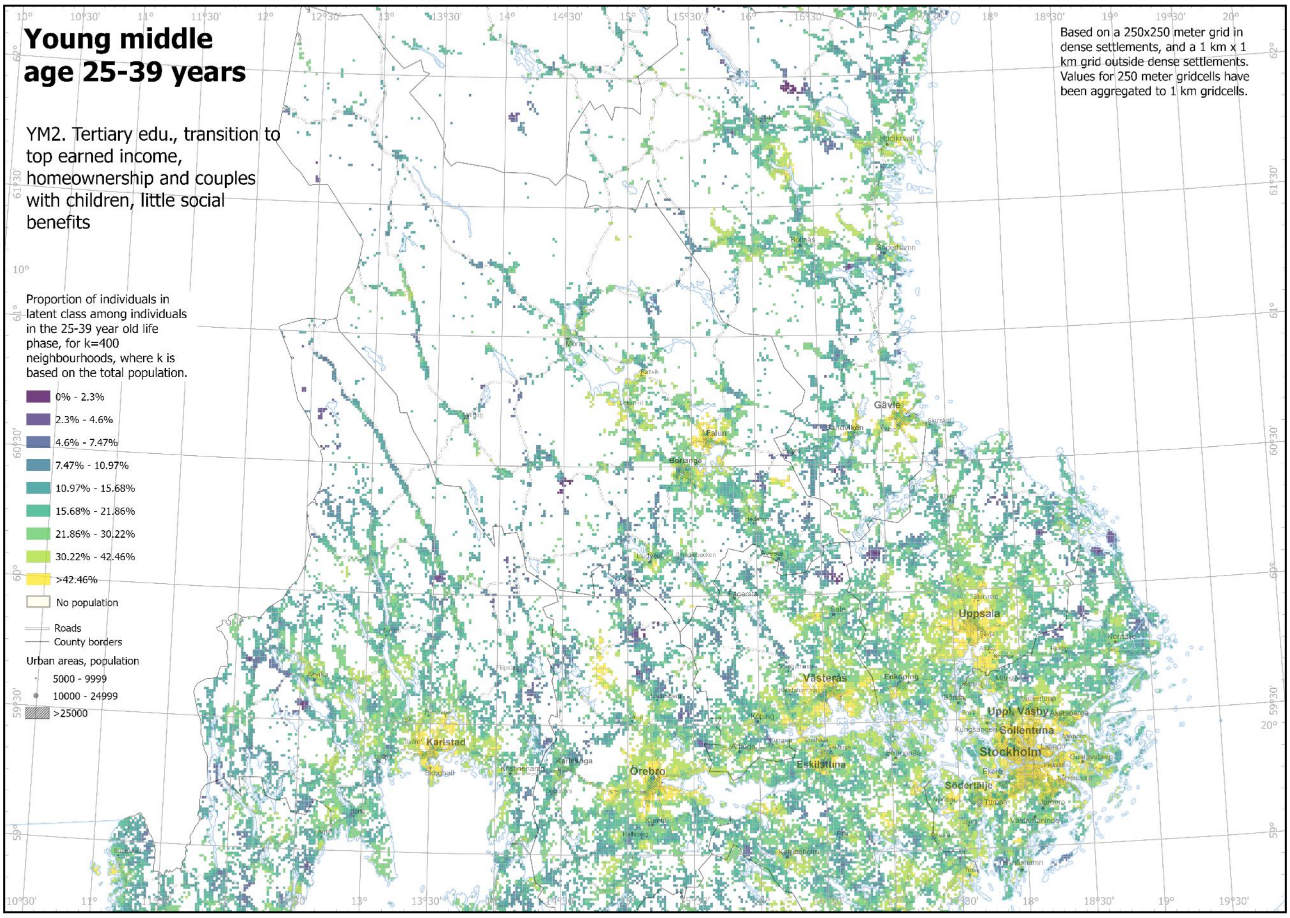

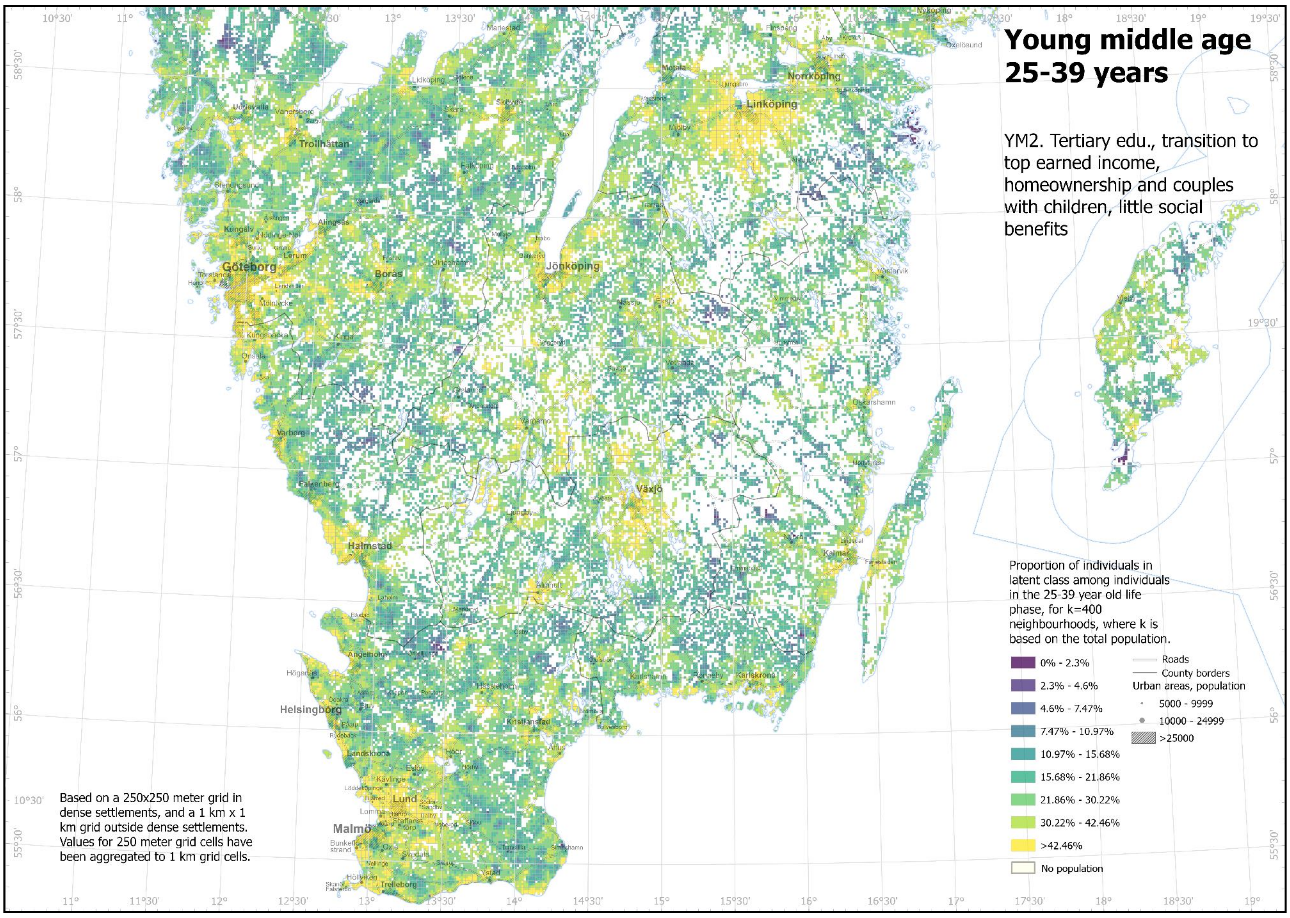

Supplementary Figure S 28 Spatial distribution of advantaged life course YM2, continued

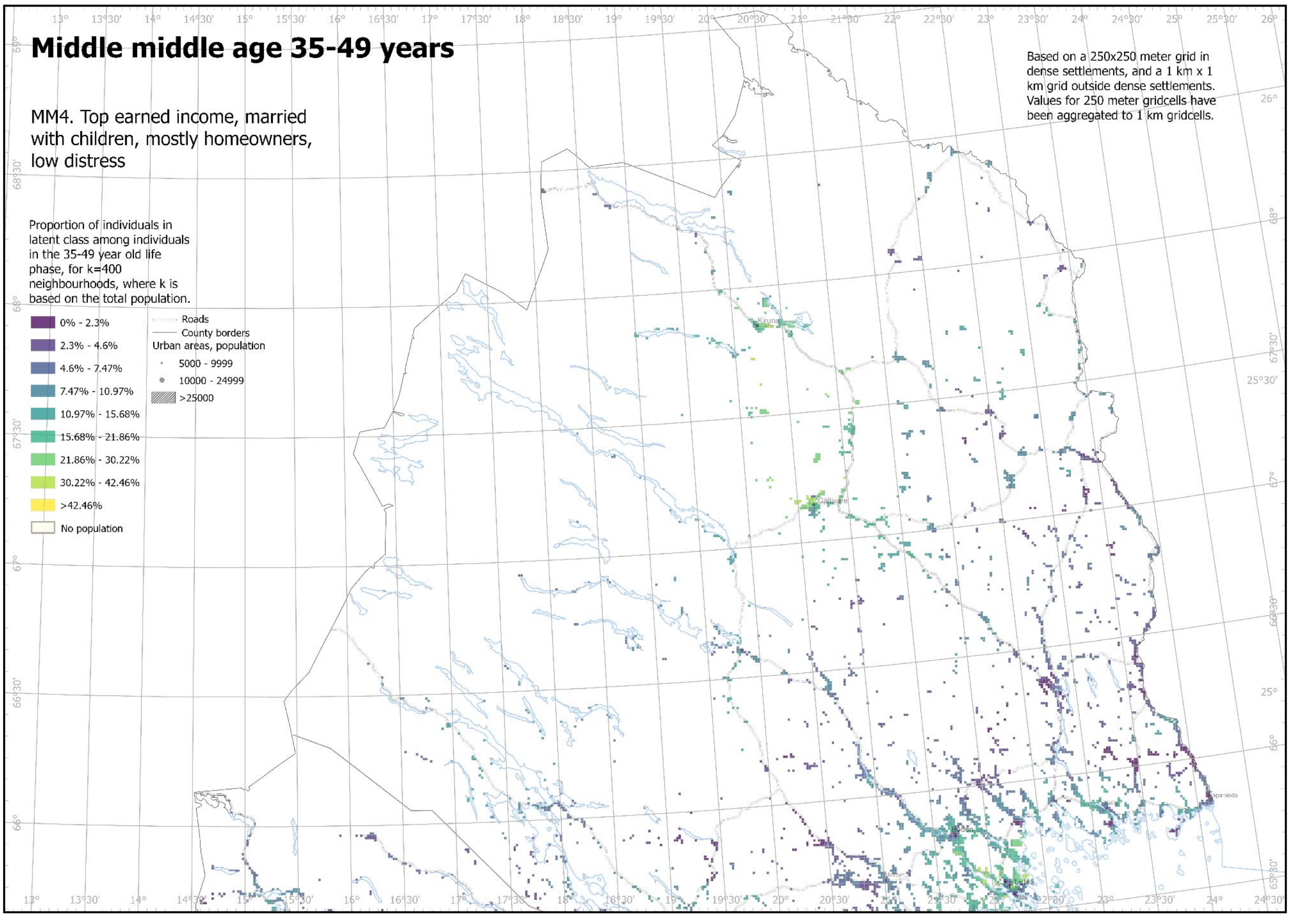

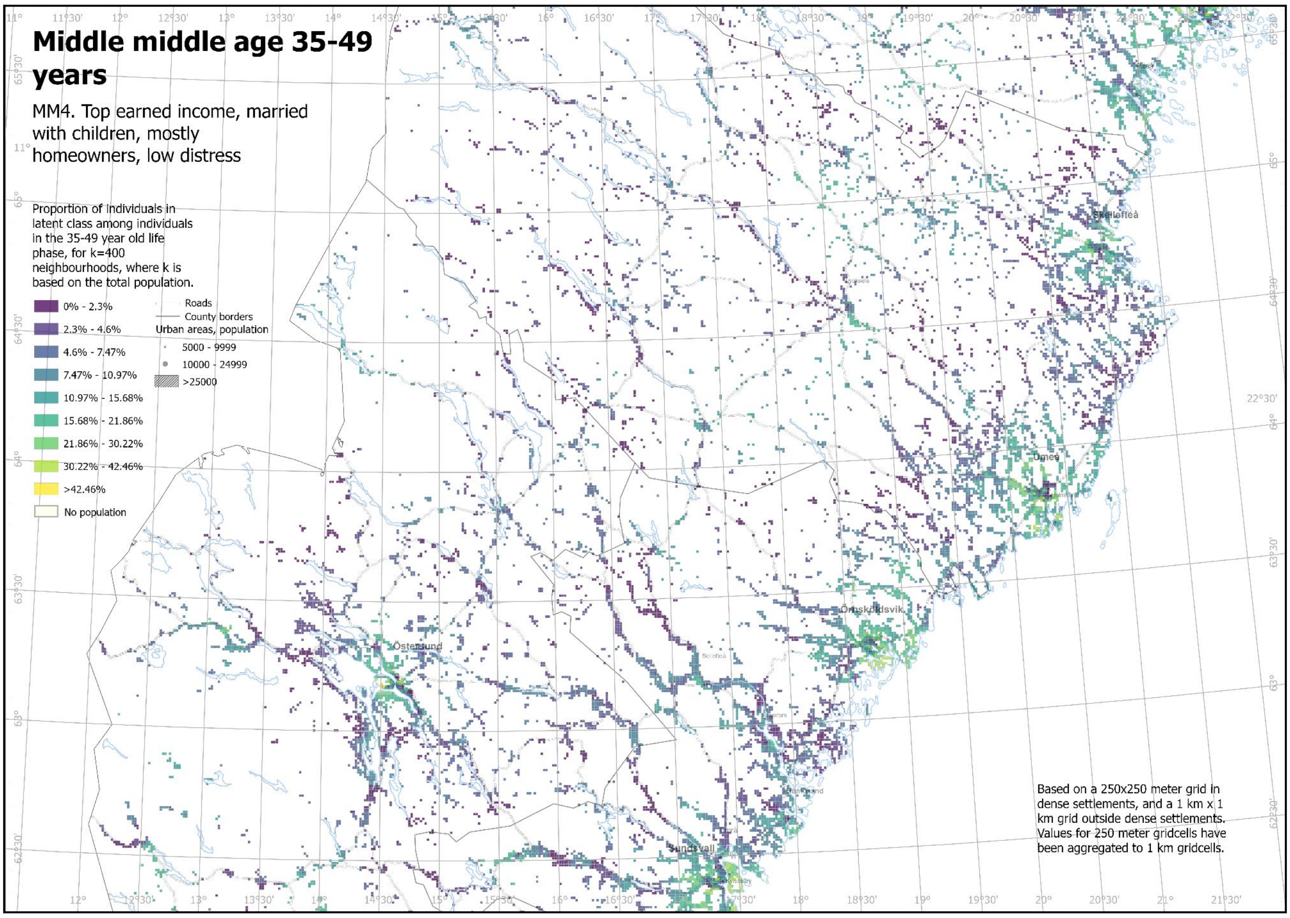

Supplementary Figure S 29 Spatial distribution of advantaged life course MM4

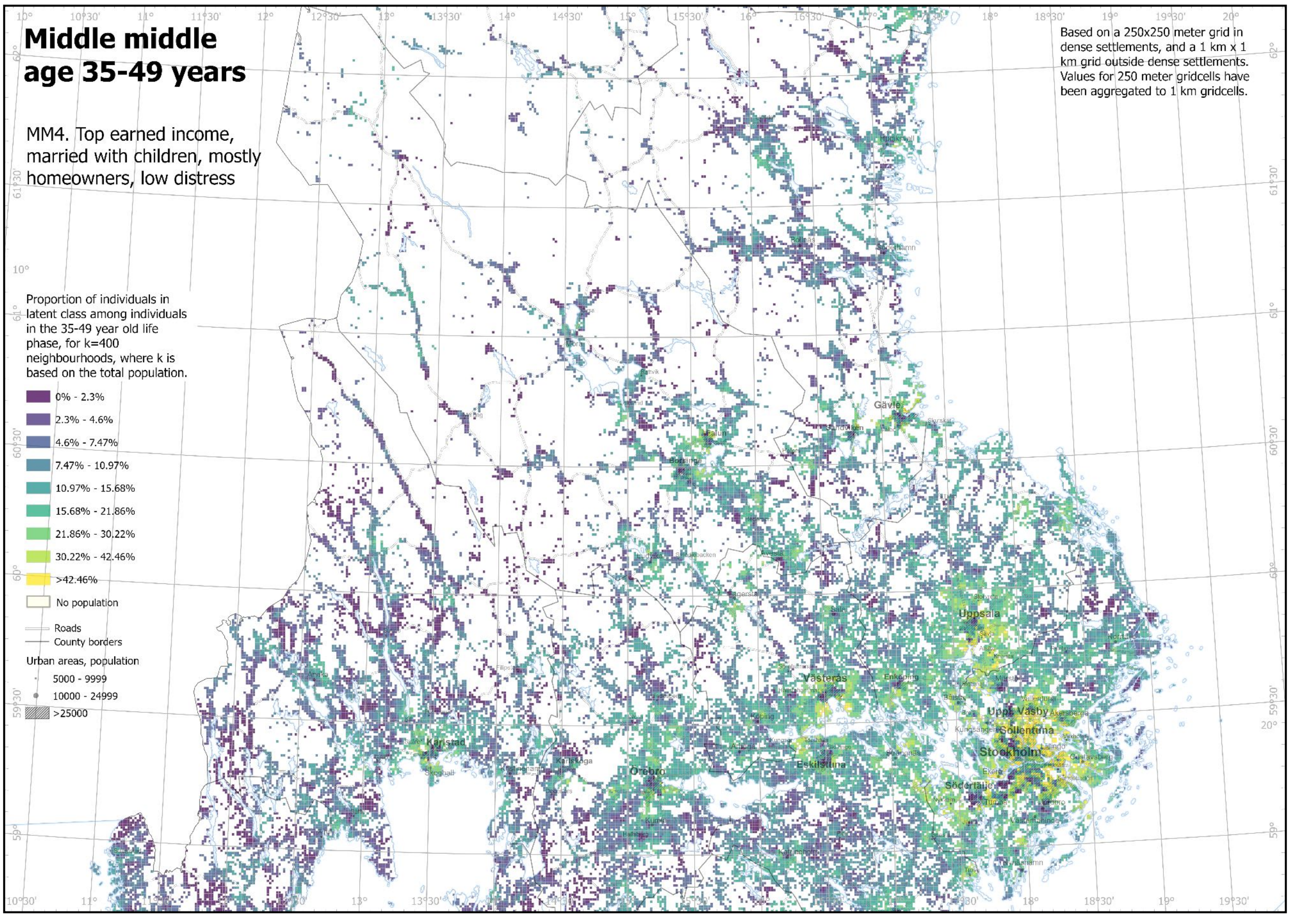

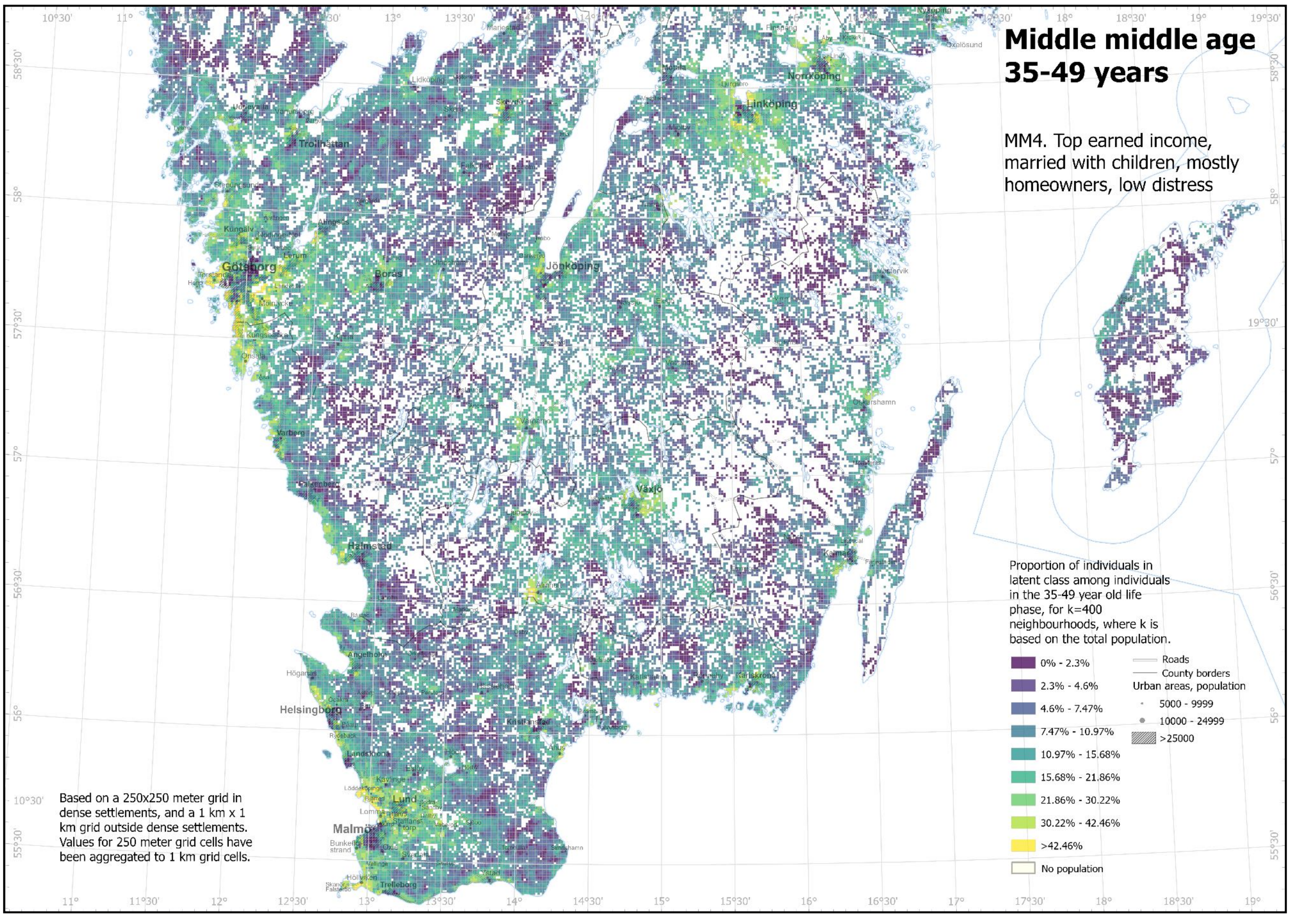

Supplementary Figure S 29 Spatial distribution of advantaged life course MM4, continued

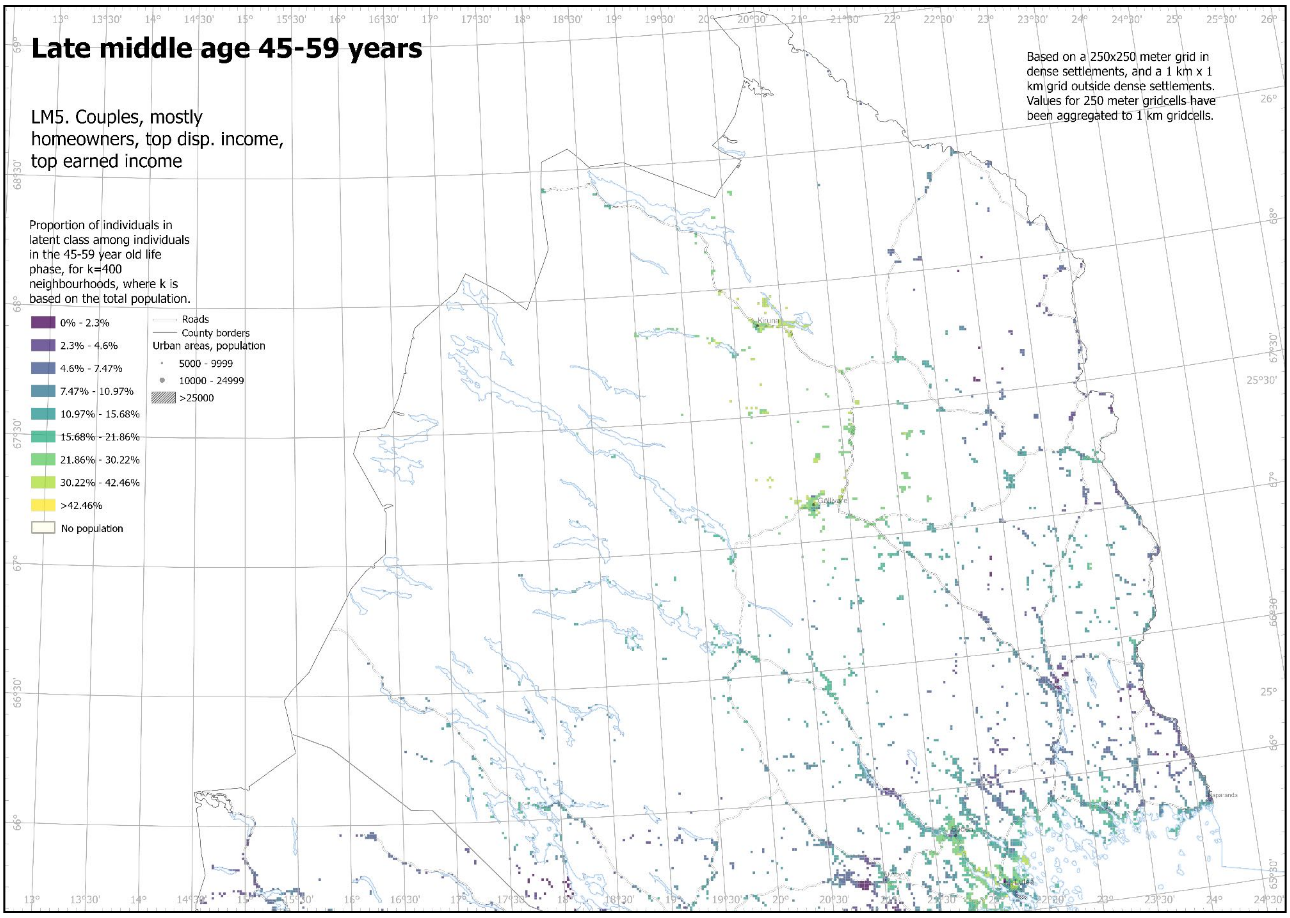

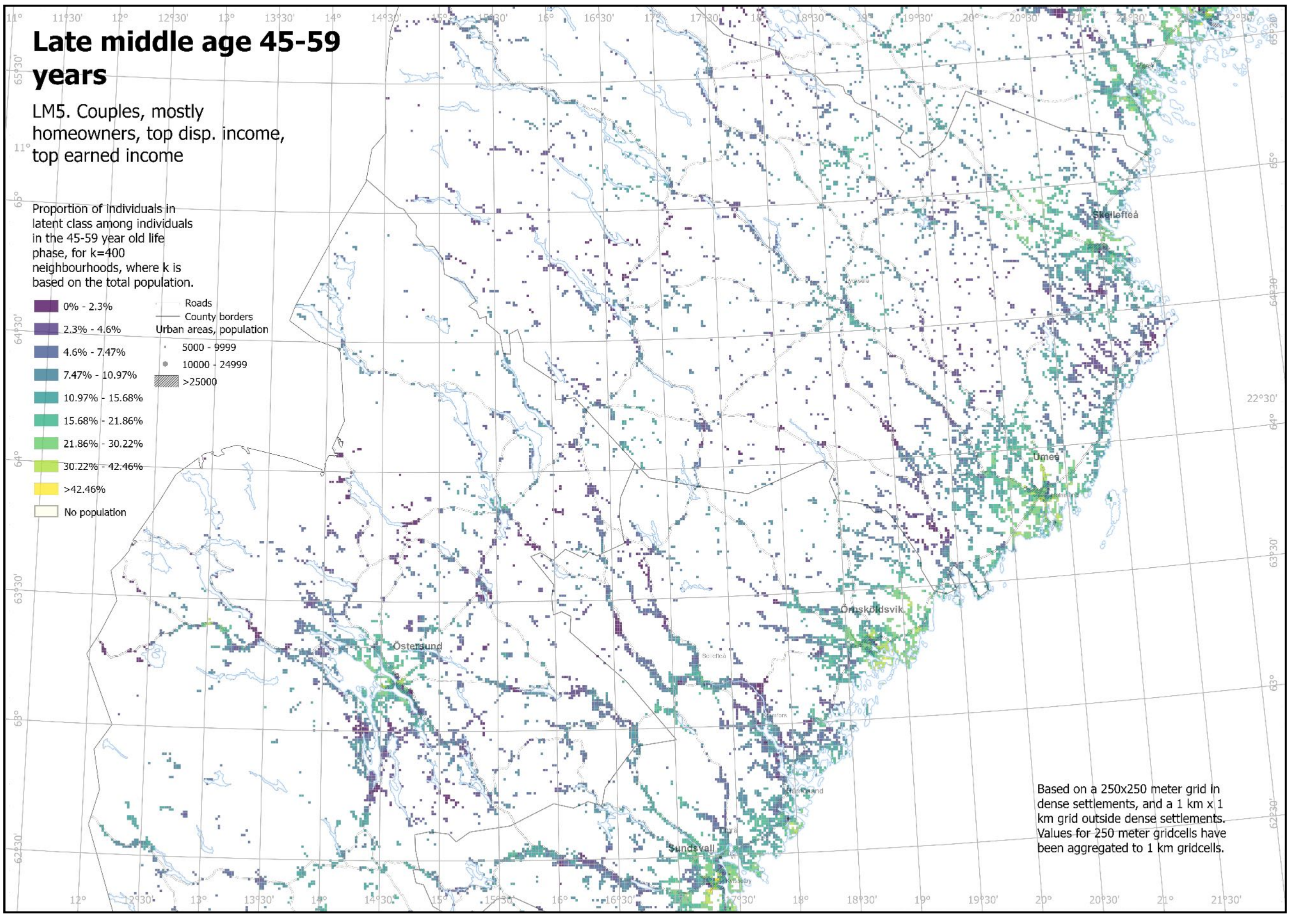

Supplementary Figure S 30 Spatial distribution of advantaged life course LM5

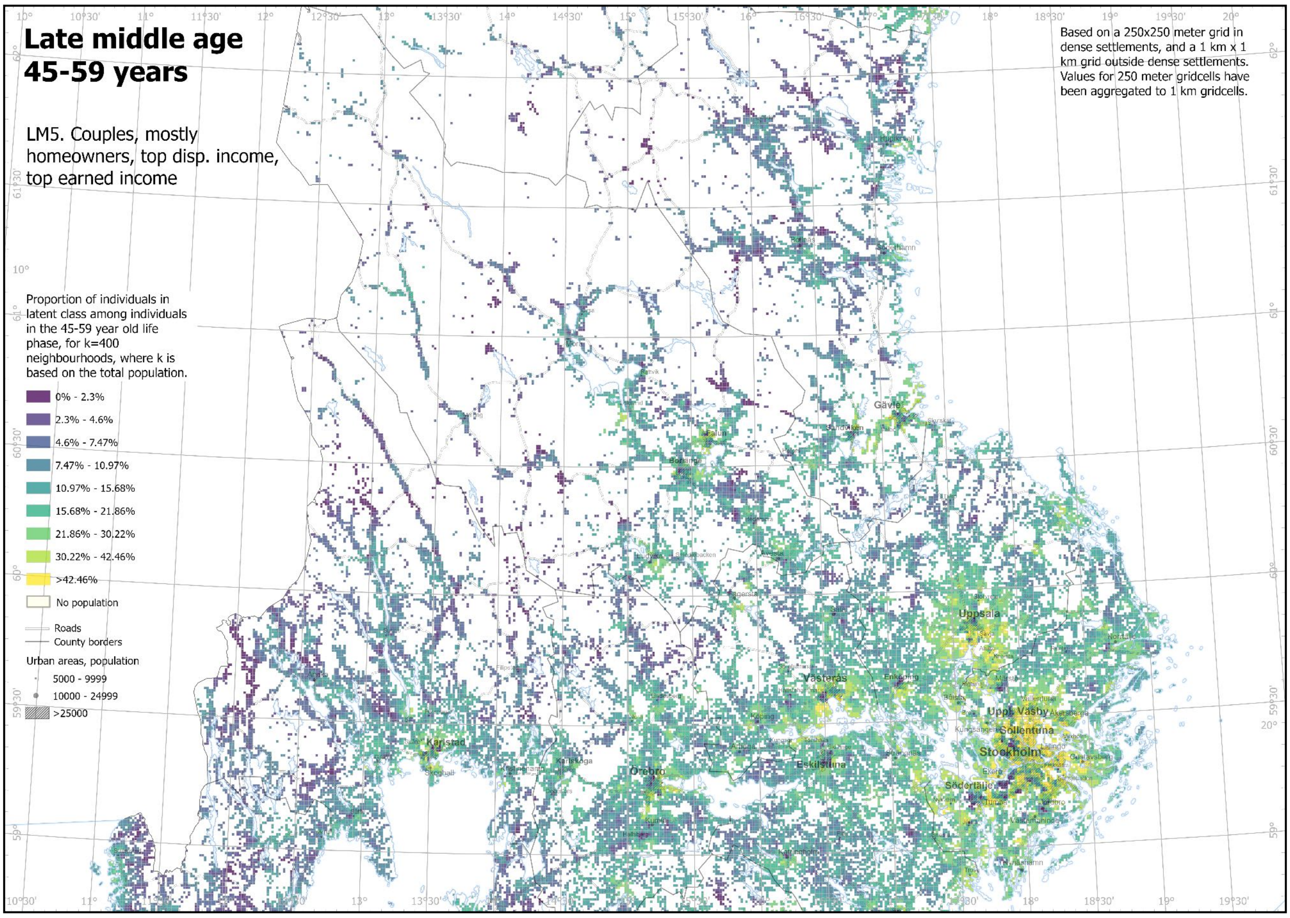

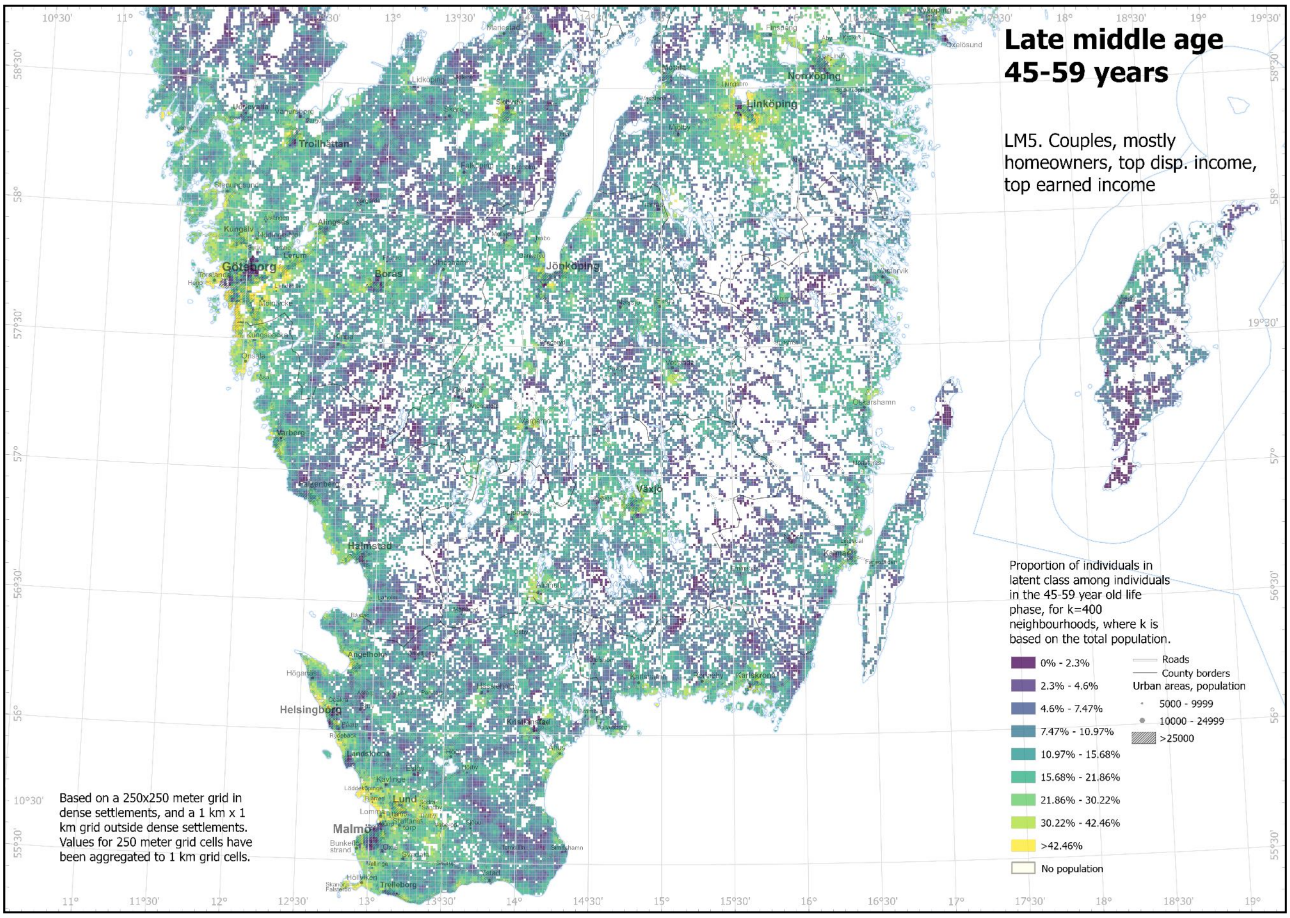

Supplementary Figure S 30 Spatial distribution of advantaged life course LM5, continued

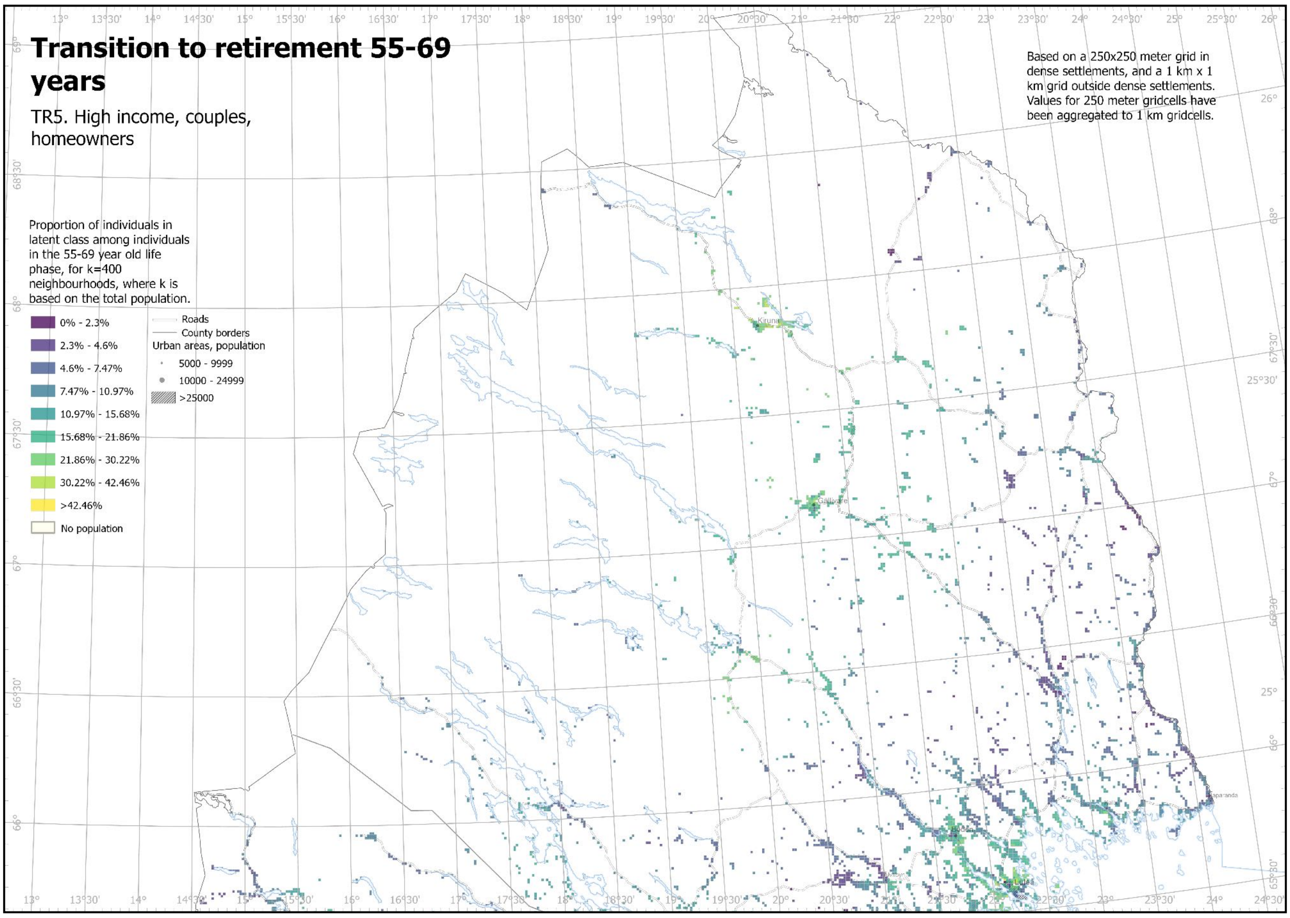

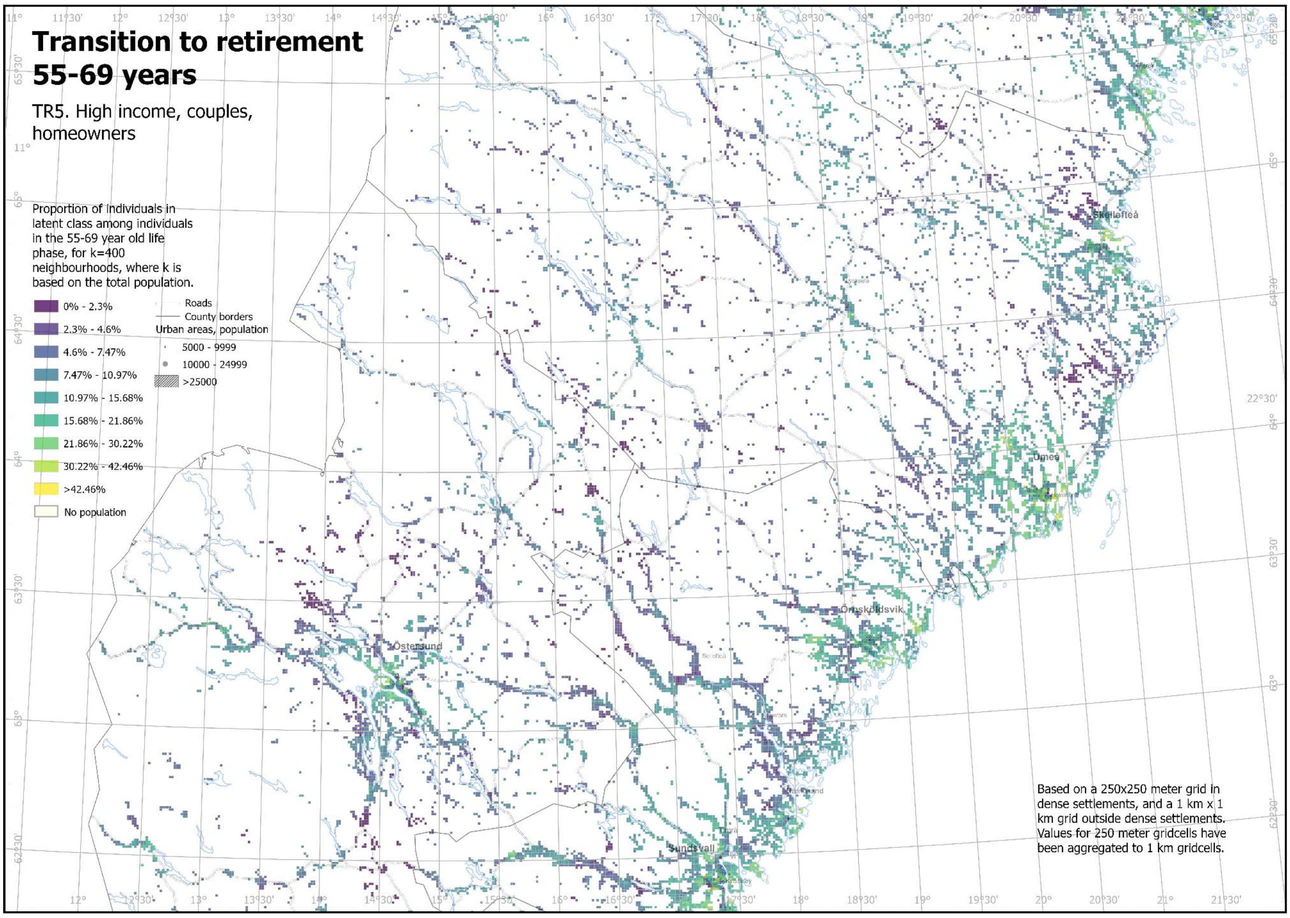

Supplementary Figure S 31 Spatial distribution of advantaged life course TR5

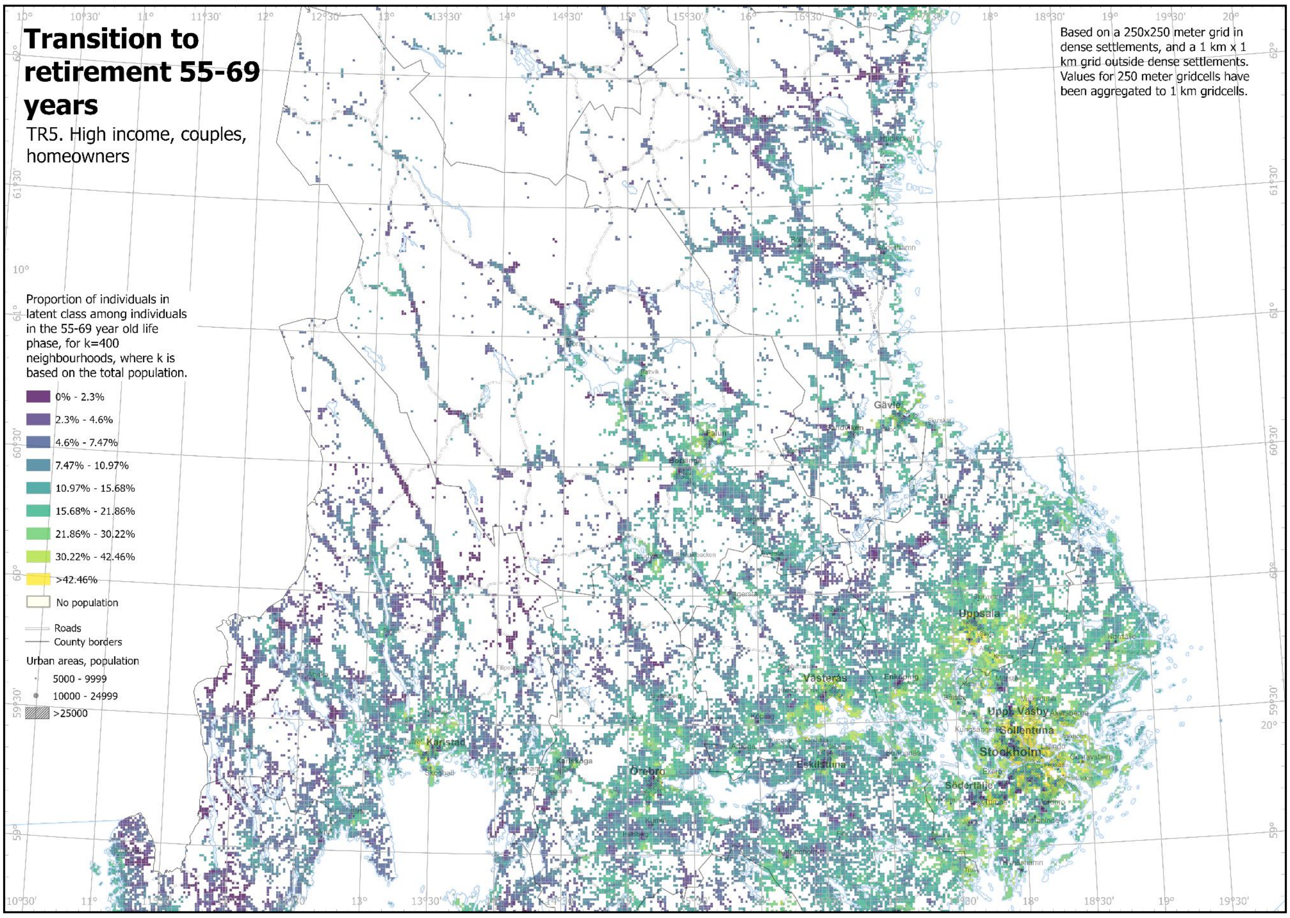

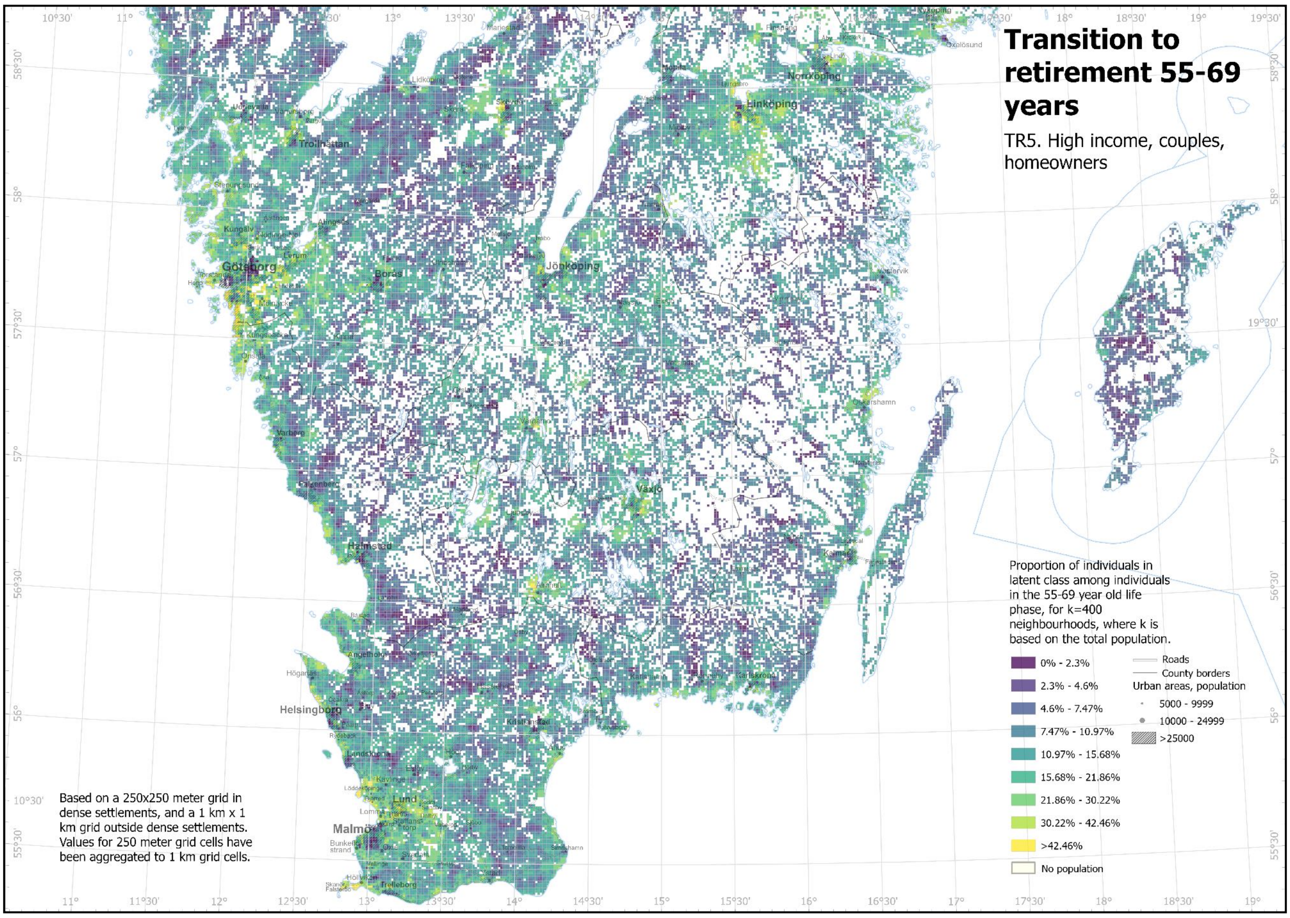

Supplementary Figure S 31 Spatial distribution of advantaged life course TR5, continued

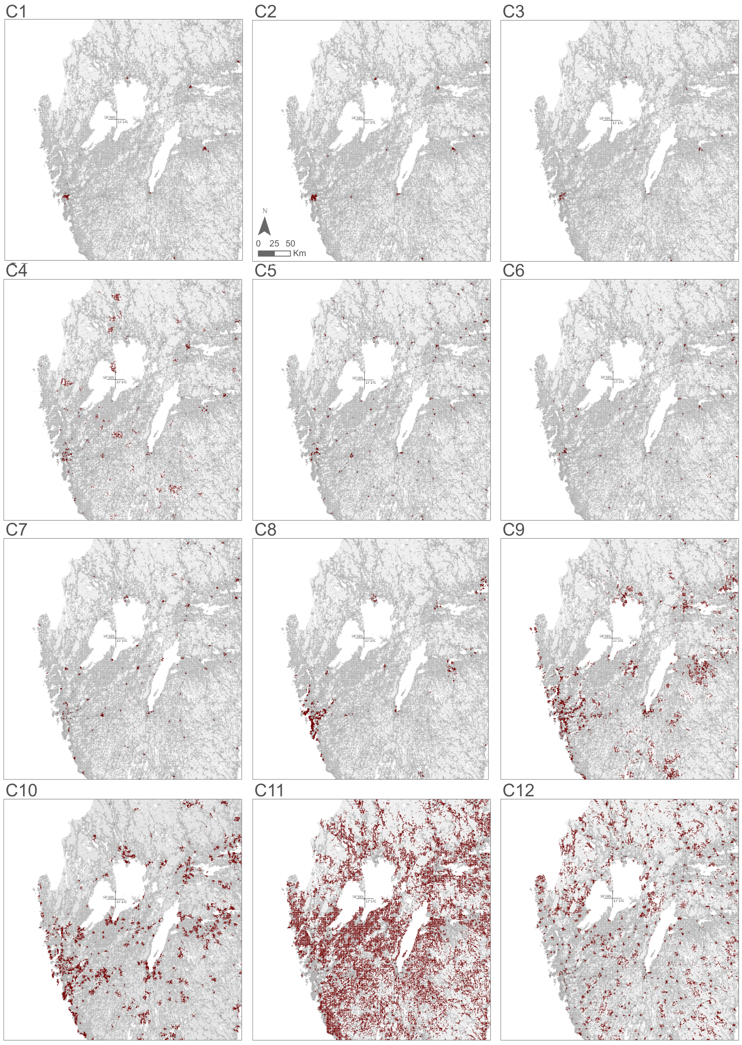

Supplementary Figure 32: Red dots indicate the locations of clusters with different compositions of life-course trajectories in Western Sweden, based on the nearest 400 neighbors. Dark grey areas represent populated grid cells.

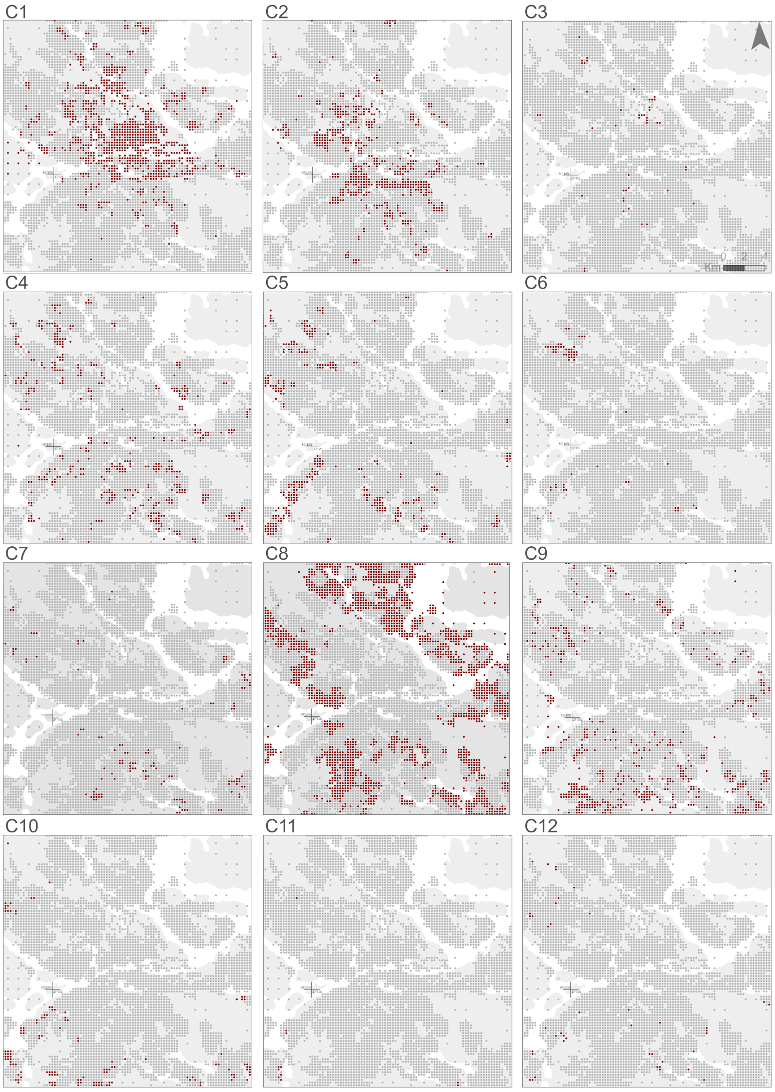

Supplementary Figure 33: Red dots indicate the locations of clusters with different compositions of life-course trajectories in Stockholm, based on the nearest 400 neighbors. Dark grey areas represent populated grid cells.

		Age	Educational domain				Precarity				Affluence		Family status			
			Lower Secondary	Upper Secondary	Short Tertiary	Long Tertiary	Low Earned Income	Labor Market Distress	At Risk of Poverty	Social Benefit	Top Earned Income	Top Disp. Income	Single Family House	Couple	With Child	Child
Disadvantaged	Same		*Latent Class YM1*													
		**25-27**	0.99	0.01	0.00	0.00	0.43	0.27	0.26	0.23	0.02	0.02	0.22	0.39	0.52	0.18
		**28-30**	0.98	0.01	0.00	0.00	0.40	0.29	0.24	0.19	0.03	0.03	0.26	0.46	0.58	0.11
		**31-33**	0.96	0.03	0.00	0.00	0.37	0.29	0.22	0.17	0.04	0.03	0.32	0.52	0.64	0.08
		**34-36**	0.91	0.07	0.00	0.00	0.34	0.28	0.21	0.14	0.06	0.03	0.37	0.56	0.68	0.06
		**37-39**	0.88	0.10	0.01	0.01	0.32	0.27	0.20	0.13	0.07	0.03	0.40	0.57	0.70	0.05
Middle income, homeowner	Same		*Latent Class YM4*													
		**25-27**	0.02	0.98	0.00	0.00	0.19	0.29	0.08	0.04	0.04	0.04	0.38	0.58	0.60	0.15
		**28-30**	0.01	0.99	0.00	0.00	0.18	0.28	0.08	0.03	0.06	0.04	0.51	0.78	0.79	0.07
		**31-33**	0.00	1.00	0.00	0.00	0.16	0.26	0.08	0.02	0.08	0.03	0.62	0.90	0.93	0.04
		**34-36**	0.00	0.99	0.01	0.01	0.14	0.23	0.09	0.01	0.10	0.02	0.69	0.92	0.98	0.03
		**37-39**	0.00	0.96	0.02	0.02	0.12	0.21	0.09	0.01	0.12	0.03	0.71	0.90	0.97	0.02
Advantaged	Same		*Latent Class YM2*													
		**25-27**	0.00	0.06	0.12	0.82	0.23	0.13	0.16	0.02	0.05	0.06	0.17	0.25	0.24	0.13
		**28-30**	0.00	0.01	0.02	0.98	0.15	0.15	0.10	0.01	0.14	0.12	0.23	0.41	0.35	0.06
		**31-33**	0.00	0.00	0.00	1.00	0.11	0.15	0.07	0.01	0.22	0.16	0.36	0.60	0.56	0.03
		**34-36**	0.00	0.00	0.00	1.00	0.09	0.14	0.05	0.00	0.28	0.16	0.49	0.72	0.72	0.02
		**37-39**	0.00	0.00	0.00	1.00	0.07	0.13	0.04	0.00	0.34	0.15	0.58	0.77	0.79	0.01
Middle income, single	Almost same		*Latent Class YM5*													
		**25-27**	0.03	0.97	0.00	0.00	0.24	0.26	0.15	0.12	0.04	0.04	0.14	0.15	0.24	0.14
		**28-30**	0.01	0.99	0.00	0.00	0.25	0.27	0.15	0.11	0.06	0.06	0.14	0.15	0.23	0.08
		**31-33**	0.00	1.00	0.00	0.00	0.26	0.27	0.16	0.11	0.08	0.07	0.16	0.18	0.26	0.05
		**34-36**	0.00	0.99	0.00	0.00	0.27	0.27	0.17	0.11	0.09	0.08	0.19	0.25	0.34	0.04
		**37-39**	0.00	0.98	0.01	0.01	0.28	0.26	0.17	0.11	0.09	0.08	0.24	0.31	0.42	0.03
Disadvantaged	Split		*Latent Class YM3*													
		**25-27**	0.03	0.37	0.48	0.02	0.38	0.21	0.26	0.12	0.03	0.05	0.19	0.33	0.37	0.15
		**28-30**	0.01	0.23	0.53	0.11	0.39	0.21	0.28	0.12	0.07	0.06	0.20	0.41	0.43	0.08
		**31-33**	0.00	0.10	0.48	0.29	0.39	0.20	0.29	0.13	0.10	0.07	0.25	0.50	0.53	0.04
		**34-36**	0.00	0.03	0.42	0.41	0.36	0.20	0.29	0.12	0.13	0.07	0.30	0.57	0.62	0.03
		**37-39**	0.00	0.02	0.38	0.45	0.32	0.19	0.27	0.12	0.16	0.07	0.34	0.62	0.68	0.02

		Age	Educational domain				Precarity				Affluence		Family status			
			Lower Secondary	Upper Secondary	Short Tertiary	Long Tertiary	Low Earned Income	Labor Market Distress	At Risk of Poverty	Social Benefit	Top Earned Income	Top Disp. Income	Single Family House	Couple	With Child	Child
Disadvantaged	Same		*Latent Class YM2*													
		**25-27**	0.99	0.00	0.00	0.00	0.40	0.28	0.23	0.21	0.02	0.03	0.23	0.39	0.51	0.18
		**28-30**	0.99	0.00	0.00	0.00	0.37	0.29	0.21	0.17	0.03	0.03	0.27	0.46	0.57	0.11
		**31-33**	0.98	0.01	0.00	0.00	0.35	0.30	0.20	0.15	0.05	0.03	0.33	0.52	0.63	0.08
		**34-36**	0.95	0.04	0.00	0.00	0.32	0.28	0.19	0.13	0.06	0.03	0.38	0.56	0.67	0.06
		**37-39**	0.91	0.07	0.01	0.01	0.30	0.27	0.18	0.11	0.07	0.03	0.42	0.58	0.70	0.05
Middle income, homeowner	Same		*Latent Class YM4*													
		**25-27**	25-27	0.02	0.98	0.00	0.00	0.19	0.29	0.08	0.04	0.04	0.04	0.38	0.58	0.60
		**28-30**	28-30	0.01	0.99	0.00	0.00	0.18	0.28	0.08	0.03	0.06	0.04	0.51	0.78	0.79
		**31-33**	31-33	0.00	1.00	0.00	0.00	0.16	0.26	0.08	0.02	0.08	0.03	0.62	0.90	0.93
		**34-36**	34-36	0.00	0.99	0.01	0.01	0.14	0.23	0.09	0.01	0.10	0.02	0.69	0.92	0.98
		**37-39**	37-39	0.00	0.96	0.02	0.02	0.12	0.21	0.09	0.01	0.12	0.03	0.71	0.90	0.97
Advantaged	Same		*Latent Class YM5*													
		**25-27**	0.00	0.09	0.13	0.77	0.23	0.14	0.15	0.02	0.04	0.06	0.17	0.25	0.24	0.13
		**28-30**	0.00	0.02	0.05	0.93	0.15	0.16	0.10	0.01	0.13	0.12	0.23	0.40	0.35	0.06
		**31-33**	0.00	0.00	0.01	0.99	0.12	0.16	0.07	0.01	0.21	0.15	0.36	0.59	0.55	0.03
		**34-36**	0.00	0.00	0.00	1.00	0.09	0.15	0.05	0.00	0.26	0.15	0.48	0.71	0.71	0.02
		**37-39**	0.00	0.00	0.00	1.00	0.07	0.13	0.04	0.00	0.32	0.15	0.57	0.76	0.79	0.01
Middle income, single	Almost same		*Latent Class YM6*													
		**25-27**	0.01	0.99	0.00	0.00	0.10	0.25	0.06	0.05	0.05	0.05	0.15	0.12	0.18	0.12
		**28-30**	0.00	0.99	0.00	0.00	0.10	0.25	0.05	0.03	0.09	0.08	0.16	0.12	0.17	0.06
		**31-33**	0.00	1.00	0.00	0.00	0.10	0.25	0.04	0.03	0.11	0.10	0.20	0.17	0.21	0.04
		**34-36**	0.00	0.99	0.01	0.00	0.11	0.25	0.04	0.02	0.13	0.11	0.26	0.26	0.31	0.03
		**37-39**	0.00	0.97	0.01	0.01	0.13	0.24	0.05	0.02	0.14	0.11	0.31	0.33	0.39	0.03
	New		*Latent Class YM4*													
		**25-27**	0.12	0.68	0.04	0.05	0.72	0.22	0.52	0.31	0.00	0.01	0.11	0.35	0.47	0.15
		**28-30**	0.06	0.62	0.06	0.11	0.71	0.23	0.54	0.30	0.00	0.01	0.09	0.39	0.50	0.09
		**31-33**	0.03	0.55	0.07	0.20	0.70	0.24	0.55	0.30	0.00	0.00	0.09	0.43	0.55	0.05
		**34-36**	0.01	0.50	0.05	0.27	0.66	0.25	0.54	0.29	0.00	0.01	0.09	0.46	0.60	0.04
		**37-39**	0.01	0.47	0.04	0.30	0.61	0.25	0.51	0.27	0.01	0.01	0.11	0.49	0.64	0.03
	New		*Latent Class YM1*													
		**25-27**	0.01	0.20	0.77	0.00	0.22	0.18	0.15	0.04	0.06	0.07	0.22	0.30	0.32	0.18
		**28-30**	0.00	0.09	0.88	0.01	0.18	0.19	0.11	0.03	0.14	0.11	0.28	0.41	0.40	0.09
		**31-33**	0.00	0.02	0.94	0.02	0.15	0.19	0.08	0.02	0.21	0.14	0.37	0.54	0.53	0.05
		**34-36**	0.00	0.00	0.91	0.07	0.13	0.17	0.07	0.01	0.28	0.14	0.47	0.63	0.64	0.03
		**37-39**	0.00	0.00	0.88	0.10	0.11	0.15	0.06	0.01	0.35	0.14	0.54	0.68	0.71	0.02
