# Supplementary material for: Life course trajectory segregation and political polarization in contemporary Sweden
Source: Nat Commun. 2026 Jul 30;17:7116. doi: 10.1038/s41467-026-75072-y (PMC13424875; doi:10.1038/s41467-026-75072-y)
Supplement: Supplementary file 3 — Transparent Peer Review file [file 41467_2026_75072_MOESM3_ESM.pdf]

nature portfolio

Peer Review File

Life course trajectory segregation and political polarization in contemporary Sweden

Corresponding Author: Professor Bo Malmberg

This manuscript has been previously reviewed at another journal. This document only contains information relating to versions considered at Nature Communications.

This file contains all reviewer reports in order by version, followed by all author rebuttals in order by version.

Version 1:

Reviewer comments:

Reviewer #1

(Remarks to the Author)
The revised manuscript shows substantial improvement and addresses most of the concerns raised in the previous review round. The authors have addressed most previous concerns regarding theoretical framing, methodological transparency, and presentation; The integration of Bourdieu’s concept of social stratification strengthens the theoretical framing, linking segregation to life-course trajectories. The expanded methodological details, including robustness checks for LCA and rationale for k-nearest neighbor parameters improves methodological clarity. The addition of Sankey diagrams and reworked figures (grayscale) enhances readability. The paper has also been strengthened trough the reorganization and clarification of supplementary materials.

Minor comments:
Remaining suggestions for further strengthening the paper:
1. The introduction is now long and dense and would benefit of being condensed and more streamlined. One suggestion is to further clarify the theoretical contribution of the trajectory approach. While the link between Bourdieu’s concept of social trajectories and the empirical approach is interesting the transition from general segregation literature to the trajectory approach could be explained more explicitly and concisely. Consider adding a short paragraph summarizing why this theoretical lens adds value compared to traditional segregation studies.
2. Although the authors have added some discussion of implications, the interpretation of results remains limited. Consider expanding on what these patterns mean for theory and practice, beyond descriptive findings. For example, Figure 5 shows strong segregation for advantaged and homeowner trajectories after age 40, while younger phases are more mixed. What does this imply for housing market dynamics, social mixing, and long-term inequality? Similarly, the link to political polarization in Figure 7 is presented descriptively. The authors could explain why these spatial patterns might influence voting behaviour and whether these supports or challenges existing theories.

These additions would help readers understand the broader significance of the findings and strengthen the paper’s impact.

3. While policy relevance, linking trajectory composition to political polarization and theoretical implications, now is mentioned the discussion remains very brief. For a journal like Nature Communications, a more explicit and structured discussion of policy implications would strengthen the societal impact of the paper. The discussion could be more explicit about practical implications for urban planning or anti-segregation measures.
4. Figure clarity: Repeat definitions of TA, YM, MM, LM, TR in all relevant figure captions for reader convenience.
5. Figure 5 caption: Update color description to match grayscale shading.

Regarding Sex and Gender Equity (SAGER) guidelines: The study uses register data that includes sex/gender information, but the manuscript does not include sex-disaggregated analyses. This seems appropriate given the study’s focus on life-course trajectories and spatial segregation rather than biological or gender-specific outcomes.

(Remarks on code availability)
No, because no code was provided for review.

The manuscript states that the data are confidential and require ethical approval from Statistics Sweden, which is understandable. However, the journal expects that analysis scripts be shared to ensure reproducibility. Please confirm that code and documentation will be made available in a repository or as supplementary material, even if the data cannot be shared.

Reviewer #2

(Remarks to the Author)
I believe the authors appropriately addressed my comments. I believe the manuscript is much improved.

(Remarks on code availability)

Open Access This Peer Review File is licensed under a Creative Commons Attribution 4.0 International License, which permits use, sharing, adaptation, distribution and reproduction in any medium or format, as long as you give appropriate credit to the original author(s) and the source, provide a link to the Creative Commons license, and indicate if changes were made.
In cases where reviewers are anonymous, credit should be given to 'Anonymous Referee' and the source.
The images or other third party material in this Peer Review File are included in the article's Creative Commons license, unless indicated otherwise in a credit line to the material. If material is not included in the article's Creative Commons license and your intended use is not permitted by statutory regulation or exceeds the permitted use, you will need to obtain permission directly from the copyright holder.
To view a copy of this license, visit https://creativecommons.org/licenses/by/4.0/

Response to Reviewers

We thank the editors and both reviewers for their careful reading of the manuscript and their constructive comments. The revised manuscript has been strengthened considerably in response to these suggestions. Below we address each point in turn. All changes are shown with tracked changes in the manuscript file.

We have substantially restructured the introduction in response to this and the related comments from Reviewer 1 (see below).

Reviewer 1

Comment 1. *The introduction is now long and dense and would benefit of being condensed and more streamlined. One suggestion is to further clarify the theoretical contribution of the trajectory approach. While the link between Bourdieu's concept of social trajectories and the empirical approach is interesting the transition from general segregation literature to the trajectory approach could be explained more explicitly and concisely. Consider adding a short paragraph summarizing why this theoretical lens adds value compared to traditional segregation studies.*

We have reorganised the introduction around a clearer argumentative structure, pp. 1-3. The opening paragraph retains an accessible framing Rather than then working through theoretical background, we now move directly to a statement of the paper's central hypothesis: that spatial polarization results from the uneven geographical distribution of life-course trajectories.

Bourdieu's framework is introduced immediately as the theoretical grounding for this hypothesis rather than as background literature. Critically, the advantage of the trajectory approach over single-indicator classifications is now stated explicitly and early, "trajectories capture multiple dimensions of social position simultaneously and can therefore be expected to be more strongly sorted in space" rather than being distributed tentatively across multiple later paragraphs as in the previous version. A added sentence acknowledges complementary perspectives on trajectory formation. absorbing the key references from the former "formation of distinct life-course patterns" paragraph, which has been removed.

The paragraph on the scope of the paper now closes the introduction's opening movement cleanly, making explicit that we test whether trajectories can be identified and whether they are spatially sorted, and noting that the mechanisms linking spatial concentration to attitude formation remain to be explored in future work.

Comment 2. *Although the authors have added some discussion of implications, the interpretation of results remains limited. Consider expanding on what these patterns mean for theory and practice, beyond descriptive findings. For example, Figure 5 shows strong segregation for advantaged and homeowner trajectories after age 40, while younger phases are more mixed. What does this imply for housing market dynamics, social mixing, and long-term inequality? Similarly, the link to political polarization in Figure 7 is presented descriptively. The authors could explain why these spatial patterns might influence voting behaviour and whether these supports or challenges existing theories. These additions would help readers understand the broader significance of the findings and strengthen the paper's impact.*

On the Figure 5 finding: the observation that segregation sharpens sharply after the homeownership transition is now discussed explicitly in the Discussion section pp.7-8, where we note that the period before age 40 represents a window during which residential mixing is both more common and more tractable. We connect this directly to the policy implications: housing policies that expand access to metropolitan markets for middle-income families, and that enable low-income families to enter areas dominated by middle-income homeowners, would target precisely this transition as the key mechanism driving long-term segregation.

On the Figure 7 finding: we have added a comment in the Discussion: "At the same time, our results show that metropolitan areas are not uniformly segregated: both advantaged and disadvantaged trajectories are represented there, and younger adults in particular tend to inhabit mixed residential environments to a higher degree than after age 35 when many transition to homeownership. This suggests that the period before age 40 represents a window during which residential mixing is both more common and more tractable". p.8. It is also commented in the policy recommendation, see below.

Comment 3. *While policy relevance, linking trajectory composition to political polarization and theoretical implications, now is mentioned the discussion remains very brief. For a journal like Nature Communications, a more explicit and structured discussion of policy implications would strengthen the societal impact of the paper. The discussion could be more explicit about practical implications for urban planning or anti-segregation measures.*

We have added a new closing paragraph to the Discussion making the policy implications explicit, p.8 . Three points are made. First, studies confined to metropolitan areas will systematically underestimate segregation, because the most homogeneous clustering of middle-income homeowner trajectories occurs in semi-peripheral regions. Second, metropolitan areas are not uniformly segregated — both advantaged and disadvantaged trajectories are represented there, and younger adults in particular inhabit more mixed environments before homeownership transitions harden spatial boundaries. Third, this mixing window before age 40 suggests that housing policies targeting the homeownership transition — subsidies or tenure-mix requirements directed at middle- and low-income families — could help sustain the mixing that metropolitan areas currently provide to younger adults into later life phases. We have also added a sentence to the Geographical patterns section noting that the metropolitan-non-metropolitan gradient is more continuous than a simple urban-rural dichotomy suggests, grounding the policy argument empirically before it is developed in the Discussion.

Comment 4. *Repeat definitions of TA, YM, MM, LM, TR in all relevant figure captions for reader convenience.*

We have added full definitions of all life phase abbreviations (Transition to adulthood, TA; Young middle age, YM; Middle middle age, MM; Late middle age, LM; Transition to retirement, TR) on first use in the captions for Figures 2, 5, 7, and 8.

Comment 5. *Figure 5 caption: update color description to match grayscale shading.*

The caption for Figure 5 has been updated. "High values in red, low values in green" has been replaced with "darker shading indicates higher separation values; lighter shading indicates lower separation values."

Comment 6 *Regarding Sex and Gender Equity (SAGER) guidelines: The study uses register data that includes sex/gender information, but the manuscript does not include sex-disaggregated analyses. This seems appropriate given the study's focus on life-course trajectories and spatial segregation rather than biological or gender-specific outcomes.*

Figure 3 reports sex composition of trajectories. We have added a comment on p. 4: "**Error! Reference source not found.** also shows that the gender composition of the trajectories changes across life phases, with men becoming increasingly dominant in the affluent trajectories. This suggest that in the flows depicted in Figure 2 there can be a gender imbalance for flows into or away from affluence."

Comment 7 (code availability). *The manuscript states that the data are confidential and require ethical approval from Statistics Sweden, which is understandable. However, the journal expects that analysis scripts be shared to ensure reproducibility. Please confirm that code and documentation will be made available in a repository or as supplementary material, even if the data cannot be shared..*

We have added a Code Availability statement to the manuscript: "The code used for this paper is available at https://github.com/bmalmberg/Life-course-trajectory-landscapes."

Reviewer 2

I believe the authors appropriately addressed my comments. I believe the manuscript is much improved.

We thank Reviewer 2 for this positive assessment.
